# Detailed Characterization of T Cell Receptor Repertoires in Multiple Sclerosis Brain Lesions

**DOI:** 10.3389/fimmu.2018.00509

**Published:** 2018-03-19

**Authors:** Raquel Planas, Imke Metz, Roland Martin, Mireia Sospedra

**Affiliations:** ^1^Neuroimmunology and MS Research (nims), Department of Neurology, University Zurich, Zürich, Switzerland; ^2^Institute of Neuropathology, University Medical Center Göttingen, Göttingen, Germany

**Keywords:** CD4+ T cell, CD8+ T cell, T cell receptor, multiple sclerosis, brain lesions

## Abstract

The antigen-specific activation of pathogenic T cells is considered essential in the initiation and maintenance of multiple sclerosis (MS). The site of activation, the differential involvement of CD4+, and CD8+ T cells, their functional phenotype, and specificity, are important aspects to understand MS pathogenesis. The analysis of clonal expansions of brain-infiltrating T cells may reveal local antigen-driven activation or specific brain homing and allow the identification of putatively pathogenic T cells. We used high-throughput T cell receptor β-chain variable gene (TRBV) sequencing (-seq) of genomic (g)DNA, which reflects the quantity and diversity of the TRBV repertoire, to characterize three white matter demyelinating lesions with different location and inflammatory activity, and paired peripheral blood memory CD4+ and CD8+ T cell pools from a secondary progressive (SP)MS patient. Our results revealed an important sharing of clonally expanded T cells with identical TRBV sequence (clonotypes) across MS lesions independently of their proximity or inflammatory activity. Comparison with circulating T cells showed that the most frequent brain-infiltrating CD8+, but not CD4+ clonotypes were also those with highest frequency in the peripheral blood, indicating clonal expansion inside the brain or specific brain homing of CD4+ but not CD8+ T cells. Parallel TRBV-seq of complementary (c)DNA that reflects the activation status of the cells, revealed differences between lesions regarding inflammatory activity and appears to facilitate the identification of putatively pathogenic T cells in active lesions. Approaches to identify pathogenic T cells in brain lesions using TRBV-seq may benefit from focusing on lesions with high inflammatory activity and from combining gDNA and cDNA sequencing.

## Introduction

Autoimmune inflammation in early multiple sclerosis (MS) is primarily mediated by adaptive immune responses and involves autoreactive T cells and macrophages in patients with demyelinating pattern I active brain lesions and in addition antibody/complement complexes in patients with demyelinating pattern II active lesions (1). The efficacy of therapies targeting B and T cells strongly supports a role of these cells in MS pathogenesis (2). Regarding T cells, MS is considered a CD4+ T cell-mediated autoimmune disease based on the fact that the HLA-DR15 haplotype is the strongest genetic risk factor, that myelin-specific CD4+ T cells are able to induce a demyelinating disease similar to MS in several experimental animal models (3) and also to exacerbate the human disease (4). However, the fact that the HLA-class I alleles HLA-A*0301 and HLA-A*0201 are risk-increasing or protective, respectively (5, 6), and the greater abundance and invasiveness of CD8+ T cells in acute and chronic MS lesions (7, 8), also support a role of these cells in MS.

T cells recognize complexes of specific antigenic peptides and autologous HLA molecules through their T cell receptors (TCRs), and this recognition can lead to clonal expansions by activation and proliferation of antigen-specific T cells. Analysis of T cell clonal expansions in MS brain tissue can allow the identification of T cells that have been activated locally or that have been specifically attracted to the brain and hence are likely pathogenic. In the past, several techniques have been employed for the analysis of TCR repertoires including PCR amplification of TCR β-chain variable genes (TRBV) and subsequent conventional sequencing, TCR complementary-determining region 3 (CDR3) length spectratyping, single strand conformational polymorphisms or flow cytometry-based staining with TRBV-specific monoclonal antibodies. Using these approaches, several studies have demonstrated skewed TCR repertoires and also clonal expansions of mainly CD8+ T cells with identical TRBV sequence (clonotypes) in the peripheral blood, cerebrospinal fluid (CSF), as well as brain lesions of MS patients (9–17). However, these techniques only permit interrogation of a small fraction of sequences and thus are not reliable for characterization of the entire repertoire. High-throughput TRBV sequencing (TRBV-seq) (18) overcomes this limitation by allowing sequencing of very high number of TCRs and enabling the accurate identification and quantification of individual clones in complex populations. This technique has already been used successfully to measure the renewal of the T cell repertoire in MS patients after autologous stem cell transplantation (19), to assess the diversity and overlap of CSF and blood T cell repertoires (20, 21), to identify clonotypes that are clonally expanded in pattern II MS brain lesions (22), and also to compare the TRBV repertoires between brain lesions, CSF, and blood from the same MS patients (23).

TRBV sequencing of MS brain lesions that differ in location and inflammatory activity and come from a single MS patient may facilitate the identification of pathogenic T cells and provide information about how lesion location or inflammatory activity influence brain-infiltrating TRBV repertoires. Furthermore, TRBV-seq of paired peripheral CD4+ and CD8+ memory T cell pools allows to assign brain-infiltrating T cells to either CD4+ or CD8+ subtypes and to compare their relative representation in the two compartments. This step will be useful to address the assumed predominant implication of CD8+ T cells in MS pathology as previously suggested (23). Despite the broader use of TRBV-seq in various immune conditions including MS, the method is still relatively new and technical questions, including whether parallel gDNA and cDNA sequencing might improve the characterization of TRBV repertoires in brain lesions, remain to be addressed. Since each cell should have only one copy of the productively rearranged V(D)J gDNA, the number of TRBV gDNA sequences should correlate well with cell numbers (24), and consequently gDNA TRBV-seq should reflect the quantity and overall diversity of the global TRBV repertoire. In addition, gDNA is very stable, an important feature in the analysis of brain tissue that cannot always be frozen immediately after death/biopsy. Assuming the expression of multiple copies of TRBV mRNA per T cell and that the number of copies reflects the activation/functional status of the T cells, i.e., higher number of copies in activated and replicating cells (25–28), cDNA TRBV-seq should be more sensitive and reflect the functional status of the cells. Since frozen brain lesions should represent a snapshot of the *in vivo* T cell activity at the time of autopsy/biopsy, TRBV-seq of cDNA might facilitate the identification of pathogenic T cells in brain lesions. However, the higher vulnerability of RNA to degradation might jeopardize cDNA sequencing in frozen brain tissue.

In this study, we have characterized by gDNA TRBV-seq the TRBV repertoire of three white matter demyelinating MS lesions, as well as paired peripheral memory CD4+ and CD8+ T cells. The three demyelinating lesions that had different location and inflammatory activity, were obtained from a secondary progressive (SP)MS patient with pattern II demyelinating lesions (22), for whom we had the unique opportunity to have access to peripheral blood mononuclear cells (PBMCs) and CSF cells prior to death and also autopsy brain tissue. In order to improve the characterization of the TRBV repertoire we performed cDNA TRBV-seq of the three lesions as well as *in vitro* expansion of brain-infiltrating T cell clones (TCCs) obtained from autologous CSF.

## Materials and Methods

### Patient Material

#### MS Case 1

The SPMS patient with pattern II demyelinating lesions had previously been described (22). PBMCs, CSF-derived mononuclear cells, and autopsy brain tissue were obtained from this patient as previously described (22).

#### MS Case 2

The SPMS patient with pattern III demyelinating lesions had previously been described (22).

#### Rasmussen Encephalitis (RE) Case

The RE patient had previously been described (29).

Brain-derived mononuclear cells from brain biopsies (MS case 2 and RE case) were obtained as previously described (30).

The study of MS clinical cases 1 and 2 was approved by the Ethik Kommission der Ärztekammer Hamburg, protocol No. 2758, and informed consent was obtained from the patient or relatives. The study of the RE case was approved by the Cantonal Ethics Committee Zurich (No. 33-2015), and informed consent was obtained accordingly from the parents.

### Cell Isolation, -Culture, and Generation of CSF-Derived T Cell Clones (CSF-TCCs)

All cell populations were sorted using a FACSAria™ III (BD Biosciences, Franklin Lakes, NJ USA), and only preparations with a purity of >95% were used for further experiments. Memory CD4+ and CD8+ T cells were sorted from peripheral blood after staining with the following antibodies: anti-CD3-PE (Biolegend, San Diego, CA, USA), anti-CD4-APC (eBiosciences, San Diego, CA, USA), anti-CD8-Pacific Blue (Biolegend), and anti-CD45RO FITC (Biolegend) as previously described (22). T cells expressing specific TRBV families were sorted from bulk phytohemagglutinin-expanded CSF T cells after staining with the corresponding TRBV-specific antibodies (Beckman Coulter, Nyon, Switzerland), expanded and cloned as previously described (22).

### DNA/RNA Extractions and Retrotranscription

DNA and RNA were extracted from cryopreserved cells and frozen brain tissue. DNA extraction was performed with DNeasy blood & tissue kit (QIAGEN, Hilden, Germany) according to the manufacturer’s instructions. Quantity and purity were measured using the NanoDrop ND-1000 spectrophotometer. RNA extraction, including a DNAse treatment step, was performed with RNeasy Micro (QIAGEN) following manufacturer’s instructions. RNA integrity was assessed by capillary electrophoresis (Bioanalyzer, Agilent Technologies Inc., Santa Clara, CA, USA). RNA was reverse transcribed using RevertAid H minus first strand cDNA synthesis kit (Thermo Scientific Fermentas, Vilnius, Lithuania).

### TRBV Sequencing

TRBV sequencing was performed at Adaptive Biotechnologies (Seattle, WA, USA) using the immunoSEQ platform (18). TRBV-seq survey level, designed to sample <100,000 cells, was used to sequence the brain lesions (both gDNA and cDNA) since we knew that the number of CD3+ T cells was low [<70 cells/mm^2^ (22)], and TRBV-seq deep sequencing, designed to sample ~200,000–1,000,000 cells, was used to sequence the circulating CD4+ and CD8+ memory T cell pools.

TRBV chain expression by CSF-derived, individual, *in vitro*-generated (CSF-) TCCs was assessed as previously reported (22). TCR gene designations are in accord with IMGT nomenclature (ImMunoGeneTics^1^). Throughout the manuscript clonotype refers to T cells with identical TRBV sequence while CSF-TCC stands for *in vitro*-generated T cell clone.

### Data and Statistical Analysis

Data analysis was performed using ImmunoSEQ analyzer. Statistical analysis was performed using Graphpad Prism 6 software (La Jolla, CA, USA). Correlations were assessed with linear regression analysis and the Pearson test. Multiple comparisons were assessed by one-way ANOVA with Bonferroni’s correction for multiple comparisons. *P*-values < 0.05 were considered statistically significant.

## Results

### Description of MS Lesions

**Figure 1:**
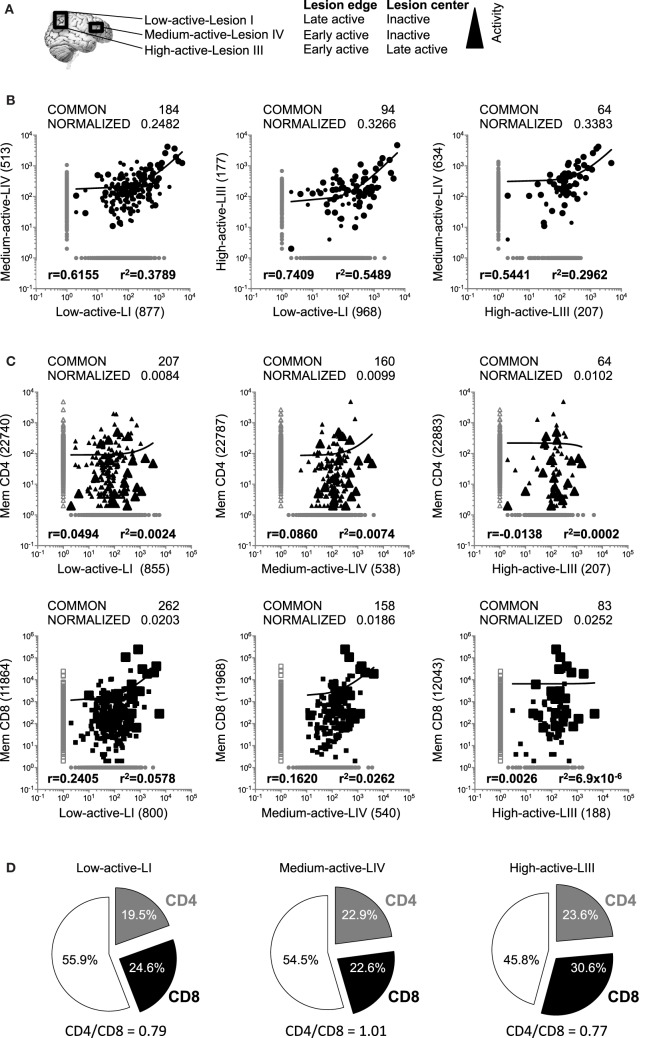
Comparison of the global TRBV repertoires in brain lesions and CD4+ and CD8+ peripheral memory T cell pools. **(A)** Scheme of the three brain lesions analyzed in this study. Location and activity levels of the three lesions are also specified. **(B,C)** Logarithmic scatter plots comparing the number of copies of each clonotype identified by gDNA sequencing and infiltrating each pair of brain lesions **(B)**, or infiltrating each lesion and the CD4+ (upper scatters) and CD8+ (lower scatters) peripheral memory T cell pools **(C)**. Clonotypes are represented by circles, CD4+ clonotypes by triangles and CD8+ clonotypes by squares. Clonotypes not shared between lesions or peripheral pools are shown in gray and shared clonotypes in black. Large symbols represent the 50 clonotypes shared by the three lesions. Actual and normalized numbers of shared clonotypes as well as correlation coefficient (*r*) and coefficient of determination (*r*^2^) of their frequencies are shown in all scatters. Numbers of clonotypes not shared by the different samples are also shown in parenthesis. **(D)** Pie charts representing the frequencies of CD4+ (in gray), CD8+ (in black) and undetermined, i.e., not identified in the peripheral pools, (white) clonotypes in the global T cell repertoires in each lesion.

Three white matter demyelinating lesions were identified in autopsy brain tissue from a SPMS patient with pattern II demyelinating active lesions (22). Lesion (L)I and LIII were in closer proximity, while LIV was more distant. On the basis of myelin degradation products present within the macrophages/microglia, lesions were classified as follows: LI: center inactive and edge late active, LIII: center late active and edge early active (22), and LIV: center inactive and edge early active. In order to facilitate reading the manuscript, lesions are named: low-active-LI, medium-active-LIV, and high-active-LIII (Figure 1A).

### Comparison of the Global TRBV Repertoires in Demyelinating Brain Lesions

We analyzed the global TRBV repertoires in the three brain lesions by gDNA survey TRBV-seq (18). Sequence information is summarized in Table 1, and a complete list of all sequences is available in Table S1 in Supplementary Material. Although we cannot completely discard that we missed relevant unique reads performing survey sequencing instead of deep sequencing in the brain lesions, the fact that only around 0.5–0.7% of the productive sequences were unique, i.e., they were unique TRBV sequence generated by immunosequencing of a given sample (Table 1), suggests that most of the sequences were sequenced many times, and hence it is unlikely that we missed relevant ones. The diversity and clonality were comparable between the three lesions, independently of their location or activity level. A search using BLASTP program of the non-redundant protein database^2^ demonstrated that five CDR3 sequences identified in low-active-LI, two in medium-active-LIV and one in high-active-LIII matched CDR3 sequences in the database but corresponded to TCRs with different -BV and -BJ sequences. Clonotypes shared by the different pairs of lesions as well as correlations of their frequencies [correlation coefficient (*r*) and coefficient of determination (*r*^2^)] are shown in Figure 1B. The total number of sequences that are compared influences the number of clonotypes shared by two samples, i.e., the probability to find shared sequences increases with the number of sequences that are compared [(31); Figure S1A in Supplementary Material]. The number of sequences in each sample is called cloneset size. To correct for different cloneset sizes, the number of shared clonotypes has been divided by the product of the intersected cloneset sizes, and these data are shown as normalized values in all comparisons. The normalized number of shared clonotypes and the correlation of their frequencies do not seem to be affected by the proximity of the lesions or their inflammatory activity level (Figure 1B). Fifty clonotypes were common to the three lesions, and interestingly several were among the most frequent clonotypes in the three lesions (big circles Figure 1B; Table 2). Seven of the ten most frequent clonotypes in low-active-LI were common to the three lesions, eight of the ten most frequent clonotypes in medium-active-LIV and nine of those in high-active-LIII (Table 2). The presence in the other lesions of the 10 most frequent clonotypes identified in each lesion is shown in Table S2 in Supplementary Material. In order to estimate better the overlap of the three lesions, we compared the TRBV repertoires obtained by sequencing two independent gDNA extractions from high-active-LIII (Figure S1B in Supplementary Material). This analysis gave a normalized number of shared clonotypes of 0.51, *r* of 0.91 and *r*^2^ of 0.82. Comparison of these values with those obtained for the pair comparisons of the three lesions suggests a high TRBV sequence overlap between the three brain samples. Also, as controls, we analyzed the global TRBV repertoires in two additional brain biopsies: one from a patient with RE (biopsy RE) and one from a patient with MS (biopsy MS case 2). Sequence information is summarized in Table 1. As expected, the number of shared clonotypes between the three brain lesions and the control brain biopsies was very low and did not include any of the 50 clonotypes common to the three brain lesions (Figure S1C in Supplementary Material). We also analyzed the presence of the 50 T cell clonotypes common to the three brain lesions in peripheral blood of a cohort of 25 MS patients and 17 HD that had previously been reported (32) (Table S3 in Supplementary Material). The frequency of MS patients, who share in their peripheral blood one of these clonotypes, was higher than the frequency of HD (Table S3 in Supplementary Material). 48% of MS patients have in their peripheral blood at least one CD8+ clonotype that is shared by the three demyelinating lesions and 28% of patients at least one CD4+ clonotype, while only 23.5% of HD have at least one CD8+ clonotype in their peripheral blood that is shared by the three demyelinating lesions, and 11.7% at least one CD4+ clonotype. The fact that the TCR repertoire is influenced by the MHC and that MS shows a strong association with certain HLA-class II and also -class I molecules, suggests that a MHC bias might be behind this higher overlap in MS patients. Only 8 of the 50 clonotypes shared by the three lesions (16%) were shared by at least one MS patient or HD. Two of these shared clonotypes, the CD4+ clonotype-7 and the CD8+ clonotype-27, were the most shared clonotypes between MS patients but also HD, suggesting that these clonotypes may be public TCCs, which are commonly found in donors disregarding pathology or MHC background (33). Supporting this idea, these clonotypes were frequent in the naïve T cell pools of HD (Table S3 in Supplementary Material). Furthermore, when the 50 clonotypes, which are shared by the three lesions, were compared with a list of 10,000 public clones previously reported (34), no shared clonotype was identified, but interestingly, the CDR3 regions of the most shared clonotypes, clonotype-7 and clonotype-27, matched two sequences from public clones, although these have different -BV and -BJ sequences.

**Table 1:** Summary of TRBV sequence information.

	Productive sequences (%)*	Unique productive sequences (%)**	Unique productive sequences (#)***	“Diversity” shannon entropy****	Clonality
Low-active-LI (gDNA)	81.50	0.7527	1,062	8.8686	0.1239
Medium-active-LIV (gDNA)	74.56	0.5425	698	8.6789	0.0994
High-active-LIII (gDNA)	65.89	0.5364	271	7.2071	0.1221
BIOPSY RE (gDNA)	76.59	0.3399	790	6.3813	0.3049
BIOPSY MS 2 (gDNA)	78.44	0.8291	2,054	8.1911	0.2185
MEM-CD4 (gDNA)	81.62	5.2018	22,947	13.0884	0.0967
MEM-CD8 (gDNA)	85.87	0.8038	12,126	8.9509	0.3405
Low-active-LI (cDNA)	90.36	2.1277	92	5.9415	0.1038
Medium-active-LIV (cDNA)	76.59	1.0060	237	7.4685	0.0597
High-active-LIII (cDNA)	95.16	0.0152	115	3.0875	0.6453

*Productive sequences (%) have been calculated as: number of productive sequences/total sequences × 100.

**Unique productive sequences (%) have been calculated as: number of unique productive sequences/total productive sequences × 100.

***Number of unique TRBV sequence generated by immunosequencing of a given sample.

****Shannon entropy is one of the most robust measurements of diversity within a complex data set. Shannon entropy is calculated by summing the frequency of each clonotype times the log (base 2) of the same frequency over all productive reads in a sample.

**Table 2:** Clonotypes shared by the three brain lesions.

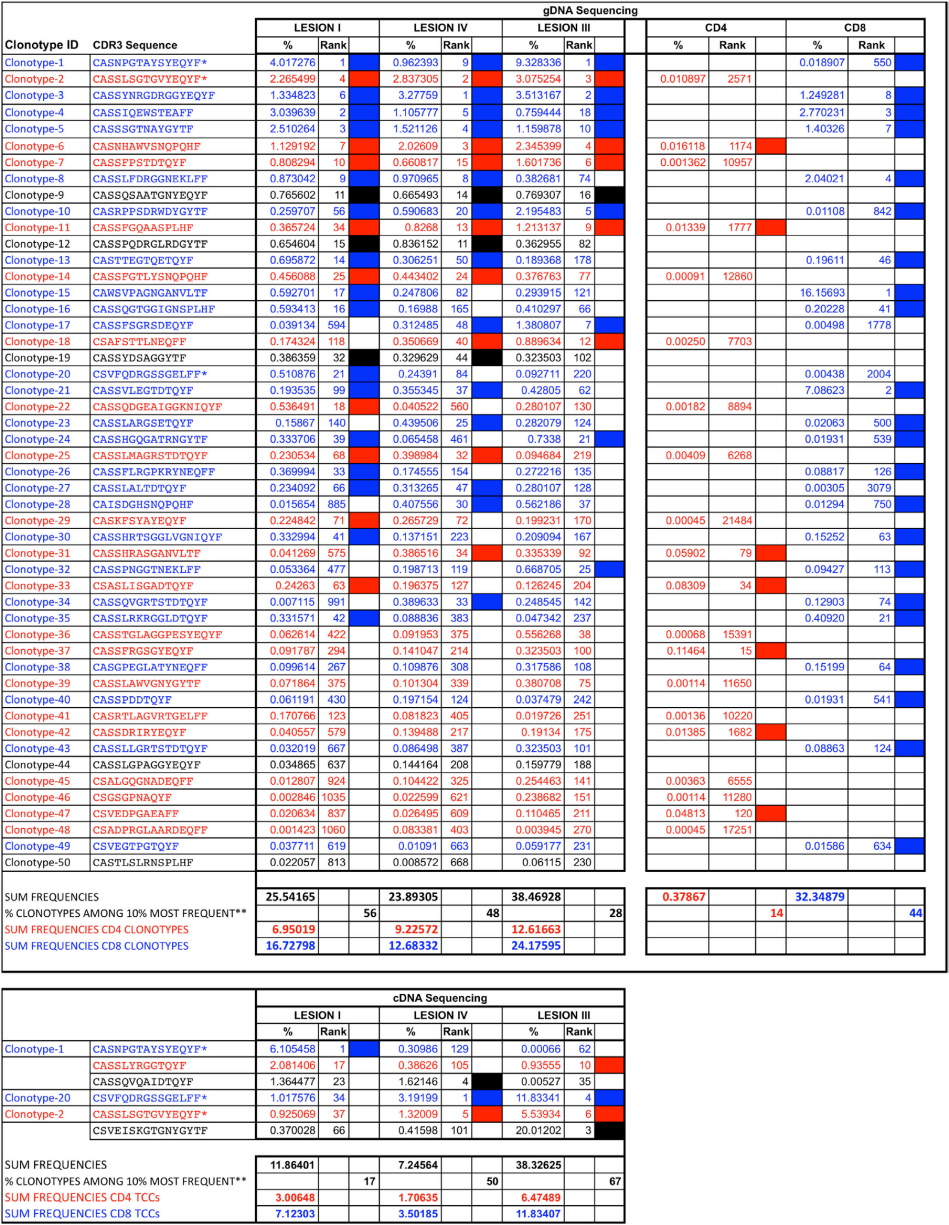

*CD4+ clonotypes are depicted in red and CD8+ clonotypes in blue*.

*Clonotypes identified in the three brain lesions using both gDNA and cDNA sequencing. %, frequency of each clonotype in each sample. Rank, position of the clonotype regarding its frequency compared with all clonotypes in the sample. Filled cells, clonotypes ranking among the 10% most frequent clonotypes in each sample.

**Frequency of the 50 clonotypes shared by the three lesions that are among the 10% most frequent clonotypes in each lesion.

### Comparison of the Global TRBV Repertoires in Demyelinated Brain Lesions and Peripheral Memory T Cell Pools

In order to explore whether brain infiltrates are primarily CD4+ or CD8+ T cell-derived and how brain-infiltrating T cells are represented in the periphery, we performed gDNA TRBV-seq of 2 × 10^5^ purified peripheral memory CD45RO+ CD4+ (mem-CD4+), and 2 × 10^5^ CD8+ (mem-CD8+) cells. Sequence information is summarized in Table 1. As expected, mem-CD8+ cells showed lower diversity and higher clonality than mem-CD4+ cells. We identified 22,947 CD4+ and 12,126 CD8+ different clonotypes. Brain-infiltrating clonotypes that are also present in mem-CD4+ or mem-CD8+ T cell pools and correlations of their frequencies are shown in Figure 1C. Since the numbers of shared clonotypes between brain lesions and the peripheral pools are also influenced by the cloneset sizes of the samples (Figure S1A in Supplementary Material), these numbers have been normalized (Figure 1C). Normalized values were very similar for the three lesions and slightly higher for the mem-CD8+ pool (Figure 1C). As expected, the number of shared clonotypes between these T cell pools and the control brain biopsies was very low (Figure S1C in Supplementary Material). Low-active-LI and medium-active-LIV showed higher correlation with mem-CD8+ than with mem-CD4+ cells and higher correlation than high-active-LIII for both T cell pools (Figure 1C). Similar CD4/CD8 ratios were observed in the three lesions (Figure 1D). From the 50 clonotypes common to the three lesions, 40% were CD4+, 50% CD8+, and 10% undetermined, i.e., not identified in the peripheral pools (Table 2). These shared clonotypes were among the most frequent clonotypes in the mem-CD8+ but not in the mem-CD4+ pool (Figure 1C large symbols). While only 7 of the 20 CD4+ clonotypes common to the three lesions were among the 10% most frequent clonotypes in mem-CD4+, representing only 0.37% of all cells; 22 of the 25 CD8+ clonotypes common to the three lesions were among the 10% most frequent clonotypes in mem-CD8+, representing 32.3% of all cells (filled cells Table 2). Analysis of the 10 most frequent clonotypes identified in each lesion also showed a higher overlap with mem-CD8+ than with mem-CD4+ cells (Table S2 in Supplementary Material). Accordingly, the frequency of the 10 most frequent clonotypes in the mem-CD8+ T cell pool that were also identified in lesions was higher than in the mem-CD4+ T cell pool (Table S2 in Supplementary Material).

### Comparison of the CD4+ and CD8+ Global TRBV Repertoires in Demyelinating Brain Lesions

**Figure 2:**
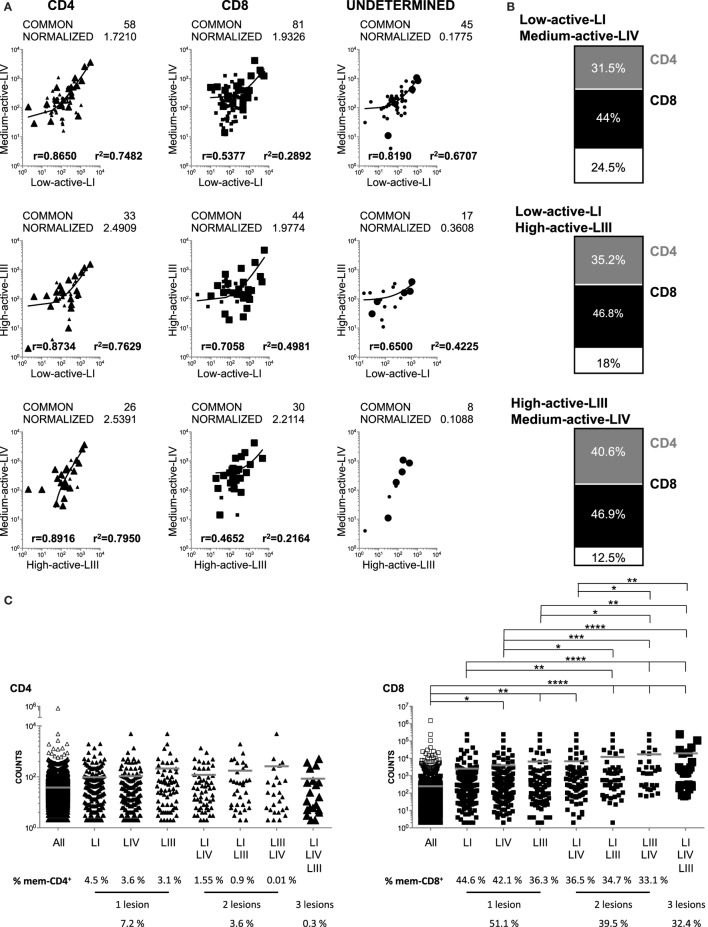
Comparison of the global CD4+ and CD8+ TRBV repertoires in brain lesions and their presence in peripheral CD4+ and CD8+-ory T cell pools. **(A)** Logarithmic scatter plots comparing the number of copies of each CD4+ (left scatters), CD8+ (middle scatters), or undetermined (right scatters) clonotypes identified by gDNA sequencing and shared by each pair of brain lesions. CD4+ clonotypes are represented by triangles, CD8+ by squares and undetermined clonotypes by circles. Clonotypes not shared by lesions are shown in gray and shared ones in black. Large symbols represent clonotypes shared by the three lesions. Actual and normalized numbers of shared clonotypes as well as correlation coefficient (r) and coefficient of determination (*r*^2^) of their frequencies are shown in all scatters. **(B)** Charts representing the frequencies of CD4+ (in gray), CD8+ (in black), and undetermined, i.e., not identified in the peripheral pools, (in white) clonotypes in the global T cell repertoires and shared by each pair of brain lesions. **(C)** Dot plots comparing the numbers of copies in CD4+ (triangles, left graph) and CD8+ (squares, right graph) peripheral memory T cell pools of: all mem-CD4+ and mem-CD8+ peripheral clonotypes (all, white) and peripheral clonotypes also present in one, two or the three brain lesions (black). Large symbols represent clonotypes shared by the three lesions. Mean (gray line) and statistical significance (*p < 0.05, **p < 0.01, ***p < 0.001, and ****p < 0.0001) are shown. Frequencies of mem-CD4+ and mem-CD8+ clonotypes present in one, two, or three lesions are also shown.

Next, we analyzed the CD4+ and CD8+ clonotypes shared by the different lesions (Figure 2A). The three comparisons showed similar normalized numbers of shared CD8+ clonotypes, while the low-active-LI/medium-active-LIV lesions shared less CD4+ clonotypes (Figure 2A). In the three comparisons, the frequencies of shared CD4+ clonotypes showed higher correlation than those of shared CD8+ clonotypes (Figure 2A). The fraction of CD4+ and CD8+ clonotypes shared by the different lesions was very similar (Figure 2B), with only slightly higher frequency of shared CD4+ clonotypes for high-active-LIII/medium-active-LIV. Clonotypes shared by these two lesions also showed the lowest frequency of clonotypes that were not identified in the peripheral pools (12.5%), similar to the frequency observed in the clonotypes shared by the three lesions (10%, Table 2).

The counts in the peripheral memory T cell pools of brain-infiltrating clonotypes present in one, two, or three lesions are shown in Figure 2C. CD8+ clonotypes, which were present in the high-active-LIII and medium-active-LIV, were significantly more abundant in the corresponding peripheral memory T cell pool, and the significance was even higher for CD8+ clonotypes shared by two and three lesions (Figure 2C). This was not the case for CD4+ clonotypes. Furthermore, 51% of peripheral mem-CD8+ cells were present in one lesion, 39% in two and 32% in the three lesions, while only 7% of mem-CD4+ cells were present in one brain lesion, 3% in two and 0.38% in the three brain lesions (Figure 2C). The percentages of mem-CD8+ and mem-CD4+ cells present in the brain lesions showed a slightly negative correlation with the inflammatory activity of the lesion, and accordingly, the lowest percentages were found for high-active-LIII (Figure 2C).

### Comparison of the Functional TRBV Repertoires in Demyelinating Brain Lesions

**Figure 3:**
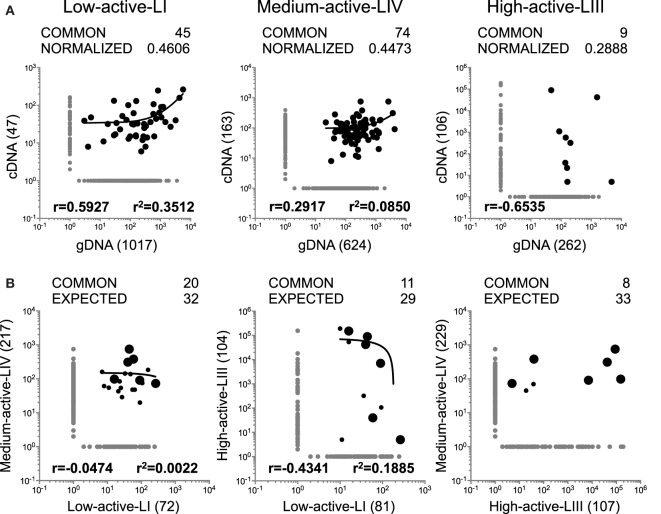
Analysis of TRBV repertoires in brain lesions by cDNA TRBV sequencing. **(A,B)** Logarithmic scatter plots comparing the number of copies of each clonotype identified by gDNA and cDNA sequencing in each brain lesion **(A)** and the number of copies of each clonotype (cDNA sequencing) infiltrating each pair of brain lesions **(B)**. Clonotypes not shared by brain lesions are shown in gray and clonotypes shared by brain lesions in black. Large symbols represent the six clonotypes shared by the three lesions. Actual, normalized and expected numbers of shared clonotypes as well as correlation coefficient (*r*) and coefficient of determination (*r*^2^) of their frequencies are shown in all scatters. Number of clonotypes not shared by the different samples are also shown in parenthesis.

As mentioned above, while gDNA TRBV-seq should reflect the “global” TRBV repertoires very well, cDNA TRBV-seq might provide additional information about the functional status of infiltrating T cells. Considering that the lesions analyzed in this study have different levels of inflammatory activity and might contain different frequencies of active T cells, we analyzed the TRBV repertoires in the three lesions also by cDNA TRBV-seq. Sequence information is summarized in Table 1 and the complete list of all sequences is available in Ref. (22) and Table S1 in Supplementary Material. A search using BLASTP program of the non-redundant protein database indicated that none of these TRBV sequences matched any sequence in the database. Unexpectedly, the number of sequences obtained by cDNA sequencing was lower in the three lesions than those obtained by gDNA sequencing. Although we only analyzed samples with RIN values around 6, the partial RNA degradation, which cannot completely be avoided when working with human autopsy brain tissue, was most likely responsible for these differences. However, the differences associated with the inflammatory activity of the lesions and in particular the lowest diversity and highest clonality in high-active-LIII support the relevance of the cDNA TRBV-seq data. Comparisons of TRBV repertoires obtained by gDNA and cDNA sequencing in the three lesions are shown in Figure 3A. Normalized values have been used for these comparisons since the numbers of shared clonotypes correlated with the cloneset sizes of the samples (Figure S2A in Supplementary Material). As foreseeable, the highest normalized number of shared clonotypes using both approaches as well as the highest correlation of their frequencies were found in the low-active-LI, in which a higher frequency of inactive TCCs with similar numbers of TRBV gDNA and cDNA copies per cell is expected. Accordingly, the lowest number of shared clonotypes and correlation of their frequencies were found in the high-active-LIII, in which a higher frequency of active TCCs having one TRBV gDNA and several cDNA copies per cell is expected.

Clonotypes, which were identified by cDNA TRBV-seq and which are shared among the different pairs of lesions as well as the correlations of their frequencies are represented in Figure 3B. For the first time, no correlation was observed between the numbers of shared clonotypes identified by cDNA sequencing and the cloneset sizes of the samples (Figure S2B in Supplementary Material), and therefore, we did not normalize for cloneset sizes. Using the regression line of the correlation between the numbers of shared clonotypes in each pair of samples and the product of the intersected cloneset sizes obtained by gDNA sequencing (Figure S2C in Supplementary Material), we calculated the expected number of clonotypes shared between lesions with the assumption that TCCs were inactive (one cDNA copy per cell) (Figure 3B). The number of common clonotypes, which we identified by cDNA sequencing, was lower than the expected one for the three comparisons. However, clonotypes shared by high-active-LIII/medium-active-LIV, the two lesions most likely containing higher numbers of active T cells, showed the highest discrepancy between the numbers of expected and observed common clonotypes. Regarding the frequencies of clonotypes shared by the different lesions, no correlations were found.

Six clonotypes identified by cDNA sequencing were common to the three lesions. Four of these six clonotypes were among the most frequent clonotypes in high-active-LIII, three in medium-active-LIV, and only one in low-active-LI (large circles in Figure 3B; Table 2). Three of these six clonotypes were also identified in the three lesions by gDNA sequencing (Table 2). The presence in the other lesions of the 10 most frequent clonotypes identified in each lesion by cDNA-seq is shown in Table S2 in Supplementary Material. Interestingly, the 10 most frequent clonotypes identified in low-active-LI by c-DNA-seq that were also identified by gDNA in brain lesions were all CD8+ (Table S2 in Supplementary Material).

### Only Brain-Infiltrating TCCs Identified by cDNA Sequencing in Active Lesions Can Be *In Vitro* Expanded From the CSF

Using monoclonal antibodies specific for the TRBV families expressed by the 10 most frequent clonotypes identified by gDNA and cDNA sequencing in the three lesions, we sorted previously phytohemagglutinin-expanded autologous CSF-infiltrating T cells, cloned them by limiting dilution and sequenced the CDR3 region as previously described (22). Using this approach we have generated 34 CSF-TCCs (Table 3). Two of these CSF-TCCs were identified also in the mem-CD4 + pool and four in the mem-CD8+. One of these CSF-derived TCCs, a CD8 + CSF-TCC, was among the clonotypes identified in low-active-LI by gDNA sequencing, while 13 CSF-TCCs, 8 CD8+, and 5 CD4+, were identified in high-active-LIII by cDNA sequencing. Ten of these brain-infiltrating CSF-TCCs have been functionally characterized in a previous study (22).

**Table 3:** CSF-derived TCCs.

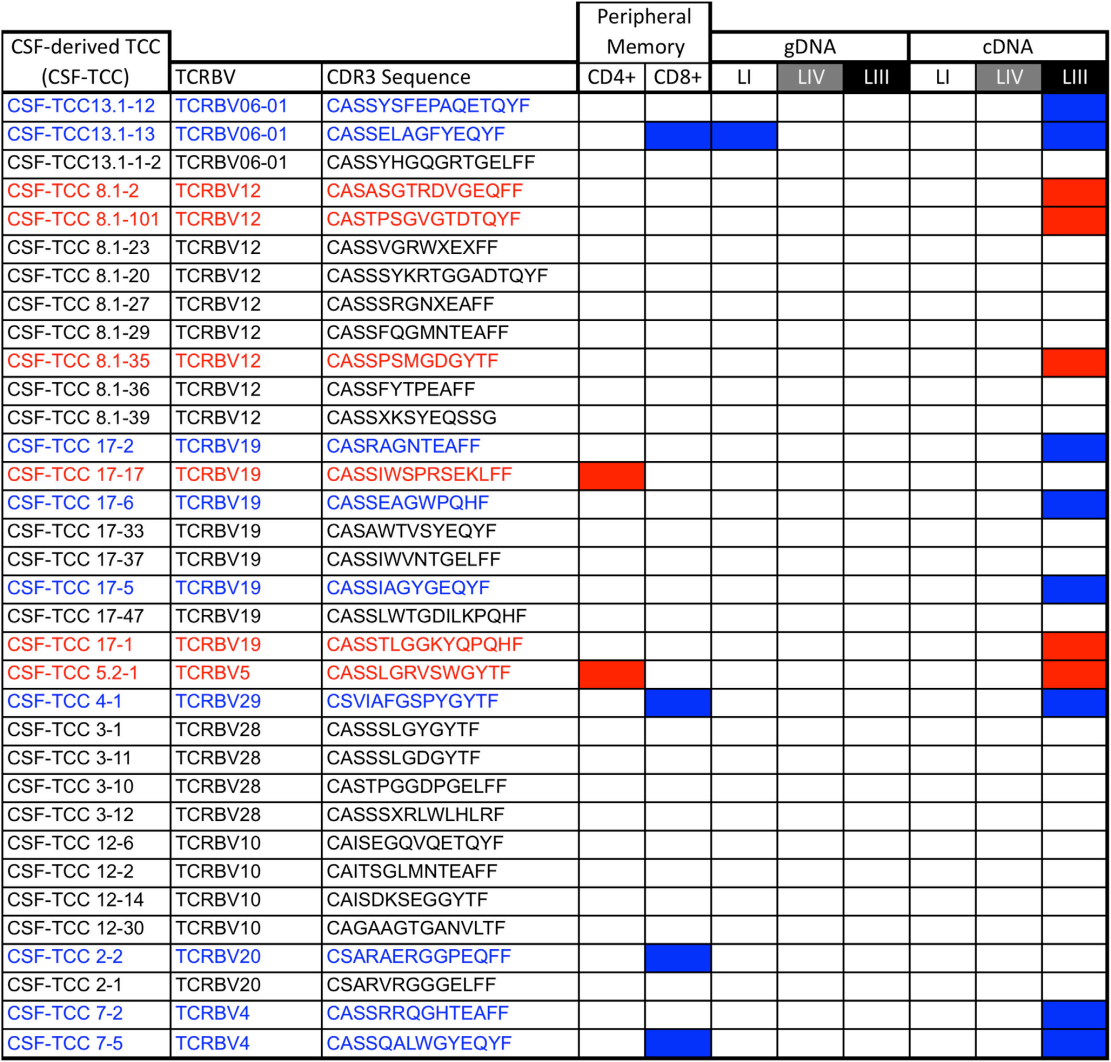

*CD4+ TCCs are depicted in red and CD8+ TCCs in blue*.

## Discussion

Clonal T cell expansions in MS brain lesions allow the identification of T cells that are assumed to be involved in the disease process. The TCR sequence functions as fingerprint for each TCC, and TRBV-seq serves as a powerful tool for T cell analysis and has already provided important new knowledge about TRBV repertoires in MS (19–23). In the present study, we have used TRBV-seq of MS brain lesions with different location and inflammatory activity and paired circulating memory CD4+ and CD8+ T cells to identify putatively pathogenic T cells and to address a number of important questions such as how lesion location or inflammatory activity influence brain-infiltrating TRBV repertoires, whether brain infiltrates are primarily CD4+ or CD8+ T cell-derived, and how brain-infiltrating T cells are represented in the peripheral memory T cell compartments. Furthermore, we explored whether parallel TRBV-seq of gDNA and cDNA improves characterization of TRBV repertoires in autopsy brain tissue. It is important to note that the three demyelinating brain lesions analyzed in this study were from a single SPMS patient with pattern II demyelinating lesions (22), and therefore, one may not be able to extrapolate our results to patients with other clinical forms of MS or other demyelinating patterns. In addition, brain tissue was obtained after the patient had been prepared for autologous hematopoietic stem cell transplantation with cyclophosphamide and G-CSF, treatments that may affect the T cell repertoire (35). Pattern II demyelination is associated with macrophages and T cell inflammation, as well as prominent deposition of antibody/complement complexes at sites of active myelin destruction (1). Pattern II has been the dominant pattern in histopathological studies, although it might be overrepresented in biopsy cohorts (36). The different location and level of inflammatory activity of the three demyelinating lesions allowed us to explore the putative contribution of these two factors to T cell repertoires.

The global TRBV repertoires analyzed by gDNA TRBV-seq showed similar diversity and clonality in the three MS lesions as well as a noteworthy overlap, which was not influenced by lesion location or activity level, and hence suggests an important sharing of clonally expanded clonotypes across MS lesions. Since different autologous brain lesions were available only from one MS patient and no non-MS control, we cannot discern whether the level of overlap is a general feature of tissue-resident, brain-infiltrating memory T cells, which could also be found in healthy donors, whether it is a common feature in brain inflammation, or is a characteristic of MS lesions. In a previous study, TCR analysis of two brain regions from a RE patient demonstrated that 7 of the top 10 CNS-expanded clones matched between the two regions (37), suggesting that the sharing of clonotypes across brain regions is not a specific feature of MS infiltrates. Regarding the pathological relevance of the shared T cell clonotypes, we assume that these clonotypes contain both public clones without association with MS, but also some that are most likely disease-relevant. The relatively low overlap between these clonotypes and peripheral T cells in HD as well as the fact that these clonotypes were not among the 10,000 reported public TCR sequences support our assumption, although further comparison with a MHC-matched HD cohort is required. In order to explore these putatively relevant shared- and clonally expanded clonotypes in more detail and to analyze also whether the overall brain infiltrates are primarily CD4+ or CD8+ T cell-derived, we compared these repertoires with those of autologous peripheral blood mem-CD8+ and mem-CD4+ T cell pools. In agreement with previous studies (7, 8), we found a greater abundance of CD8+ clonotypes in the overall TRBV repertoires in the three lesions as well as in the clonally expanded and shared clonotypes. However, despite the overall low abundance of CD4+ clonotypes, several of the most clonally expanded clonotypes shared by the three lesions were CD4+. The CD4/CD8 ratios were comparable in the three lesions. These CD4/CD8 ratios were higher than those found by immunohistochemistry in the same patient (22) or reported in other studies (7, 8, 11, 16, 17). The higher clonal expansions in the mem-CD8+ compared with the mem-CD4+ T cell pool result in a lower number of unique TRBV sequences for the same number of cells analyzed. A lower number of unique sequences to compare translates into a lower overlap and as a result higher frequency of undetermined CD8+ than CD4+ clonotypes, i.e., TRBV sequences that were not found in the peripheral mem-CD4+ and mem-CD8+ pools and hence could not be assigned to either CD4+ or CD8+ T cells. This probably explains the difference in the CD4/CD8 ratios. We found a higher overlap between the global TRBV repertoires of the three lesions and the TRBV repertoire of mem-CD8+ when compared with mem-CD4+ T cell pool. Although the normalized numbers of common clonotypes between lesions and the two peripheral pools were comparable in the three lesions, the high-active-LIII showed a lower correlation of its frequencies indicating a repertoire that is more different from the peripheral one. The most frequent CD8+ clonotypes identified in the lesions, and particularly those shared by different lesions, also showed significantly higher frequencies in the peripheral blood. This finding suggests that clonally expanded CD8+ clonotypes in brain lesions, that might include some MAIT CD8+ T cells, most likely derive from TCCs that are clonally expanded outside the CNS. Several studies have reported a skewed TCR repertoire in the peripheral blood of MS patients (9, 13), and this observation is more pronounced in the CD8+ compartment (15). However, since clonal CD8+ T cell expansions are also common in healthy individuals and have been associated with age, lymphopenic conditions, and infection with cytomegalovirus (38–43), it is difficult to estimate the putative involvement of these cells in MS pathogenesis. Furthermore, after autologous stem cell transplantation, one of the most efficacious therapies in MS by resetting the immune system, the reestablished CD8+ T cell repertoire contains clones already present before treatment, while the CD4+ T cell repertoire was reset by newly generated clones (19). Regarding CD4+ T cells, the most frequent clonotypes identified in the global TRBV repertoires of brain lesions were not the most frequent in the mem-CD4+ peripheral T cell pool suggesting clonal expansions inside the brain or specific recruitment of CD4+ TCCs into this organ. This observation supports a putative role of these cells in MS pathogenesis, and consistent with this, a very high correlation coefficient of the frequencies of the CD4+ TCCs shared by the different brain lesions was observed.

TRBV repertoires in the three brain lesions analyzed by cDNA TRBV-seq showed differences in diversity and clonality associated with the activity level of the lesion that were not found by gDNA TRBV-seq. The high-active-LIII, in which we expect a higher frequency of active pathogenic T cells, accordingly showed the lowest diversity and highest clonality. In addition, this lesion also showed the lowest overlap between the TRBV repertoires identified by gDNA and cDNA TRBV-seq. The overlap between TRBV repertoires identified by gDNA and cDNA-seq was lower than expected in the three lesions. Assuming that cDNA-seq most likely shows the overall TRBV repertoire and cDNA the active TRBV repertoire, we expect differences regarding the frequency of the clonotypes but not their identity. Technical artifacts such as preferential degradation of RNA from some cells or a problem during retrotranscription and sequencing, when mRNA for some clonotypes is much more abundant than for others might be behind this low overlap. Although we know that, due to partial degradation of RNA in our autopsy brain tissue, cDNA TRBV-seq only provides limited information, these differences in the TRBV repertoires associated with the inflammatory activity of the lesion support the value of our analysis. cDNA TRBV repertoires showed lower overlap between the three lesions and particularly between the most active lesions, which indicates more distinct active and putatively pathogenic TRBV repertoires. The *in vitro* expansion from the CSF of only those TCCs that are brain-infiltrating and clonally expanded according to cDNA TRBV-seq in high-active-LIII and the ability of several of these CSF-TCCs to release Th2 cytokines and help B cells in pattern II demyelination (22), strongly support their pathogenic role and also underline the value of cDNA TRBV-seq of active lesions to identify pathogenic cells. The difficulty to expand *in vitro* CSF-TCCs that infiltrated other lesions with lower inflammatory activity most likely is due to exhaustion and not to the fact that they do not recirculate through the CSF, since a previous study demonstrated that the TRBV repertoire in the CSF analyzed by cDNA TRBV-seq almost completely mirrored that in brain lesions (23).

In conclusion, MS brain lesions, at least in a SPMS patient with pattern II demyelination, independently of their proximity or inflammatory activity, contain CD4+ and CD8+ clonally expanded clonotypes that may play a role in disease pathogenesis. Of particular interest are brain-infiltrating CD4+ clonotypes, since they are infrequent in peripheral blood and, therefore, might be clonally expanded inside the brain or specifically recruited to this tissue. Including cDNA TRBV-seq in the characterization of TRBV repertoires of brain lesions may facilitate the identification of putatively pathogenic T cells in lesions with high inflammatory activity.

## Ethics Statement

The study of MS clinical cases 1 and 2 was approved by the Ethik Kommission der ärztekammer Hamburg, protocol No. 2758, and informed consent was obtained from the patient or relatives. The study of the RE case was approved by the Cantonal Ethics Committee Zurich (No. 33-2015) and informed consent was obtained accordingly from the parents.

## Author Contributions

RP, sample preparation, generation of TCCs, and critical reading of the manuscript; IM, immunohistopathology, analysis of results, and critical reading of the manuscript; RM, outline of scientific questions, design and supervision of experiments, and editing manuscript; MS, outline of scientific questions, design and supervision of all experimental aspects of the study, analysis of results, and writing and editing manuscript.

## Conflict of Interest Statement

The authors declare that the research was conducted in the absence of any commercial or financial relationships that could be construed as a potential conflict of interest.

## Footnotes

^1^www.IMGT.org (accessed July 22, 2004).

^2^http://blast.ncbi.nlm.nih.gov (accessed April 23, 2017).
