# Supplementary material for: Detailed Characterization of T Cell Receptor Repertoires in Multiple Sclerosis Brain Lesions
Source: Front Immunol. 2018 Mar 19;9:509. doi: 10.3389/fimmu.2018.00509 (PMC5867461; doi:10.3389/fimmu.2018.00509)
Supplement: Supplementary file 1 [file Presentation_1.PDF]

Table SI. Complete list of TCR sequences

Genomic DNA

LESION I

AminoAcid	Count (reads)	Frequency Count (%)	CDR3Length	*VMaxResolved*	*DMaxResolved*	*JMaxResolved*
CASNPGTAYSYEQYF	5646	4.017275852	45	*TCRBV06-01*01*	*TCRBD01-01*01*	*TCRBJ02-07*01*
CASSIQEWSTEAFF	4272	3.039639114	42	*TCRBV19-01*	*unresolved*	*TCRBJ01-01*01*
CASSSGTNAYGYTF	3528	2.510263763	42	*TCRBV06-01*01*	*TCRBD01-01*01*	*TCRBJ01-02*01*
CASSLSGTGVYEQYF	3184	2.265498815	45	*TCRBV12*	*TCRBD01-01*01*	*TCRBJ02-07*01*
CASGDYEQYF	2170	1.544011441	30	*TCRBV28-01*01*	*unresolved*	*TCRBJ02-07*01*
CASSYNRGDRGGYEQYF	1876	1.334822794	51	*TCRBV18-01*01*	*TCRBD01-01*01*	*TCRBJ02-07*01*
CASNHAWVSNQPQHF	1587	1.129191778	45	*TCRBV23-01*01*	*unresolved*	*TCRBJ01-05*01*
CSAGTIDLNTEAFF	1462	1.040251026	42	*TCRBV29-01*01*	*unresolved*	*TCRBJ01-01*01*
CASSLFDRGGNEKLFF	1227	0.873042414	48	*TCRBV28-01*01*	*TCRBD02-01*02*	*TCRBJ01-04*01*
CASSFPSTDTQYF	1136	0.808293547	39	*TCRBV05-06*01*	*TCRBD02-01*02*	*TCRBJ02-03*01*
CASSQSAATGNYEQYF	1076	0.765601987	48	*TCRBV07-06*01*	*TCRBD01-01*01*	*TCRBJ02-07*01*
CASSLYRGGTQYF	1052	0.748525362	39	*TCRBV05-06*01*	*TCRBD01-01*01*	*TCRBJ02-03*01*
CASSRLLNTIYF	995	0.70796838	36	*TCRBV03*	*TCRBD02-01*	*TCRBJ01-03*01*
CASTTEGTQETQYF	978	0.695872438	42	*TCRBV06-06*	*unresolved*	*TCRBJ02-05*01*
CASSPQDRGLRDGYTF	920	0.654603929	48	*TCRBV27-01*01*	*TCRBD01-01*01*	*TCRBJ01-02*01*
CASSQGTGGIGNSPLHF	834	0.593412692	51	*TCRBV06*	*TCRBD01-01*01*	*TCRBJ01-06*01*
CAWSVPAGNGANVLTF	833	0.592701166	48	*TCRBV30-01*01*	*TCRBD01-01*01*	*TCRBJ02-06*01*
CASSQDGEAIGGKNIQYF	754	0.536490611	54	*TCRBV04-01*01*	*TCRBD02-01*02*	*TCRBJ02-04*01*
CASSQAKNTEAFF	741	0.527240773	39	*TCRBV04-01*01*	*unresolved*	*TCRBJ01-01*01*
CASSPPGRGSYEQYF	734	0.522260091	45	*TCRBV06-06*	*TCRBD01-01*01*	*TCRBJ02-07*01*
CSVFQDRGSSGELFF	718	0.510875675	45	*TCRBV29-01*01*	*TCRBD01-01*01*	*TCRBJ02-02*01*
CASSLNTGELFF	706	0.502337363	36	*TCRBV05-01*01*	*unresolved*	*TCRBJ02-02*01*
CASSPIGESWGEQYF	699	0.497356681	45	*TCRBV04-02*01*	*unresolved*	*TCRBJ02-07*01*
CASSFTGRAYQPQHF	694	0.493799051	45	*TCRBV28-01*01*	*unresolved*	*TCRBJ01-05*01*
CASSFGTLYSNQPQHF	641	0.456088172	48	*TCRBV06-05*01*	*unresolved*	*TCRBJ01-05*01*
CASSVGPGSSYEQYF	622	0.442569178	45	*TCRBV09-01*	*TCRBD01-01*01*	*TCRBJ02-07*01*

CASSLFTGDEAFF	591	0.420511872	39	*TCRBV27-01*01*	*TCRBD01-01*01*	*TCRBJ01-01*01*
CASSIAQGATEAFF	582	0.414108138	42	*TCRBV19-01*	*TCRBD01-01*01*	*TCRBJ01-01*01*
CASSQVQAIDTQYF	580	0.412685086	42	*TCRBV04-01*01*	*TCRBD02-01*	*TCRBJ02-03*01*
CASIQDNVEGITGELFF	573	0.407704404	51	*TCRBV12*	*TCRBD02-01*02*	*TCRBJ02-02*01*
CASSHGTGNQPQHF	554	0.394185409	42	*TCRBV06-05*01*	*TCRBD01-01*01*	*TCRBJ01-05*01*
CASSYDSAGGYTF	543	0.386358623	39	*TCRBV05-05*01*	*TCRBD02-01*	*TCRBJ01-02*01*
CASSFLRGPKRYNEQFF	520	0.369993525	51	*TCRBV12*	*unresolved*	*TCRBJ02-01*01*
CASSFGQAASPLHF	514	0.365724369	42	*TCRBV12*	*unresolved*	*TCRBJ01-06*01*
CASVNGQADTEAFF	502	0.357186057	42	*TCRBV28-01*01*	*TCRBD01-01*01*	*TCRBJ01-01*01*
CASSYRSSGANVLTF	492	0.350070797	45	*TCRBV07-03*01*	*unresolved*	*TCRBJ02-06*01*
CASSPALHEQYF	481	0.342244011	36	*TCRBV12*	*TCRBD02-01*	*TCRBJ02-07*01*
CASSYGGQVDEAFF	473	0.336551803	42	*TCRBV06-05*01*	*TCRBD01-01*01*	*TCRBJ01-01*01*
CASSHGQGATRNGYTF	469	0.333705699	48	*TCRBV12*	*TCRBD01-01*01*	*TCRBJ01-02*01*
CSARESSHSGANVLTF	469	0.333705699	48	*TCRBV20*	*TCRBD02-01*	*TCRBJ02-06*01*
CASSHRTSGGLVGNIQYF	468	0.332994173	54	*TCRBV03*	*TCRBD02-01*01*	*TCRBJ02-04*01*
CASSLRKRGGLDTQYF	466	0.331571121	48	*TCRBV28-01*01*	*TCRBD01-01*01*	*TCRBJ02-03*01*
CASQVEGINYGYTF	441	0.31378297	42	*TCRBV10-01*	*TCRBD02-01*02*	*TCRBJ01-02*01*
CASSASIAGGNNEQFF	434	0.308802288	48	*TCRBV06-01*01*	*TCRBD02-01*01*	*TCRBJ02-01*01*
CASSLAYRDESEQYF	424	0.301687028	45	*TCRBV05-06*01*	*TCRBD02-01*02*	*TCRBJ02-07*01*
CASSLWRGATNEKLFF	420	0.298840924	48	*TCRBV28-01*01*	*unresolved*	*TCRBJ01-04*01*
CARSLWHSYEQYF	414	0.294571768	39	*TCRBV05-03*	*TCRBD01-01*01*	*TCRBJ02-07*01*
CASSPPGQGVKLFF	412	0.293148716	42	*TCRBV06-06*	*TCRBD01-01*01*	*TCRBJ01-04*01*
CASSPTGREAFF	402	0.286033456	36	*TCRBV05-01*01*	*unresolved*	*TCRBJ01-01*01*
CATRGEDNEQFF	398	0.283187352	36	*TCRBV12*	*TCRBD02-01*01*	*TCRBJ02-01*01*
**CASSELAGFYEQYF***	396	0.2817643	42	*TCRBV06-01*01*	*TCRBD02-01*01*	*TCRBJ02-07*01*
CASSEGANTEAFF	393	0.279629722	39	*TCRBV06-01*01*	*TCRBD02-01*02*	*TCRBJ01-01*01*
CASSPISGGVSGANVLTF	392	0.278918196	54	*TCRBV12*	*TCRBD02-01*	*TCRBJ02-06*01*
CASKKRDRVREAFF	378	0.268956832	42	*TCRBV12*	*TCRBD01-01*01*	*TCRBJ01-01*01*
CATGRRGSTYNEQFF	372	0.264687676	45	*TCRBV05-06*01*	*unresolved*	*TCRBJ02-01*01*
CASRPPSDRWDYGYTF	365	0.259706994	48	*TCRBV28-01*01*	*unresolved*	*TCRBJ01-02*01*
CASSLAGQTTNEKLFF	363	0.258283942	48	*TCRBV05-01*01*	*TCRBD01-01*01*	*TCRBJ01-04*01*
CASSQAPGAYNEQFF	362	0.257572416	45	*TCRBV04-02*01*	*TCRBD02-01*01*	*TCRBJ02-01*01*

CASSYRQPFYNEQFF	362	0.257572416	45	*TCRBV06-05*01*	*TCRBD01-01*01*	*TCRBJ02-01*01*
CASSWGGGGFYGYTF	355	0.252591733	45	*TCRBV05-06*01*	*TCRBD01-01*01*	*TCRBJ01-02*01*
CASRRGAGPQETQCF	351	0.249745629	45	*TCRBV06-05*01*	*TCRBD02-01*01*	*TCRBJ02*
CASSDWGEEGTEAFF	342	0.243341895	45	*TCRBV12*	*TCRBD02-01*02*	*TCRBJ01-01*01*
CSASLISGADTQYF	341	0.242630369	42	*TCRBV20*	*TCRBD02-01*	*TCRBJ02-03*01*
CASSYDRGDEQFF	335	0.238361213	39	*TCRBV06-06*	*TCRBD01-01*01*	*TCRBJ02-01*01*
CASSIGTYNEQFF	332	0.236226635	39	*TCRBV19-01*	*unresolved*	*TCRBJ02-01*01*
CASSLALTDTQYF	329	0.234092057	39	*TCRBV07-08*01*	*unresolved*	*TCRBJ02-03*01*
CASSIVGRAGEKLFF	325	0.231245953	45	*TCRBV19-01*	*unresolved*	*TCRBJ01-04*01*
CASSLMAGRSTDTQYF	324	0.230534427	48	*TCRBV12*	*TCRBD02-01*01*	*TCRBJ02-03*01*
CASTVSNSPLHF	321	0.228399849	36	*TCRBV13-01*01*	*TCRBD02-01*	*TCRBJ01-06*01*
CASSNPTSGSLYEQYF	318	0.226265271	48	*TCRBV21-01*01*	*TCRBD02-01*02*	*TCRBJ02-07*01*
CASKFSYAYEQYF	316	0.224842219	39	*TCRBV02-01*01*	*TCRBD02-01*	*TCRBJ02-07*01*
CASSIRQIGNQPQHF	314	0.223419167	45	*TCRBV19-01*	*TCRBD01-01*01*	*TCRBJ01-05*01*
CASSPNRFNEQYF	312	0.221996115	39	*TCRBV06-01*01*	*unresolved*	*TCRBJ02-07*01*
CASSLVAGTGEAFF	309	0.219861537	42	*TCRBV05-05*01*	*TCRBD01-01*01*	*TCRBJ01-01*01*
CASSQDWPGGSYEQYF	306	0.217726959	48	*TCRBV04-01*01*	*TCRBD02-01*01*	*TCRBJ02-07*01*
CSFRQGAGNEQYF	306	0.217726959	39	*TCRBV29-01*01*	*TCRBD01-01*01*	*TCRBJ02-07*01*
CASSPRRTEKAFF	304	0.216303907	39	*TCRBV06-05*01*	*TCRBD01-01*01*	*TCRBJ01-01*01*
CASSQDLVTEAFF	304	0.216303907	39	*TCRBV14-01*01*	*TCRBD02-01*	*TCRBJ01-01*01*
CSAREPQGGGYTF	298	0.212034751	39	*TCRBV20*	*TCRBD01-01*01*	*TCRBJ01-02*01*
CASTLVAGNTIYF	297	0.211323225	39	*TCRBV12*	*unresolved*	*TCRBJ01-03*01*
CASSFWAEEEANEKLFF	294	0.209188647	51	*TCRBV05-06*01*	*TCRBD02-01*02*	*TCRBJ01-04*01*
CASSHRNSGGDNEQFF	292	0.207765595	48	*TCRBV07-06*01*	*TCRBD02-01*01*	*TCRBJ02-01*01*
CASSQDLYGYTF	291	0.207054069	36	*TCRBV19-01*	*unresolved*	*TCRBJ01-02*01*
CASSPSGGAPLNTEAFF	289	0.205631017	51	*TCRBV07-06*01*	*TCRBD02-01*	*TCRBJ01-01*01*
CASRANSGNTIYF	287	0.204207965	39	*TCRBV05-01*01*	*unresolved*	*TCRBJ01-03*01*
CASSFGTGTPESMRNYGYTF	287	0.204207965	60	*TCRBV05-01*01*	*TCRBD01-01*01*	*TCRBJ01-02*01*
CASSFAGNQPQHF	283	0.201361861	39	*TCRBV06-05*01*	*unresolved*	*TCRBJ01-05*01*
CASSERSGANVLTF	282	0.200650335	42	*TCRBV06-04*	*TCRBD02-01*	*TCRBJ02-06*01*
CASSLEGRGPFYEQYF	280	0.199227283	48	*TCRBV05-06*01*	*TCRBD01-01*01*	*TCRBJ02-07*01*
CASSPAGRVNTEAFF	280	0.199227283	45	*TCRBV05-04*01*	*TCRBD02-01*	*TCRBJ01-01*01*

CASSLFGNTEAFF	279	0.198515757	39	*TCRBV28-01*01*	*TCRBD02-01*	*TCRBJ01-01*01*
CASSTRGPSYEQYF	279	0.198515757	42	*TCRBV19-01*	*TCRBD02-01*01*	*TCRBJ02-07*01*
CASTWGDGSPLHF	279	0.198515757	39	*TCRBV10-02*01*	*TCRBD02-01*01*	*TCRBJ01-06*01*
CASSLDHFYGYTF	277	0.197092705	39	*TCRBV07-09*	*unresolved*	*TCRBJ01-02*01*
CASSLVSQWETQYF	276	0.196381179	42	*TCRBV07-09*	*TCRBD01-01*01*	*TCRBJ02-05*01*
CAIRAGTGAFF	275	0.195669653	33	*TCRBV10-03*01*	*unresolved*	*TCRBJ01-01*01*
CASSSRTSGRAIREQYF	275	0.195669653	51	*TCRBV06*	*TCRBD02-01*02*	*TCRBJ02-07*01*
CASSNSIAGGTNQPQHF	274	0.194958127	51	*TCRBV06*	*TCRBD01-01*01*	*TCRBJ01-05*01*
CASSVLEGTDTQYF	272	0.193535075	42	*TCRBV06-06*	*TCRBD02-01*02*	*TCRBJ02-03*01*
CASNTGTTNEQFF	266	0.189265919	39	*TCRBV19-01*	*TCRBD02-01*	*TCRBJ02-01*01*
CASSFSGNQPQHF	265	0.188554393	39	*TCRBV07-02*01*	*unresolved*	*TCRBJ01-05*01*
CASSAQGGIGTIYEQYF	264	0.187842867	51	*TCRBV07-08*01*	*TCRBD01-01*01*	*TCRBJ02-07*01*
CSVAGRVSNEQFF	264	0.187842867	39	*TCRBV29-01*01*	*TCRBD01-01*01*	*TCRBJ02-01*01*
CASSTGNYGYTF	263	0.187131341	36	*TCRBV19-01*	*TCRBD02-01*01*	*TCRBJ01-02*01*
CASSQAKANQPQHF	262	0.186419815	42	*TCRBV03*	*unresolved*	*TCRBJ01-05*01*
CASRQGAYTF	261	0.185708289	30	*TCRBV06-01*01*	*TCRBD01-01*01*	*TCRBJ01-02*01*
CASSLRVGALPTNEKLFF	261	0.185708289	54	*TCRBV05-04*01*	*TCRBD01-01*01*	*TCRBJ01-04*01*
CASEGTGTTNEKLFF	259	0.184285237	45	*TCRBV02-01*01*	*unresolved*	*TCRBJ01-04*01*
CASTQVGTAYEQYF	258	0.183573711	42	*TCRBV06*	*TCRBD01-01*01*	*TCRBJ02-07*01*
CASSPRPEGPEQYF	257	0.182862185	42	*TCRBV14-01*01*	*unresolved*	*TCRBJ02-07*01*
CASSKEGGMNTEAFF	255	0.181439133	45	*TCRBV21-01*01*	*TCRBD01-01*01*	*TCRBJ01-01*01*
CASSPGPNQPQHF	254	0.180727606	39	*TCRBV05-01*01*	*TCRBD01-01*01*	*TCRBJ01-05*01*
CASSSTATMNTEAFF	253	0.18001608	45	*TCRBV06-06*	*unresolved*	*TCRBJ01-01*01*
CASRPTRTQTYSNQPQHF	251	0.178593028	54	*TCRBV06*	*TCRBD01-01*01*	*TCRBJ01-05*01*
CASSSGAREGTEAFF	249	0.177169976	45	*TCRBV07-09*	*TCRBD02-01*02*	*TCRBJ01-01*01*
CASSQGAPGSGYEQYF	246	0.175035398	48	*TCRBV04-02*01*	*TCRBD02-01*02*	*TCRBJ02-07*01*
CASSLIGTLSTDTQYF	245	0.174323872	48	*TCRBV12*	*TCRBD01-01*01*	*TCRBJ02-03*01*
CSAFSTTLNEQFF	245	0.174323872	39	*TCRBV20*	*unresolved*	*TCRBJ02-01*01*
CASRWTGGNTEAFF	244	0.173612346	42	*TCRBV19-01*	*TCRBD01-01*01*	*TCRBJ01-01*01*
CASSSNMGYTF	242	0.172189294	33	*TCRBV05-06*01*	*unresolved*	*TCRBJ01-02*01*
CASTLGGNQHF	241	0.171477768	33	*TCRBV06-01*01*	*TCRBD02-01*02*	*TCRBJ01-05*01*
CASIPHEGSSGNTIYF	240	0.170766242	48	*TCRBV28-01*01*	*unresolved*	*TCRBJ01-03*01*

CASRTLAGVRTGELFF	240	0.170766242	48	*TCRBV06*	*TCRBD02-01*02*	*TCRBJ02-02*01*
CASSRDHRTDYGYTF	240	0.170766242	45	*TCRBV04-02*01*	*TCRBD01-01*01*	*TCRBJ01-02*01*
CASSRRAGSYNEQFF	239	0.170054716	45	*TCRBV06-05*01*	*TCRBD01-01*01*	*TCRBJ02-01*01*
CASSPGQGTGYTF	237	0.168631664	39	*TCRBV05-06*01*	*TCRBD01-01*01*	*TCRBJ01-02*01*
CAITEGHSNTEAFF	235	0.167208612	42	*TCRBV10-03*01*	*TCRBD01-01*01*	*TCRBJ01-01*01*
CATSRGTGNTEAFF	234	0.166497086	42	*TCRBV15-01*01*	*TCRBD02-01*	*TCRBJ01-01*01*
CSAREQVMMNTEAFF	234	0.166497086	45	*TCRBV20*	*TCRBD01-01*01*	*TCRBJ01-01*01*
CASSLGGTEYYGYTF	233	0.16578556	45	*TCRBV07-09*	*TCRBD01-01*01*	*TCRBJ01-02*01*
CASSVDGRALDTQYF	232	0.165074034	45	*TCRBV09-01*	*TCRBD02-01*02*	*TCRBJ02-03*01*
CASSLGGPFEAFF	231	0.164362508	39	*TCRBV07-02*01*	*unresolved*	*TCRBJ01-01*01*
CASSYSSLSTKNYEQYF	231	0.164362508	51	*TCRBV06*	*unresolved*	*TCRBJ02-07*01*
CASSQRPRNFEAFF	229	0.162939456	42	*TCRBV23-01*01*	*unresolved*	*TCRBJ01-01*01*
CASSSIDRFTEAFF	229	0.162939456	42	*TCRBV27-01*01*	*TCRBD01-01*01*	*TCRBJ01-01*01*
CASLRGDVGMNIQYF	227	0.161516404	45	*TCRBV02-01*01*	*TCRBD01-01*01*	*TCRBJ02-04*01*
CASRQTTSGNTIYF	227	0.161516404	42	*TCRBV02-01*01*	*TCRBD02-01*	*TCRBJ01-03*01*
CASSLVGPGELFF	225	0.160093352	39	*TCRBV05-01*01*	*TCRBD01-01*01*	*TCRBJ02-02*01*
CASSTSYRENTEAFF	224	0.159381826	45	*TCRBV06-06*	*TCRBD01-01*01*	*TCRBJ01-01*01*
CASSLARGSETQYF	223	0.1586703	42	*TCRBV05-01*01*	*unresolved*	*TCRBJ02-05*01*
CASSEQGFNEQYF	221	0.157247248	39	*TCRBV02-01*01*	*TCRBD01-01*01*	*TCRBJ02-07*01*
CASSGRGPSYGYTF	221	0.157247248	42	*TCRBV11-02*02*	*TCRBD01-01*01*	*TCRBJ01-02*01*
CASSLYDRESVLTF	221	0.157247248	42	*TCRBV27-01*01*	*TCRBD01-01*01*	*TCRBJ02-06*01*
CASSLFTGRPYEQYF	220	0.156535722	45	*TCRBV27-01*01*	*TCRBD01-01*01*	*TCRBJ02-07*01*
CASSQWTGPPGNTIYF	220	0.156535722	48	*TCRBV06*	*TCRBD01-01*01*	*TCRBJ01-03*01*
CASSSEGQYGYTF	220	0.156535722	39	*TCRBV05-01*01*	*TCRBD01-01*01*	*TCRBJ01-02*01*
CATREQYF	220	0.156535722	24	*TCRBV05-01*01*	*TCRBD02-01*	*TCRBJ02-07*01*
CASSPPSSAGNTIYF	219	0.155824196	45	*TCRBV18-01*01*	*TCRBD02-01*	*TCRBJ01-03*01*
CASSPQAGYNEKLFF	219	0.155824196	45	*TCRBV04-03*01*	*TCRBD01-01*01*	*TCRBJ01-04*01*
CASSEGDPSGNTIYF	218	0.15511267	45	*TCRBV06-01*01*	*unresolved*	*TCRBJ01-03*01*
CASSPRTALSYEQYF	218	0.15511267	45	*TCRBV05-06*01*	*TCRBD01-01*01*	*TCRBJ02-07*01*
CASRGFPLRNEQFF	214	0.152266566	42	*TCRBV06-05*01*	*unresolved*	*TCRBJ02-01*01*
CASSERLGNQPQHF	214	0.152266566	42	*TCRBV02-01*01*	*unresolved*	*TCRBJ01-05*01*
CASSLGLGGGNTGELFF	214	0.152266566	51	*TCRBV07-09*	*unresolved*	*TCRBJ02-02*01*

CASSLFAGEGYGYTF	213	0.15155504	45	*TCRBV05-05*01*	*TCRBD02-01*02*	*TCRBJ01-02*01*
CASSDGPGLAGNHFSYEQYF	210	0.149420462	60	*TCRBV12*	*TCRBD02-01*01*	*TCRBJ02-07*01*
CASSSGGGAAYEQYF	210	0.149420462	45	*TCRBV07-09*	*TCRBD02-01*01*	*TCRBJ02-07*01*
CASGEANSPLHF	209	0.148708936	36	*TCRBV23-01*01*	*unresolved*	*TCRBJ01-06*01*
CASSLNPGGRNTGELFF	209	0.148708936	51	*TCRBV05-01*01*	*TCRBD02-01*01*	*TCRBJ02-02*01*
CASSFDRLLTGENEQFF	206	0.146574358	51	*TCRBV28-01*01*	*TCRBD01-01*01*	*TCRBJ02-01*01*
CASSPPGDEQFF	205	0.145862832	36	*TCRBV27-01*01*	*TCRBD01-01*01*	*TCRBJ02-01*01*
CAISGSDSNSPLHF	203	0.14443978	42	*TCRBV10-03*01*	*TCRBD02-01*	*TCRBJ01-06*01*
CASNRRTNWNTGELFF	202	0.143728254	48	*TCRBV06*	*unresolved*	*TCRBJ02-02*01*
CASSPGPARENPLHF	202	0.143728254	45	*TCRBV18-01*01*	*unresolved*	*TCRBJ01-06*01*
CASNHRTGPNYEQYF	201	0.143016728	45	*TCRBV12*	*TCRBD01-01*01*	*TCRBJ02-07*01*
CASSFDSPNYGYTF	201	0.143016728	42	*TCRBV12*	*TCRBD01-01*01*	*TCRBJ01-02*01*
CASSQEFGIEQFF	201	0.143016728	39	*TCRBV04-02*01*	*TCRBD01-01*01*	*TCRBJ02-01*01*
CASRGGNQPQHF	199	0.141593676	36	*TCRBV10-02*01*	*TCRBD02-01*02*	*TCRBJ01-05*01*
CASSNLPLDRNTEAFF	198	0.14088215	48	*TCRBV18-01*01*	*TCRBD01-01*01*	*TCRBJ01-01*01*
CASSLAGLNQPQHF	197	0.140170624	42	*TCRBV05-05*01*	*TCRBD01-01*01*	*TCRBJ01-05*01*
CASSEAFSWVNTEAFF	195	0.138747572	48	*TCRBV02-01*01*	*unresolved*	*TCRBJ01-01*01*
CASSESAGAGPPQHF	195	0.138747572	45	*TCRBV02-01*01*	*TCRBD01-01*01*	*TCRBJ01-05*01*
CASSQEGGRTAF	195	0.138747572	36	*TCRBV04-01*01*	*unresolved*	*TCRBJ01-02*01*
CASSPPGSLPDTQYF	194	0.138036046	45	*TCRBV18-01*01*	*TCRBD02-01*	*TCRBJ02-03*01*
CASSPRGPTNTEAFF	194	0.138036046	45	*TCRBV28-01*01*	*TCRBD01-01*01*	*TCRBJ01-01*01*
CASSYGRNYGYTF	193	0.13732452	39	*TCRBV27-01*01*	*unresolved*	*TCRBJ01-02*01*
CAEGTGLYGYTF	192	0.136612994	36	*TCRBV02-01*01*	*TCRBD01-01*01*	*TCRBJ01-02*01*
CASSMAGYDYEQYF	191	0.135901468	42	*TCRBV19-01*	*TCRBD02-01*01*	*TCRBJ02-07*01*
CASRRGAVETQYF	190	0.135189942	39	*TCRBV06-05*01*	*unresolved*	*TCRBJ02-05*01*
CASSQGGNSYEQYF	187	0.133055364	42	*TCRBV03*	*unresolved*	*TCRBJ02-07*01*
CASSYEGASNQPQHF	187	0.133055364	45	*TCRBV06-09*01*	*TCRBD02-01*02*	*TCRBJ01-05*01*
CASRIPTGDDYGYTF	186	0.132343838	45	*TCRBV06-06*	*TCRBD01-01*01*	*TCRBJ01-02*01*
CASRLGDRGSEQYF	185	0.131632312	42	*TCRBV06-01*01*	*TCRBD01-01*01*	*TCRBJ02-07*01*
CASSFWGPMNNEQFF	185	0.131632312	45	*TCRBV05-04*01*	*unresolved*	*TCRBJ02-01*01*
CASRTDVGGFYGYTF	184	0.130920786	45	*TCRBV05-01*01*	*TCRBD02-01*02*	*TCRBJ01-02*01*
CASSQLAGGGNEQFF	184	0.130920786	45	*TCRBV07-02*01*	*TCRBD02-01*01*	*TCRBJ02-01*01*

CASRGQTNTEAFF	183	0.13020926	39	*TCRBV05-01*01*	*TCRBD01-01*01*	*TCRBJ01-01*01*
CASSESVFGEAFF	183	0.13020926	39	*TCRBV25-01*01*	*TCRBD01-01*01*	*TCRBJ01-01*01*
CASSPQTGGGNTEAFF	183	0.13020926	48	*TCRBV28-01*01*	*TCRBD01-01*01*	*TCRBJ01-01*01*
CASSQGSPLDKYGYTF	183	0.13020926	48	*TCRBV04-01*01*	*TCRBD01-01*01*	*TCRBJ01-02*01*
CASSLEGKYVYTF	179	0.127363156	39	*TCRBV05-01*01*	*unresolved*	*TCRBJ01-02*01*
CASSLWRGTDTQYF	179	0.127363156	42	*TCRBV12*	*TCRBD02-01*02*	*TCRBJ02-03*01*
CASSLRDTGNTIYF	177	0.125940104	42	*TCRBV05-01*01*	*TCRBD01-01*01*	*TCRBJ01-03*01*
CATSREIYNEQFF	176	0.125228578	39	*TCRBV15-01*01*	*unresolved*	*TCRBJ02-01*01*
CASSQGLGPGELFF	175	0.124517052	42	*TCRBV07-06*01*	*TCRBD01-01*01*	*TCRBJ02-02*01*
CASDSGGVNQPQHF	174	0.123805526	42	*TCRBV28-01*01*	*unresolved*	*TCRBJ01-05*01*
CASSETWDQPQHF	174	0.123805526	39	*TCRBV06-01*01*	*TCRBD02-01*	*TCRBJ01-05*01*
CASSFAKQGGDHEQYF	174	0.123805526	48	*TCRBV07-06*01*	*TCRBD01-01*01*	*TCRBJ02-07*01*
CASSLDRGTYSGANVLTF	174	0.123805526	54	*TCRBV07-02*01*	*TCRBD01-01*01*	*TCRBJ02-06*01*
CASSPVDTQYF	174	0.123805526	33	*TCRBV02-01*01*	*TCRBD02-01*	*TCRBJ02-03*01*
CASSYPEDTQYF	174	0.123805526	36	*TCRBV06*	*unresolved*	*TCRBJ02-03*01*
CSVEDKQEGYEKLFF	174	0.123805526	45	*TCRBV29-01*01*	*TCRBD02-01*02*	*TCRBJ01-04*01*
CASSPRTAIQEQYF	173	0.123094	42	*TCRBV18-01*01*	*TCRBD01-01*01*	*TCRBJ02-07*01*
CASSQDRWDIDNTEAFF	173	0.123094	51	*TCRBV04-01*01*	*TCRBD01-01*01*	*TCRBJ01-01*01*
CASSQSGTGTFGELFF	171	0.121670948	48	*TCRBV06*	*TCRBD01-01*01*	*TCRBJ02-02*01*
CASSEGGNQPQHF	170	0.120959422	39	*TCRBV06-01*01*	*unresolved*	*TCRBJ01-05*01*
CAIVGELGLAGNHEQFF	168	0.11953637	51	*TCRBV10-03*01*	*TCRBD02-01*	*TCRBJ02-01*01*
CASSDQGGDQPQHF	168	0.11953637	42	*TCRBV18-01*01*	*TCRBD02-01*02*	*TCRBJ01-05*01*
CASSLDWNTEAFF	168	0.11953637	39	*TCRBV11-02*02*	*unresolved*	*TCRBJ01-01*01*
CASSRGQNTDTQYF	168	0.11953637	42	*TCRBV05-01*01*	*unresolved*	*TCRBJ02-03*01*
CASSLASQNYGYTF	167	0.118824844	42	*TCRBV05-04*01*	*TCRBD01-01*01*	*TCRBJ01-02*01*
CASSLDPGGNGYTF	166	0.118113318	42	*TCRBV07-03*01*	*TCRBD01-01*01*	*TCRBJ01-02*01*
CASSSDTHPWELFF	166	0.118113318	42	*TCRBV18-01*01*	*TCRBD01-01*01*	*TCRBJ02-02*01*
CSAGVTGGGGSPLHF	166	0.118113318	45	*TCRBV20*	*TCRBD01-01*01*	*TCRBJ01-06*01*
CASINRSSGRFWSETQYF	165	0.117401792	54	*TCRBV28-01*01*	*TCRBD02-01*02*	*TCRBJ02-05*01*
CASSEDSSSSYEQYF	165	0.117401792	45	*TCRBV13-01*01*	*TCRBD01-01*01*	*TCRBJ02-07*01*
CASSGREGPGYTF	165	0.117401792	39	*TCRBV02-01*01*	*TCRBD02-01*02*	*TCRBJ01-02*01*
CASSLLAGSYNEQFF	163	0.11597874	45	*TCRBV07-03*01*	*TCRBD02-01*	*TCRBJ02-01*01*

CASSPDSYNEQFF	163	0.11597874	39	*TCRBV05-01*01*	*TCRBD01-01*01*	*TCRBJ02-01*01*
CASSQAFGVPGELFF	163	0.11597874	45	*TCRBV04-02*01*	*unresolved*	*TCRBJ02-02*01*
CASNPAGGPREKLFF	162	0.115267214	45	*TCRBV06-01*01*	*TCRBD02-01*	*TCRBJ01-04*01*
CASSPWGGYGYTF	162	0.115267214	39	*TCRBV02-01*01*	*TCRBD01-01*01*	*TCRBJ01-02*01*
CASSFGSNQPQHF	161	0.114555688	39	*TCRBV27-01*01*	*unresolved*	*TCRBJ01-05*01*
CASSLNHLHRQANYNSPLHF	161	0.114555688	60	*TCRBV05-01*01*	*TCRBD01-01*01*	*TCRBJ01-06*01*
CASSPGQGNSYEQYF	161	0.114555688	45	*TCRBV07-09*	*TCRBD01-01*01*	*TCRBJ02-07*01*
CASSRDKPLAGGNQETQYF	160	0.113844162	57	*TCRBV04-01*01*	*TCRBD02-01*01*	*TCRBJ02-05*01*
CATKKGRRATNEKLFF	160	0.113844162	48	*TCRBV15-01*01*	*unresolved*	*TCRBJ01-04*01*
CASSRGDRGRNTEAFF	158	0.11242111	48	*TCRBV05-01*01*	*TCRBD01-01*01*	*TCRBJ01-01*01*
CASTGWQMRDSNQPQHF	158	0.11242111	51	*TCRBV19-01*	*TCRBD02-01*02*	*TCRBJ01-05*01*
CASRGQGSAYNSPLHF	157	0.111709584	48	*TCRBV07-09*	*TCRBD01-01*01*	*TCRBJ01-06*01*
CASSEVVAAGNQPQHF	156	0.110998058	48	*TCRBV06-01*01*	*TCRBD02-01*	*TCRBJ01-05*01*
CASSPYTGELFF	156	0.110998058	36	*TCRBV07-02*01*	*TCRBD02-01*	*TCRBJ02-02*01*
CATLQGPTEAFF	155	0.110286532	36	*TCRBV19-01*	*TCRBD01-01*01*	*TCRBJ01-01*01*
CASSESRSGGNEKLFF	154	0.109575006	48	*TCRBV05-01*01*	*TCRBD01-01*01*	*TCRBJ01-04*01*
CASSSSGRATGELFF	154	0.109575006	45	*TCRBV07-09*	*TCRBD02-01*02*	*TCRBJ02-02*01*
CSVEISKGTGNYGYTF	154	0.109575006	48	*TCRBV29-01*01*	*TCRBD01-01*01*	*TCRBJ01-02*01*
CASSDRQVLGYGYTF	153	0.10886348	45	*TCRBV10-01*	*TCRBD01-01*01*	*TCRBJ01-02*01*
CASSTGLTAISYGYTF	153	0.10886348	48	*TCRBV07-07*01*	*unresolved*	*TCRBJ01-02*01*
CATSRDPDSHEQYF	153	0.10886348	42	*TCRBV15-01*01*	*TCRBD02-01*	*TCRBJ02-07*01*
CASATDPNEQFF	152	0.108151953	36	*TCRBV06-06*	*unresolved*	*TCRBJ02-01*01*
CASSPRTGFEAFF	152	0.108151953	39	*TCRBV27-01*01*	*TCRBD01-01*01*	*TCRBJ01-01*01*
CASSSRTSSVGQFF	152	0.108151953	42	*TCRBV03*	*TCRBD02-01*	*TCRBJ02-01*01*
CAIKGGVSGYTF	151	0.107440427	36	*TCRBV10-01*	*TCRBD02-01*02*	*TCRBJ01-02*01*
CASSLPDRGTNEKLFF	151	0.107440427	48	*TCRBV28-01*01*	*TCRBD01-01*01*	*TCRBJ01-04*01*
CASSLTPTGQDEKLFF	151	0.107440427	48	*TCRBV28-01*01*	*TCRBD01-01*01*	*TCRBJ01-04*01*
CASSDRSQLSYEQYF	150	0.106728901	45	*TCRBV06-01*01*	*TCRBD02-01*02*	*TCRBJ02-07*01*
CASSEMKGGRGGYTF	150	0.106728901	45	*TCRBV10-02*01*	*TCRBD02-01*01*	*TCRBJ01-02*01*
CASSFGSGNQPQHF	150	0.106728901	42	*TCRBV12*	*unresolved*	*TCRBJ01-05*01*
CASSLGRIYEQYF	150	0.106728901	39	*TCRBV05-08*01*	*TCRBD02-01*02*	*TCRBJ02-07*01*
CASSLSQGNYGYTF	150	0.106728901	42	*TCRBV27-01*01*	*TCRBD01-01*01*	*TCRBJ01-02*01*

CASRRDRGPREQFF	149	0.106017375	42	*TCRBV02-01*01*	*TCRBD01-01*01*	*TCRBJ02-01*01*
CASSARAGLLYYGYTF	149	0.106017375	48	*TCRBV07-09*	*TCRBD02-01*	*TCRBJ01-02*01*
CASSQVPRGTDTQYF	149	0.106017375	45	*TCRBV04-01*01*	*TCRBD02-01*	*TCRBJ02-03*01*
CASSDFEGGEQFF	147	0.104594323	39	*TCRBV06-04*	*unresolved*	*TCRBJ02-01*01*
CASSDRDLFF	147	0.104594323	30	*TCRBV07-09*	*TCRBD02-01*	*TCRBJ02-01*01*
CASSGGEPLRGWNEQFF	147	0.104594323	51	*TCRBV18-01*01*	*TCRBD02-01*01*	*TCRBJ02-01*01*
CASSHSTAYNVLTF	146	0.103882797	42	*TCRBV07-09*	*TCRBD01-01*01*	*TCRBJ02-06*01*
CASSLFRNTEAFF	145	0.103171271	39	*TCRBV27-01*01*	*TCRBD02-01*	*TCRBJ01-01*01*
CASSVGGLASDTQYF	145	0.103171271	45	*TCRBV09-01*	*TCRBD02-01*	*TCRBJ02-03*01*
CASSLSRTGGYYGYTF	144	0.102459745	48	*TCRBV27-01*01*	*TCRBD01-01*01*	*TCRBJ01-02*01*
CASGGGTSGTDTQYF	143	0.101748219	45	*TCRBV06-05*01*	*TCRBD02-01*	*TCRBJ02-03*01*
CSALGGYTEAFF	143	0.101748219	36	*TCRBV20*	*unresolved*	*TCRBJ01-01*01*
CASSTPGATDTQYF	142	0.101036693	42	*TCRBV04-01*01*	*unresolved*	*TCRBJ02-03*01*
CASNTGEGQFF	141	0.100325167	33	*TCRBV06-05*01*	*TCRBD02-01*02*	*TCRBJ02-01*01*
CASSGTGTGGGNEQFF	141	0.100325167	48	*TCRBV05-01*01*	*TCRBD01-01*01*	*TCRBJ02-01*01*
CSARDGRRNTEAFF	141	0.100325167	42	*TCRBV20*	*TCRBD02-01*02*	*TCRBJ01-01*01*
CASGPEGLATYNEQFF	140	0.099613641	48	*TCRBV12-05*01*	*TCRBD02-01*	*TCRBJ02-01*01*
CASSSTGGYNEQFF	140	0.099613641	42	*TCRBV05-05*01*	*TCRBD01-01*01*	*TCRBJ02-01*01*
CASSHKHRVSNQPQHF	139	0.098902115	48	*TCRBV04-02*01*	*TCRBD01-01*01*	*TCRBJ01-05*01*
CASSLVGGIFGEAFF	139	0.098902115	45	*TCRBV28-01*01*	*TCRBD02-01*02*	*TCRBJ01-01*01*
CASSEALAGGPDTQYF	137	0.097479063	48	*TCRBV06-01*01*	*TCRBD02-01*02*	*TCRBJ02-03*01*
CASSLLTGYNEQFF	137	0.097479063	42	*TCRBV27-01*01*	*TCRBD01-01*01*	*TCRBJ02-01*01*
CASSAQRDRRTYEQYF	136	0.096767537	48	*TCRBV02-01*01*	*TCRBD01-01*01*	*TCRBJ02-07*01*
CASSPRTASPWKYGYTF	136	0.096767537	51	*TCRBV07-08*01*	*TCRBD02-01*	*TCRBJ01-02*01*
CASTIPGHSGEQFF	136	0.096767537	42	*TCRBV19-01*	*TCRBD01-01*01*	*TCRBJ02-01*01*
CSAGTGLQPQHF	136	0.096767537	36	*TCRBV20*	*TCRBD01-01*01*	*TCRBJ01-05*01*
CASSPQDTGELFF	135	0.096056011	39	*TCRBV07-09*	*TCRBD01-01*01*	*TCRBJ02-02*01*
CASSRGQGAGEAFF	135	0.096056011	42	*TCRBV05-01*01*	*TCRBD01-01*01*	*TCRBJ01-01*01*
CASRSGVLIQETQYF	134	0.095344485	45	*TCRBV12*	*TCRBD02-01*01*	*TCRBJ02-05*01*
CASSLVGKQPQHF	134	0.095344485	39	*TCRBV28-01*01*	*unresolved*	*TCRBJ01-05*01*
CASSQVRKSAGTGLYEQYF	134	0.095344485	57	*TCRBV14-01*01*	*TCRBD01-01*01*	*TCRBJ02-07*01*
CASKGGTGIYNEQFF	132	0.093921433	45	*TCRBV02-01*01*	*TCRBD01-01*01*	*TCRBJ02-01*01*

CASSFIGAGANVLTF	132	0.093921433	45	*TCRBV12*	*unresolved*	*TCRBJ02-06*01*
CASSLDRAGGYTF	132	0.093921433	39	*TCRBV05-01*01*	*TCRBD01-01*01*	*TCRBJ01-02*01*
CASSASGRGGELFF	131	0.093209907	42	*TCRBV18-01*01*	*TCRBD02-01*02*	*TCRBJ02-02*01*
CASSLRGGDRSSYEQYF	131	0.093209907	51	*TCRBV07-02*01*	*TCRBD01-01*01*	*TCRBJ02-07*01*
CASSQVLAGTSGTQYF	131	0.093209907	48	*TCRBV04-01*01*	*TCRBD02-01*01*	*TCRBJ02-05*01*
CSVERGQGMNTEAFF	131	0.093209907	45	*TCRBV29-01*01*	*TCRBD01-01*01*	*TCRBJ01-01*01*
CASSELASGGITDTQYF	130	0.092498381	51	*TCRBV02-01*01*	*TCRBD02-01*01*	*TCRBJ02-03*01*
CASSGRTSGGAPNEQFF	130	0.092498381	51	*TCRBV05-01*01*	*TCRBD02-01*01*	*TCRBJ02-01*01*
CASSSGNYGYTF	130	0.092498381	36	*TCRBV19-01*	*TCRBD02-01*	*TCRBJ01-02*01*
CAWSPGLMNTEAFF	130	0.092498381	42	*TCRBV30-01*01*	*TCRBD02-01*	*TCRBJ01-01*01*
CSARPRGGNVHEQYF	130	0.092498381	45	*TCRBV20*	*TCRBD02-01*01*	*TCRBJ02-07*01*
CASSFRGSGYEQYF	129	0.091786855	42	*TCRBV28-01*01*	*TCRBD02-01*02*	*TCRBJ02-07*01*
CASSLERPTYNEQFF	129	0.091786855	45	*TCRBV05-01*01*	*TCRBD02-01*02*	*TCRBJ02-01*01*
CASSPRGQGYYEQYF	129	0.091786855	45	*TCRBV13-01*01*	*TCRBD01-01*01*	*TCRBJ02-07*01*
CASSPTGETNEKLFF	129	0.091786855	45	*TCRBV18-01*01*	*TCRBD01-01*01*	*TCRBJ01-04*01*
CASNIAGPAYEQYF	128	0.091075329	42	*TCRBV12*	*TCRBD02-01*01*	*TCRBJ02-07*01*
CASRTGGAPGFQYF	127	0.090363803	42	*TCRBV19-01*	*TCRBD01-01*01*	*TCRBJ02-03*01*
CASSIGQKDTQYF	127	0.090363803	39	*TCRBV12*	*TCRBD01-01*01*	*TCRBJ02-03*01*
CASSLATDTQYF	127	0.090363803	36	*TCRBV03*	*TCRBD02-01*	*TCRBJ02-03*01*
CASSPNNEQFF	127	0.090363803	33	*TCRBV07-06*01*	*unresolved*	*TCRBJ02-01*01*
CATSDWGTDTQYF	127	0.090363803	39	*TCRBV24*	*unresolved*	*TCRBJ02-03*01*
CAIGGGEGYGYTF	126	0.089652277	39	*TCRBV10-03*01*	*TCRBD01-01*01*	*TCRBJ01-02*01*
CASSLYTGGWYGYTF	126	0.089652277	45	*TCRBV27-01*01*	*TCRBD01-01*01*	*TCRBJ01-02*01*
CASNPPGQGVTGELFF	125	0.088940751	48	*TCRBV19-01*	*TCRBD01-01*01*	*TCRBJ02-02*01*
CASSDAGYGEQFF	125	0.088940751	39	*TCRBV06-04*	*TCRBD02-01*	*TCRBJ02-01*01*
CASSEGGWYEQYF	125	0.088940751	39	*TCRBV25-01*01*	*TCRBD01-01*01*	*TCRBJ02-07*01*
CASSKRKRAGLYEQYF	125	0.088940751	48	*TCRBV28-01*01*	*TCRBD02-01*	*TCRBJ02-07*01*
CAGRLAGKDTQYF	124	0.088229225	39	*TCRBV18-01*01*	*TCRBD02-01*01*	*TCRBJ02-03*01*
CASSSLGRVGEKLFF	124	0.088229225	45	*TCRBV07-02*01*	*TCRBD01-01*01*	*TCRBJ01-04*01*
CASDWTSGGNNEQFF	123	0.087517699	45	*TCRBV10-02*01*	*TCRBD02-01*	*TCRBJ02-01*01*
CASSFYRDISYEQYF	123	0.087517699	45	*TCRBV27-01*01*	*TCRBD01-01*01*	*TCRBJ02-07*01*
CASSPPGTKNTGELFF	123	0.087517699	48	*TCRBV11-02*02*	*TCRBD02-01*	*TCRBJ02-02*01*

CASSVAAGANEKLFF	123	0.087517699	45	*TCRBV09-01*	*TCRBD02-01*01*	*TCRBJ01-04*01*
CASSYDLSYEQYF	123	0.087517699	39	*TCRBV06-06*	*unresolved*	*TCRBJ02-07*01*
CASSDPHEYGYTF	122	0.086806173	39	*TCRBV25-01*01*	*unresolved*	*TCRBJ01-02*01*
CASSRLKTDTQYF	122	0.086806173	39	*TCRBV04-01*01*	*unresolved*	*TCRBJ02-03*01*
CASSTGLAGGRVGEQYF	122	0.086806173	51	*TCRBV05-01*01*	*TCRBD02-01*01*	*TCRBJ02-07*01*
CASSVGADTGELFF	122	0.086806173	42	*TCRBV09-01*	*TCRBD01-01*01*	*TCRBJ02-02*01*
CASSYWTGTKAGYTF	120	0.085383121	45	*TCRBV02-01*01*	*TCRBD01-01*01*	*TCRBJ01-02*01*
CASSIDGSSYNEQFF	119	0.084671595	45	*TCRBV13-01*01*	*unresolved*	*TCRBJ02-01*01*
CASSLYGGDKNIQYF	119	0.084671595	45	*TCRBV05-05*01*	*TCRBD02-01*02*	*TCRBJ02-04*01*
CASSPLGYEQYF	119	0.084671595	36	*TCRBV12*	*unresolved*	*TCRBJ02-07*01*
CASSSTWGQEYGYTF	119	0.084671595	45	*TCRBV28-01*01*	*TCRBD01-01*01*	*TCRBJ01-02*01*
CASSYSGNRWDTQYF	119	0.084671595	45	*TCRBV06*	*unresolved*	*TCRBJ02-03*01*
CSARQVGQPQHF	119	0.084671595	36	*TCRBV20*	*TCRBD01-01*01*	*TCRBJ01-05*01*
CASSPFPGLAGGVGWETQYF	118	0.083960069	60	*TCRBV18-01*01*	*TCRBD02-01*02*	*TCRBJ02-05*01*
CASSPRGTGGSYEQYF	118	0.083960069	48	*TCRBV07-09*	*TCRBD01-01*01*	*TCRBJ02-07*01*
CATTRDVSSYEQYF	117	0.083248543	42	*TCRBV28-01*01*	*unresolved*	*TCRBJ02-07*01*
CSATQGGNQPQHF	117	0.083248543	39	*TCRBV20*	*TCRBD01-01*01*	*TCRBJ01-05*01*
CASSESMGSETQYF	116	0.082537017	42	*TCRBV07-09*	*TCRBD01-01*01*	*TCRBJ02-05*01*
CASSLVWDTQYF	116	0.082537017	36	*TCRBV12*	*unresolved*	*TCRBJ02-03*01*
CATSSGGNSPLHF	116	0.082537017	39	*TCRBV24*	*TCRBD01-01*01*	*TCRBJ01-06*01*
CASRGLALGETQYF	115	0.081825491	42	*TCRBV12*	*TCRBD02-01*	*TCRBJ02-05*01*
CASSIGTTSNQPQHF	115	0.081825491	45	*TCRBV02-01*01*	*TCRBD01-01*01*	*TCRBJ01-05*01*
CASSISDQPQHF	114	0.081113965	36	*TCRBV19-01*	*TCRBD02-01*	*TCRBJ01-05*01*
CASSLDPTGGTQYF	114	0.081113965	42	*TCRBV07-06*01*	*TCRBD01-01*01*	*TCRBJ02-05*01*
CASSQGASGGGGDYGYTF	114	0.081113965	54	*TCRBV13-01*01*	*TCRBD01-01*01*	*TCRBJ01-02*01*
CASSVGVPAGVHNEQFF	114	0.081113965	51	*TCRBV09-01*	*TCRBD02-01*02*	*TCRBJ02-01*01*
CATSDGDRVHANTIYF	114	0.081113965	48	*TCRBV24*	*TCRBD01-01*01*	*TCRBJ01-03*01*
CSVRGLAGSAYEQYF	114	0.081113965	45	*TCRBV20*	*TCRBD02-01*01*	*TCRBJ02-07*01*
CASSLPTFGMNTEAFF	113	0.080402439	48	*TCRBV05-06*01*	*unresolved*	*TCRBJ01-01*01*
CASSLQQHGELFF	113	0.080402439	39	*TCRBV12*	*TCRBD01-01*01*	*TCRBJ02-02*01*
CASSPFRGQGLSPLHF	113	0.080402439	48	*TCRBV13-01*01*	*unresolved*	*TCRBJ01-06*01*
CASSRTGTEQYF	113	0.080402439	36	*TCRBV28-01*01*	*TCRBD01-01*01*	*TCRBJ02-07*01*

CASTWLAGDGEQFF	113	0.080402439	42	*TCRBV05-01*01*	*TCRBD02-01*02*	*TCRBJ02-01*01*
CSASLSDPEQYF	113	0.080402439	36	*TCRBV20*	*unresolved*	*TCRBJ02-07*01*
CASRGDIRGNEQFF	112	0.079690913	42	*TCRBV02-01*01*	*TCRBD01-01*01*	*TCRBJ02-01*01*
CASSEYGTSGYTF	112	0.079690913	39	*TCRBV25-01*01*	*TCRBD01-01*01*	*TCRBJ01-02*01*
CASSFPALEQYF	112	0.079690913	36	*TCRBV06*	*TCRBD02-01*	*TCRBJ02-07*01*
CATSRDRTNYEQYF	112	0.079690913	42	*TCRBV15-01*01*	*TCRBD02-01*	*TCRBJ02-07*01*
CASTYGQETQYF	111	0.078979387	36	*TCRBV02-01*01*	*TCRBD02-01*	*TCRBJ02-05*01*
CASSIDETYEQYF	110	0.078267861	39	*TCRBV19-01*	*unresolved*	*TCRBJ02-07*01*
CASSLGNGETQYF	110	0.078267861	39	*TCRBV05-06*01*	*unresolved*	*TCRBJ02-05*01*
CASSVGGTGTGYGYTF	108	0.076844809	48	*TCRBV09-01*	*TCRBD01-01*01*	*TCRBJ01-02*01*
CASSLGNVPNYGYTF	107	0.076133283	45	*TCRBV05-01*01*	*unresolved*	*TCRBJ01-02*01*
CASSTRDLNTQYF	107	0.076133283	39	*TCRBV19-01*	*unresolved*	*TCRBJ02-03*01*
CASRTNIRTNEKLFF	106	0.075421757	45	*TCRBV27-01*01*	*unresolved*	*TCRBJ01-04*01*
CASSLLGTLSTDTQYF	106	0.075421757	48	*TCRBV28-01*01*	*unresolved*	*TCRBJ02-03*01*
CASSPLGEAAGGYTF	106	0.075421757	45	*TCRBV03*	*unresolved*	*TCRBJ01-02*01*
CASSWSQRGSLHEQFF	106	0.075421757	48	*TCRBV07-09*	*TCRBD02-01*01*	*TCRBJ02-01*01*
CAWTSGYEQYF	106	0.075421757	33	*TCRBV30-01*01*	*TCRBD02-01*	*TCRBJ02-07*01*
CASSIHRGYSTDTQYF	105	0.074710231	48	*TCRBV05-08*01*	*unresolved*	*TCRBJ02-03*01*
CASSLVRGRYEQYF	105	0.074710231	42	*TCRBV27-01*01*	*TCRBD02-01*02*	*TCRBJ02-07*01*
CATSEQGARNNEQFF	105	0.074710231	45	*TCRBV24*	*TCRBD01-01*01*	*TCRBJ02-01*01*
CASRTGHWVYEQYF	104	0.073998705	42	*TCRBV06-01*01*	*TCRBD01-01*01*	*TCRBJ02-07*01*
CASSLTDRFGMETQYF	104	0.073998705	48	*TCRBV07-07*01*	*TCRBD01-01*01*	*TCRBJ02-05*01*
CATLLGQVTIGYTF	104	0.073998705	42	*TCRBV28-01*01*	*TCRBD01-01*01*	*TCRBJ01-02*01*
CAISDPNLDRYNEQFF	103	0.073287179	48	*TCRBV10-03*01*	*TCRBD01-01*01*	*TCRBJ02-01*01*
CASSQANHYNEQFF	103	0.073287179	42	*TCRBV03*	*unresolved*	*TCRBJ02-01*01*
CSALPPGQAFEQYF	103	0.073287179	42	*TCRBV20*	*TCRBD01-01*01*	*TCRBJ02-07*01*
CASSLSPGRMNTEAFF	102	0.072575653	48	*TCRBV27-01*01*	*unresolved*	*TCRBJ01-01*01*
CASSEGGNKNIQYF	101	0.071864127	42	*TCRBV25-01*01*	*unresolved*	*TCRBJ02-04*01*
CASSLAWVGNYGYTF	101	0.071864127	45	*TCRBV07-02*01*	*unresolved*	*TCRBJ01-02*01*
CASSPGLGPYNEQFF	101	0.071864127	45	*TCRBV12*	*TCRBD02-01*	*TCRBJ02-01*01*
CASSVGGSYTF	101	0.071864127	33	*TCRBV05-06*01*	*TCRBD02-01*01*	*TCRBJ01-02*01*
CSVGGNQPQHF	101	0.071864127	33	*TCRBV29-01*01*	*TCRBD02-01*02*	*TCRBJ01-05*01*

CASSPNGETQYF	100	0.071152601	36	*TCRBV06-05*01*	*unresolved*	*TCRBJ02-05*01*
CASSPRLGGPNNEQFF	100	0.071152601	48	*TCRBV18-01*01*	*TCRBD02-01*01*	*TCRBJ02-01*01*
CASSTRTDGHNEQFF	100	0.071152601	45	*TCRBV27-01*01*	*unresolved*	*TCRBJ02-01*01*
CASSEELAGGRDQETQYF	99	0.070441075	54	*TCRBV06-01*01*	*TCRBD02-01*01*	*TCRBJ02-05*01*
CASSPALLGPTDTQYF	99	0.070441075	48	*TCRBV28-01*01*	*unresolved*	*TCRBJ02-03*01*
CASSQAKSTEAFF	98	0.069729549	39	*TCRBV04-01*01*	*TCRBD02-01*02*	*TCRBJ01-01*01*
CASSYGQGVGEKLFF	98	0.069729549	45	*TCRBV05-01*01*	*TCRBD01-01*01*	*TCRBJ01-04*01*
CASSDDGQGNTGELFF	97	0.069018023	48	*TCRBV02-01*01*	*TCRBD01-01*01*	*TCRBJ02-02*01*
CASSFVGYETQYF	97	0.069018023	39	*TCRBV05-06*01*	*unresolved*	*TCRBJ02-05*01*
CTSRGFLGGLGYGYTF	97	0.069018023	48	*TCRBV01-01*01*	*TCRBD02-01*01*	*TCRBJ01-02*01*
CASSLRTSGFYNEQFF	96	0.068306497	48	*TCRBV12*	*TCRBD02-01*	*TCRBJ02-01*01*
CASSWTISSTDTQYF	96	0.068306497	45	*TCRBV06-04*	*TCRBD02-01*	*TCRBJ02-03*01*
CASSARVRRQETQYF	95	0.067594971	45	*TCRBV28-01*01*	*TCRBD02-01*	*TCRBJ02-05*01*
CASSAYEQYF	95	0.067594971	30	*TCRBV09-01*	*unresolved*	*TCRBJ02-07*01*
CASSLDSGTEAFF	95	0.067594971	39	*TCRBV12*	*TCRBD01-01*01*	*TCRBJ01-01*01*
CASSSSGRGTDTQYF	95	0.067594971	45	*TCRBV09-01*	*TCRBD02-01*02*	*TCRBJ02-03*01*
CAWTREGSPLHF	95	0.067594971	36	*TCRBV30-01*01*	*TCRBD02-01*02*	*TCRBJ01-06*01*
CSVEGFGGDYGYTF	95	0.067594971	42	*TCRBV29-01*01*	*unresolved*	*TCRBJ01-02*01*
CASRTPDGLKYEQYF	94	0.066883445	45	*TCRBV02-01*01*	*TCRBD02-01*	*TCRBJ02-07*01*
CASRGSNYGYTF	93	0.066171919	36	*TCRBV06-05*01*	*unresolved*	*TCRBJ01-02*01*
CASSLEDRGYEQYF	93	0.066171919	42	*TCRBV11-02*02*	*TCRBD01-01*01*	*TCRBJ02-07*01*
CAWSGQDNQPQHF	93	0.066171919	39	*TCRBV30-01*01*	*unresolved*	*TCRBJ01-05*01*
CSVEDGGFGYTF	93	0.066171919	36	*TCRBV29-01*01*	*unresolved*	*TCRBJ01-02*01*
CASSLVGTGNEKLFF	92	0.065460393	45	*TCRBV27-01*01*	*TCRBD01-01*01*	*TCRBJ01-04*01*
CASSWSYEQYF	92	0.065460393	33	*TCRBV11-02*02*	*TCRBD02-01*02*	*TCRBJ02-07*01*
CASSYSPGNGAEQYF	92	0.065460393	45	*TCRBV06-06*	*TCRBD01-01*01*	*TCRBJ02-07*01*
CSAPVRDSYGYTF	92	0.065460393	39	*TCRBV20*	*TCRBD02-01*	*TCRBJ01-02*01*
CAIRGLAGPYNEQFF	91	0.064748867	45	*TCRBV10-03*01*	*TCRBD02-01*01*	*TCRBJ02-01*01*
CASSLRQGGSPLHF	91	0.064748867	42	*TCRBV09-01*	*unresolved*	*TCRBJ01-06*01*
CASTSGGEDGTDTQYF	91	0.064748867	48	*TCRBV06-06*	*TCRBD02-01*01*	*TCRBJ02-03*01*
CASLDVLEAFF	90	0.064037341	33	*TCRBV06-01*01*	*unresolved*	*TCRBJ01-01*01*
CASSGASSTDTQYF	90	0.064037341	42	*TCRBV07-06*01*	*TCRBD02-01*02*	*TCRBJ02-03*01*

CASSLGGGADGLNQPQHF	90	0.064037341	54	*TCRBV05-01*01*	*TCRBD02-01*02*	*TCRBJ01-05*01*
CASSLGQGNPDTQYF	90	0.064037341	45	*TCRBV09-01*	*TCRBD01-01*01*	*TCRBJ02-03*01*
CASSQSWGQGPRETQYF	90	0.064037341	51	*TCRBV16-01*	*TCRBD01-01*01*	*TCRBJ02-05*01*
CASSRQQGDYGYTF	90	0.064037341	42	*TCRBV04-03*01*	*TCRBD01-01*01*	*TCRBJ01-02*01*
CASSSLAPTVYNEQFF	90	0.064037341	48	*TCRBV12*	*TCRBD02-01*	*TCRBJ02-01*01*
CSAGENQPQHF	90	0.064037341	33	*TCRBV20-01*01*	*TCRBD02-01*02*	*TCRBJ01-05*01*
CASSPRLAEANTGELFF	89	0.063325815	51	*TCRBV07-09*	*TCRBD02-01*	*TCRBJ02-02*01*
CASRPGLAGVLRKNEQFF	88	0.062614289	54	*TCRBV06*	*TCRBD02-01*02*	*TCRBJ02-01*01*
CASSPGNTEAFF	88	0.062614289	36	*TCRBV02-01*01*	*TCRBD01-01*01*	*TCRBJ01-01*01*
CASSSLAGGPYEQYF	88	0.062614289	45	*TCRBV07-02*01*	*TCRBD02-01*01*	*TCRBJ02-07*01*
CASSSSGGAVDTQYF	88	0.062614289	45	*TCRBV06-01*01*	*TCRBD02-01*01*	*TCRBJ02-03*01*
CASSTGLAGGPESYEQYF	88	0.062614289	54	*TCRBV05-01*01*	*TCRBD02-01*02*	*TCRBJ02-07*01*
CATSSTGTLSF	88	0.062614289	33	*TCRBV15-01*01*	*unresolved*	*TCRBJ02-06*01*
CSAAKPGVTYEQYF	88	0.062614289	42	*TCRBV20*	*TCRBD02-01*01*	*TCRBJ02-07*01*
CSAQTSGRHYEQFF	88	0.062614289	42	*TCRBV20*	*TCRBD02-01*02*	*TCRBJ02-01*01*
CASSATAGNQPQHF	87	0.061902763	42	*TCRBV06-05*01*	*TCRBD02-01*	*TCRBJ01-05*01*
CASSQEDTTYEQYF	87	0.061902763	42	*TCRBV04-01*01*	*TCRBD01-01*01*	*TCRBJ02-07*01*
CASSSAGYNEQFF	87	0.061902763	39	*TCRBV05-06*01*	*TCRBD02-01*01*	*TCRBJ02-01*01*
CASSWTSGGAETQYF	87	0.061902763	45	*TCRBV06*	*TCRBD02-01*01*	*TCRBJ02-05*01*
CASSPDDTQYF	86	0.061191237	33	*TCRBV18-01*01*	*unresolved*	*TCRBJ02-03*01*
CSARDLTSNEQYF	86	0.061191237	39	*TCRBV20*	*TCRBD02-01*	*TCRBJ02-07*01*
CASSASIRDLDVNTEAFF	85	0.060479711	54	*TCRBV05-08*01*	*TCRBD02-01*02*	*TCRBJ01-01*01*
CASSGTATNYGYTF	85	0.060479711	42	*TCRBV25-01*01*	*TCRBD01-01*01*	*TCRBJ01-02*01*
CASSLMTPLNTEAFF	85	0.060479711	45	*TCRBV05-01*01*	*TCRBD01-01*01*	*TCRBJ01-01*01*
CASSLSGSSYNEQFF	85	0.060479711	45	*TCRBV06-01*01*	*TCRBD02-01*02*	*TCRBJ02-01*01*
CASSNHRQGSGTGELFF	85	0.060479711	51	*TCRBV25-01*01*	*TCRBD01-01*01*	*TCRBJ02-02*01*
CASSRQGQETQYF	85	0.060479711	39	*TCRBV25-01*01*	*TCRBD01-01*01*	*TCRBJ02-05*01*
CASSSGGVYGELFF	85	0.060479711	42	*TCRBV28-01*01*	*TCRBD01-01*01*	*TCRBJ02-02*01*
CASSSPKGVRGYTF	85	0.060479711	42	*TCRBV07-09*	*TCRBD01-01*01*	*TCRBJ01-02*01*
CATSRDRGQPLYEQYF	85	0.060479711	48	*TCRBV15-01*01*	*TCRBD02-01*02*	*TCRBJ02-07*01*
CASGTSGSYEQYF	84	0.059768185	39	*TCRBV06-05*01*	*TCRBD02-01*02*	*TCRBJ02-07*01*
CASSKSLPGQFHSYEQYF	84	0.059768185	54	*TCRBV21-01*01*	*TCRBD01-01*01*	*TCRBJ02-07*01*

CASSLDGAVINTEAFF	84	0.059768185	48	*TCRBV12*	*unresolved*	*TCRBJ01-01*01*
CASSWTSGGYNEQFF	84	0.059768185	45	*TCRBV19-01*	*TCRBD02-01*01*	*TCRBJ02-01*01*
CASSDNRGLANTEAFF	83	0.059056659	48	*TCRBV09-01*	*TCRBD01-01*01*	*TCRBJ01-01*01*
CASSFKGNLGTGELFF	83	0.059056659	48	*TCRBV12*	*TCRBD01-01*01*	*TCRBJ02-02*01*
CASSIRPRGTVNYGYTF	83	0.059056659	51	*TCRBV19-01*	*TCRBD01-01*01*	*TCRBJ01-02*01*
CASSRTDSNQPQHF	83	0.059056659	42	*TCRBV10-02*01*	*TCRBD01-01*01*	*TCRBJ01-05*01*
CSALTSGRADEQFF	83	0.059056659	42	*TCRBV20*	*TCRBD02-01*02*	*TCRBJ02-01*01*
CASSKTSGSSYNEQFF	82	0.058345133	48	*TCRBV02-01*01*	*unresolved*	*TCRBJ02-01*01*
CASSLGPYSNQPQHF	82	0.058345133	45	*TCRBV03*	*unresolved*	*TCRBJ01-05*01*
CASSPEGETQYF	82	0.058345133	36	*TCRBV10-02*01*	*unresolved*	*TCRBJ02-05*01*
CASSSVDWGSPLHF	82	0.058345133	42	*TCRBV07-09*	*unresolved*	*TCRBJ01-06*01*
CASSYRDRDAGELFF	82	0.058345133	45	*TCRBV05-06*01*	*TCRBD01-01*01*	*TCRBJ02-02*01*
CASSPLTSGSSGEQYF	81	0.057633607	48	*TCRBV28-01*01*	*TCRBD02-01*02*	*TCRBJ02-07*01*
CASSPRWLERQYF	81	0.057633607	39	*TCRBV14-01*01*	*TCRBD02-01*02*	*TCRBJ02-03*01*
CAWSVLGETQYF	81	0.057633607	36	*TCRBV30-01*01*	*unresolved*	*TCRBJ02-05*01*
CSVEGGASRGSYEQYF	81	0.057633607	48	*TCRBV29-01*01*	*TCRBD01-01*01*	*TCRBJ02-07*01*
CASSRDQSTDTQYF	80	0.056922081	42	*TCRBV28-01*01*	*unresolved*	*TCRBJ02-03*01*
CASSTPTAGTSTVSSYNEQFF	80	0.056922081	63	*TCRBV19-01*	*TCRBD01-01*01*	*TCRBJ02-01*01*
CASSKGTQETQYF	79	0.056210555	39	*TCRBV05-04*01*	*unresolved*	*TCRBJ02-05*01*
CASSLARGDTGELFF	79	0.056210555	45	*TCRBV11-02*02*	*TCRBD02-01*02*	*TCRBJ02-02*01*
CASRIEGNIQYF	78	0.055499029	36	*TCRBV05-01*01*	*unresolved*	*TCRBJ02-04*01*
CASSWAEDYEQYF	78	0.055499029	39	*TCRBV05-01*01*	*TCRBD02-01*02*	*TCRBJ02-07*01*
CASYSGTGDQPQHF	78	0.055499029	42	*TCRBV28-01*01*	*TCRBD01-01*01*	*TCRBJ01-05*01*
CATRGQGGPEAFF	78	0.055499029	39	*TCRBV24*	*TCRBD01-01*01*	*TCRBJ01-01*01*
CSAPGIAQETQYF	78	0.055499029	39	*TCRBV20*	*TCRBD02-01*	*TCRBJ02-05*01*
CASRQNTEGLLGEQFF	77	0.054787503	48	*TCRBV05-01*01*	*TCRBD02-01*	*TCRBJ02-01*01*
CASSLGFDRSSYEQYF	77	0.054787503	48	*TCRBV07-06*01*	*TCRBD02-01*02*	*TCRBJ02-07*01*
CASSQGGRAEAFF	77	0.054787503	39	*TCRBV04-01*01*	*TCRBD01-01*01*	*TCRBJ01-01*01*
CASSWAGSLLEQYF	77	0.054787503	42	*TCRBV06*	*TCRBD01-01*01*	*TCRBJ02-07*01*
CSVEPGGETQYF	77	0.054787503	36	*TCRBV29-01*01*	*TCRBD02-01*	*TCRBJ02-05*01*
CAISGQGSHTGELFF	76	0.054075977	45	*TCRBV10-03*01*	*TCRBD01-01*01*	*TCRBJ02-02*01*
CATRQNNEQFF	76	0.054075977	33	*TCRBV02-01*01*	*TCRBD01-01*01*	*TCRBJ02-01*01*

CASQAGVTDTQYF	75	0.053364451	39	*TCRBV07-06*01*	*TCRBD01-01*01*	*TCRBJ02-03*01*
CASSHNTGGNQPQHF	75	0.053364451	45	*TCRBV06-05*01*	*TCRBD01-01*01*	*TCRBJ01-05*01*
CASSPNGGTNEKLFF	75	0.053364451	45	*TCRBV07-03*01*	*unresolved*	*TCRBJ01-04*01*
CASSQAHNYGYTF	75	0.053364451	39	*TCRBV04-01*01*	*TCRBD01-01*01*	*TCRBJ01-02*01*
CASILTGGLETQYF	74	0.052652925	42	*TCRBV07-09*	*TCRBD02-01*01*	*TCRBJ02-05*01*
CASSQGGATDTQYF	74	0.052652925	42	*TCRBV03*	*unresolved*	*TCRBJ02-03*01*
CSASKGMETQYF	74	0.052652925	36	*TCRBV20*	*TCRBD01-01*01*	*TCRBJ02-05*01*
CAAEGLESYEQYF	73	0.051941399	39	*TCRBV06*	*unresolved*	*TCRBJ02-07*01*
CASSLLGQVSGQGALMSYEQYF	73	0.051941399	66	*TCRBV12*	*TCRBD01-01*01*	*TCRBJ02-07*01*
CASSPTGDSGYEQYF	73	0.051941399	45	*TCRBV04-01*01*	*TCRBD01-01*01*	*TCRBJ02-07*01*
CASSQGGRSNTEAFF	73	0.051941399	45	*TCRBV03*	*unresolved*	*TCRBJ01-01*01*
CASSTGQVGEQFF	73	0.051941399	39	*TCRBV07-02*01*	*TCRBD01-01*01*	*TCRBJ02-01*01*
CASTQRGGTPSTEAFF	73	0.051941399	48	*TCRBV03*	*TCRBD02-01*01*	*TCRBJ01-01*01*
CASSLPGQGLNEQFF	72	0.051229873	45	*TCRBV27-01*01*	*TCRBD01-01*01*	*TCRBJ02-01*01*
CASSVGPNTEAFF	72	0.051229873	39	*TCRBV09-01*	*unresolved*	*TCRBJ01-01*01*
CASSYDSMLTEAFF	72	0.051229873	42	*TCRBV06-05*01*	*TCRBD01-01*01*	*TCRBJ01-01*01*
CASSYGAEAFF	72	0.051229873	33	*TCRBV06-05*01*	*TCRBD01-01*01*	*TCRBJ01-01*01*
CASSYPPQPQHF	72	0.051229873	36	*TCRBV06*	*unresolved*	*TCRBJ01-05*01*
CSVEVRVDNEQFF	72	0.051229873	39	*TCRBV29-01*01*	*TCRBD01-01*01*	*TCRBJ02-01*01*
CASGQPYSNQPQHF	71	0.050518347	42	*TCRBV27-01*01*	*TCRBD01-01*01*	*TCRBJ01-05*01*
CASNRGQVEKLFF	71	0.050518347	39	*TCRBV28-01*01*	*unresolved*	*TCRBJ01-04*01*
CASSGLVETQYF	71	0.050518347	36	*TCRBV28-01*01*	*TCRBD02-01*	*TCRBJ02-05*01*
CASSLAPGQGGPETQYF	71	0.050518347	51	*TCRBV05-01*01*	*TCRBD01-01*01*	*TCRBJ02-05*01*
CASSLVSGEAKQFF	71	0.050518347	42	*TCRBV05-01*01*	*TCRBD02-01*01*	*TCRBJ02-01*01*
CASSPSRTGGRGTEAFF	71	0.050518347	51	*TCRBV05-01*01*	*TCRBD01-01*01*	*TCRBJ01-01*01*
CASSSGGPGEQYF	71	0.050518347	39	*TCRBV09-01*	*TCRBD02-01*01*	*TCRBJ02-07*01*
CASSVALAGGYEQYF	71	0.050518347	45	*TCRBV05-01*01*	*TCRBD02-01*01*	*TCRBJ02-07*01*
CASSVTLEVSGGYTF	71	0.050518347	45	*TCRBV06-01*01*	*unresolved*	*TCRBJ01-02*01*
CSADRTSGGNEQFF	71	0.050518347	42	*TCRBV20*	*TCRBD02-01*01*	*TCRBJ02-01*01*
CSASGSAGELFF	71	0.050518347	36	*TCRBV20*	*TCRBD02-01*	*TCRBJ02-02*01*
CASRGTGGTYEQYF	70	0.049806821	42	*TCRBV11-02*02*	*TCRBD01-01*01*	*TCRBJ02-07*01*
CASSHLAGPYNEQFF	70	0.049806821	45	*TCRBV11-01*01*	*TCRBD02-01*01*	*TCRBJ02-01*01*

CASSKTGDEQFF	70	0.049806821	36	*TCRBV07-03*01*	*TCRBD02-01*02*	*TCRBJ02-01*01*
CASSLTGGLGRMTSTDTQYF	70	0.049806821	60	*TCRBV19-01*	*TCRBD02-01*	*TCRBJ02-03*01*
CAALYNYEQYF	69	0.049095295	33	*TCRBV06-01*01*	*unresolved*	*TCRBJ02-07*01*
CASSFYRQGWQETQYF	69	0.049095295	48	*TCRBV27-01*01*	*TCRBD01-01*01*	*TCRBJ02-05*01*
CASSQGASYEQYF	69	0.049095295	39	*TCRBV03*	*TCRBD01-01*01*	*TCRBJ02-07*01*
CASSSDWSSYNEQFF	69	0.049095295	45	*TCRBV02-01*01*	*TCRBD02-01*02*	*TCRBJ02-01*01*
CASSWGGTEAFF	69	0.049095295	36	*TCRBV19-01*	*TCRBD02-01*01*	*TCRBJ01-01*01*
CASSWQDSRSVQGRYF	69	0.049095295	48	*TCRBV06*	*TCRBD01-01*01*	*TCRBJ02-07*01*
CASSPPGIFSYNEQFF	68	0.048383769	48	*TCRBV18-01*01*	*unresolved*	*TCRBJ02-01*01*
CASSRDDTQYF	68	0.048383769	33	*TCRBV07-09*	*TCRBD02-01*02*	*TCRBJ02-03*01*
CASSSGRASGQYF	68	0.048383769	39	*TCRBV12*	*TCRBD02-01*02*	*TCRBJ02-07*01*
CASTSRGQPNNEQFF	68	0.048383769	45	*TCRBV19-01*	*TCRBD01-01*01*	*TCRBJ02-01*01*
CAIGGTGIGYTF	67	0.047672243	36	*TCRBV07-06*01*	*TCRBD02-01*02*	*TCRBJ01-02*01*
CASSLGPSGRNEQFF	67	0.047672243	45	*TCRBV07-02*01*	*unresolved*	*TCRBJ02-01*01*
CASSQSINARGKNIQYF	67	0.047672243	51	*TCRBV23-01*01*	*TCRBD01-01*01*	*TCRBJ02-04*01*
CASSQSWGGETQYF	67	0.047672243	42	*TCRBV09-01*	*TCRBD02-01*01*	*TCRBJ02-05*01*
CASSSARNLANYGYTF	67	0.047672243	48	*TCRBV13-01*01*	*TCRBD02-01*	*TCRBJ01-02*01*
CASKPRGRIEKLFF	66	0.046960717	42	*TCRBV02-01*01*	*unresolved*	*TCRBJ01-04*01*
CASSEWGTKDNEQFF	66	0.046960717	45	*TCRBV10-02*01*	*TCRBD02-01*	*TCRBJ02-01*01*
CASSRTRTGAYEQYF	66	0.046960717	45	*TCRBV11-01*01*	*TCRBD02-01*01*	*TCRBJ02-07*01*
CASSYSWWDEQYF	66	0.046960717	39	*TCRBV05-05*01*	*unresolved*	*TCRBJ02-07*01*
CASSYTGTGGWKQYF	66	0.046960717	45	*TCRBV05-06*01*	*TCRBD01-01*01*	*TCRBJ02-07*01*
CASTVTSGPPNTGELFF	66	0.046960717	51	*TCRBV05-01*01*	*TCRBD02-01*	*TCRBJ02-02*01*
CAWSGQHNEQFF	66	0.046960717	36	*TCRBV30-01*01*	*TCRBD01-01*01*	*TCRBJ02-01*01*
CSAEGPGGHYEQYF	66	0.046960717	42	*TCRBV20*	*TCRBD02-01*01*	*TCRBJ02-07*01*
CASQGLPMNTEAFF	65	0.046249191	42	*TCRBV10-02*01*	*TCRBD02-01*	*TCRBJ01-01*01*
CASSPGQRSGNEQFF	65	0.046249191	45	*TCRBV07-08*01*	*TCRBD01-01*01*	*TCRBJ02-01*01*
CASSSTQGAYNQPQHF	65	0.046249191	48	*TCRBV06*	*TCRBD01-01*01*	*TCRBJ01-05*01*
CSATGAGARETQYF	65	0.046249191	42	*TCRBV20*	*TCRBD02-01*01*	*TCRBJ02-05*01*
CASSFSPFDEQYF	64	0.045537665	39	*TCRBV12*	*unresolved*	*TCRBJ02-07*01*
CASSFVRGRAGELFF	64	0.045537665	45	*TCRBV12*	*TCRBD01-01*01*	*TCRBJ02-02*01*
CASSLGVNYEQYF	64	0.045537665	39	*TCRBV27-01*01*	*unresolved*	*TCRBJ02-07*01*

CSARVPYPGQGLDQPQHF	64	0.045537665	54	*TCRBV20*	*TCRBD01-01*01*	*TCRBJ01-05*01*
CASAPGLTYEQYF	63	0.044826139	39	*TCRBV12*	*TCRBD02-01*	*TCRBJ02-07*01*
CASSDRGNQPQHF	63	0.044826139	39	*TCRBV19-01*	*TCRBD01-01*01*	*TCRBJ01-05*01*
CASSGTGGVFYGYTF	63	0.044826139	45	*TCRBV12*	*TCRBD01-01*01*	*TCRBJ01-02*01*
CASRQGLYEKLFF	62	0.044114613	39	*TCRBV07-08*01*	*TCRBD01-01*01*	*TCRBJ01-04*01*
CASSPRASGGPDNTEAFF	62	0.044114613	54	*TCRBV18-01*01*	*TCRBD01-01*01*	*TCRBJ01-01*01*
CATSRGFEGPYNSPLHF	62	0.044114613	51	*TCRBV15-01*01*	*unresolved*	*TCRBJ01-06*01*
CSARDNGVWEKLFF	62	0.044114613	42	*TCRBV20*	*unresolved*	*TCRBJ01-04*01*
CSARDRVGQGYHEQFF	62	0.044114613	48	*TCRBV20*	*TCRBD01-01*01*	*TCRBJ02-01*01*
CSARGNYEQYF	62	0.044114613	33	*TCRBV20-01*01*	*unresolved*	*TCRBJ02-07*01*
CASEGQEYYGYTF	61	0.043403087	39	*TCRBV11-02*02*	*TCRBD01-01*01*	*TCRBJ01-02*01*
CASSEGQGPYNEQFF	61	0.043403087	45	*TCRBV06-01*01*	*TCRBD01-01*01*	*TCRBJ02-01*01*
CASSLQGARTDTQYF	61	0.043403087	45	*TCRBV06-06*	*TCRBD01-01*01*	*TCRBJ02-03*01*
CASSNQGDTGELFF	61	0.043403087	42	*TCRBV25-01*01*	*TCRBD01-01*01*	*TCRBJ02-02*01*
CRGTSGGASTDTQYF	61	0.043403087	45	*TCRBV20*	*TCRBD02-01*01*	*TCRBJ02-03*01*
CSARTINTGELFF	61	0.043403087	39	*TCRBV20*	*unresolved*	*TCRBJ02-02*01*
CSVEGQGASETQYF	61	0.043403087	42	*TCRBV29-01*01*	*TCRBD01-01*01*	*TCRBJ02-05*01*
CASSIARGVAGAPQHF	60	0.042691561	48	*TCRBV19-01*	*unresolved*	*TCRBJ01-05*01*
CASSIPGSAGGWDEQFF	60	0.042691561	51	*TCRBV19-01*	*TCRBD02-01*02*	*TCRBJ02-01*01*
CASSLSFSPSGYGYTF	60	0.042691561	48	*TCRBV07-03*01*	*TCRBD02-01*	*TCRBJ01-02*01*
CASSLVRSYEQYF	60	0.042691561	39	*TCRBV11-02*02*	*unresolved*	*TCRBJ02-07*01*
CASSPPGRYGYTF	60	0.042691561	39	*TCRBV04-01*01*	*TCRBD02-01*02*	*TCRBJ01-02*01*
CASSPPTADTQYF	60	0.042691561	39	*TCRBV28-01*01*	*unresolved*	*TCRBJ02-03*01*
CASSQADSLYEQYF	60	0.042691561	42	*TCRBV23-01*01*	*TCRBD01-01*01*	*TCRBJ02-07*01*
CSASYPRGGQPIESQHF	60	0.042691561	51	*TCRBV20*	*TCRBD01-01*01*	*TCRBJ01-05*01*
CASSALTGQSEQYF	59	0.041980035	42	*TCRBV02-01*01*	*TCRBD01-01*01*	*TCRBJ02-07*01*
CASSEGGYNYGYTF	59	0.041980035	42	*TCRBV27-01*01*	*unresolved*	*TCRBJ01-02*01*
CASSFYRTSADTQYF	59	0.041980035	45	*TCRBV27-01*01*	*TCRBD02-01*	*TCRBJ02-03*01*
CASSIAAYNEQFF	59	0.041980035	39	*TCRBV07-09*	*unresolved*	*TCRBJ02-01*01*
CASSPGHLSYGYTF	59	0.041980035	42	*TCRBV02-01*01*	*TCRBD01-01*01*	*TCRBJ01-02*01*
CASSSAADGETQYF	59	0.041980035	42	*TCRBV05-04*01*	*unresolved*	*TCRBJ02-05*01*
CASTRDLQAYEQYF	59	0.041980035	42	*TCRBV28-01*01*	*unresolved*	*TCRBJ02-07*01*

CATAFGGSEQYF	59	0.041980035	36	*TCRBV19-01*	*unresolved*	*TCRBJ02-07*01*
CATTQGGLQPQHF	59	0.041980035	39	*TCRBV19-01*	*TCRBD01-01*01*	*TCRBJ01-05*01*
CSAGGQAVQPQHF	59	0.041980035	39	*TCRBV20*	*TCRBD01-01*01*	*TCRBJ01-05*01*
CASSFPGTDQYF	58	0.041268509	36	*TCRBV07-02*01*	*unresolved*	*TCRBJ02-05*01*
CASSHRASGANVLTF	58	0.041268509	45	*TCRBV07-02*01*	*TCRBD01-01*01*	*TCRBJ02-06*01*
CASSLEGRYEQYF	58	0.041268509	39	*TCRBV05-01*01*	*unresolved*	*TCRBJ02-07*01*
CASTLLGTINEQFF	58	0.041268509	42	*TCRBV12*	*TCRBD02-01*	*TCRBJ02-01*01*
CAYRGEEGAGANVLTF	58	0.041268509	48	*TCRBV10-03*01*	*unresolved*	*TCRBJ02-06*01*
CASSDRIRYEQYF	57	0.040556983	39	*TCRBV02-01*01*	*unresolved*	*TCRBJ02-07*01*
CASSLLLPSGANVLTF	57	0.040556983	48	*TCRBV27-01*01*	*TCRBD02-01*	*TCRBJ02-06*01*
CASSLTATHEQFF	57	0.040556983	39	*TCRBV13-01*01*	*unresolved*	*TCRBJ02-01*01*
CASSLTTISSYEQYF	57	0.040556983	45	*TCRBV07-02*01*	*TCRBD02-01*	*TCRBJ02-07*01*
CASSQMTAANTEAFF	57	0.040556983	45	*TCRBV09-01*	*TCRBD02-01*	*TCRBJ01-01*01*
CASSRGEANSPLHF	57	0.040556983	42	*TCRBV06-04*	*TCRBD02-01*	*TCRBJ01-06*01*
CASYSSSYNEQFF	57	0.040556983	39	*TCRBV27-01*01*	*TCRBD02-01*	*TCRBJ02-01*01*
CASSLSGTGFTDTQYF	56	0.039845457	48	*TCRBV05-06*01*	*TCRBD01-01*01*	*TCRBJ02-03*01*
CATSRAGLAGEGEQFF	56	0.039845457	48	*TCRBV15-01*01*	*TCRBD02-01*02*	*TCRBJ02-01*01*
CAWSLAEPRTDTQYF	56	0.039845457	45	*TCRBV30-01*01*	*TCRBD02-01*	*TCRBJ02-03*01*
CAINQRESQTSYEQYF	55	0.039133931	48	*TCRBV10-03*01*	*TCRBD02-01*02*	*TCRBJ02-07*01*
CASRPRSTGTGNTIYF	55	0.039133931	48	*TCRBV05-05*01*	*TCRBD01-01*01*	*TCRBJ01-03*01*
CASSEPQGSYTF	55	0.039133931	36	*TCRBV02-01*01*	*TCRBD01-01*01*	*TCRBJ01-02*01*
CASSERYGYTF	55	0.039133931	33	*TCRBV02-01*01*	*TCRBD02-01*	*TCRBJ01-02*01*
CASSFFVDGSYEQYF	55	0.039133931	45	*TCRBV27-01*01*	*unresolved*	*TCRBJ02-07*01*
CASSFSGRSDEQYF	55	0.039133931	42	*TCRBV19-01*	*TCRBD02-01*02*	*TCRBJ02-07*01*
CASSLGGYSYQETQYF	55	0.039133931	48	*TCRBV05-06*01*	*unresolved*	*TCRBJ02-05*01*
CASSLSAGGLGYTF	55	0.039133931	42	*TCRBV28-01*01*	*TCRBD01-01*01*	*TCRBJ01-02*01*
CASSQEGFSYEQYF	55	0.039133931	42	*TCRBV04-02*01*	*unresolved*	*TCRBJ02-07*01*
CASSRIGHTAYNEQFF	55	0.039133931	48	*TCRBV04-01*01*	*TCRBD01-01*01*	*TCRBJ02-01*01*
CASSSPNRVSGNTIYF	55	0.039133931	48	*TCRBV07-09*	*TCRBD01-01*01*	*TCRBJ01-03*01*
CASSYSNAPAGGQTGELFF	55	0.039133931	57	*TCRBV06-07*01*	*TCRBD02-01*01*	*TCRBJ02-02*01*
CASSYTAGGSYEQYF	55	0.039133931	45	*TCRBV05-01*01*	*TCRBD02-01*01*	*TCRBJ02-07*01*
CAWSVRRNQPQHF	55	0.039133931	39	*TCRBV30-01*01*	*TCRBD02-01*02*	*TCRBJ01-05*01*

CASRSGALPDNEQFF	54	0.038422405	45	*TCRBV12*	*unresolved*	*TCRBJ02-01*01*
CASSLGAYKNTEAFF	54	0.038422405	45	*TCRBV07-02*01*	*TCRBD01-01*01*	*TCRBJ01-01*01*
CASSLGRGSGRSENEQYF	54	0.038422405	54	*TCRBV07-06*01*	*TCRBD02-01*02*	*TCRBJ02-07*01*
CASSLNAVYGYTF	54	0.038422405	39	*TCRBV28-01*01*	*unresolved*	*TCRBJ01-02*01*
CASSQERQIQLYGYTF	54	0.038422405	48	*TCRBV04-01*01*	*TCRBD01-01*01*	*TCRBJ01-02*01*
CASSTLGRDTQYF	54	0.038422405	39	*TCRBV09-01*	*TCRBD02-01*	*TCRBJ02-03*01*
CASSYSGQNSNQPQHF	54	0.038422405	48	*TCRBV06-06*	*TCRBD01-01*01*	*TCRBJ01-05*01*
CASTSGVQPQHF	54	0.038422405	36	*TCRBV06*	*TCRBD02-01*01*	*TCRBJ01-05*01*
CSARGNLGADTQYF	54	0.038422405	42	*TCRBV20*	*unresolved*	*TCRBJ02-03*01*
CSVEDLDSTEAFF	54	0.038422405	39	*TCRBV29-01*01*	*TCRBD01-01*01*	*TCRBJ01-01*01*
CASRGGLSSGNTIYF	53	0.037710879	45	*TCRBV02-01*01*	*TCRBD02-01*	*TCRBJ01-03*01*
CASSLPRAGTGNNYGYTF	53	0.037710879	54	*TCRBV07-09*	*TCRBD01-01*01*	*TCRBJ01-02*01*
CASSLSAGGGTDTQYF	53	0.037710879	48	*TCRBV07-03*01*	*TCRBD01-01*01*	*TCRBJ02-03*01*
CASSPNRGRPYGYTF	53	0.037710879	45	*TCRBV23-01*01*	*TCRBD01-01*01*	*TCRBJ01-02*01*
CASSVNSATSYNEQFF	53	0.037710879	48	*TCRBV09-01*	*TCRBD02-01*	*TCRBJ02-01*01*
CASSYSGNYYTF	53	0.037710879	36	*TCRBV06-05*01*	*unresolved*	*TCRBJ01-02*01*
CSVEGTPGTQYF	53	0.037710879	36	*TCRBV29-01*01*	*unresolved*	*TCRBJ02-05*01*
CASSLDLSPGWGTGELFF	52	0.036999353	54	*TCRBV11-02*02*	*unresolved*	*TCRBJ02-02*01*
CATSRDRTRRGEQYF	52	0.036999353	45	*TCRBV15-01*01*	*TCRBD02-01*01*	*TCRBJ02-07*01*
CAISIGRFTDTQYF	51	0.036287827	42	*TCRBV10-03*01*	*unresolved*	*TCRBJ02-03*01*
CASSLAGYPGEQYF	51	0.036287827	42	*TCRBV28-01*01*	*TCRBD01-01*01*	*TCRBJ02-07*01*
CASSLEGPSGNTIYF	51	0.036287827	45	*TCRBV05-06*01*	*TCRBD01-01*01*	*TCRBJ01-03*01*
CASSLGRVLDYGYTF	51	0.036287827	45	*TCRBV05-01*01*	*TCRBD01-01*01*	*TCRBJ01-02*01*
CASSLMQGYQSYEQYF	51	0.036287827	48	*TCRBV12*	*TCRBD01-01*01*	*TCRBJ02-07*01*
CASSPGQGSRDEQFF	51	0.036287827	45	*TCRBV05-04*01*	*TCRBD01-01*01*	*TCRBJ02-01*01*
CASSPPADNQPQHF	51	0.036287827	42	*TCRBV28-01*01*	*TCRBD02-01*	*TCRBJ01-05*01*
CASSRLAGGPSYNEQFF	51	0.036287827	51	*TCRBV06*	*TCRBD02-01*01*	*TCRBJ02-01*01*
CASSSQGVRGNPEAFF	51	0.036287827	48	*TCRBV05-01*01*	*TCRBD01-01*01*	*TCRBJ01-01*01*
CASSSTRQVSNTEAFF	51	0.036287827	48	*TCRBV28-01*01*	*TCRBD01-01*01*	*TCRBJ01-01*01*
CASTGRTYEQYF	51	0.036287827	36	*TCRBV06-06*	*unresolved*	*TCRBJ02-07*01*
CASKADPNIQYF	50	0.0355763	36	*TCRBV06-01*01*	*TCRBD02-01*	*TCRBJ02-04*01*
CASSFDSPYNQPQHF	50	0.0355763	45	*TCRBV12*	*TCRBD01-01*01*	*TCRBJ01-05*01*

CASSPWYEQYF	50	0.0355763	33	*TCRBV28-01*01*	*unresolved*	*TCRBJ02-07*01*
CASRGGNQPQHF	49	0.034864774	36	*TCRBV10-02*01*	*TCRBD02-01*02*	*TCRBJ01-05*01*
CASSLGPAGGYEQYF	49	0.034864774	45	*TCRBV27-01*01*	*TCRBD02-01*02*	*TCRBJ02-07*01*
CASSLGQAASTGELFF	49	0.034864774	48	*TCRBV05-01*01*	*TCRBD01-01*01*	*TCRBJ02-02*01*
CASSLSEGSGYTF	49	0.034864774	39	*TCRBV27-01*01*	*TCRBD02-01*02*	*TCRBJ01-02*01*
CASSRLRGRDTQYF	49	0.034864774	42	*TCRBV09-01*	*TCRBD01-01*01*	*TCRBJ02-03*01*
CASSSLGSGGAIEQFF	49	0.034864774	48	*TCRBV07-06*01*	*TCRBD02-01*01*	*TCRBJ02-01*01*
CASSTGGRRSREQSF	49	0.034864774	45	*TCRBV19-01*	*TCRBD01-01*01*	*TCRBJ01-04*01*
CASRDRSIYGYTF	48	0.034153248	39	*TCRBV19-01*	*TCRBD01-01*01*	*TCRBJ01-02*01*
CASSFGPTNEKLFF	48	0.034153248	42	*TCRBV28-01*01*	*unresolved*	*TCRBJ01-04*01*
CASSPGLAGGHEQFF	48	0.034153248	45	*TCRBV12*	*TCRBD02-01*01*	*TCRBJ02-01*01*
CASSPLSRSGYEQYF	48	0.034153248	45	*TCRBV06-01*01*	*unresolved*	*TCRBJ02-07*01*
CASSQEWGLDSEQFF	48	0.034153248	45	*TCRBV03*	*TCRBD01-01*01*	*TCRBJ02-01*01*
CASSEEVFQTF	47	0.033441722	33	*TCRBV02-01*01*	*unresolved*	*TCRBJ01-02*01*
CASSEIGPNEKLFF	47	0.033441722	42	*TCRBV02-01*01*	*unresolved*	*TCRBJ01-04*01*
CASSLGLADYNEQFF	47	0.033441722	45	*TCRBV05-01*01*	*TCRBD02-01*	*TCRBJ02-01*01*
CASSLPTSGGDNEQFF	47	0.033441722	48	*TCRBV13-01*01*	*TCRBD02-01*01*	*TCRBJ02-01*01*
CASSPLPSGSQNTEAFF	47	0.033441722	51	*TCRBV18-01*01*	*TCRBD02-01*	*TCRBJ01-01*01*
CASSPPERGGFNEQFF	47	0.033441722	48	*TCRBV18-01*01*	*TCRBD02-01*01*	*TCRBJ02-01*01*
CASSQAQGLSNQPQHF	47	0.033441722	48	*TCRBV04-01*01*	*TCRBD01-01*01*	*TCRBJ01-05*01*
CASSQVRLNDEQFF	47	0.033441722	42	*TCRBV04-01*01*	*TCRBD02-01*	*TCRBJ02-01*01*
CRGAASNYGYTF	47	0.033441722	36	*TCRBV29-01*01*	*TCRBD01-01*01*	*TCRBJ01-02*01*
CSAQEVLNTEAFF	47	0.033441722	39	*TCRBV20*	*unresolved*	*TCRBJ01-01*01*
CAISQLLQETQYF	46	0.032730196	39	*TCRBV10-03*01*	*unresolved*	*TCRBJ02-05*01*
CASRADSRESEANVLTF	46	0.032730196	51	*TCRBV02-01*01*	*unresolved*	*TCRBJ02-06*01*
CASSFHRKAGGPGDTQYF	46	0.032730196	54	*TCRBV27-01*01*	*TCRBD02-01*01*	*TCRBJ02-03*01*
CASSPLPPTGANTEAFF	46	0.032730196	51	*TCRBV18-01*01*	*TCRBD01-01*01*	*TCRBJ01-01*01*
CASSQDPTSGGTYNEQFF	46	0.032730196	54	*TCRBV03*	*TCRBD02-01*	*TCRBJ02-01*01*
CASSYDLPGTLEGTQYF	46	0.032730196	51	*TCRBV07-02*01*	*TCRBD02-01*	*TCRBJ02-05*01*
CASSYGGKDTQYF	46	0.032730196	39	*TCRBV06-04*	*TCRBD02-01*01*	*TCRBJ02-03*01*
CSAISGVAGPNEQFF	46	0.032730196	45	*TCRBV20*	*TCRBD02-01*	*TCRBJ02-01*01*
CASSLGVGTTNEKLFF	45	0.03201867	48	*TCRBV05-01*01*	*unresolved*	*TCRBJ01-04*01*

CASSLLGRTSTDTQYF	45	0.03201867	48	*TCRBV12*	*unresolved*	*TCRBJ02-03*01*
CASSPPSSSGGELFF	45	0.03201867	45	*TCRBV18-01*01*	*TCRBD02-01*	*TCRBJ02-02*01*
CASSPRFTEAFF	45	0.03201867	36	*TCRBV11-01*01*	*TCRBD02-01*02*	*TCRBJ01-01*01*
CASSQGSATSGRTYEQYF	45	0.03201867	54	*TCRBV04-02*01*	*TCRBD02-01*02*	*TCRBJ02-07*01*
CASSSGTTGTTGELFF	45	0.03201867	48	*TCRBV07-06*01*	*TCRBD01-01*01*	*TCRBJ02-02*01*
CASSVEWTGEQYF	45	0.03201867	39	*TCRBV09-01*	*TCRBD02-01*	*TCRBJ02-07*01*
CASSVGGPYEQYF	45	0.03201867	39	*TCRBV02-01*01*	*unresolved*	*TCRBJ02-07*01*
CASSWGQPDTQYF	45	0.03201867	39	*TCRBV05-01*01*	*TCRBD01-01*01*	*TCRBJ02-03*01*
CATSDEGFYGYTF	45	0.03201867	39	*TCRBV24*	*unresolved*	*TCRBJ01-02*01*
CASLPGPSTGNSPLHF	44	0.031307144	48	*TCRBV06*	*unresolved*	*TCRBJ01-06*01*
CASSGGGLMNTEAFF	44	0.031307144	45	*TCRBV27-01*01*	*TCRBD02-01*01*	*TCRBJ01-01*01*
CASSGGQSSSYEQYF	44	0.031307144	45	*TCRBV05-01*01*	*TCRBD01-01*01*	*TCRBJ02-07*01*
CASSLNRAREQYF	44	0.031307144	39	*TCRBV11-02*02*	*TCRBD01-01*01*	*TCRBJ02-07*01*
CASSSGFANYGYTF	44	0.031307144	42	*TCRBV05-01*01*	*TCRBD01-01*01*	*TCRBJ01-02*01*
CASSSSGASNQPQHF	44	0.031307144	45	*TCRBV27-01*01*	*unresolved*	*TCRBJ01-05*01*
CASSSTGTLQETQYF	44	0.031307144	45	*TCRBV07-03*01*	*TCRBD01-01*01*	*TCRBJ02-05*01*
CASSYPPPGHYGYTF	44	0.031307144	45	*TCRBV06-05*01*	*TCRBD01-01*01*	*TCRBJ01-02*01*
CAIPMSGQSYGYTF	43	0.030595618	42	*TCRBV10-03*01*	*TCRBD01-01*01*	*TCRBJ01-02*01*
CASKNGAGNTIYF	43	0.030595618	39	*TCRBV02-01*01*	*unresolved*	*TCRBJ01-03*01*
CASRTRDRGTDTQYF	43	0.030595618	45	*TCRBV06-06*	*TCRBD01-01*01*	*TCRBJ02-03*01*
CASSGGATYNEQFF	43	0.030595618	42	*TCRBV19-01*	*TCRBD02-01*01*	*TCRBJ02-01*01*
CASSQGLAGLGTDTQYF	43	0.030595618	51	*TCRBV03*	*TCRBD02-01*01*	*TCRBJ02-03*01*
CASSSRGWGRERYNEQFF	43	0.030595618	54	*TCRBV27-01*01*	*unresolved*	*TCRBJ02-01*01*
CASSYSTGYANTGELFF	43	0.030595618	51	*TCRBV06*	*unresolved*	*TCRBJ02-02*01*
CASSYTGSYGYTF	43	0.030595618	39	*TCRBV06-05*01*	*TCRBD01-01*01*	*TCRBJ01-02*01*
CSVAEYEQYF	43	0.030595618	30	*TCRBV29-01*01*	*unresolved*	*TCRBJ02-07*01*
CSVEEDRVEQFF	43	0.030595618	36	*TCRBV29-01*01*	*TCRBD01-01*01*	*TCRBJ02-01*01*
CAISDSGTSNEQFF	42	0.029884092	42	*TCRBV10-03*01*	*TCRBD02-01*	*TCRBJ02-01*01*
CASSDPWGQVGGYTF	42	0.029884092	45	*TCRBV02-01*01*	*TCRBD01-01*01*	*TCRBJ01-02*01*
CASSDRDTGELFF	42	0.029884092	39	*TCRBV06-04*	*unresolved*	*TCRBJ02-02*01*
CASSFQLAGDVYNEQFF	42	0.029884092	51	*TCRBV11-02*02*	*TCRBD02-01*02*	*TCRBJ02-01*01*
CASSLSSLGRTVEKLFF	42	0.029884092	51	*TCRBV07-09*	*unresolved*	*TCRBJ01-04*01*

CASSLSTGRSAYQPQHF	42	0.029884092	51	*TCRBV28-01*01*	*unresolved*	*TCRBJ01-05*01*
CASSPKDEQYF	42	0.029884092	33	*TCRBV06-01*01*	*unresolved*	*TCRBJ02-07*01*
CASSREPLSSYEQYF	42	0.029884092	45	*TCRBV07-09*	*TCRBD02-01*02*	*TCRBJ02-07*01*
CASSSRLAGSSYNEQFF	42	0.029884092	51	*TCRBV12*	*TCRBD02-01*	*TCRBJ02-01*01*
CASSSTPGTSGSHEQFF	42	0.029884092	51	*TCRBV12*	*TCRBD02-01*02*	*TCRBJ02-01*01*
CSAYRGRETQYF	42	0.029884092	36	*TCRBV20*	*TCRBD01-01*01*	*TCRBJ02-05*01*
CSTGQLDPQYF	42	0.029884092	33	*TCRBV29-01*01*	*TCRBD01-01*01*	*TCRBJ02-05*01*
CASSAGHVDTEAFF	41	0.029172566	42	*TCRBV09-01*	*TCRBD01-01*01*	*TCRBJ01-01*01*
CASSIGTASYNEQFF	41	0.029172566	45	*TCRBV28-01*01*	*TCRBD01-01*01*	*TCRBJ02-01*01*
CASSPGLAGRDTQYF	41	0.029172566	45	*TCRBV12*	*TCRBD02-01*02*	*TCRBJ02-03*01*
CASSPNLAGDYNEQFF	41	0.029172566	48	*TCRBV04-01*01*	*TCRBD02-01*01*	*TCRBJ02-01*01*
CASSPPNRGFREQFF	41	0.029172566	45	*TCRBV11-02*02*	*TCRBD01-01*01*	*TCRBJ02-01*01*
CASSPQGRAAEAFF	41	0.029172566	42	*TCRBV19-01*	*TCRBD01-01*01*	*TCRBJ01-01*01*
CASSQDLVRRIEF	41	0.029172566	39	*TCRBV14-01*01*	*TCRBD02-01*	*TCRBJ02-07*01*
CASSQGANGYTF	41	0.029172566	36	*TCRBV27-01*01*	*TCRBD01-01*01*	*TCRBJ01-02*01*
CATSRGGTSKDEQFF	41	0.029172566	45	*TCRBV15-01*01*	*TCRBD02-01*	*TCRBJ02-01*01*
CASIRGGQGAYEQYF	40	0.02846104	45	*TCRBV02-01*01*	*TCRBD01-01*01*	*TCRBJ02-07*01*
CASNSGGTVTDTQYF	40	0.02846104	45	*TCRBV28-01*01*	*TCRBD01-01*01*	*TCRBJ02-03*01*
CASSEGVGNTEAFF	40	0.02846104	42	*TCRBV25-01*01*	*unresolved*	*TCRBJ01-01*01*
CASSPISDGYYGYTF	40	0.02846104	45	*TCRBV04-01*01*	*unresolved*	*TCRBJ01-02*01*
CASSVGREQFF	40	0.02846104	33	*TCRBV09-01*	*TCRBD02-01*02*	*TCRBJ02-01*01*
CASSYIPRGPIPDPQHF	40	0.02846104	51	*TCRBV06*	*unresolved*	*TCRBJ01-05*01*
CSARNRVNEQFF	40	0.02846104	36	*TCRBV20*	*TCRBD01-01*01*	*TCRBJ02-01*01*
CAQGRGSYEQYF	39	0.027749514	36	*TCRBV02-01*01*	*TCRBD02-01*02*	*TCRBJ02-07*01*
CASRTWGRESTEAFF	39	0.027749514	45	*TCRBV27-01*01*	*TCRBD02-01*02*	*TCRBJ01-01*01*
CASSAELYNEQFF	39	0.027749514	39	*TCRBV11-02*02*	*TCRBD02-01*	*TCRBJ02-01*01*
CASSFLLLSDTQYF	39	0.027749514	42	*TCRBV11-02*02*	*unresolved*	*TCRBJ02-03*01*
CASSLTGRGYYGYTF	39	0.027749514	45	*TCRBV07-02*01*	*TCRBD02-01*02*	*TCRBJ01-02*01*
CASSQGSRGLRETQYF	39	0.027749514	48	*TCRBV14-01*01*	*TCRBD01-01*01*	*TCRBJ02-05*01*
CASSSGPGKTQYF	39	0.027749514	39	*TCRBV07-03*01*	*unresolved*	*TCRBJ02-05*01*
CASTGRQGWSANQPQHF	39	0.027749514	51	*TCRBV12*	*TCRBD01-01*01*	*TCRBJ01-05*01*
CATSYDGYEQYF	39	0.027749514	36	*TCRBV15-01*01*	*unresolved*	*TCRBJ02-07*01*

CASATGISSYEQYF	38	0.027037988	42	*TCRBV02-01*01*	*TCRBD01-01*01*	*TCRBJ02-07*01*
CASSLAQEGDNSPLHF	38	0.027037988	48	*TCRBV07-09*	*TCRBD02-01*02*	*TCRBJ01-06*01*
CASSLEMDSPLHF	38	0.027037988	39	*TCRBV07-09*	*unresolved*	*TCRBJ01-06*01*
CASSLGELGIYEQYF	38	0.027037988	45	*TCRBV07-08*01*	*unresolved*	*TCRBJ02-07*01*
CASSLIAGGTTDTQYF	38	0.027037988	48	*TCRBV07-06*01*	*TCRBD02-01*01*	*TCRBJ02-03*01*
CASSQDIGTDTQYF	38	0.027037988	42	*TCRBV04-02*01*	*TCRBD02-01*	*TCRBJ02-03*01*
CSVDTGGLGEAFF	38	0.027037988	39	*TCRBV29-01*01*	*TCRBD01-01*01*	*TCRBJ01-01*01*
CASRKGYEQYF	37	0.026326462	33	*TCRBV12*	*unresolved*	*TCRBJ02-07*01*
CASSPLRGRGETQYF	37	0.026326462	45	*TCRBV03*	*TCRBD01-01*01*	*TCRBJ02-05*01*
CASSRPDRGTNEKLFF	37	0.026326462	48	*TCRBV28-01*01*	*TCRBD01-01*01*	*TCRBJ01-04*01*
CATSRDLLTSGATQYF	37	0.026326462	48	*TCRBV15-01*01*	*TCRBD01-01*01*	*TCRBJ02-03*01*
CAISAGTGAFNQPQHF	36	0.025614936	48	*TCRBV10-03*01*	*TCRBD01-01*01*	*TCRBJ01-05*01*
CAISDTGRLNTEAFF	36	0.025614936	45	*TCRBV10-03*01*	*TCRBD01-01*01*	*TCRBJ01-01*01*
CASSAGRQILYTF	36	0.025614936	39	*TCRBV05-04*01*	*unresolved*	*TCRBJ01-02*01*
CASSAQVSEQFF	36	0.025614936	36	*TCRBV06*	*TCRBD01-01*01*	*TCRBJ02-01*01*
CASSARVSGYTF	36	0.025614936	36	*TCRBV07-08*01*	*TCRBD02-01*	*TCRBJ01-02*01*
CASSELGGRNTEAFF	36	0.025614936	45	*TCRBV02-01*01*	*unresolved*	*TCRBJ01-01*01*
CASSPPSRGANYEQYF	36	0.025614936	48	*TCRBV07-09*	*TCRBD02-01*01*	*TCRBJ02-07*01*
CASSRIGRTSTDTQYF	36	0.025614936	48	*TCRBV28-01*01*	*unresolved*	*TCRBJ02-03*01*
CATSGYVRSGNTIYF	36	0.025614936	45	*TCRBV24*	*unresolved*	*TCRBJ01-03*01*
CATSRGVSGGASDTQYF	36	0.025614936	51	*TCRBV15-01*01*	*TCRBD02-01*01*	*TCRBJ02-03*01*
CAWSVTHRYEQYF	36	0.025614936	39	*TCRBV30-01*01*	*TCRBD01-01*01*	*TCRBJ02-07*01*
CSAGPSPGLETQYF	36	0.025614936	42	*TCRBV20*	*TCRBD01-01*01*	*TCRBJ02-05*01*
CSASRQGAEAFF	36	0.025614936	36	*TCRBV20*	*TCRBD01-01*01*	*TCRBJ01-01*01*
CAGSEYSNQPQHF	35	0.02490341	39	*TCRBV28-01*01*	*unresolved*	*TCRBJ01-05*01*
CASSASSQGLIQETQYF	35	0.02490341	51	*TCRBV07-09*	*TCRBD01-01*01*	*TCRBJ02-05*01*
CASSLGGGRQTQYF	35	0.02490341	42	*TCRBV07-03*01*	*TCRBD02-01*01*	*TCRBJ02-05*01*
CASSPRDRGLGYTF	35	0.02490341	42	*TCRBV28-01*01*	*TCRBD01-01*01*	*TCRBJ01-02*01*
CASSTYGTANYGYTF	35	0.02490341	45	*TCRBV05-01*01*	*TCRBD01-01*01*	*TCRBJ01-02*01*
CASSVGGMGYNEQFF	35	0.02490341	45	*TCRBV09-01*	*unresolved*	*TCRBJ02-01*01*
CASSVNTGGSGYTF	35	0.02490341	42	*TCRBV09-01*	*TCRBD01-01*01*	*TCRBJ01-02*01*
CASSVWGSGGSTDTQYF	35	0.02490341	51	*TCRBV09-01*	*TCRBD02-01*01*	*TCRBJ02-03*01*

CASSYPGPEAFF	35	0.02490341	36	*TCRBV06-05*01*	*TCRBD01-01*01*	*TCRBJ01-01*01*
CATLGAGEETQYF	35	0.02490341	39	*TCRBV30-01*01*	*TCRBD02-01*02*	*TCRBJ02-05*01*
CSARADRLIGQAFF	35	0.02490341	42	*TCRBV20*	*TCRBD01-01*01*	*TCRBJ01-01*01*
CSASGDSTDTQYF	35	0.02490341	39	*TCRBV20*	*unresolved*	*TCRBJ02-03*01*
CASSLLSRLSTDTQYF	34	0.024191884	48	*TCRBV28-01*01*	*TCRBD02-01*	*TCRBJ02-03*01*
CASSSRQSGANVLTF	34	0.024191884	45	*TCRBV18-01*01*	*TCRBD01-01*01*	*TCRBJ02-06*01*
CASSSTRDGYTF	34	0.024191884	36	*TCRBV05-01*01*	*unresolved*	*TCRBJ01-02*01*
CASSSVLDSYEQYF	34	0.024191884	42	*TCRBV05-01*01*	*TCRBD02-01*	*TCRBJ02-07*01*
CASSTGTGGSDTQYF	34	0.024191884	45	*TCRBV05-05*01*	*TCRBD01-01*01*	*TCRBJ02-03*01*
CASTFLRTPNTGELFF	34	0.024191884	48	*TCRBV02-01*01*	*unresolved*	*TCRBJ02-02*01*
CASWHRDYGNTEAFF	34	0.024191884	45	*TCRBV27-01*01*	*TCRBD02-01*	*TCRBJ01-01*01*
CARGETQYF	33	0.023480358	27	*TCRBV25-01*01*	*unresolved*	*TCRBJ02-05*01*
CASSEDWVRELFF	33	0.023480358	39	*TCRBV02-01*01*	*unresolved*	*TCRBJ02-02*01*
CASSFPGGGETQYF	33	0.023480358	42	*TCRBV05-01*01*	*unresolved*	*TCRBJ02-05*01*
CASSHRGLAGQETQYF	33	0.023480358	48	*TCRBV04-02*01*	*TCRBD02-01*	*TCRBJ02-05*01*
CASSLDGPSGSGEQFF	33	0.023480358	48	*TCRBV11-03*01*	*TCRBD02-01*02*	*TCRBJ02-01*01*
CASSLMVAYEQYF	33	0.023480358	39	*TCRBV07-08*01*	*TCRBD02-01*	*TCRBJ02-07*01*
CASSLPERAETQYF	33	0.023480358	42	*TCRBV28-01*01*	*TCRBD02-01*	*TCRBJ02-05*01*
CASSPLGVLNEKLFF	33	0.023480358	45	*TCRBV02-01*01*	*TCRBD01-01*01*	*TCRBJ01-04*01*
CASSQGTSGRVVYEQYF	33	0.023480358	51	*TCRBV03*	*TCRBD02-01*02*	*TCRBJ02-07*01*
CASSVAGDRTDTQYF	33	0.023480358	45	*TCRBV09-01*	*TCRBD02-01*02*	*TCRBJ02-03*01*
CASSVSPWVRDTQYF	33	0.023480358	45	*TCRBV09-01*	*unresolved*	*TCRBJ02-03*01*
CAWSVLGETQYF	33	0.023480358	36	*TCRBV30-01*01*	*unresolved*	*TCRBJ02-05*01*
CAWSVSGELFF	33	0.023480358	33	*TCRBV30-01*01*	*unresolved*	*TCRBJ02-02*01*
CSARLAGGQETQYF	33	0.023480358	42	*TCRBV20*	*TCRBD02-01*01*	*TCRBJ02-05*01*
CSVVVTGELFF	33	0.023480358	33	*TCRBV29-01*01*	*TCRBD02-01*	*TCRBJ02-02*01*
CASRRGTSGALNTGELFF	32	0.022768832	54	*TCRBV28-01*01*	*TCRBD02-01*01*	*TCRBJ02-02*01*
CASSEASGFTDTQYF	32	0.022768832	45	*TCRBV06-04*	*TCRBD02-01*	*TCRBJ02-03*01*
CASSESAGTSSGNTIYF	32	0.022768832	51	*TCRBV10-02*01*	*unresolved*	*TCRBJ01-03*01*
CASSFDGGADEKLFF	32	0.022768832	45	*TCRBV28-01*01*	*unresolved*	*TCRBJ01-04*01*
CASSLGGTRTRGSYEQYF	32	0.022768832	54	*TCRBV05-01*01*	*unresolved*	*TCRBJ02-07*01*
CASSLIKINRTALAKNIQYF	32	0.022768832	60	*TCRBV05*	*unresolved*	*TCRBJ02-04*01*

CASSPWDREETQYF	32	0.022768832	42	*TCRBV09-01*	*TCRBD01-01*01*	*TCRBJ02-05*01*
CASSQDGGSTQYF	32	0.022768832	39	*TCRBV03*	*TCRBD02-01*02*	*TCRBJ02-03*01*
CASSSIFLGTDTQYF	32	0.022768832	45	*TCRBV02-01*01*	*TCRBD02-01*	*TCRBJ02-03*01*
CASSSRTVPYEQYF	32	0.022768832	42	*TCRBV10-01*	*unresolved*	*TCRBJ02-07*01*
CASSVTTGAGYTF	32	0.022768832	39	*TCRBV07-03*01*	*TCRBD01-01*01*	*TCRBJ01-02*01*
CASSVVPQGDYGYTF	32	0.022768832	45	*TCRBV09-01*	*TCRBD01-01*01*	*TCRBJ01-02*01*
CASSVYRSTDTQYF	32	0.022768832	42	*TCRBV05-01*01*	*unresolved*	*TCRBJ02-03*01*
CAWGDSPNTEAFF	32	0.022768832	39	*TCRBV30-01*01*	*TCRBD01-01*01*	*TCRBJ01-01*01*
CSAQRGAYNEQFF	32	0.022768832	39	*TCRBV29-01*01*	*TCRBD02-01*01*	*TCRBJ02-01*01*
CSATGQYNEQFF	32	0.022768832	36	*TCRBV20*	*TCRBD01-01*01*	*TCRBJ02-01*01*
CASRVETGKGETQYF	31	0.022057306	45	*TCRBV06-05*01*	*TCRBD01-01*01*	*TCRBJ02-05*01*
CASSARGDHPLHNEQFF	31	0.022057306	51	*TCRBV27-01*01*	*TCRBD02-01*	*TCRBJ02-01*01*
CASSERSGDTYEQYF	31	0.022057306	45	*TCRBV02-01*01*	*TCRBD01-01*01*	*TCRBJ02-07*01*
CASSPTASTDTQYF	31	0.022057306	42	*TCRBV05-04*01*	*TCRBD01-01*01*	*TCRBJ02-03*01*
CASSPWTGTGASSYNEQFF	31	0.022057306	57	*TCRBV14-01*01*	*TCRBD01-01*01*	*TCRBJ02-01*01*
CASSVADTYEQYF	31	0.022057306	39	*TCRBV10-03*01*	*unresolved*	*TCRBJ02-07*01*
CASSVGLAGRNEQFF	31	0.022057306	45	*TCRBV07-08*01*	*TCRBD02-01*02*	*TCRBJ02-01*01*
CASSYFLGQPNTGELFF	31	0.022057306	51	*TCRBV06*	*TCRBD01-01*01*	*TCRBJ02-02*01*
CASTLSLRNSPLHF	31	0.022057306	42	*TCRBV28-01*01*	*TCRBD02-01*02*	*TCRBJ01-06*01*
CSVEGQGVADTQYF	31	0.022057306	42	*TCRBV29-01*01*	*TCRBD01-01*01*	*TCRBJ02-03*01*
CASIPQAGINTEAFF	30	0.02134578	45	*TCRBV12*	*TCRBD01-01*01*	*TCRBJ01-01*01*
CASKEGGPSSSYEQYF	30	0.02134578	48	*TCRBV28-01*01*	*unresolved*	*TCRBJ02-07*01*
CASRRLAGNQETQYF	30	0.02134578	45	*TCRBV05-01*01*	*TCRBD02-01*01*	*TCRBJ02-05*01*
CASRTPGRMNTEAFF	30	0.02134578	45	*TCRBV27-01*01*	*TCRBD01-01*01*	*TCRBJ01-01*01*
CASSEARGSGGGETQYF	30	0.02134578	51	*TCRBV06-01*01*	*TCRBD02-01*01*	*TCRBJ02-05*01*
CASSLLAGYNEQFF	30	0.02134578	42	*TCRBV27-01*01*	*TCRBD02-01*	*TCRBJ02-01*01*
CASSMGPFSSYEQYF	30	0.02134578	45	*TCRBV27-01*01*	*unresolved*	*TCRBJ02-07*01*
CASSPYLPGNTEAFF	30	0.02134578	45	*TCRBV18-01*01*	*TCRBD02-01*	*TCRBJ01-01*01*
CASSQAELNTGELFF	30	0.02134578	45	*TCRBV09-01*	*TCRBD02-01*	*TCRBJ02-02*01*
CASSYGTSSYEQYF	30	0.02134578	42	*TCRBV06*	*TCRBD02-01*	*TCRBJ02-07*01*
CASTLTGAIYEQYF	30	0.02134578	42	*TCRBV02-01*01*	*TCRBD01-01*01*	*TCRBJ02-07*01*
CSVAPGLAGAQETQYF	30	0.02134578	48	*TCRBV29-01*01*	*TCRBD02-01*02*	*TCRBJ02-05*01*

CSVRGGDSYEQYF	30	0.02134578	39	*TCRBV29-01*01*	*TCRBD02-01*01*	*TCRBJ02-07*01*
CAISEAGGRDTQYF	29	0.020634254	42	*TCRBV10-03*01*	*TCRBD02-01*01*	*TCRBJ02-03*01*
CAISGTGKPGSGNEQFF	29	0.020634254	51	*TCRBV10-03*01*	*TCRBD01-01*01*	*TCRBJ02-01*01*
CASIVRDRENTQYF	29	0.020634254	42	*TCRBV02-01*01*	*TCRBD01-01*01*	*TCRBJ02-03*01*
CASSALGTGENSPLHF	29	0.020634254	48	*TCRBV07-08*01*	*TCRBD01-01*01*	*TCRBJ01-06*01*
CASSEGGGATDTQYF	29	0.020634254	45	*TCRBV02-01*01*	*TCRBD02-01*01*	*TCRBJ02-03*01*
CASSFFSGYTF	29	0.020634254	33	*TCRBV28-01*01*	*TCRBD02-01*	*TCRBJ01-02*01*
CASSLRRGTIQPQHF	29	0.020634254	45	*TCRBV05-06*01*	*unresolved*	*TCRBJ01-05*01*
CASSPVIRSTNEKLFF	29	0.020634254	48	*TCRBV11-02*02*	*unresolved*	*TCRBJ01-04*01*
CSAQGLAGGHVDEQFF	29	0.020634254	48	*TCRBV20*	*TCRBD02-01*01*	*TCRBJ02-01*01*
CSVEDPGAEAFF	29	0.020634254	36	*TCRBV29-01*01*	*TCRBD01-01*01*	*TCRBJ01-01*01*
CASSESGLASSSTDTQYF	28	0.019922728	54	*TCRBV25-01*01*	*TCRBD02-01*	*TCRBJ02-03*01*
CASSLALGGNTEAFF	28	0.019922728	45	*TCRBV12*	*TCRBD02-01*01*	*TCRBJ01-01*01*
CASSLTGWKRDTEAFF	28	0.019922728	48	*TCRBV05-04*01*	*TCRBD01-01*01*	*TCRBJ01-01*01*
CASSPLPDRTDNEQFF	28	0.019922728	48	*TCRBV18-01*01*	*TCRBD01-01*01*	*TCRBJ02-01*01*
CASSPSGAPNYGYTF	28	0.019922728	45	*TCRBV18-01*01*	*TCRBD01-01*01*	*TCRBJ01-02*01*
CASSQGGLAGYEQYF	28	0.019922728	45	*TCRBV03*	*TCRBD02-01*	*TCRBJ02-07*01*
CASSQGVAANTGELFF	28	0.019922728	48	*TCRBV03*	*TCRBD02-01*	*TCRBJ02-02*01*
CASSRRGGQGPWNGYTF	28	0.019922728	51	*TCRBV03*	*TCRBD01-01*01*	*TCRBJ01-02*01*
CASSEAGGPYEQYF	27	0.019211202	42	*TCRBV02-01*01*	*TCRBD01-01*01*	*TCRBJ02-07*01*
CASSGGVGGGNQPQHF	27	0.019211202	48	*TCRBV02-01*01*	*TCRBD02-01*01*	*TCRBJ01-05*01*
CASSGLAEGTQYF	27	0.019211202	39	*TCRBV06-01*01*	*TCRBD02-01*	*TCRBJ02-03*01*
CASSIVGNEAQPQHF	27	0.019211202	45	*TCRBV19-01*	*unresolved*	*TCRBJ01-05*01*
CASSVFRSSPDRRNQPQHF	27	0.019211202	57	*TCRBV09-01*	*TCRBD01-01*01*	*TCRBJ01-05*01*
CATSDWGTGTQYF	27	0.019211202	39	*TCRBV24*	*TCRBD01-01*01*	*TCRBJ02-03*01*
CAISARAQETQYF	26	0.018499676	39	*TCRBV10-03*01*	*TCRBD01-01*01*	*TCRBJ02-05*01*
CASRAGQGPPEAFF	26	0.018499676	42	*TCRBV02-01*01*	*TCRBD01-01*01*	*TCRBJ01-01*01*
CASRHDRSQSPLHF	26	0.018499676	42	*TCRBV05-01*01*	*TCRBD01-01*01*	*TCRBJ01-06*01*
CASSFFLGQELFF	26	0.018499676	39	*TCRBV07-02*01*	*TCRBD01-01*01*	*TCRBJ02-02*01*
CASSHPTDSGGVNTEAFF	26	0.018499676	54	*TCRBV28-01*01*	*unresolved*	*TCRBJ01-01*01*
CASSKGLAGAQETQYF	26	0.018499676	48	*TCRBV06*	*TCRBD02-01*01*	*TCRBJ02-05*01*
CASSLDGTSGPQETQYF	26	0.018499676	51	*TCRBV05-01*01*	*TCRBD02-01*	*TCRBJ02-05*01*

CASSSRTDYNEQFF	26	0.018499676	42	*TCRBV11-02*02*	*TCRBD01-01*01*	*TCRBJ02-01*01*
CASVKDRYNSPLHF	26	0.018499676	42	*TCRBV02-01*01*	*TCRBD02-01*	*TCRBJ01-06*01*
CSAPPHGRTYNEQFF	26	0.018499676	45	*TCRBV20*	*unresolved*	*TCRBJ02-01*01*
CAPDPDRDLADTQYF	25	0.01778815	45	*TCRBV10-01*	*TCRBD01-01*01*	*TCRBJ02-03*01*
CASSGHGGAGADTQYF	25	0.01778815	48	*TCRBV10-02*01*	*TCRBD02-01*01*	*TCRBJ02-03*01*
CASSLGAGLHTDTQYF	25	0.01778815	48	*TCRBV07-02*01*	*TCRBD02-01*01*	*TCRBJ02-03*01*
CASSLRQGGGEQYF	25	0.01778815	42	*TCRBV05-05*01*	*unresolved*	*TCRBJ02-07*01*
CASSSESYNEQFF	25	0.01778815	39	*TCRBV02-01*01*	*TCRBD02-01*	*TCRBJ02-01*01*
CASSYGTGDQPQHF	25	0.01778815	42	*TCRBV05-05*01*	*TCRBD01-01*01*	*TCRBJ01-05*01*
CSAMGAHSNQPQHF	25	0.01778815	42	*TCRBV20*	*TCRBD01-01*01*	*TCRBJ01-05*01*
CASGAYPTEAFF	24	0.017076624	36	*TCRBV07-08*01*	*TCRBD01-01*01*	*TCRBJ01-01*01*
CASSDGSGSSGNTIYF	24	0.017076624	48	*TCRBV02-01*01*	*unresolved*	*TCRBJ01-03*01*
CASSDSGRGADTQYF	24	0.017076624	45	*TCRBV02-01*01*	*TCRBD02-01*02*	*TCRBJ02-03*01*
CASSLVSGSLDTDTQYF	24	0.017076624	51	*TCRBV28-01*01*	*TCRBD02-01*02*	*TCRBJ02-03*01*
CASSSSRGQGETQYF	24	0.017076624	45	*TCRBV12*	*TCRBD02-01*01*	*TCRBJ02-05*01*
CAISESLTGPSGANVLTF	23	0.016365098	54	*TCRBV10-03*01*	*TCRBD01-01*01*	*TCRBJ02-06*01*
CASESGVRTGELFF	23	0.016365098	42	*TCRBV02-01*01*	*TCRBD01-01*01*	*TCRBJ02-02*01*
CASNLDRFNYGYTF	23	0.016365098	42	*TCRBV19-01*	*TCRBD01-01*01*	*TCRBJ01-02*01*
CASRAFRFNSYNSPLHF	23	0.016365098	51	*TCRBV06*	*TCRBD01-01*01*	*TCRBJ01-06*01*
CASRPGTGGSGELFF	23	0.016365098	45	*TCRBV02-01*01*	*TCRBD01-01*01*	*TCRBJ02-02*01*
CASSISGRGKSYEQYF	23	0.016365098	48	*TCRBV06*	*TCRBD01-01*01*	*TCRBJ02-07*01*
CASSLSRGVGTGELFF	23	0.016365098	48	*TCRBV12*	*unresolved*	*TCRBJ02-02*01*
CASSPRDGGSNQPQHF	23	0.016365098	48	*TCRBV09-01*	*unresolved*	*TCRBJ01-05*01*
CASSQPRQGQETQYF	23	0.016365098	45	*TCRBV16-01*	*TCRBD01-01*01*	*TCRBJ02-05*01*
CSAAGDSLSGNTIYF	23	0.016365098	45	*TCRBV29-01*01*	*TCRBD01-01*01*	*TCRBJ01-03*01*
CSAGDLSGNTIYF	23	0.016365098	39	*TCRBV20*	*TCRBD02-01*02*	*TCRBJ01-03*01*
CAISDGHSNQPQHF	22	0.015653572	42	*TCRBV10-03*01*	*unresolved*	*TCRBJ01-05*01*
CASSLGSRGQPQHF	22	0.015653572	42	*TCRBV11-02*02*	*TCRBD02-01*02*	*TCRBJ01-05*01*
CASSSGARGQPQHF	22	0.015653572	42	*TCRBV11-02*02*	*TCRBD01-01*01*	*TCRBJ01-05*01*
CASSVVGGTGLAEYNEQFF	22	0.015653572	57	*TCRBV09-01*	*TCRBD02-01*	*TCRBJ02-01*01*
CASSYRVGLRDAYEQYF	22	0.015653572	51	*TCRBV27-01*01*	*TCRBD02-01*02*	*TCRBJ02-07*01*
CASSYSAFGQGGNEQFF	22	0.015653572	51	*TCRBV06*	*TCRBD01-01*01*	*TCRBJ02-01*01*

CASSESLRTGAYGYTF	21	0.014942046	48	*TCRBV10-02*01*	*TCRBD01-01*01*	*TCRBJ01-02*01*
CASSFIAGGLSYEQYF	21	0.014942046	48	*TCRBV27-01*01*	*TCRBD02-01*01*	*TCRBJ02-07*01*
CASSLAPTGADAYIEQFF	21	0.014942046	54	*TCRBV07-09*	*TCRBD01-01*01*	*TCRBJ02-01*01*
CASSLGDSRRETQYF	21	0.014942046	45	*TCRBV13-01*01*	*TCRBD01-01*01*	*TCRBJ02-05*01*
CASSPELGVSSSTDTQYF	21	0.014942046	54	*TCRBV28-01*01*	*TCRBD02-01*02*	*TCRBJ02-03*01*
CASSPPGASGGNTGELFF	21	0.014942046	54	*TCRBV18-01*01*	*TCRBD02-01*01*	*TCRBJ02-02*01*
CASSSSLLDGWSYGYTF	21	0.014942046	51	*TCRBV07-02*01*	*unresolved*	*TCRBJ01-02*01*
CSARDLSGDEFNYGYTF	21	0.014942046	51	*TCRBV20*	*TCRBD02-01*01*	*TCRBJ01-02*01*
CSVGDYGYTF	21	0.014942046	30	*TCRBV29-01*01*	*TCRBD02-01*02*	*TCRBJ01-02*01*
CASSARGLQYF	20	0.01423052	33	*TCRBV25-01*01*	*TCRBD01-01*01*	*TCRBJ02-07*01*
CASSEGSETGELFF	20	0.01423052	42	*TCRBV06-01*01*	*TCRBD02-01*	*TCRBJ02-02*01*
CASSEGTSVTDTQYF	20	0.01423052	45	*TCRBV02-01*01*	*TCRBD02-01*	*TCRBJ02-03*01*
CASSIREGSPLHF	20	0.01423052	39	*TCRBV27-01*01*	*TCRBD02-01*02*	*TCRBJ01-06*01*
CASSLTQVLGGYTF	20	0.01423052	42	*TCRBV28-01*01*	*unresolved*	*TCRBJ01-02*01*
CASSQPGSGNTIYF	20	0.01423052	42	*TCRBV25-01*01*	*TCRBD01-01*01*	*TCRBJ01-03*01*
CASSQYQGANEQFF	20	0.01423052	42	*TCRBV04-01*01*	*TCRBD01-01*01*	*TCRBJ02-01*01*
CASSRLAGEGWEQSF	20	0.01423052	45	*TCRBV06-05*01*	*TCRBD02-01*02*	*TCRBJ02*
CASSSARGPEKLFF	20	0.01423052	42	*TCRBV11-01*01*	*unresolved*	*TCRBJ01-04*01*
CASTRDLSAYEQYF	20	0.01423052	42	*TCRBV28-01*01*	*unresolved*	*TCRBJ02-07*01*
CSVGLLNTGELFF	20	0.01423052	39	*TCRBV29-01*01*	*TCRBD02-01*	*TCRBJ02-02*01*
CASMGRLATGRETQYF	19	0.013518994	48	*TCRBV19-01*	*TCRBD01-01*01*	*TCRBJ02-05*01*
CASSLGQGPFF	19	0.013518994	33	*TCRBV11-02*02*	*TCRBD01-01*01*	*TCRBJ01-02*01*
CASSLVPSQGAGVSLHF	19	0.013518994	51	*TCRBV07-02*01*	*TCRBD01-01*01*	*TCRBJ01-06*01*
CASSLWGGQETQYF	19	0.013518994	42	*TCRBV28-01*01*	*unresolved*	*TCRBJ02-05*01*
CASSNRGSYDTEAFF	19	0.013518994	45	*TCRBV19-01*	*TCRBD01-01*01*	*TCRBJ01-01*01*
CASSPGPGVLLLSGNTIYF	19	0.013518994	57	*TCRBV28-01*01*	*TCRBD02-01*01*	*TCRBJ01-03*01*
CASSPKRGRSTGELFF	19	0.013518994	48	*TCRBV11-02*02*	*TCRBD01-01*01*	*TCRBJ02-02*01*
CASTPVYTDANTGELFF	19	0.013518994	51	*TCRBV12*	*unresolved*	*TCRBJ02-02*01*
CASSGLGGGTDTQYF	18	0.012807468	45	*TCRBV06-05*01*	*TCRBD02-01*01*	*TCRBJ02-03*01*
CASSLRVRAGTGELFF	18	0.012807468	48	*TCRBV28-01*01*	*TCRBD01-01*01*	*TCRBJ02-02*01*
CASSPLDGGLSYEQYF	18	0.012807468	48	*TCRBV27-01*01*	*TCRBD02-01*01*	*TCRBJ02-07*01*
CASSRSHNSPLHF	18	0.012807468	39	*TCRBV06-05*01*	*TCRBD02-01*	*TCRBJ01-06*01*

CASSSGTAYTDTQYF	18	0.012807468	45	*TCRBV19-01*	*unresolved*	*TCRBJ02-03*01*
CSALGQGNADEQFF	18	0.012807468	42	*TCRBV20*	*TCRBD01-01*01*	*TCRBJ02-01*01*
CSASFGTEAFF	18	0.012807468	33	*TCRBV20-01*01*	*unresolved*	*TCRBJ01-01*01*
CAISDIGRLNTEAFF	17	0.012095942	45	*TCRBV10-03*01*	*unresolved*	*TCRBJ01-01*01*
CANSGTGGGGSTDTQYF	17	0.012095942	51	*TCRBV28-01*01*	*TCRBD01-01*01*	*TCRBJ02-03*01*
CASRIAGTGFSGANVLTF	17	0.012095942	54	*TCRBV02-01*01*	*TCRBD01-01*01*	*TCRBJ02-06*01*
CASSQKVVIDTGELFF	17	0.012095942	48	*TCRBV02-01*01*	*unresolved*	*TCRBJ02-02*01*
CSARDGGRVYEQYF	17	0.012095942	42	*TCRBV20*	*TCRBD01-01*01*	*TCRBJ02-07*01*
CSASQGGNQPQHF	17	0.012095942	39	*TCRBV20*	*TCRBD01-01*01*	*TCRBJ01-05*01*
CASRRDRTPNQPQHF	16	0.011384416	45	*TCRBV06-05*01*	*TCRBD01-01*01*	*TCRBJ01-05*01*
CASSEGGSSYNSPLHF	16	0.011384416	48	*TCRBV06-01*01*	*TCRBD02-01*	*TCRBJ01-06*01*
CASSLEDRGHYGYTF	16	0.011384416	45	*TCRBV05-01*01*	*TCRBD01-01*01*	*TCRBJ01-02*01*
CASSPGPGRNQPQHF	16	0.011384416	45	*TCRBV05-01*01*	*unresolved*	*TCRBJ01-05*01*
CASSVAYTGELFF	16	0.011384416	39	*TCRBV09-01*	*TCRBD01-01*01*	*TCRBJ02-02*01*
CASTYGQETQYF	16	0.011384416	36	*TCRBV02-01*01*	*TCRBD02-01*	*TCRBJ02-05*01*
CSAHSGRTYEQYF	16	0.011384416	39	*TCRBV20*	*TCRBD02-01*02*	*TCRBJ02-07*01*
CSAISGGAIYNEQFF	16	0.011384416	45	*TCRBV29-01*01*	*TCRBD02-01*01*	*TCRBJ02-01*01*
CSVARTGGTDTQYF	16	0.011384416	42	*TCRBV29-01*01*	*TCRBD01-01*01*	*TCRBJ02-03*01*
CASSEQGPTGELFF	15	0.01067289	42	*TCRBV02-01*01*	*TCRBD01-01*01*	*TCRBJ02-02*01*
CASSGAFPGVGETQYF	15	0.01067289	48	*TCRBV02-01*01*	*TCRBD02-01*01*	*TCRBJ02-05*01*
CASSLDRAAGELFF	15	0.01067289	42	*TCRBV19-01*	*TCRBD01-01*01*	*TCRBJ02-02*01*
CASSLGSEHNEQFF	15	0.01067289	42	*TCRBV11-03*01*	*TCRBD02-01*	*TCRBJ02-01*01*
CASSNSGSYEQYF	15	0.01067289	39	*TCRBV06-05*01*	*TCRBD02-01*	*TCRBJ02-07*01*
CASSPLRLGAAYNSPLHF	15	0.01067289	54	*TCRBV28-01*01*	*TCRBD02-01*	*TCRBJ01-06*01*
CASSQFGKYSGYGYTF	15	0.01067289	48	*TCRBV16-01*	*TCRBD01-01*01*	*TCRBJ01-02*01*
CASSQTGLERDTQYF	15	0.01067289	45	*TCRBV12*	*unresolved*	*TCRBJ02-03*01*
CASSYHRGTDEQFF	15	0.01067289	42	*TCRBV27-01*01*	*TCRBD01-01*01*	*TCRBJ02-01*01*
CATPGQGTNYSPLHF	15	0.01067289	45	*TCRBV06-01*01*	*TCRBD01-01*01*	*TCRBJ01-06*01*
CSARGGTRGSYEQYF	15	0.01067289	45	*TCRBV20*	*unresolved*	*TCRBJ02-07*01*
CSATQSVSDTQYF	15	0.01067289	39	*TCRBV20*	*TCRBD01-01*01*	*TCRBJ02-03*01*
CSVSGAGGQPQHF	15	0.01067289	39	*TCRBV29-01*01*	*TCRBD02-01*01*	*TCRBJ01-05*01*
CAIRGGQATFADTQYF	14	0.009961364	48	*TCRBV10-03*01*	*TCRBD01-01*01*	*TCRBJ02-03*01*

CASGAGFSGAQGPYEQYF	14	0.009961364	54	*TCRBV02-01*01*	*unresolved*	*TCRBJ02-07*01*
CASRSTGTTAGGEQFF	14	0.009961364	48	*TCRBV05-01*01*	*TCRBD02-01*01*	*TCRBJ02-01*01*
CASSDQGVSPLHF	14	0.009961364	39	*TCRBV25-01*01*	*TCRBD01-01*01*	*TCRBJ01-06*01*
CASSEGGAGGYTF	14	0.009961364	39	*TCRBV02-01*01*	*unresolved*	*TCRBJ01-02*01*
CASSFPRDRVSYEQYF	14	0.009961364	48	*TCRBV11-02*02*	*TCRBD01-01*01*	*TCRBJ02-07*01*
CASSPLTATEAFF	14	0.009961364	39	*TCRBV18-01*01*	*TCRBD01-01*01*	*TCRBJ01-01*01*
CASSPVGGFANTGELFF	14	0.009961364	51	*TCRBV09-01*	*TCRBD02-01*02*	*TCRBJ02-02*01*
CASSVGVGSGANVLTF	14	0.009961364	48	*TCRBV09-01*	*TCRBD02-01*02*	*TCRBJ02-06*01*
CASRPSWLAGGPGDTQYF	13	0.009249838	54	*TCRBV25-01*01*	*TCRBD02-01*02*	*TCRBJ02-03*01*
CASRRPNSPLHF	13	0.009249838	36	*TCRBV07-02*01*	*unresolved*	*TCRBJ01-06*01*
CASSEAMAGPDTQYF	13	0.009249838	45	*TCRBV02-01*01*	*TCRBD02-01*02*	*TCRBJ02-03*01*
CASSLTQGLSYNEQFF	13	0.009249838	48	*TCRBV06-06*	*TCRBD01-01*01*	*TCRBJ02-01*01*
CASSQASGELFF	13	0.009249838	36	*TCRBV04-01*01*	*TCRBD02-01*	*TCRBJ02-02*01*
CASSQDMTGTVHNSPLHF	13	0.009249838	54	*TCRBV07-02*01*	*TCRBD01-01*01*	*TCRBJ01-06*01*
CASSSFGSGDSYEQYF	13	0.009249838	48	*TCRBV07-09*	*TCRBD02-01*01*	*TCRBJ02-07*01*
CASSSGLLDTQYF	13	0.009249838	39	*TCRBV27-01*01*	*TCRBD02-01*	*TCRBJ02-03*01*
CASSSSAVAGRRVYEQYF	13	0.009249838	54	*TCRBV07-09*	*TCRBD02-01*01*	*TCRBJ02-07*01*
CASSVEGSGGAGETQYF	13	0.009249838	51	*TCRBV09-01*	*TCRBD02-01*01*	*TCRBJ02-05*01*
CATSPAAWENTGELFF	13	0.009249838	48	*TCRBV15-01*01*	*unresolved*	*TCRBJ02-02*01*
CSARGPSQGYEQYF	13	0.009249838	42	*TCRBV20*	*unresolved*	*TCRBJ02-07*01*
CSASAFAGLGDTQYF	13	0.009249838	45	*TCRBV20*	*TCRBD02-01*01*	*TCRBJ02-03*01*
CSASMPGSGSPLHF	13	0.009249838	42	*TCRBV20*	*TCRBD02-01*01*	*TCRBJ01-06*01*
CASSLMPGQKNEQYF	12	0.008538312	45	*TCRBV05-01*01*	*TCRBD01-01*01*	*TCRBJ02-07*01*
CASSSGLAGDEQFF	12	0.008538312	42	*TCRBV07-03*01*	*TCRBD02-01*	*TCRBJ02-01*01*
CSARVPHPGQGLDQPQHF	12	0.008538312	54	*TCRBV20*	*TCRBD01-01*01*	*TCRBJ01-05*01*
CASRIQGTGNTIYF	11	0.007826786	42	*TCRBV28-01*01*	*TCRBD01-01*01*	*TCRBJ01-03*01*
CASSEGLAGGPDTQYF	11	0.007826786	48	*TCRBV06-01*01*	*TCRBD02-01*01*	*TCRBJ02-03*01*
CASSLAVRDSPLHF	11	0.007826786	42	*TCRBV05-01*01*	*TCRBD02-01*	*TCRBJ01-06*01*
CASSQSANAYNEQFF	11	0.007826786	45	*TCRBV11-02*02*	*unresolved*	*TCRBJ02-01*01*
CASSSGTGDTQYF	11	0.007826786	39	*TCRBV11-03*01*	*TCRBD02-01*	*TCRBJ02-03*01*
CASTPVGAQSVSQYF	11	0.007826786	45	*TCRBV02-01*01*	*TCRBD02-01*	*TCRBJ02-03*01*
CSARSGWIDTGELFF	11	0.007826786	45	*TCRBV20*	*TCRBD02-01*	*TCRBJ02-02*01*

CSATTSVEQYF	11	0.007826786	33	*TCRBV20-01*01*	*unresolved*	*TCRBJ02-07*01*
CSVRGTGGRSSGNTIYF	11	0.007826786	51	*TCRBV29-01*01*	*TCRBD01-01*01*	*TCRBJ01-03*01*
CASSLTDYNSPLHF	10	0.00711526	42	*TCRBV05-06*01*	*unresolved*	*TCRBJ01-06*01*
CASSPTGRTGELFF	10	0.00711526	42	*TCRBV18-01*01*	*TCRBD01-01*01*	*TCRBJ02-02*01*
CASSQVGRTSTDTQYF	10	0.00711526	48	*TCRBV28-01*01*	*TCRBD02-01*02*	*TCRBJ02-03*01*
CASSTGLRYEQYF	10	0.00711526	39	*TCRBV19-01*	*unresolved*	*TCRBJ02-07*01*
CSARDINGATRHGYTF	10	0.00711526	48	*TCRBV20*	*TCRBD01-01*01*	*TCRBJ01-02*01*
CSVDLTGTYEQYF	10	0.00711526	39	*TCRBV29-01*01*	*TCRBD01-01*01*	*TCRBJ02-05*01*
CSVEFVTGQDVGTDTQYF	10	0.00711526	54	*TCRBV29-01*01*	*TCRBD01-01*01*	*TCRBJ02-03*01*
CASSRELTEYSNQPQHF	9	0.006403734	51	*TCRBV19-01*	*unresolved*	*TCRBJ01-05*01*
CASSSPKGVRDYTF	9	0.006403734	42	*TCRBV07-09*	*TCRBD01-01*01*	*TCRBJ01-02*01*
CASSSTFPGMYNEQFF	9	0.006403734	48	*TCRBV07-09*	*TCRBD02-01*02*	*TCRBJ02-01*01*
CATSRVMGREETQYF	9	0.006403734	45	*TCRBV15-01*01*	*unresolved*	*TCRBJ02-05*01*
CSAITGSYEQYF	9	0.006403734	36	*TCRBV20*	*TCRBD01-01*01*	*TCRBJ02-07*01*
CSATDRETSTDTQYF	9	0.006403734	45	*TCRBV20*	*TCRBD01-01*01*	*TCRBJ02-03*01*
CSATPGLAGAGQFF	9	0.006403734	42	*TCRBV20*	*TCRBD02-01*01*	*TCRBJ02-01*01*
CASSQRGQLLTGELFF	8	0.005692208	48	*TCRBV19-01*	*TCRBD01-01*01*	*TCRBJ02-02*01*
CASSRSGFRSNQPQHF	8	0.005692208	48	*TCRBV12*	*TCRBD01-01*01*	*TCRBJ01-05*01*
CSAKWDLSYNEQFF	8	0.005692208	42	*TCRBV20*	*unresolved*	*TCRBJ02-01*01*
CSAQQDYEQYF	8	0.005692208	33	*TCRBV20-01*01*	*TCRBD01-01*01*	*TCRBJ02-07*01*
CSAYRGAETQYF	8	0.005692208	36	*TCRBV20*	*TCRBD01-01*01*	*TCRBJ02-05*01*
CAGSYSWWDEQYF	7	0.004980682	39	*TCRBV05-05*01*	*unresolved*	*TCRBJ02-07*01*
CAISEIGQTTTYNEQFF	7	0.004980682	51	*TCRBV10-03*01*	*TCRBD01-01*01*	*TCRBJ02-01*01*
CASRPGLAVTPTDTQYF	7	0.004980682	51	*TCRBV12*	*TCRBD02-01*	*TCRBJ02-03*01*
CASSLSATGGTGELFF	7	0.004980682	48	*TCRBV27-01*01*	*TCRBD01-01*01*	*TCRBJ02-02*01*
CASSSDGVNYGYTF	7	0.004980682	42	*TCRBV11-02*02*	*TCRBD02-01*01*	*TCRBJ01-02*01*
CASSSWGSLYEQYF	7	0.004980682	42	*TCRBV11-01*01*	*unresolved*	*TCRBJ02-07*01*
CSARDPDSYEQYF	7	0.004980682	39	*TCRBV20*	*unresolved*	*TCRBJ02-07*01*
CASSADGAANSPLHF	6	0.004269156	45	*TCRBV09-01*	*unresolved*	*TCRBJ01-06*01*
CASSLTGTFNGNSPLHF	6	0.004269156	51	*TCRBV07-02*01*	*TCRBD01-01*01*	*TCRBJ01-06*01*
CASSLTSYTGELFF	6	0.004269156	42	*TCRBV27-01*01*	*TCRBD02-01*	*TCRBJ02-02*01*
CASSPGARVTEAFF	6	0.004269156	42	*TCRBV19-01*	*TCRBD01-01*01*	*TCRBJ01-01*01*

CASSQGGAGGTDTQYF	6	0.004269156	48	*TCRBV03*	*TCRBD02-01*01*	*TCRBJ02-03*01*
CASSVLDISPLHF	6	0.004269156	39	*TCRBV09-01*	*TCRBD01-01*01*	*TCRBJ01-06*01*
CAIKGGASGYTF	5	0.00355763	36	*TCRBV10-01*	*TCRBD02-01*02*	*TCRBJ01-02*01*
CASRISGGETQYF	5	0.00355763	39	*TCRBV11-02*02*	*TCRBD02-01*01*	*TCRBJ02-05*01*
CASSLVASPNTGELFF	5	0.00355763	48	*TCRBV11-02*02*	*TCRBD01-01*01*	*TCRBJ02-02*01*
CAWSVSGELFF	5	0.00355763	33	*TCRBV30-01*01*	*unresolved*	*TCRBJ02-02*01*
CSARDQGGTQSYNSPLHF	5	0.00355763	54	*TCRBV20*	*TCRBD01-01*01*	*TCRBJ01-06*01*
CSARDSVVQDFYGYTF	5	0.00355763	48	*TCRBV20*	*TCRBD02-01*	*TCRBJ01-02*01*
CAGSSGTNAYGYTF	4	0.002846104	42	*TCRBV06-01*01*	*TCRBD01-01*01*	*TCRBJ01-02*01*
CASSIGGSEAFF	4	0.002846104	36	*TCRBV19-01*	*unresolved*	*TCRBJ01-01*01*
CASSLEVAGGFTSTDTQYF	4	0.002846104	57	*TCRBV11-03*01*	*TCRBD02-01*01*	*TCRBJ02-03*01*
CSAFAVRGGITDTQYF	4	0.002846104	48	*TCRBV20*	*TCRBD02-01*01*	*TCRBJ02-03*01*
CSAGQGVTNSPLHF	4	0.002846104	42	*TCRBV20*	*TCRBD01-01*01*	*TCRBJ01-06*01*
CSARDSSDRVFLDTQYF	4	0.002846104	51	*TCRBV20*	*TCRBD01-01*01*	*TCRBJ02-03*01*
CSARVYSLAGGGITSTDTQYF	4	0.002846104	63	*TCRBV20*	*TCRBD02-01*01*	*TCRBJ02-03*01*
CSASPSAMNTEAFF	4	0.002846104	42	*TCRBV20*	*TCRBD02-01*	*TCRBJ01-01*01*
CSGSGPNAQYF	4	0.002846104	33	*TCRBV20-01*01*	*unresolved*	*TCRBJ02-05*01*
CSVSGAGGQPQHF	4	0.002846104	39	*TCRBV29-01*01*	*TCRBD02-01*01*	*TCRBJ01-05*01*
CASSEMASGSFYNEQFF	3	0.002134578	51	*TCRBV06-01*01*	*TCRBD02-01*02*	*TCRBJ02-01*01*
CASSEVVAAGNQPRHF	3	0.002134578	48	*TCRBV06-01*01*	*TCRBD02-01*	*TCRBJ01-05*01*
CASSGYEQYF	3	0.002134578	30	*TCRBV02-01*01*	*unresolved*	*TCRBJ02-07*01*
CASSQFPGQALFQSSGNTIYF	3	0.002134578	63	*TCRBV18-01*01*	*TCRBD01-01*01*	*TCRBJ01-03*01*
CSAHLGGGSNQPQHF	3	0.002134578	45	*TCRBV20*	*TCRBD02-01*01*	*TCRBJ01-05*01*
CSAIGLASSYNEQFF	3	0.002134578	45	*TCRBV20*	*TCRBD02-01*	*TCRBJ02-01*01*
CSARESSHPGANVLTF	3	0.002134578	48	*TCRBV20*	*TCRBD02-01*	*TCRBJ02-06*01*
CSARGPGVGETQYF	3	0.002134578	42	*TCRBV20*	*TCRBD02-01*01*	*TCRBJ02-05*01*
CSASTTSGGASYNEQFF	3	0.002134578	51	*TCRBV20*	*TCRBD02-01*01*	*TCRBJ02-01*01*
CAALGGGEETQYF	2	0.001423052	39	*TCRBV30-01*01*	*TCRBD02-01*01*	*TCRBJ02-05*01*
CAGRTHHNEQFF	2	0.001423052	36	*TCRBV30-01*01*	*TCRBD01-01*01*	*TCRBJ02-01*01*
CAIKGGVSGYTS	2	0.001423052	36	*TCRBV10-01*	*TCRBD02-01*02*	*TCRBJ01-02*01*
CASGTSGSYEQYF	2	0.001423052	39	*TCRBV06-01*01*	*TCRBD02-01*02*	*TCRBJ02-07*01*
CASKFSYAYERYF	2	0.001423052	39	*TCRBV02-01*01*	*TCRBD02-01*	*TCRBJ02-07*01*

CASPPWGGQETQYF	2	0.001423052	42	*TCRBV07-06*01*	*TCRBD02-01*01*	*TCRBJ02-05*01*
CASSHSGTGGLGYTF	2	0.001423052	45	*TCRBV03*	*TCRBD01-01*01*	*TCRBJ01-02*01*
CASSLAVRDSPLHF	2	0.001423052	42	*TCRBV05-01*01*	*TCRBD02-01*	*TCRBJ01-06*01*
CASSLDRGAYSGANVLTF	2	0.001423052	54	*TCRBV07-02*01*	*TCRBD01-01*01*	*TCRBJ02-06*01*
CASSLEVLDNSPLHF	2	0.001423052	45	*TCRBV05-01*01*	*unresolved*	*TCRBJ01-06*01*
CASSLSGTGVHEQYF	2	0.001423052	45	*TCRBV12*	*TCRBD01-01*01*	*TCRBJ02-07*01*
CASSSGTNAYGHTF	2	0.001423052	42	*TCRBV06-01*01*	*TCRBD01-01*01*	*TCRBJ01-02*01*
CAWTPGYPNYGYPF	2	0.001423052	42	*TCRBV30-01*01*	*TCRBD01-01*01*	*TCRBJ01-02*01*
CAWTPGYPNYGYTF	2	0.001423052	42	*TCRBV30-01*01*	*TCRBD01-01*01*	*TCRBJ01-02*01*
CSADPRGLAARDEQFF	2	0.001423052	48	*TCRBV20*	*TCRBD02-01*	*TCRBJ02-01*01*
CSAIDFGLAGTLPVKETQYF	2	0.001423052	60	*TCRBV20*	*TCRBD02-01*02*	*TCRBJ02-05*01*
CSARTLLKGAAGEQYF	2	0.001423052	48	*TCRBV20*	*TCRBD01-01*01*	*TCRBJ02-07*01*
**LESION III**						
CASNPGTAYSYEQYF	4729	9.328336128	45	*TCRBV06-01*01*	*TCRBD01-01*01*	*TCRBJ02-07*01*
CASSYNRGDRGGYEQYF	1781	3.513166979	51	*TCRBV18-01*01*	*TCRBD01-01*01*	*TCRBJ02-07*01*
CASSLSGTGVYEQYF	1559	3.07525397	45	*TCRBV12*	*TCRBD01-01*01*	*TCRBJ02-07*01*
CASNHAWVSNQPQHF	1189	2.345398955	45	*TCRBV23-01*01*	*unresolved*	*TCRBJ01-05*01*
CASRPPSDRWDYGYTF	1113	2.195482789	48	*TCRBV28-01*01*	*unresolved*	*TCRBJ01-02*01*
CASSFPSTDTQYF	812	1.601735871	39	*TCRBV05-06*01*	*TCRBD02-01*02*	*TCRBJ02-03*01*
CASSFSGRSDEQYF	700	1.380806786	42	*TCRBV19-01*	*TCRBD02-01*02*	*TCRBJ02-07*01*
CASSPDRVVGYTF	683	1.347272907	39	*TCRBV19-01*	*unresolved*	*TCRBJ01-02*01*
CASSFGQAASPLHF	615	1.21313739	42	*TCRBV12*	*unresolved*	*TCRBJ01-06*01*
CASSSGTNAYGYTF	588	1.1598777	42	*TCRBV06-01*01*	*TCRBD01-01*01*	*TCRBJ01-02*01*
CASSQADSLYEQYF	478	0.942893777	42	*TCRBV23-01*01*	*TCRBD01-01*01*	*TCRBJ02-07*01*
CSAFSTTLNEQFF	451	0.889634086	39	*TCRBV20*	*unresolved*	*TCRBJ02-01*01*
CASSLSAGGLGYTF	411	0.810730841	42	*TCRBV28-01*01*	*TCRBD01-01*01*	*TCRBJ01-02*01*
CASSHGTGNQPQHF	406	0.800867936	42	*TCRBV06-05*01*	*TCRBD01-01*01*	*TCRBJ01-05*01*
CASSVEWTGEQYF	392	0.7732518	39	*TCRBV09-01*	*TCRBD02-01*	*TCRBJ02-07*01*
CASSQSAATGNYEQYF	390	0.769306638	48	*TCRBV07-06*01*	*TCRBD01-01*01*	*TCRBJ02-07*01*
CASRTALHHSDNEQFF	386	0.761416313	48	*TCRBV21-01*01*	*unresolved*	*TCRBJ02-01*01*
CASSIQEWSTEAFF	385	0.759443732	42	*TCRBV19-01*	*unresolved*	*TCRBJ01-01*01*

CASERGGSSYEQYF	379	0.747608245	42	*TCRBV28-01*01*	*TCRBD01-01*01*	*TCRBJ02-07*01*
CASSEPGAHNYGYTF	374	0.73774534	45	*TCRBV06*	*TCRBD02-01*02*	*TCRBJ01-02*01*
CASSHGQGATRNGYTF	372	0.733800178	48	*TCRBV12*	*TCRBD01-01*01*	*TCRBJ01-02*01*
CASSGMNTARQSRANVLTF	371	0.731827596	57	*TCRBV12*	*unresolved*	*TCRBJ02-06*01*
CSAGGQAVQPQHF	367	0.723937272	39	*TCRBV20*	*TCRBD01-01*01*	*TCRBJ01-05*01*
CASNPGTTNGYTF	341	0.672650163	39	*TCRBV19-01*	*TCRBD01-01*01*	*TCRBJ01-02*01*
CASSPNGGTNEKLFF	339	0.668705	45	*TCRBV07-03*01*	*unresolved*	*TCRBJ01-04*01*
CASSLGGTEYYGYTF	336	0.662787257	45	*TCRBV07-09*	*TCRBD01-01*01*	*TCRBJ01-02*01*
CASSQGGNSYEQYF	336	0.662787257	42	*TCRBV03*	*unresolved*	*TCRBJ02-07*01*
CAIGDGAAGANVLTF	320	0.631225959	45	*TCRBV10-03*01*	*unresolved*	*TCRBJ02-06*01*
CASSFGTDGYTF	318	0.627280797	36	*TCRBV05-06*01*	*unresolved*	*TCRBJ01-02*01*
CASQVEGINYGYTF	316	0.623335635	42	*TCRBV10-01*	*TCRBD02-01*02*	*TCRBJ01-02*01*
CASSLGTGGRSEAFF	315	0.621363054	45	*TCRBV18-01*01*	*TCRBD01-01*01*	*TCRBJ01-01*01*
CASSRLLNTIYF	315	0.621363054	36	*TCRBV03*	*TCRBD02-01*	*TCRBJ01-03*01*
CAISGPPGRALYGYTF	304	0.599664661	48	*TCRBV10-03*01*	*unresolved*	*TCRBJ01-02*01*
CASSLGQGLHEQYF	303	0.59769208	42	*TCRBV05-06*01*	*TCRBD01-01*01*	*TCRBJ02-07*01*
CASSEQGPQSRANVLTF	296	0.583884012	51	*TCRBV07-07*01*	*TCRBD01-01*01*	*TCRBJ02-06*01*
CASSPDGGKTGELFF	293	0.577966269	45	*TCRBV07-03*01*	*TCRBD02-01*	*TCRBJ02-02*01*
CAISDGHSNQPQHF	285	0.56218562	42	*TCRBV10-03*01*	*unresolved*	*TCRBJ01-05*01*
CASSTGLAGGPESYEQYF	282	0.556267877	54	*TCRBV05-01*01*	*TCRBD02-01*02*	*TCRBJ02-07*01*
CASSLITAGAYEQYF	280	0.552322714	45	*TCRBV28-01*01*	*TCRBD02-01*02*	*TCRBJ02-07*01*
CASSLAYRDESEQYF	279	0.550350133	45	*TCRBV05-06*01*	*TCRBD02-01*02*	*TCRBJ02-07*01*
CASSPTGDSGYEQYF	272	0.536542065	45	*TCRBV04-01*01*	*TCRBD01-01*01*	*TCRBJ02-07*01*
CASSSVAGGPYEQYF	267	0.52667916	45	*TCRBV07-09*	*TCRBD02-01*02*	*TCRBJ02-07*01*
CATPRVDGYTF	267	0.52667916	33	*TCRBV11-02*02*	*TCRBD01-01*01*	*TCRBJ01-02*01*
CASRLGQGAYEQYF	263	0.518788835	42	*TCRBV19-01*	*TCRBD01-01*01*	*TCRBJ02-07*01*
CASSQVQDGEQYF	261	0.514843673	39	*TCRBV04-01*01*	*unresolved*	*TCRBJ02-07*01*
CAISVEPLNEKLFF	260	0.512871092	42	*TCRBV10-03*01*	*TCRBD02-01*	*TCRBJ01-04*01*
CASSSGQDYSPLHF	257	0.506953348	42	*TCRBV05-01*01*	*TCRBD01-01*01*	*TCRBJ01-06*01*
CASNRRTNWNTGELFF	256	0.504980767	48	*TCRBV06*	*unresolved*	*TCRBJ02-02*01*
CASSPPSSSGGELFF	256	0.504980767	45	*TCRBV18-01*01*	*TCRBD02-01*	*TCRBJ02-02*01*
CASSNAVATLHHQPQHF	247	0.487227537	51	*TCRBV21-01*01*	*TCRBD02-01*	*TCRBJ01-05*01*

CASNVEGRREKLFF	246	0.485254956	42	*TCRBV06-01*01*	*TCRBD02-01*02*	*TCRBJ01-04*01*
CASSREIATIHHNSPLHF	246	0.485254956	54	*TCRBV21-01*01*	*TCRBD02-01*	*TCRBJ01-06*01*
CASSMARALSHGSYNEQFF	242	0.477364632	57	*TCRBV19-01*	*TCRBD02-01*	*TCRBJ02-01*01*
CASSITLRSYEQYF	240	0.473419469	42	*TCRBV19-01*	*TCRBD02-01*	*TCRBJ02-07*01*
CASSHLRSGGDYEQYF	239	0.471446888	48	*TCRBV07-06*01*	*TCRBD01-01*01*	*TCRBJ02-07*01*
CASSPLPGNENSPLHF	238	0.469474307	48	*TCRBV18-01*01*	*unresolved*	*TCRBJ01-06*01*
CASSSLRGNTEAFF	236	0.465529145	42	*TCRBV11-02*02*	*unresolved*	*TCRBJ01-01*01*
CASSRRENSNQPQHF	235	0.463556564	45	*TCRBV06-01*01*	*TCRBD02-01*02*	*TCRBJ01-05*01*
CASSQAYGGGGTEAFF	226	0.445803334	48	*TCRBV04-01*01*	*unresolved*	*TCRBJ01-01*01*
CASSSQGYEQYF	222	0.437913009	36	*TCRBV07-06*01*	*TCRBD01-01*01*	*TCRBJ02-07*01*
CASSLVPTGYSNQPQHF	217	0.428050104	51	*TCRBV05-01*01*	*TCRBD01-01*01*	*TCRBJ01-05*01*
CASSVLEGTDTQYF	217	0.428050104	42	*TCRBV06-06*	*TCRBD02-01*02*	*TCRBJ02-03*01*
CASSLAQGSTGELFF	216	0.426077522	45	*TCRBV13-01*01*	*TCRBD01-01*01*	*TCRBJ02-02*01*
CASSDNYPFVMQDTQYF	211	0.416214617	51	*TCRBV03*	*unresolved*	*TCRBJ02-03*01*
CSARDEGFYGYTF	209	0.412269455	39	*TCRBV20*	*TCRBD02-01*02*	*TCRBJ01-02*01*
CASSQGTGGIGNSPLHF	208	0.410296873	51	*TCRBV06*	*TCRBD01-01*01*	*TCRBJ01-06*01*
CSAGTIDLNTEAFF	203	0.400433968	42	*TCRBV29-01*01*	*unresolved*	*TCRBJ01-01*01*
CSASSWGNYEQYF	203	0.400433968	39	*TCRBV20*	*unresolved*	*TCRBJ02-07*01*
CASSPLPRGLGYGYTF	199	0.392543643	48	*TCRBV28-01*01*	*TCRBD01-01*01*	*TCRBJ01-02*01*
CASTTGPWTYEQYF	199	0.392543643	42	*TCRBV05-05*01*	*TCRBD01-01*01*	*TCRBJ02-07*01*
CASSQACPILLDEQYF	196	0.3866259	48	*TCRBV03*	*unresolved*	*TCRBJ02-07*01*
CASSSVDWGSPLHF	195	0.384653319	42	*TCRBV07-09*	*unresolved*	*TCRBJ01-06*01*
CASSYGIRGVGTGELFF	195	0.384653319	51	*TCRBV06*	*TCRBD02-01*01*	*TCRBJ02-02*01*
CASSLFDRGGNEKLFF	194	0.382680738	48	*TCRBV28-01*01*	*TCRBD02-01*02*	*TCRBJ01-04*01*
CASSLAWVGNYGYTF	193	0.380708157	45	*TCRBV07-02*01*	*unresolved*	*TCRBJ01-02*01*
CASSPIGESWGEQYF	192	0.378735576	45	*TCRBV04-02*01*	*unresolved*	*TCRBJ02-07*01*
CASSFGTLYSNQPQHF	191	0.376762994	48	*TCRBV06-05*01*	*unresolved*	*TCRBJ01-05*01*
CASSFPARTEQYF	190	0.374790413	39	*TCRBV07-02*01*	*unresolved*	*TCRBJ02-07*01*
CASSYLSPIRKNIQYF	190	0.374790413	48	*TCRBV03*	*TCRBD02-01*	*TCRBJ02-04*01*
CASSLENQPQHF	189	0.372817832	36	*TCRBV05-06*01*	*TCRBD02-01*02*	*TCRBJ01-05*01*
CASRRGTSGSYEQYF	185	0.364927508	45	*TCRBV03*	*TCRBD02-01*02*	*TCRBJ02-07*01*
CASSPQDRGLRDGYTF	184	0.362954927	48	*TCRBV27-01*01*	*TCRBD01-01*01*	*TCRBJ01-02*01*

CASSKSLPGQFHSYEQYF	183	0.360982345	54	*TCRBV21-01*01*	*TCRBD01-01*01*	*TCRBJ02-07*01*
CASTGAGANVLTF	182	0.359009764	39	*TCRBV05-01*01*	*TCRBD01-01*01*	*TCRBJ02-06*01*
CASSPARGTGANVLTF	181	0.357037183	48	*TCRBV28-01*01*	*TCRBD02-01*02*	*TCRBJ02-06*01*
CASRQGAYTF	179	0.353092021	30	*TCRBV06-01*01*	*TCRBD01-01*01*	*TCRBJ01-02*01*
CASSRSLTSYEQYF	176	0.347174278	42	*TCRBV09-01*	*unresolved*	*TCRBJ02-07*01*
CASSQDLAGAQNTGELFF	175	0.345201696	54	*TCRBV04-01*01*	*TCRBD02-01*01*	*TCRBJ02-02*01*
CASSS*QTYGKTYGYTF	175	0.345201696	51	*TCRBV06-09*01*	*TCRBD02-01*	*TCRBJ01-02*01*
CASSLVVTQYF	173	0.341256534	33	*TCRBV07-09*	*unresolved*	*TCRBJ02-05*01*
CASSQDFDSYFTDTQYF	171	0.337311372	51	*TCRBV04-01*01*	*TCRBD01-01*01*	*TCRBJ02-03*01*
CASSHRASGANVLTF	170	0.335338791	45	*TCRBV07-02*01*	*TCRBD01-01*01*	*TCRBJ02-06*01*
CASSLIGTLSTDTQYF	170	0.335338791	48	*TCRBV12*	*TCRBD01-01*01*	*TCRBJ02-03*01*
CASSLASNEQFF	169	0.33336621	36	*TCRBV05-05*01*	*TCRBD02-01*	*TCRBJ02-01*01*
CASSSWGSLYEQYF	169	0.33336621	42	*TCRBV11-01*01*	*unresolved*	*TCRBJ02-07*01*
CASSHDGAEAFF	168	0.331393629	36	*TCRBV04-01*01*	*unresolved*	*TCRBJ01-01*01*
CASSHNTGGNQPQHF	167	0.329421047	45	*TCRBV06-05*01*	*TCRBD01-01*01*	*TCRBJ01-05*01*
CASTGGISYEQYF	167	0.329421047	39	*TCRBV02-01*01*	*TCRBD01-01*01*	*TCRBJ02-07*01*
CAIIPGFHSPLHF	166	0.327448466	39	*TCRBV02-01*01*	*TCRBD01-01*01*	*TCRBJ01-06*01*
CASSFRGSGYEQYF	164	0.323503304	42	*TCRBV28-01*01*	*TCRBD02-01*02*	*TCRBJ02-07*01*
CASSLLGRTSTDTQYF	164	0.323503304	48	*TCRBV12*	*unresolved*	*TCRBJ02-03*01*
CASSYDSAGGYTF	164	0.323503304	39	*TCRBV05-05*01*	*TCRBD02-01*	*TCRBJ01-02*01*
CASSPPGRGSYEQYF	163	0.321530723	45	*TCRBV06-06*	*TCRBD01-01*01*	*TCRBJ02-07*01*
CSAPPHGRTYNEQFF	163	0.321530723	45	*TCRBV20*	*unresolved*	*TCRBJ02-01*01*
CASRSGGAREQYF	162	0.319558142	39	*TCRBV06-01*01*	*TCRBD01-01*01*	*TCRBJ02-07*01*
CASSFYRGQGTEAFF	162	0.319558142	45	*TCRBV05-01*01*	*TCRBD01-01*01*	*TCRBJ01-01*01*
CASSTGTSYEQYF	162	0.319558142	39	*TCRBV05-01*01*	*TCRBD02-01*	*TCRBJ02-07*01*
CASGPEGLATYNEQFF	161	0.317585561	48	*TCRBV12-05*01*	*TCRBD02-01*	*TCRBJ02-01*01*
CASWDRGGSPLHF	159	0.313640398	39	*TCRBV02-01*01*	*TCRBD01-01*01*	*TCRBJ01-06*01*
CSAFPGRGSNQPQHF	159	0.313640398	45	*TCRBV20*	*TCRBD01-01*01*	*TCRBJ01-05*01*
CASSNSGSYEQYF	158	0.311667817	39	*TCRBV06-05*01*	*TCRBD02-01*	*TCRBJ02-07*01*
CSAREPQGGGYTF	158	0.311667817	39	*TCRBV20*	*TCRBD01-01*01*	*TCRBJ01-02*01*
CSARDLRQGTSNTEAFF	157	0.309695236	51	*TCRBV20*	*TCRBD01-01*01*	*TCRBJ01-01*01*
CASSPSMNTEAFF	156	0.307722655	39	*TCRBV11-02*02*	*unresolved*	*TCRBJ01-01*01*

CASSLGRADTQYF	155	0.305750074	39	*TCRBV13-01*01*	*unresolved*	*TCRBJ02-03*01*
CASTGLDRTNTGELFF	152	0.299832331	48	*TCRBV19-01*	*TCRBD01-01*01*	*TCRBJ02-02*01*
CASRRRGGNLSDEQYF	150	0.295887168	48	*TCRBV05-01*01*	*TCRBD02-01*02*	*TCRBJ02-07*01*
CASSASVATWHHQPQHF	150	0.295887168	51	*TCRBV21-01*01*	*TCRBD02-01*	*TCRBJ01-05*01*
CASSTRGRTSTNYGYTF	149	0.293914587	51	*TCRBV07-09*	*unresolved*	*TCRBJ01-02*01*
CASSVGGLAGDEQFF	149	0.293914587	45	*TCRBV09-01*	*TCRBD02-01*02*	*TCRBJ02-01*01*
CAWSVPAGNGANVLTF	149	0.293914587	48	*TCRBV30-01*01*	*TCRBD01-01*01*	*TCRBJ02-06*01*
CARPIGNTGELFF	146	0.287996844	39	*TCRBV12*	*TCRBD01-01*01*	*TCRBJ02-02*01*
CASSGHRMGYGYTF	145	0.286024263	42	*TCRBV06-06*	*TCRBD01-01*01*	*TCRBJ01-02*01*
CASSLARGSETQYF	143	0.282079101	42	*TCRBV05-01*01*	*unresolved*	*TCRBJ02-05*01*
CASSPNRFNEQYF	143	0.282079101	39	*TCRBV06-01*01*	*unresolved*	*TCRBJ02-07*01*
CSAWVGPQSGTDTQYF	143	0.282079101	48	*TCRBV20*	*unresolved*	*TCRBJ02-03*01*
CASSHGTDQIYNEQFF	142	0.280106519	48	*TCRBV14-01*01*	*TCRBD02-01*	*TCRBJ02-01*01*
CASSLALTDTQYF	142	0.280106519	39	*TCRBV07-08*01*	*unresolved*	*TCRBJ02-03*01*
CASSLGHEGYTF	142	0.280106519	36	*TCRBV12*	*TCRBD01-01*01*	*TCRBJ01-02*01*
CASSQDGEAIGGKNIQYF	142	0.280106519	54	*TCRBV04-01*01*	*TCRBD02-01*02*	*TCRBJ02-04*01*
CSAPRVREGVEQYF	142	0.280106519	42	*TCRBV20*	*TCRBD02-01*02*	*TCRBJ02-07*01*
CSARRGQNSLYEQYF	142	0.280106519	45	*TCRBV20*	*TCRBD01-01*01*	*TCRBJ02-07*01*
CSARTLLKGAAGEQYF	141	0.278133938	48	*TCRBV20*	*TCRBD01-01*01*	*TCRBJ02-07*01*
CASSLSRDPGDTQYF	140	0.276161357	45	*TCRBV12*	*unresolved*	*TCRBJ02-03*01*
CASSFLRGPKRYNEQFF	138	0.272216195	51	*TCRBV12*	*unresolved*	*TCRBJ02-01*01*
CASSIVTRSRKTYGY	135	0.266298452	45	*TCRBV06-08*01*	*TCRBD02-01*	*TCRBJ01-02*01*
CASKLANTEAFF	133	0.262353289	36	*TCRBV07-09*	*TCRBD02-01*	*TCRBJ01-01*01*
CASSPTITGEEQYF	133	0.262353289	42	*TCRBV07-03*01*	*TCRBD01-01*01*	*TCRBJ02-07*01*
CASSSPGVETQYF	131	0.258408127	39	*TCRBV05-06*01*	*TCRBD01-01*01*	*TCRBJ02-05*01*
CASSNRVLSYGYTF	129	0.254462965	42	*TCRBV11-02*02*	*TCRBD01-01*01*	*TCRBJ01-02*01*
CSALGQGNADEQFF	129	0.254462965	42	*TCRBV20*	*TCRBD01-01*01*	*TCRBJ02-01*01*
CASSQVGRTSTDTQYF	126	0.248545221	48	*TCRBV28-01*01*	*TCRBD02-01*02*	*TCRBJ02-03*01*
CATSVVLPYPQPPINEKLFF	126	0.248545221	60	*TCRBV24*	*TCRBD02-01*	*TCRBJ01-04*01*
CASSGLTRGTGELFF	124	0.244600059	45	*TCRBV06-01*01*	*TCRBD02-01*	*TCRBJ02-02*01*
CASSTPFNLGKTYGYTF	124	0.244600059	51	*TCRBV11-03*01*	*unresolved*	*TCRBJ01-02*01*
CASTGTGVTEAFF	124	0.244600059	39	*TCRBV28-01*01*	*TCRBD01-01*01*	*TCRBJ01-01*01*

CSATQGGNQPQHF	123	0.242627478	39	*TCRBV20*	*TCRBD01-01*01*	*TCRBJ01-05*01*
CASSFSGVEKLFF	122	0.240654897	39	*TCRBV12*	*TCRBD01-01*01*	*TCRBJ01-04*01*
CASSERSQNRKTYGYTF	121	0.238682316	51	*TCRBV04-01*01*	*TCRBD02-01*	*TCRBJ01-02*01*
CASSGRDGGSEQYF	121	0.238682316	42	*TCRBV19-01*	*TCRBD02-01*01*	*TCRBJ02-07*01*
CSGSGPNAQYF	121	0.238682316	33	*TCRBV20-01*01*	*unresolved*	*TCRBJ02-05*01*
CASSGGGNTEAFF	120	0.236709735	39	*TCRBV07-08*01*	*TCRBD01-01*01*	*TCRBJ01-01*01*
CASSQANQGLAETQYF	119	0.234737154	48	*TCRBV05-01*01*	*unresolved*	*TCRBJ02-05*01*
CASTDTGGRQYF	119	0.234737154	36	*TCRBV06-05*01*	*unresolved*	*TCRBJ02-07*01*
CASSQEWGRGYEQYF	118	0.232764572	45	*TCRBV04-02*01*	*TCRBD01-01*01*	*TCRBJ02-07*01*
CSVVRGLRGYEQYF	116	0.22881941	42	*TCRBV29-01*01*	*unresolved*	*TCRBJ02-07*01*
CASSLATSGEQFF	115	0.226846829	39	*TCRBV07-09*	*TCRBD02-01*	*TCRBJ02-01*01*
CASSPGPGQGLYEQYF	115	0.226846829	48	*TCRBV06-06*	*TCRBD01-01*01*	*TCRBJ02-07*01*
CASSSGQASWNTEAFF	115	0.226846829	48	*TCRBV06*	*TCRBD01-01*01*	*TCRBJ01-01*01*
CASSLNTGELFF	114	0.224874248	36	*TCRBV05-01*01*	*unresolved*	*TCRBJ02-02*01*
CATSDWGTDTQYF	112	0.220929086	39	*TCRBV24*	*unresolved*	*TCRBJ02-03*01*
CASSLVGQKGEKLFF	109	0.215011342	45	*TCRBV27-01*01*	*TCRBD01-01*01*	*TCRBJ01-04*01*
CASSLSDSGSRTDTQYF	108	0.213038761	51	*TCRBV11-02*02*	*TCRBD02-01*02*	*TCRBJ02-03*01*
CASSSSNHFIHHNSPLHF	108	0.213038761	54	*TCRBV03*	*TCRBD01-01*01*	*TCRBJ01-06*01*
CASSQDDRAAEAFF	107	0.21106618	42	*TCRBV04-01*01*	*TCRBD01-01*01*	*TCRBJ01-01*01*
CATRGEDNEQFF	107	0.21106618	36	*TCRBV12*	*TCRBD02-01*01*	*TCRBJ02-01*01*
CASSHRTSGGLVGNIQYF	106	0.209093599	54	*TCRBV03*	*TCRBD02-01*01*	*TCRBJ02-04*01*
CASSRTYHFVLDEQYF	106	0.209093599	48	*TCRBV03*	*unresolved*	*TCRBJ02-07*01*
CASSSPTTNVLTF	102	0.201203274	39	*TCRBV18-01*01*	*unresolved*	*TCRBJ02-06*01*
CASKFSYAYEQYF	101	0.199230693	39	*TCRBV02-01*01*	*TCRBD02-01*	*TCRBJ02-07*01*
CSASPMTGGQETQYF	101	0.199230693	45	*TCRBV20*	*TCRBD01-01*01*	*TCRBJ02-05*01*
CSALTSGRADEQFF	100	0.197258112	42	*TCRBV20*	*TCRBD02-01*02*	*TCRBJ02-01*01*
CASSSTGTNYGYTF	99	0.195285531	42	*TCRBV05-01*01*	*TCRBD01-01*01*	*TCRBJ01-02*01*
CASDVFRDRGLYGYTF	98	0.19331295	48	*TCRBV05-06*01*	*TCRBD01-01*01*	*TCRBJ01-02*01*
CASSDRIRYEQYF	97	0.191340369	39	*TCRBV02-01*01*	*unresolved*	*TCRBJ02-07*01*
CASSYSSGGGSGTGELFF	97	0.191340369	54	*TCRBV06*	*TCRBD02-01*01*	*TCRBJ02-02*01*
CASSQARGGSYNEQFF	96	0.189367788	48	*TCRBV03*	*TCRBD02-01*02*	*TCRBJ02-01*01*
CASTTEGTQETQYF	96	0.189367788	42	*TCRBV06-06*	*unresolved*	*TCRBJ02-05*01*

CASSRRRPIFGHHQPQHF	95	0.187395207	54	*TCRBV03*	*TCRBD01-01*01*	*TCRBJ01-05*01*
CSAEGPGGHYEQYF	95	0.187395207	42	*TCRBV20*	*TCRBD02-01*01*	*TCRBJ02-07*01*
CSVVPRGRSTEAFF	91	0.179504882	42	*TCRBV29-01*01*	*unresolved*	*TCRBJ01-01*01*
CSARDLLAGEMETQYF	88	0.173587139	48	*TCRBV20*	*TCRBD02-01*02*	*TCRBJ02-05*01*
CASSSKQVSNEQFF	87	0.171614558	42	*TCRBV12*	*TCRBD02-01*	*TCRBJ02-01*01*
CASSPPGEREGEKNIQYF	86	0.169641977	54	*TCRBV09-01*	*TCRBD02-01*02*	*TCRBJ02-04*01*
CASGFGGNQPQHF	85	0.167669395	39	*TCRBV12-05*01*	*TCRBD02-01*01*	*TCRBJ01-05*01*
CASSTGRFQETQYF	84	0.165696814	42	*TCRBV19-01*	*unresolved*	*TCRBJ02-05*01*
CASSYRTASSYNEQFF	82	0.161751652	48	*TCRBV06-05*01*	*TCRBD02-01*	*TCRBJ02-01*01*
CASSLGPAGGYEQYF	81	0.159779071	45	*TCRBV27-01*01*	*TCRBD02-01*02*	*TCRBJ02-07*01*
CASSTSYRENTEAFF	81	0.159779071	45	*TCRBV06-06*	*TCRBD01-01*01*	*TCRBJ01-01*01*
CSVDVAGGNEQFF	81	0.159779071	39	*TCRBV29-01*01*	*TCRBD02-01*01*	*TCRBJ02-01*01*
CASSLYDRESVLTF	80	0.15780649	42	*TCRBV27-01*01*	*TCRBD01-01*01*	*TCRBJ02-06*01*
CASSVNTGGSGYTF	79	0.155833909	42	*TCRBV09-01*	*TCRBD01-01*01*	*TCRBJ01-02*01*
CASSYTNTGELFF	79	0.155833909	39	*TCRBV06-05*01*	*unresolved*	*TCRBJ02-02*01*
CGTLLKITQSRANVLTF	79	0.155833909	51	*TCRBV08-02*01*	*TCRBD02-01*	*TCRBJ02-06*01*
CASGSSGQVVDEQFF	78	0.153861328	45	*TCRBV12-05*01*	*unresolved*	*TCRBJ02-01*01*
CASSFSIGDTRAFF	78	0.153861328	42	*TCRBV05-06*01*	*TCRBD01-01*01*	*TCRBJ01-01*01*
CASSLGGRNQPQHF	76	0.149916165	42	*TCRBV12*	*TCRBD01-01*01*	*TCRBJ01-05*01*
CAGITENYGYTF	73	0.143998422	36	*TCRBV30-01*01*	*TCRBD01-01*01*	*TCRBJ01-02*01*
CASSGASKCRKIYGYTF	72	0.142025841	51	*TCRBV07-07*01*	*TCRBD01-01*01*	*TCRBJ01-02*01*
CASSTLTSGANVLTF	72	0.142025841	45	*TCRBV06-05*01*	*unresolved*	*TCRBJ02-06*01*
CASSEIPTGRGENYGYTF	71	0.14005326	54	*TCRBV06-01*01*	*TCRBD01-01*01*	*TCRBJ01-02*01*
CASSVDPTSGVNNEQFF	71	0.14005326	51	*TCRBV09-01*	*TCRBD02-01*01*	*TCRBJ02-01*01*
CSATPGGNQPQHF	69	0.136108097	39	*TCRBV20*	*TCRBD01-01*01*	*TCRBJ01-05*01*
CSASLISGADTQYF	64	0.126245192	42	*TCRBV20*	*TCRBD02-01*	*TCRBJ02-03*01*
CSVRAAYNEQFF	64	0.126245192	36	*TCRBV29-01*01*	*unresolved*	*TCRBJ02-01*01*
CASSLSGAGTPYEQYF	62	0.12230003	48	*TCRBV27-01*01*	*unresolved*	*TCRBJ02-07*01*
CARSIGNTGELFF	60	0.118354867	39	*TCRBV12*	*TCRBD01-01*01*	*TCRBJ02-02*01*
CASSTAGTPLNEQFF	60	0.118354867	45	*TCRBV27-01*01*	*unresolved*	*TCRBJ02-01*01*
CASRRDRGPREQFF	59	0.116382286	42	*TCRBV02-01*01*	*TCRBD01-01*01*	*TCRBJ02-01*01*
CASSNAVATLHHQPQHF	59	0.116382286	51	*TCRBV21-01*01*	*TCRBD02-01*	*TCRBJ01-05*01*

CSVEDPGAEAFF	56	0.110464543	36	*TCRBV29-01*01*	*TCRBD01-01*01*	*TCRBJ01-01*01*
CAISEIGQTTTYNEQFF	54	0.106519381	51	*TCRBV10-03*01*	*TCRBD01-01*01*	*TCRBJ02-01*01*
CASSSSGRGTDTQYF	54	0.106519381	45	*TCRBV09-01*	*TCRBD02-01*02*	*TCRBJ02-03*01*
CASSPGPYTGELFF	53	0.104546799	42	*TCRBV05-06*01*	*unresolved*	*TCRBJ02-02*01*
CASSRDHRTDYGYTF	53	0.104546799	45	*TCRBV04-02*01*	*TCRBD01-01*01*	*TCRBJ01-02*01*
CASSDAGANVLTF	52	0.102574218	39	*TCRBV06-04*	*unresolved*	*TCRBJ02-06*01*
CASSDSDTGELFF	51	0.100601637	39	*TCRBV06-04*	*unresolved*	*TCRBJ02-02*01*
CGNGCKITQSRANVLTF	51	0.100601637	51	*TCRBV08-02*01*	*unresolved*	*TCRBJ02-06*01*
CASSLMAGRSTDTQYF	48	0.094683894	48	*TCRBV12*	*TCRBD02-01*01*	*TCRBJ02-03*01*
CSVFQDRGSSGELFF	47	0.092711313	45	*TCRBV29-01*01*	*TCRBD01-01*01*	*TCRBJ02-02*01*
CASSLTGGSTDTGELFF	46	0.090738732	51	*TCRBV18-01*01*	*TCRBD01-01*01*	*TCRBJ02-02*01*
CASSLNTEAFF	45	0.088766151	33	*TCRBV28-01*01*	*TCRBD02-01*	*TCRBJ01-01*01*
CASSQEIAGAAYNEQFF	45	0.088766151	51	*TCRBV04-02*01*	*TCRBD02-01*01*	*TCRBJ02-01*01*
CASSSDGAGTGTGELFF	41	0.080875826	51	*TCRBV05-01*01*	*TCRBD02-01*	*TCRBJ02-02*01*
CATSRDQGHSGMADTQYF	38	0.074958083	54	*TCRBV15-01*01*	*unresolved*	*TCRBJ02-03*01*
CSAHRGQETQYF	38	0.074958083	36	*TCRBV20*	*TCRBD01-01*01*	*TCRBJ02-05*01*
CRGAGGVDEQFF	36	0.07101292	36	*TCRBV20*	*TCRBD02-01*02*	*TCRBJ02-01*01*
CSVEGTGQADTQYF	36	0.07101292	42	*TCRBV29-01*01*	*unresolved*	*TCRBJ02-03*01*
CASSSLAGYNEQFF	32	0.063122596	42	*TCRBV27-01*01*	*TCRBD02-01*01*	*TCRBJ02-01*01*
CASTLSLRNSPLHF	31	0.061150015	42	*TCRBV28-01*01*	*TCRBD02-01*02*	*TCRBJ01-06*01*
CSVEGTPGTQYF	30	0.059177434	36	*TCRBV29-01*01*	*unresolved*	*TCRBJ02-05*01*
CASSLDSDQETQYF	29	0.057204853	42	*TCRBV05-04*01*	*unresolved*	*TCRBJ02-05*01*
CSASPGGNQPQHF	29	0.057204853	39	*TCRBV20*	*TCRBD01-01*01*	*TCRBJ01-05*01*
CASSYQGSETQYF	28	0.055232271	39	*TCRBV06-05*01*	*TCRBD01-01*01*	*TCRBJ02-05*01*
CASSQDRDSGANVLTF	27	0.05325969	48	*TCRBV04-02*01*	*TCRBD02-01*02*	*TCRBJ02-06*01*
CSARRGKTQYF	26	0.051287109	33	*TCRBV20-01*01*	*TCRBD01-01*01*	*TCRBJ02-05*01*
CASSLRKRGGLDTQYF	24	0.047341947	48	*TCRBV28-01*01*	*TCRBD01-01*01*	*TCRBJ02-03*01*
CASRPGQGADNEQFF	21	0.041424204	45	*TCRBV12*	*TCRBD01-01*01*	*TCRBJ02-01*01*
CASSLGSVVAPLHF	20	0.039451622	42	*TCRBV07-08*01*	*TCRBD02-01*	*TCRBJ01-06*01*
CSVAGRVSNEQFF	20	0.039451622	39	*TCRBV29-01*01*	*TCRBD01-01*01*	*TCRBJ02-01*01*
CAISPLDKALEVFF	19	0.037479041	42	*TCRBV10-03*01*	*TCRBD01-01*01*	*TCRBJ01-01*01*
CASSPDDTQYF	19	0.037479041	33	*TCRBV18-01*01*	*unresolved*	*TCRBJ02-03*01*

CASSQGGATDTQYF	19	0.037479041	42	*TCRBV03*	*unresolved*	*TCRBJ02-03*01*
CASSEVEKLGKTYGYTF	16	0.031561298	51	*TCRBV25-01*01*	*unresolved*	*TCRBJ01-02*01*
CASSQDFDSYFTGTQYF	15	0.029588717	51	*TCRBV04-01*01*	*TCRBD01-01*01*	*TCRBJ02-03*01*
CALAGVGKTQYF	14	0.027616136	36	*TCRBV30-01*01*	*TCRBD02-01*02*	*TCRBJ02-05*01*
CASSLEAGGGSDTQYF	12	0.023670973	48	*TCRBV05-04*01*	*TCRBD02-01*01*	*TCRBJ02-03*01*
CASSLYVATVMQDMQYF	11	0.021698392	51	*TCRBV21-01*01*	*TCRBD01-01*01*	*TCRBJ02-03*01*
CASSSTIATHSDNEQFF	11	0.021698392	51	*TCRBV21-01*01*	*TCRBD02-01*	*TCRBJ02-01*01*
CASSWTSGGYNEQFF	11	0.021698392	45	*TCRBV19-01*	*TCRBD02-01*01*	*TCRBJ02-01*01*
CASRTLAGVRTGELFF	10	0.019725811	48	*TCRBV06*	*TCRBD02-01*02*	*TCRBJ02-02*01*
CASSKRGFGGPRTKNIQYF	10	0.019725811	57	*TCRBV21-01*01*	*TCRBD02-01*01*	*TCRBJ02-04*01*
CAISEAESGAGANVLTF	6	0.011835487	51	*TCRBV10-03*01*	*unresolved*	*TCRBJ02-06*01*
CASSLTGGSTDAGELFF	6	0.011835487	51	*TCRBV18-01*01*	*TCRBD01-01*01*	*TCRBJ02-02*01*
CASSFGTDGYTF	5	0.009862906	36	*TCRBV05-04*01*	*unresolved*	*TCRBJ01-02*01*
CASSGTNPICMQDTQYF	5	0.009862906	51	*TCRBV03*	*TCRBD01-01*01*	*TCRBJ02-03*01*
CASSLMAGRGTDTQYF	5	0.009862906	48	*TCRBV12*	*TCRBD02-01*01*	*TCRBJ02-03*01*
CAGSIGNTGELFF	4	0.007890324	39	*TCRBV12*	*unresolved*	*TCRBJ02-02*01*
CSAKLSPLTSGSPHEQYF	4	0.007890324	54	*TCRBV20*	*TCRBD02-01*02*	*TCRBJ02-07*01*
CSARADRLIGQAFF	4	0.007890324	42	*TCRBV20*	*TCRBD01-01*01*	*TCRBJ01-01*01*
CASSFPSTDTQYF	3	0.005917743	39	*TCRBV11*	*TCRBD02-01*02*	*TCRBJ02-03*01*
CASSLEAGGGADTQYF	3	0.005917743	48	*TCRBV05-04*01*	*TCRBD02-01*01*	*TCRBJ02-03*01*
CASSQLGGEETGELFF	3	0.005917743	48	*TCRBV03*	*TCRBD02-01*01*	*TCRBJ02-02*01*
CACSQGGNSYEQYF	2	0.003945162	42	*TCRBV03*	*unresolved*	*TCRBJ02-07*01*
CASRASGGPTDTQYF	2	0.003945162	45	*TCRBV06-05*01*	*TCRBD01-01*01*	*TCRBJ02-03*01*
CASRDRGGSPLHF	2	0.003945162	39	*TCRBV02-01*01*	*TCRBD01-01*01*	*TCRBJ01-06*01*
CASSCIILATSRANVLTF	2	0.003945162	54	*TCRBV27-01*01*	*unresolved*	*TCRBJ02-06*01*
CASSKYLPFVMQDTQYF	2	0.003945162	51	*TCRBV03*	*unresolved*	*TCRBJ02-03*01*
CASSLCQRLAGGTYEQYF	2	0.003945162	54	*TCRBV27-01*01*	*TCRBD02-01*01*	*TCRBJ02-07*01*
CSADPRGLAARDEQFF	2	0.003945162	48	*TCRBV20*	*TCRBD02-01*	*TCRBJ02-01*01*
CSARFSGGPGTQYF	2	0.003945162	42	*TCRBV20*	*TCRBD02-01*02*	*TCRBJ02-05*01*
**LESION IV**						
CASSYNRGDRGGYEQYF	4206	3.277589888	51	*TCRBV18-01*01*	*TCRBD01-01*01*	*TCRBJ02-07*01*

CASSLSGTGVYEQYF	3641	2.837304989	45	*TCRBV12*	*TCRBD01-01*01*	*TCRBJ02-07*01*
CASNHAWVSNQPQHF	2600	2.026089803	45	*TCRBV23-01*01*	*unresolved*	*TCRBJ01-05*01*
CASSSGTNAYGYTF	1952	1.521125883	42	*TCRBV06-01*01*	*TCRBD01-01*01*	*TCRBJ01-02*01*
CASSIQEWSTEAFF	1419	1.105777473	42	*TCRBV19-01*	*unresolved*	*TCRBJ01-01*01*
CASSMAGYDYEQYF	1360	1.05980082	42	*TCRBV19-01*	*TCRBD02-01*01*	*TCRBJ02-07*01*
CASSSPNRVSGNTIYF	1261	0.982653554	48	*TCRBV07-09*	*TCRBD01-01*01*	*TCRBJ01-03*01*
CASSLFDRGGNEKLFF	1246	0.970964575	48	*TCRBV28-01*01*	*TCRBD02-01*02*	*TCRBJ01-04*01*
CASNPGTAYSYEQYF	1235	0.962392656	45	*TCRBV06-01*01*	*TCRBD01-01*01*	*TCRBJ02-07*01*
CASNRGQVEKLFF	1093	0.851736982	39	*TCRBV28-01*01*	*unresolved*	*TCRBJ01-04*01*
CASSPQDRGLRDGYTF	1073	0.836151676	48	*TCRBV27-01*01*	*TCRBD01-01*01*	*TCRBJ01-02*01*
CAIGDGAAGANVLTF	1069	0.833034615	45	*TCRBV10-03*01*	*unresolved*	*TCRBJ02-06*01*
CASSFGQAASPLHF	1061	0.826800492	42	*TCRBV12*	*unresolved*	*TCRBJ01-06*01*
CASSQSAATGNYEQYF	854	0.665492574	48	*TCRBV07-06*01*	*TCRBD01-01*01*	*TCRBJ02-07*01*
CASSFPSTDTQYF	848	0.660816982	39	*TCRBV05-06*01*	*TCRBD02-01*02*	*TCRBJ02-03*01*
CASSAQGGIGTIYEQYF	825	0.64289388	51	*TCRBV07-08*01*	*TCRBD01-01*01*	*TCRBJ02-07*01*
CAIGGGEGYGYTF	785	0.611723267	39	*TCRBV10-03*01*	*TCRBD01-01*01*	*TCRBJ01-02*01*
CSARESSHSGANVLTF	783	0.610164737	48	*TCRBV20*	*TCRBD02-01*	*TCRBJ02-06*01*
CASIQDNVEGITGELFF	769	0.599255022	51	*TCRBV12*	*TCRBD02-01*02*	*TCRBJ02-02*01*
CASRPPSDRWDYGYTF	758	0.590683104	48	*TCRBV28-01*01*	*unresolved*	*TCRBJ01-02*01*
CASSPQAGSPGNTIYF	628	0.489378614	48	*TCRBV28-01*01*	*TCRBD02-01*01*	*TCRBJ01-03*01*
CASSGTGWKNTIYF	622	0.484703022	42	*TCRBV02-01*01*	*TCRBD01-01*01*	*TCRBJ01-03*01*
CASSPPTADTQYF	571	0.444960491	39	*TCRBV28-01*01*	*unresolved*	*TCRBJ02-03*01*
CASSFGTLYSNQPQHF	569	0.443401961	48	*TCRBV06-05*01*	*unresolved*	*TCRBJ01-05*01*
CASSLARGSETQYF	564	0.439505634	42	*TCRBV05-01*01*	*unresolved*	*TCRBJ02-05*01*
CASSLSGTEGEQYF	562	0.437947103	42	*TCRBV28-01*01*	*TCRBD01-01*01*	*TCRBJ02-07*01*
CAISVGTSGRTDEQFF	556	0.433271512	48	*TCRBV10-03*01*	*TCRBD02-01*02*	*TCRBJ02-01*01*
CASSEMKGGRGGYTF	529	0.412231348	45	*TCRBV10-02*01*	*TCRBD02-01*01*	*TCRBJ01-02*01*
CASSLGQGLHEQYF	525	0.409114287	42	*TCRBV05-06*01*	*TCRBD01-01*01*	*TCRBJ02-07*01*
CAISDGHSNQPQHF	523	0.407555756	42	*TCRBV10-03*01*	*unresolved*	*TCRBJ01-05*01*
CSARDLTSNEQYF	523	0.407555756	39	*TCRBV20*	*TCRBD02-01*	*TCRBJ02-07*01*
CASSLMAGRSTDTQYF	512	0.398983838	48	*TCRBV12*	*TCRBD02-01*01*	*TCRBJ02-03*01*
CASSQVGRTSTDTQYF	500	0.389632654	48	*TCRBV28-01*01*	*TCRBD02-01*02*	*TCRBJ02-03*01*

CASSHRASGANVLTF	496	0.386515593	45	*TCRBV07-02*01*	*TCRBD01-01*01*	*TCRBJ02-06*01*
CASGEANSPLHF	488	0.380281471	36	*TCRBV23-01*01*	*unresolved*	*TCRBJ01-06*01*
CASRVSYEQYF	478	0.372488818	33	*TCRBV06*	*unresolved*	*TCRBJ02-07*01*
CASSVLEGTDTQYF	456	0.355344981	42	*TCRBV06-06*	*TCRBD02-01*02*	*TCRBJ02-03*01*
CASSSSAVAGRRVYEQYF	455	0.354565715	54	*TCRBV07-09*	*TCRBD02-01*01*	*TCRBJ02-07*01*
CASVNGQADTEAFF	450	0.350669389	42	*TCRBV28-01*01*	*TCRBD01-01*01*	*TCRBJ01-01*01*
CSAFSTTLNEQFF	450	0.350669389	39	*TCRBV20*	*unresolved*	*TCRBJ02-01*01*
CASSFSGNQPQHF	446	0.347552328	39	*TCRBV07-02*01*	*unresolved*	*TCRBJ01-05*01*
CASSVGPGSSYEQYF	440	0.342876736	45	*TCRBV09-01*	*TCRBD01-01*01*	*TCRBJ02-07*01*
CASSQIGSSGNTIYF	434	0.338201144	45	*TCRBV04-01*01*	*TCRBD02-01*	*TCRBJ01-03*01*
CASSYDSAGGYTF	423	0.329629226	39	*TCRBV05-05*01*	*TCRBD02-01*	*TCRBJ01-02*01*
CASSVGREGSNMANVLTF	412	0.321057307	54	*TCRBV09-01*	*unresolved*	*TCRBJ02-06*01*
CASRGTGGTYEQYF	411	0.320278042	42	*TCRBV11-02*02*	*TCRBD01-01*01*	*TCRBJ02-07*01*
CASSLALTDTQYF	402	0.313264654	39	*TCRBV07-08*01*	*unresolved*	*TCRBJ02-03*01*
CASSFSGRSDEQYF	401	0.312485389	42	*TCRBV19-01*	*TCRBD02-01*02*	*TCRBJ02-07*01*
CASSGNQGANGYTF	401	0.312485389	42	*TCRBV05-04*01*	*TCRBD01-01*01*	*TCRBJ01-02*01*
CASTTEGTQETQYF	393	0.306251266	42	*TCRBV06-06*	*unresolved*	*TCRBJ02-05*01*
CASSPSGDGYNEQFF	391	0.304692736	45	*TCRBV07-09*	*TCRBD02-01*01*	*TCRBJ02-01*01*
CASSRGTGTGPSYEQYF	388	0.30235494	51	*TCRBV19-01*	*TCRBD01-01*01*	*TCRBJ02-07*01*
CAISELLGYTEAFF	381	0.296900083	42	*TCRBV10-03*01*	*unresolved*	*TCRBJ01-01*01*
CASSPWYEQYF	381	0.296900083	33	*TCRBV28-01*01*	*unresolved*	*TCRBJ02-07*01*
CASSLGGQRAYEQYF	380	0.296120817	45	*TCRBV05-01*01*	*unresolved*	*TCRBJ02-07*01*
CASSPPSRGANYEQYF	376	0.293003756	48	*TCRBV07-09*	*TCRBD02-01*01*	*TCRBJ02-07*01*
CASSQDEGYNEKLFF	370	0.288328164	45	*TCRBV04-01*01*	*unresolved*	*TCRBJ01-04*01*
CASRGQGSAYNSPLHF	366	0.285211103	48	*TCRBV07-09*	*TCRBD01-01*01*	*TCRBJ01-06*01*
CASSIQRDYGYTF	363	0.282873307	39	*TCRBV19-01*	*unresolved*	*TCRBJ01-02*01*
CASSPKAQHSGYTF	360	0.280535511	42	*TCRBV19-01*	*unresolved*	*TCRBJ01-02*01*
CASSSLAYEQYF	358	0.278976981	36	*TCRBV06-06*	*TCRBD02-01*	*TCRBJ02-07*01*
CASRGSNYGYTF	357	0.278197715	36	*TCRBV06-05*01*	*unresolved*	*TCRBJ01-02*01*
CASSASGGVYEQYF	357	0.278197715	42	*TCRBV10-02*01*	*TCRBD02-01*01*	*TCRBJ02-07*01*
CASSLDWDQPQHF	357	0.278197715	39	*TCRBV07-09*	*unresolved*	*TCRBJ01-05*01*
CASSLGVGNSPLHF	354	0.275859919	42	*TCRBV28-01*01*	*unresolved*	*TCRBJ01-06*01*

CAIRGGQATFADTQYF	353	0.275080654	48	*TCRBV10-03*01*	*TCRBD01-01*01*	*TCRBJ02-03*01*
CASSGAEYGYTF	353	0.275080654	36	*TCRBV05-05*01*	*unresolved*	*TCRBJ01-02*01*
CASSQVQAIDTQYF	351	0.273522123	42	*TCRBV04-01*01*	*TCRBD02-01*	*TCRBJ02-03*01*
CATRPHDNQPQHF	351	0.273522123	39	*TCRBV24*	*unresolved*	*TCRBJ01-05*01*
CASRVENTGELFF	348	0.271184327	39	*TCRBV19-01*	*unresolved*	*TCRBJ02-02*01*
CASSPRPEGPEQYF	345	0.268846531	42	*TCRBV14-01*01*	*unresolved*	*TCRBJ02-07*01*
CASKFSYAYEQYF	341	0.26572947	39	*TCRBV02-01*01*	*TCRBD02-01*	*TCRBJ02-07*01*
CASSSRLDSTLNSPLHF	341	0.26572947	51	*TCRBV19-01*	*TCRBD01-01*01*	*TCRBJ01-06*01*
CASGLEDRISGNTIYF	337	0.262612409	48	*TCRBV12-05*01*	*TCRBD01-01*01*	*TCRBJ01-03*01*
CASSKAHVANTGELFF	336	0.261833144	48	*TCRBV21-01*01*	*unresolved*	*TCRBJ02-02*01*
CASGRTYNEQFF	332	0.258716082	36	*TCRBV10-01*	*TCRBD02-01*02*	*TCRBJ02-01*01*
CATNGGPLGNEQFF	332	0.258716082	42	*TCRBV06-06*	*unresolved*	*TCRBJ02-01*01*
CASSGTGGAEKLFF	327	0.254819756	42	*TCRBV06*	*TCRBD01-01*01*	*TCRBJ01-04*01*
CASSMGLGSYEQYF	322	0.250923429	42	*TCRBV19-01*	*TCRBD02-01*	*TCRBJ02-07*01*
CASTPPRGPQHF	319	0.248585633	36	*TCRBV28-01*01*	*TCRBD02-01*01*	*TCRBJ01-05*01*
CASPPYFYGTGPVNEQFF	318	0.247806368	54	*TCRBV19-01*	*TCRBD01-01*01*	*TCRBJ02-01*01*
CAWSVPAGNGANVLTF	318	0.247806368	48	*TCRBV30-01*01*	*TCRBD01-01*01*	*TCRBJ02-06*01*
CSARVQGLGNTIYF	316	0.246247838	42	*TCRBV20*	*TCRBD01-01*01*	*TCRBJ01-03*01*
CSVFQDRGSSGELFF	313	0.243910042	45	*TCRBV29-01*01*	*TCRBD01-01*01*	*TCRBJ02-02*01*
CASSFAKQGGDHEQYF	311	0.242351511	48	*TCRBV07-06*01*	*TCRBD01-01*01*	*TCRBJ02-07*01*
CASSLFSGEQYF	309	0.24079298	36	*TCRBV05-06*01*	*TCRBD02-01*	*TCRBJ02-07*01*
CASRTGYREQYF	308	0.240013715	36	*TCRBV06-05*01*	*TCRBD01-01*01*	*TCRBJ02-07*01*
CASLEGSPGSYSPLHF	306	0.238455184	48	*TCRBV06*	*TCRBD02-01*02*	*TCRBJ01-06*01*
CASSPRPGSGYEQYF	305	0.237675919	45	*TCRBV18-01*01*	*unresolved*	*TCRBJ02-07*01*
CASSQDRVTGYTF	301	0.234558858	39	*TCRBV19-01*	*TCRBD01-01*01*	*TCRBJ01-02*01*
CASSIEHVSYEQYF	299	0.233000327	42	*TCRBV19-01*	*unresolved*	*TCRBJ02-07*01*
CASSPQTGGGNTEAFF	298	0.232221062	48	*TCRBV28-01*01*	*TCRBD01-01*01*	*TCRBJ01-01*01*
CASSSGRQGAEKLFF	298	0.232221062	45	*TCRBV05-01*01*	*TCRBD01-01*01*	*TCRBJ01-04*01*
CATSRDRLGQPQHF	297	0.231441797	42	*TCRBV15-01*01*	*unresolved*	*TCRBJ01-05*01*
CASSGGNTEAFF	296	0.230662531	36	*TCRBV10-01*	*TCRBD02-01*02*	*TCRBJ01-01*01*
CASSWDRGAPYEQYF	295	0.229883266	45	*TCRBV19-01*	*TCRBD01-01*01*	*TCRBJ02-07*01*
CASSLGTGGRSEAFF	292	0.22754547	45	*TCRBV18-01*01*	*TCRBD01-01*01*	*TCRBJ01-01*01*

CASSLIRTVDEQYF	288	0.224428409	42	*TCRBV05-01*01*	*TCRBD01-01*01*	*TCRBJ02-07*01*
CASSRDQAVHEQYF	287	0.223649144	42	*TCRBV28-01*01*	*unresolved*	*TCRBJ02-07*01*
CASAAYNEQFF	285	0.222090613	33	*TCRBV12-05*01*	*TCRBD02-01*	*TCRBJ02-01*01*
CASSGLTRGTGELFF	284	0.221311348	45	*TCRBV06-01*01*	*TCRBD02-01*	*TCRBJ02-02*01*
CASSRTDSNQPQHF	284	0.221311348	42	*TCRBV10-02*01*	*TCRBD01-01*01*	*TCRBJ01-05*01*
CASSSAGGRSQPQHF	280	0.218194286	45	*TCRBV12*	*TCRBD01-01*01*	*TCRBJ01-05*01*
CASSGREGPGYTF	277	0.215856491	39	*TCRBV02-01*01*	*TCRBD02-01*02*	*TCRBJ01-02*01*
CASSLYGAGLYNEQFF	276	0.215077225	48	*TCRBV07-03*01*	*TCRBD02-01*	*TCRBJ02-01*01*
CASSRDGNYEQYF	276	0.215077225	39	*TCRBV19-01*	*unresolved*	*TCRBJ02-07*01*
CASSFQPLPPSPYGYTF	274	0.213518695	51	*TCRBV07-09*	*TCRBD02-01*	*TCRBJ01-02*01*
CASSLTGYNSPLHF	274	0.213518695	42	*TCRBV14-01*01*	*TCRBD01-01*01*	*TCRBJ01-06*01*
CASSTYGQGLYEQYF	269	0.209622368	45	*TCRBV05-04*01*	*TCRBD01-01*01*	*TCRBJ02-07*01*
CASSLSGGLGGTQYF	266	0.207284572	45	*TCRBV07-03*01*	*TCRBD02-01*01*	*TCRBJ02-05*01*
CASREAGGGDEQYF	265	0.206505307	42	*TCRBV06-05*01*	*TCRBD02-01*02*	*TCRBJ02-07*01*
CASSPSAISTGELFF	265	0.206505307	45	*TCRBV12*	*TCRBD02-01*	*TCRBJ02-02*01*
CASSFTPYGYTF	264	0.205726041	36	*TCRBV12*	*TCRBD02-01*	*TCRBJ01-02*01*
CSVEISKGTGNYGYTF	264	0.205726041	48	*TCRBV29-01*01*	*TCRBD01-01*01*	*TCRBJ01-02*01*
CASRRLAGNQETQYF	263	0.204946776	45	*TCRBV05-01*01*	*TCRBD02-01*01*	*TCRBJ02-05*01*
CASSPPGAGYGYTF	260	0.20260898	42	*TCRBV06-05*01*	*unresolved*	*TCRBJ01-02*01*
CASSLRQGYGYTF	256	0.199491919	39	*TCRBV05-01*01*	*TCRBD01-01*01*	*TCRBJ01-02*01*
CATPSRGENTIYF	256	0.199491919	39	*TCRBV07-03*01*	*TCRBD01-01*01*	*TCRBJ01-03*01*
CASSPNGGTNEKLFF	255	0.198712654	45	*TCRBV07-03*01*	*unresolved*	*TCRBJ01-04*01*
CASSQTPNYEQYF	255	0.198712654	39	*TCRBV03*	*TCRBD02-01*	*TCRBJ02-07*01*
CAISSIQGANYGYTF	254	0.197933388	45	*TCRBV10-03*01*	*TCRBD01-01*01*	*TCRBJ01-02*01*
CASSPPGTKNTGELFF	254	0.197933388	48	*TCRBV11-02*02*	*TCRBD02-01*	*TCRBJ02-02*01*
CASSSQQGPYEQYF	254	0.197933388	42	*TCRBV07-03*01*	*TCRBD01-01*01*	*TCRBJ02-07*01*
CASSPDDTQYF	253	0.197154123	33	*TCRBV18-01*01*	*unresolved*	*TCRBJ02-03*01*
CASSPYTGELFF	253	0.197154123	36	*TCRBV07-02*01*	*TCRBD02-01*	*TCRBJ02-02*01*
CASSRTRILSPYEQYF	253	0.197154123	48	*TCRBV19-01*	*TCRBD02-01*	*TCRBJ02-07*01*
CSASLISGADTQYF	252	0.196374858	42	*TCRBV20*	*TCRBD02-01*	*TCRBJ02-03*01*
CASSQGRSLSGNTIYF	251	0.195595592	48	*TCRBV03*	*TCRBD02-01*	*TCRBJ01-03*01*
CASSQVRLNDEQFF	251	0.195595592	42	*TCRBV04-01*01*	*TCRBD02-01*	*TCRBJ02-01*01*

CASSPSKVEEQYF	250	0.194816327	39	*TCRBV04-01*01*	*unresolved*	*TCRBJ02-07*01*
CASSIRPRGTVNYGYTF	249	0.194037062	51	*TCRBV19-01*	*TCRBD01-01*01*	*TCRBJ01-02*01*
CASRDTYRGQYGYTF	246	0.191699266	45	*TCRBV06-06*	*TCRBD01-01*01*	*TCRBJ01-02*01*
CASSLGLAGDTQYF	246	0.191699266	42	*TCRBV07-03*01*	*TCRBD02-01*	*TCRBJ02-03*01*
CASSPLGGLDNEKLFF	245	0.190920001	48	*TCRBV28-01*01*	*TCRBD02-01*	*TCRBJ01-04*01*
CATSELRQALSYEQYF	245	0.190920001	48	*TCRBV24*	*TCRBD01-01*01*	*TCRBJ02-07*01*
CASSHGQVRDQPQHF	244	0.190140735	45	*TCRBV28-01*01*	*TCRBD01-01*01*	*TCRBJ01-05*01*
CASSPVIRSTNEKLFF	244	0.190140735	48	*TCRBV11-02*02*	*unresolved*	*TCRBJ01-04*01*
CASSLITAGAYEQYF	243	0.18936147	45	*TCRBV28-01*01*	*TCRBD02-01*02*	*TCRBJ02-07*01*
CAITEGHSNTEAFF	241	0.187802939	42	*TCRBV10-03*01*	*TCRBD01-01*01*	*TCRBJ01-01*01*
CASGGRDRGHEQYF	240	0.187023674	42	*TCRBV12-05*01*	*TCRBD01-01*01*	*TCRBJ02-07*01*
CATSMGPSEKLFF	237	0.184685878	39	*TCRBV15-01*01*	*TCRBD01-01*01*	*TCRBJ01-04*01*
CASSLDRTGELFF	235	0.183127348	39	*TCRBV11-03*01*	*TCRBD02-01*	*TCRBJ02-02*01*
CASSLFRAGGYTF	235	0.183127348	39	*TCRBV11-02*02*	*TCRBD02-01*	*TCRBJ01-02*01*
CASSQYQGLTIYF	235	0.183127348	39	*TCRBV03*	*TCRBD01-01*01*	*TCRBJ01-03*01*
CASSVGGSYTF	235	0.183127348	33	*TCRBV05-06*01*	*TCRBD02-01*01*	*TCRBJ01-02*01*
CASSSGGGAAYEQYF	231	0.180010286	45	*TCRBV07-09*	*TCRBD02-01*01*	*TCRBJ02-07*01*
CASSLTGNGYTF	230	0.179231021	36	*TCRBV12*	*TCRBD02-01*01*	*TCRBJ01-02*01*
CASSLYRTITGELFF	229	0.178451756	45	*TCRBV05-01*01*	*TCRBD01-01*01*	*TCRBJ02-02*01*
CASSFTGSGYTF	227	0.176893225	36	*TCRBV12*	*unresolved*	*TCRBJ01-02*01*
CASSFHRGTGELFF	226	0.17611396	42	*TCRBV05-01*01*	*TCRBD02-01*01*	*TCRBJ02-02*01*
CASSLLYNEQFF	226	0.17611396	36	*TCRBV28-01*01*	*TCRBD02-01*	*TCRBJ02-01*01*
CASSRSGGPDGYTF	225	0.175334694	42	*TCRBV05-01*01*	*unresolved*	*TCRBJ01-02*01*
CASRDSLYEQYF	224	0.174555429	36	*TCRBV06-05*01*	*TCRBD01-01*01*	*TCRBJ02-07*01*
CASSFLRGPKRYNEQFF	224	0.174555429	51	*TCRBV12*	*unresolved*	*TCRBJ02-01*01*
CASSRTSGSSEQYF	224	0.174555429	42	*TCRBV05-01*01*	*TCRBD02-01*02*	*TCRBJ02-07*01*
CASSFGTGTPESMRNYGYTF	223	0.173776164	60	*TCRBV05-01*01*	*TCRBD01-01*01*	*TCRBJ01-02*01*
CASAAYSGRLSLNTEAFF	222	0.172996899	54	*TCRBV19-01*	*TCRBD02-01*02*	*TCRBJ01-01*01*
CASNTGTTNQPQHF	222	0.172996899	42	*TCRBV19-01*	*TCRBD02-01*	*TCRBJ01-05*01*
CASSFGSQPGEQYF	222	0.172996899	42	*TCRBV12*	*TCRBD02-01*	*TCRBJ02-07*01*
CASSLEGTSDNEQFF	221	0.172217633	45	*TCRBV05-01*01*	*TCRBD02-01*	*TCRBJ02-01*01*
CASSLGTVSEQYF	221	0.172217633	39	*TCRBV05-01*01*	*TCRBD01-01*01*	*TCRBJ02-07*01*

CASSYFHLLAGVNEQFF	220	0.171438368	51	*TCRBV06*	*TCRBD02-01*01*	*TCRBJ02-01*01*
CASSEYGTSGYTF	218	0.169879837	39	*TCRBV25-01*01*	*TCRBD01-01*01*	*TCRBJ01-02*01*
CASSNRGSYDTEAFF	218	0.169879837	45	*TCRBV19-01*	*TCRBD01-01*01*	*TCRBJ01-01*01*
CASSQGTGGIGNSPLHF	218	0.169879837	51	*TCRBV06*	*TCRBD01-01*01*	*TCRBJ01-06*01*
CSARDPVRYEQYF	218	0.169879837	39	*TCRBV20*	*TCRBD02-01*	*TCRBJ02-07*01*
CAISDSGTSNEQFF	217	0.169100572	42	*TCRBV10-03*01*	*TCRBD02-01*	*TCRBJ02-01*01*
CASSPRTGEIRELFF	217	0.169100572	45	*TCRBV05-01*01*	*TCRBD01-01*01*	*TCRBJ02-02*01*
CASSDSPGRGSNQPQHF	216	0.168321307	51	*TCRBV09-01*	*TCRBD01-01*01*	*TCRBJ01-05*01*
CASSLAGNGYTF	215	0.167542041	36	*TCRBV11-02*02*	*TCRBD02-01*02*	*TCRBJ01-02*01*
CASSLSIAGGVTDTQYF	215	0.167542041	51	*TCRBV06*	*TCRBD02-01*02*	*TCRBJ02-03*01*
CASSTPTAGTSTVSSYNEQFF	210	0.163645715	63	*TCRBV19-01*	*TCRBD01-01*01*	*TCRBJ02-01*01*
CASSGGHQSSYEQYF	209	0.16286645	45	*TCRBV05-01*01*	*TCRBD01-01*01*	*TCRBJ02-07*01*
CASSRTRTGAYEQYF	208	0.162087184	45	*TCRBV11-01*01*	*TCRBD02-01*01*	*TCRBJ02-07*01*
CASSSGTAYGYTF	208	0.162087184	39	*TCRBV06-05*01*	*unresolved*	*TCRBJ01-02*01*
CASSWTISSTDTQYF	207	0.161307919	45	*TCRBV06-04*	*TCRBD02-01*	*TCRBJ02-03*01*
CASSYTPRAGELFF	207	0.161307919	42	*TCRBV06-05*01*	*TCRBD02-01*	*TCRBJ02-02*01*
CASGDYEQYF	206	0.160528654	30	*TCRBV28-01*01*	*unresolved*	*TCRBJ02-07*01*
CASRRVHQPQHF	205	0.159749388	36	*TCRBV12-02*01*	*unresolved*	*TCRBJ01-05*01*
CASSTRGGQAEAFF	205	0.159749388	42	*TCRBV19-01*	*TCRBD01-01*01*	*TCRBJ01-01*01*
CASKGQATNEKLFF	204	0.158970123	42	*TCRBV06-05*01*	*TCRBD01-01*01*	*TCRBJ01-04*01*
CASRRGAVETQYF	203	0.158190858	39	*TCRBV06-05*01*	*unresolved*	*TCRBJ02-05*01*
CASSDRGNTEAFF	203	0.158190858	39	*TCRBV10-02*01*	*TCRBD01-01*01*	*TCRBJ01-01*01*
CASSQGVVGTDTQYF	202	0.157411592	45	*TCRBV04-01*01*	*TCRBD02-01*	*TCRBJ02-03*01*
CASSRGQNTDTQYF	201	0.156632327	42	*TCRBV05-01*01*	*unresolved*	*TCRBJ02-03*01*
CSAGPSPGLETQYF	201	0.156632327	42	*TCRBV20*	*TCRBD01-01*01*	*TCRBJ02-05*01*
CASSPQRAGSYEQYF	200	0.155853062	45	*TCRBV07-08*01*	*TCRBD02-01*	*TCRBJ02-07*01*
CASSPKVATPSDNEQFF	199	0.155073796	51	*TCRBV21-01*01*	*TCRBD02-01*	*TCRBJ02-01*01*
CASSPVKIVRKTYGYTF	199	0.155073796	51	*TCRBV16-01*	*unresolved*	*TCRBJ01-02*01*
CASSFAARNNEQFF	198	0.154294531	42	*TCRBV28-01*01*	*TCRBD02-01*	*TCRBJ02-01*01*
CASSSTPTNEKLFF	198	0.154294531	42	*TCRBV05-06*01*	*TCRBD02-01*	*TCRBJ01-04*01*
CASRLDDDGGARGNTIYF	197	0.153515266	54	*TCRBV27-01*01*	*unresolved*	*TCRBJ01-03*01*
CASSDMGTALRSGYTF	197	0.153515266	48	*TCRBV06-01*01*	*TCRBD01-01*01*	*TCRBJ01-02*01*

CASSLGYGYTF	197	0.153515266	33	*TCRBV07-08*01*	*unresolved*	*TCRBJ01-02*01*
CATSRESGGAKDTQYF	197	0.153515266	48	*TCRBV15-01*01*	*TCRBD02-01*01*	*TCRBJ02-03*01*
CASSYRSSSGANVLTF	196	0.152736	48	*TCRBV06-05*01*	*TCRBD02-01*02*	*TCRBJ02-06*01*
CASRLGNSRKNYGYTF	195	0.151956735	48	*TCRBV05-06*01*	*unresolved*	*TCRBJ01-02*01*
CATSREIYNEQFF	195	0.151956735	39	*TCRBV15-01*01*	*unresolved*	*TCRBJ02-01*01*
CASSLGWAGGTETQYF	194	0.15117747	48	*TCRBV07-09*	*TCRBD01-01*01*	*TCRBJ02-05*01*
CATSLASPGEKLFF	194	0.15117747	42	*TCRBV15-01*01*	*unresolved*	*TCRBJ01-04*01*
CSAPEDLAKNIQYF	192	0.149618939	42	*TCRBV20*	*unresolved*	*TCRBJ02-04*01*
CASSQDLVRRIEF	191	0.148839674	39	*TCRBV14-01*01*	*TCRBD02-01*	*TCRBJ02-07*01*
CASSQSWGQGPRETQYF	190	0.148060409	51	*TCRBV16-01*	*TCRBD01-01*01*	*TCRBJ02-05*01*
CASSYSSNEQFF	190	0.148060409	36	*TCRBV06-06*	*unresolved*	*TCRBJ02-01*01*
CASIHPSGAGNTIYF	189	0.147281143	45	*TCRBV19-01*	*TCRBD02-01*	*TCRBJ01-03*01*
CASSFIPGVDTEAFF	188	0.146501878	45	*TCRBV28-01*01*	*TCRBD02-01*01*	*TCRBJ01-01*01*
CASSSLGRVGEKLFF	187	0.145722613	45	*TCRBV07-02*01*	*TCRBD01-01*01*	*TCRBJ01-04*01*
CASSLGPAGGYEQYF	185	0.144164082	45	*TCRBV27-01*01*	*TCRBD02-01*02*	*TCRBJ02-07*01*
CASSLTQIQGSSYEQYF	185	0.144164082	51	*TCRBV27-01*01*	*TCRBD01-01*01*	*TCRBJ02-07*01*
CASSQTSGGSYNEQFF	185	0.144164082	48	*TCRBV04-02*01*	*TCRBD02-01*01*	*TCRBJ02-01*01*
CASSGHGGAGADTQYF	184	0.143384817	48	*TCRBV10-02*01*	*TCRBD02-01*01*	*TCRBJ02-03*01*
CASSPFPGLAGGVGWETQYF	182	0.141826286	60	*TCRBV18-01*01*	*TCRBD02-01*02*	*TCRBJ02-05*01*
CASSPKDEQYF	182	0.141826286	33	*TCRBV06-01*01*	*unresolved*	*TCRBJ02-07*01*
CASSFRGSGYEQYF	181	0.141047021	42	*TCRBV28-01*01*	*TCRBD02-01*02*	*TCRBJ02-07*01*
CASSLQGVDQPQHF	180	0.140267756	42	*TCRBV10-02*01*	*TCRBD01-01*01*	*TCRBJ01-05*01*
CASSWFGGYEQYF	180	0.140267756	39	*TCRBV27-01*01*	*TCRBD02-01*01*	*TCRBJ02-07*01*
CASSDRIRYEQYF	179	0.13948849	39	*TCRBV02-01*01*	*unresolved*	*TCRBJ02-07*01*
CASSPWDRGGETQYF	179	0.13948849	45	*TCRBV06-05*01*	*TCRBD01-01*01*	*TCRBJ02-05*01*
CASSQLPPGQGDSPLHF	179	0.13948849	51	*TCRBV18-01*01*	*TCRBD01-01*01*	*TCRBJ01-06*01*
CASSRTSNEKLFF	178	0.138709225	39	*TCRBV05-01*01*	*unresolved*	*TCRBJ01-04*01*
CASSVGGLASDTQYF	177	0.13792996	45	*TCRBV09-01*	*TCRBD02-01*	*TCRBJ02-03*01*
CASSAQGRVIADTQYF	176	0.137150694	48	*TCRBV19-01*	*TCRBD02-01*02*	*TCRBJ02-03*01*
CASSHRTSGGLVGNIQYF	176	0.137150694	54	*TCRBV03*	*TCRBD02-01*01*	*TCRBJ02-04*01*
CASSPGQSSYEQYF	176	0.137150694	42	*TCRBV28-01*01*	*TCRBD01-01*01*	*TCRBJ02-07*01*
CASSSGLNTEAFF	176	0.137150694	39	*TCRBV14-01*01*	*unresolved*	*TCRBJ01-01*01*

CSAGTSGTETQYF	176	0.137150694	39	*TCRBV20*	*TCRBD02-01*02*	*TCRBJ02-05*01*
CASSSTQGAYNQPQHF	175	0.136371429	48	*TCRBV06*	*TCRBD01-01*01*	*TCRBJ01-05*01*
CASSYIPRGPIPDPQHF	175	0.136371429	51	*TCRBV06*	*unresolved*	*TCRBJ01-05*01*
CSADASGGARETQYF	175	0.136371429	45	*TCRBV20*	*TCRBD02-01*01*	*TCRBJ02-05*01*
CASSATSAGYTDTQYF	174	0.135592164	48	*TCRBV09-01*	*TCRBD02-01*01*	*TCRBJ02-03*01*
CASSKTSGSSYNEQFF	174	0.135592164	48	*TCRBV02-01*01*	*unresolved*	*TCRBJ02-01*01*
CASSLSGQGYEQYF	174	0.135592164	42	*TCRBV05-01*01*	*TCRBD01-01*01*	*TCRBJ02-07*01*
CASSLSTGRSAYQPQHF	174	0.135592164	51	*TCRBV28-01*01*	*unresolved*	*TCRBJ01-05*01*
CASAPLGRLSTDTQYF	173	0.134812898	48	*TCRBV12*	*unresolved*	*TCRBJ02-03*01*
CASSGARRSSYEQYF	173	0.134812898	45	*TCRBV07-08*01*	*TCRBD02-01*	*TCRBJ02-07*01*
CASSPMGEYQPQHF	172	0.134033633	42	*TCRBV12*	*unresolved*	*TCRBJ01-05*01*
CASSSNMGYTF	172	0.134033633	33	*TCRBV05-06*01*	*unresolved*	*TCRBJ01-02*01*
CASSETSGNAGANVLTF	171	0.133254368	51	*TCRBV10-02*01*	*TCRBD02-01*02*	*TCRBJ02-06*01*
CASSVGAITDTQYF	171	0.133254368	42	*TCRBV02-01*01*	*TCRBD01-01*01*	*TCRBJ02-03*01*
CASSSGGDPQETQYF	170	0.132475102	45	*TCRBV10-01*	*TCRBD01-01*01*	*TCRBJ02-05*01*
CASRNYGGNYGYTF	169	0.131695837	42	*TCRBV03*	*unresolved*	*TCRBJ01-02*01*
CASRVQNYEQYF	169	0.131695837	36	*TCRBV06-06*	*TCRBD01-01*01*	*TCRBJ02-07*01*
CASRDPFSRKTYGYTF	168	0.130916572	48	*TCRBV05-05*01*	*unresolved*	*TCRBJ01-02*01*
CASSLGTSGGTDTQYF	168	0.130916572	48	*TCRBV05-04*01*	*TCRBD02-01*01*	*TCRBJ02-03*01*
CASSPRRVPQSRANVLTF	168	0.130916572	54	*TCRBV07-07*01*	*unresolved*	*TCRBJ02-06*01*
CASSPSQAYEQYF	168	0.130916572	39	*TCRBV18-01*01*	*TCRBD01-01*01*	*TCRBJ02-07*01*
CASSLSTPRDSHEQYF	167	0.130137307	48	*TCRBV28-01*01*	*TCRBD01-01*01*	*TCRBJ02-07*01*
CASSLGTGSGDTQYF	166	0.129358041	45	*TCRBV07-09*	*TCRBD01-01*01*	*TCRBJ02-03*01*
CASSLTSRTSGQGNEQFF	166	0.129358041	54	*TCRBV07-03*01*	*TCRBD02-01*	*TCRBJ02-01*01*
CASSQAFGVPGELFF	166	0.129358041	45	*TCRBV04-02*01*	*unresolved*	*TCRBJ02-02*01*
CASSFAGNQPQHF	165	0.128578776	39	*TCRBV06-05*01*	*unresolved*	*TCRBJ01-05*01*
CASSFVRGYGYTF	165	0.128578776	39	*TCRBV27-01*01*	*TCRBD01-01*01*	*TCRBJ01-02*01*
CSAREKREPREQFF	165	0.128578776	42	*TCRBV20*	*TCRBD02-01*02*	*TCRBJ02-01*01*
CASSGGGNTEAFF	164	0.127799511	39	*TCRBV07-08*01*	*TCRBD01-01*01*	*TCRBJ01-01*01*
CASSPLPASSYNSPLHF	164	0.127799511	51	*TCRBV18-01*01*	*TCRBD02-01*	*TCRBJ01-06*01*
CASSPRDPIFVLDEQYF	164	0.127799511	51	*TCRBV03*	*unresolved*	*TCRBJ02-07*01*
CASSSGFANYGYTF	164	0.127799511	42	*TCRBV05-01*01*	*TCRBD01-01*01*	*TCRBJ01-02*01*

CSARDRLSSYNSPLHF	164	0.127799511	48	*TCRBV20*	*TCRBD01-01*01*	*TCRBJ01-06*01*
CASSEMTGGRGGYTF	163	0.127020245	45	*TCRBV10-02*01*	*TCRBD02-01*01*	*TCRBJ01-02*01*
CASSRGDRGRNTEAFF	163	0.127020245	48	*TCRBV05-01*01*	*TCRBD01-01*01*	*TCRBJ01-01*01*
CASSLGLSGEQFF	162	0.12624098	39	*TCRBV07-02*01*	*TCRBD02-01*02*	*TCRBJ02-01*01*
CASSQGPGTVQETQYF	161	0.125461715	48	*TCRBV14-01*01*	*unresolved*	*TCRBJ02-05*01*
CASSLNPGGRNTGELFF	160	0.124682449	51	*TCRBV05-01*01*	*TCRBD02-01*01*	*TCRBJ02-02*01*
CASRAETSTQSRANVLTF	159	0.123903184	54	*TCRBV12-02*01*	*unresolved*	*TCRBJ02-06*01*
CASSFTGAESNQPQHF	159	0.123903184	48	*TCRBV05-06*01*	*TCRBD01-01*01*	*TCRBJ01-05*01*
CASSLGINTEAFF	159	0.123903184	39	*TCRBV04-01*01*	*unresolved*	*TCRBJ01-01*01*
CSAFAVRGGITDTQYF	159	0.123903184	48	*TCRBV20*	*TCRBD02-01*01*	*TCRBJ02-03*01*
CASSIGQWDNTEAFF	158	0.123123919	45	*TCRBV19-01*	*unresolved*	*TCRBJ01-01*01*
CATEAGGANTGELFF	158	0.123123919	45	*TCRBV28-01*01*	*TCRBD02-01*01*	*TCRBJ02-02*01*
CASRHSGGRNTGELFF	157	0.122344653	48	*TCRBV28-01*01*	*TCRBD02-01*01*	*TCRBJ02-02*01*
CASSEKVTPAYNSPLHF	157	0.122344653	51	*TCRBV06-01*01*	*unresolved*	*TCRBJ01-06*01*
CASRASDRNEQYF	155	0.120786123	39	*TCRBV14-01*01*	*unresolved*	*TCRBJ02-07*01*
CASSGLANTGELFF	155	0.120786123	42	*TCRBV05-05*01*	*TCRBD02-01*	*TCRBJ02-02*01*
CASSPRGQGYYEQYF	154	0.120006858	45	*TCRBV13-01*01*	*TCRBD01-01*01*	*TCRBJ02-07*01*
CASSVTLEVSGGYTF	154	0.120006858	45	*TCRBV06-01*01*	*unresolved*	*TCRBJ01-02*01*
CASSLELAANTGELFF	153	0.119227592	48	*TCRBV05-04*01*	*TCRBD02-01*	*TCRBJ02-02*01*
CASSFDSAIREYF	152	0.118448327	39	*TCRBV12*	*TCRBD01-01*01*	*TCRBJ02-03*01*
CASSHPAGQGEETQYF	151	0.117669062	48	*TCRBV07-09*	*TCRBD01-01*01*	*TCRBJ02-05*01*
CASSLEGRGYEQYF	151	0.117669062	42	*TCRBV05-01*01*	*TCRBD01-01*01*	*TCRBJ02-07*01*
CASSLGTSGDEQYF	151	0.117669062	42	*TCRBV05-01*01*	*TCRBD02-01*01*	*TCRBJ02-07*01*
CASSPVPGTTYYEQYF	151	0.117669062	48	*TCRBV27-01*01*	*unresolved*	*TCRBJ02-07*01*
CASSLAHSASETQYF	149	0.116110531	45	*TCRBV05-01*01*	*unresolved*	*TCRBJ02-05*01*
CASSPHPSARLDRVDRSHGYTF	149	0.116110531	66	*TCRBV04-02*01*	*TCRBD01-01*01*	*TCRBJ01-02*01*
CASSRTGGQGIAYEQYF	149	0.116110531	51	*TCRBV11-02*02*	*TCRBD01-01*01*	*TCRBJ02-07*01*
CASSGNVLGEKLFF	148	0.115331266	42	*TCRBV02-01*01*	*unresolved*	*TCRBJ01-04*01*
CASSQAGAHFFNQPQHF	148	0.115331266	51	*TCRBV04-01*01*	*TCRBD01-01*01*	*TCRBJ01-05*01*
CASSSEGSGYTF	148	0.115331266	36	*TCRBV13-01*01*	*TCRBD02-01*02*	*TCRBJ01-02*01*
CASSYSGLGSYEQYF	148	0.115331266	45	*TCRBV06*	*unresolved*	*TCRBJ02-07*01*
CASTIGGGRHGYTF	148	0.115331266	42	*TCRBV12*	*TCRBD02-01*01*	*TCRBJ01-02*01*

CASSLGGESDTQYF	147	0.114552	42	*TCRBV12*	*TCRBD02-01*02*	*TCRBJ02-03*01*
CASSLWGQGLYNEQFF	147	0.114552	48	*TCRBV28-01*01*	*TCRBD01-01*01*	*TCRBJ02-01*01*
CATATGGGNTIYF	147	0.114552	39	*TCRBV15-01*01*	*TCRBD01-01*01*	*TCRBJ01-03*01*
CSASGAGGREQYF	147	0.114552	39	*TCRBV20*	*TCRBD02-01*01*	*TCRBJ02-07*01*
CASSLGGGADGLNQPQHF	146	0.113772735	54	*TCRBV05-01*01*	*TCRBD02-01*02*	*TCRBJ01-05*01*
CASSNSGSGNEQFF	146	0.113772735	42	*TCRBV11-03*01*	*TCRBD02-01*02*	*TCRBJ02-01*01*
CASTPTQGITGELFF	146	0.113772735	45	*TCRBV03*	*TCRBD01-01*01*	*TCRBJ02-02*01*
CASRSGIQPQHF	145	0.11299347	36	*TCRBV06*	*TCRBD01-01*01*	*TCRBJ01-05*01*
CASSSGNGEQYF	145	0.11299347	36	*TCRBV07-09*	*TCRBD02-01*	*TCRBJ02-07*01*
CASTVAGVNGYTF	145	0.11299347	39	*TCRBV28-01*01*	*TCRBD02-01*01*	*TCRBJ01-02*01*
CASSQDLVTEAFF	144	0.112214204	39	*TCRBV14-01*01*	*TCRBD02-01*	*TCRBJ01-01*01*
CASSVWGGLADYNEQFF	144	0.112214204	51	*TCRBV06-01*01*	*TCRBD02-01*	*TCRBJ02-01*01*
CSARGWAAGRSSYEQYF	143	0.111434939	51	*TCRBV20*	*TCRBD02-01*02*	*TCRBJ02-07*01*
CSASSGQGYNQPQHF	143	0.111434939	45	*TCRBV20*	*TCRBD01-01*01*	*TCRBJ01-05*01*
CASSLYASESYEQYF	142	0.110655674	45	*TCRBV27-01*01*	*TCRBD02-01*	*TCRBJ02-07*01*
CASSVGNSISGNTIYF	142	0.110655674	48	*TCRBV02-01*01*	*TCRBD01-01*01*	*TCRBJ01-03*01*
CASSVGQMNYGYTF	142	0.110655674	42	*TCRBV09-01*	*TCRBD01-01*01*	*TCRBJ01-02*01*
CARATGGVKEQYF	141	0.109876409	39	*TCRBV06-05*01*	*TCRBD01-01*01*	*TCRBJ02-07*01*
CASGPEGLATYNEQFF	141	0.109876409	48	*TCRBV12-05*01*	*TCRBD02-01*	*TCRBJ02-01*01*
CASSQVSGWDNEQFF	141	0.109876409	45	*TCRBV03*	*TCRBD01-01*01*	*TCRBJ02-01*01*
CASSPSWGIGELFF	140	0.109097143	42	*TCRBV09-01*	*unresolved*	*TCRBJ02-02*01*
CASSQSSGRLRNEQFF	139	0.108317878	48	*TCRBV03*	*TCRBD02-01*02*	*TCRBJ02-01*01*
CASSRDGAAGYTF	139	0.108317878	39	*TCRBV06-05*01*	*unresolved*	*TCRBJ01-02*01*
CASTMGPPNTGELFF	139	0.108317878	45	*TCRBV05-01*01*	*unresolved*	*TCRBJ02-02*01*
CASSLVGSGNTIYF	138	0.107538613	42	*TCRBV06-01*01*	*TCRBD02-01*	*TCRBJ01-03*01*
CASSYTNTGELFF	138	0.107538613	39	*TCRBV06-05*01*	*unresolved*	*TCRBJ02-02*01*
CASSERTGGANTQYF	136	0.105980082	45	*TCRBV02-01*01*	*TCRBD01-01*01*	*TCRBJ02-03*01*
CASSLAGGPGSEQYF	136	0.105980082	45	*TCRBV07-08*01*	*TCRBD02-01*01*	*TCRBJ02-07*01*
CSAQGLAGGHVDEQFF	136	0.105980082	48	*TCRBV20*	*TCRBD02-01*01*	*TCRBJ02-01*01*
CASGGRRDPPYEQYF	135	0.105200817	45	*TCRBV07-09*	*unresolved*	*TCRBJ02-07*01*
CASSAGTGYGYTF	135	0.105200817	39	*TCRBV09-01*	*TCRBD01-01*01*	*TCRBJ01-02*01*
CASSPTQGIGTDTQYF	135	0.105200817	48	*TCRBV07-06*01*	*TCRBD01-01*01*	*TCRBJ02-03*01*

CASRQGGGSPLHF	134	0.104421551	39	*TCRBV06-01*01*	*TCRBD01-01*01*	*TCRBJ01-06*01*
CASSEASGRGTTYEQYF	134	0.104421551	51	*TCRBV02-01*01*	*TCRBD01-01*01*	*TCRBJ02-07*01*
CASSSVLDSYEQYF	134	0.104421551	42	*TCRBV05-01*01*	*TCRBD02-01*	*TCRBJ02-07*01*
CSALGQGNADEQFF	134	0.104421551	42	*TCRBV20*	*TCRBD01-01*01*	*TCRBJ02-01*01*
CASRGGNYGYTF	133	0.103642286	36	*TCRBV06-05*01*	*TCRBD02-01*01*	*TCRBJ01-02*01*
CASSLSGGQAFF	133	0.103642286	36	*TCRBV12*	*TCRBD02-01*02*	*TCRBJ01-01*01*
CASSTVVATQGLTGELFF	133	0.103642286	54	*TCRBV21-01*01*	*TCRBD01-01*01*	*TCRBJ02-02*01*
CSATGGGNQPQHF	133	0.103642286	39	*TCRBV20*	*TCRBD02-01*01*	*TCRBJ01-05*01*
CASFSGSQSRANVLTF	132	0.102863021	48	*TCRBV05-01*01*	*unresolved*	*TCRBJ02-06*01*
CASSLWTSGKHTQYF	132	0.102863021	45	*TCRBV03*	*TCRBD02-01*02*	*TCRBJ02-03*01*
CASSRGTGGGNEQFF	132	0.102863021	45	*TCRBV05-01*01*	*TCRBD01-01*01*	*TCRBJ02-01*01*
CASRPRIGYTIRTEAFF	131	0.102083755	51	*TCRBV21-01*01*	*unresolved*	*TCRBJ01-01*01*
CASSGQGGSGNTIYF	131	0.102083755	45	*TCRBV25-01*01*	*TCRBD01-01*01*	*TCRBJ01-03*01*
CASSRYNEQFF	131	0.102083755	33	*TCRBV11-02*02*	*unresolved*	*TCRBJ02-01*01*
CATSRAGLAGEGEQFF	131	0.102083755	48	*TCRBV15-01*01*	*TCRBD02-01*02*	*TCRBJ02-01*01*
CASINRSSGRFWSETQYF	130	0.10130449	54	*TCRBV28-01*01*	*TCRBD02-01*02*	*TCRBJ02-05*01*
CASSHTTKSGGFSNQPQHF	130	0.10130449	57	*TCRBV06*	*TCRBD01-01*01*	*TCRBJ01-05*01*
CASSLAWVGNYGYTF	130	0.10130449	45	*TCRBV07-02*01*	*unresolved*	*TCRBJ01-02*01*
CASSSFVDYRKLFF	129	0.100525225	42	*TCRBV07-08*01*	*TCRBD02-01*	*TCRBJ01-04*01*
CASSGTAQGDTQYF	128	0.09974596	42	*TCRBV23-01*01*	*TCRBD02-01*	*TCRBJ02-03*01*
CASSPDRVVGYTF	128	0.09974596	39	*TCRBV19-01*	*unresolved*	*TCRBJ01-02*01*
CASSPTNTEAFF	128	0.09974596	36	*TCRBV10-02*01*	*unresolved*	*TCRBJ01-01*01*
CASSLGGTRTRGSYEQYF	127	0.098966694	54	*TCRBV05-01*01*	*unresolved*	*TCRBJ02-07*01*
CASSNPRDRDMAGELFF	127	0.098966694	51	*TCRBV07-09*	*TCRBD01-01*01*	*TCRBJ02-02*01*
CASSQGGNKAGELFF	127	0.098966694	45	*TCRBV14-01*01*	*unresolved*	*TCRBJ02-02*01*
CASSSSNYEQYF	127	0.098966694	36	*TCRBV07-08*01*	*unresolved*	*TCRBJ02-07*01*
CASSWDRLNDEQYF	127	0.098966694	42	*TCRBV02-01*01*	*TCRBD01-01*01*	*TCRBJ02-07*01*
CATLGGAGRSYNEQFF	127	0.098966694	48	*TCRBV19-01*	*TCRBD02-01*02*	*TCRBJ02-01*01*
CASRGQGSLGNSPLHF	126	0.098187429	48	*TCRBV07-09*	*TCRBD01-01*01*	*TCRBJ01-06*01*
CASRSSITRQYF	126	0.098187429	36	*TCRBV28-01*01*	*TCRBD02-01*	*TCRBJ02-07*01*
CASSFYGGRGADTQYF	126	0.098187429	48	*TCRBV07-02*01*	*TCRBD02-01*02*	*TCRBJ02-03*01*
CASSQVGLSLTDTQYF	126	0.098187429	48	*TCRBV03*	*TCRBD02-01*	*TCRBJ02-03*01*

CASSARTGSPLHF	125	0.097408164	39	*TCRBV06-01*01*	*TCRBD01-01*01*	*TCRBJ01-06*01*
CASSHQRSGGAAEAFF	125	0.097408164	48	*TCRBV07-06*01*	*TCRBD02-01*02*	*TCRBJ01-01*01*
CSARDLLAGSNQPQHF	125	0.097408164	48	*TCRBV20*	*TCRBD02-01*01*	*TCRBJ01-05*01*
CASSTQGQQETQYF	124	0.096628898	42	*TCRBV11-02*02*	*TCRBD01-01*01*	*TCRBJ02-05*01*
CASRTAVLHWHHQPQHF	123	0.095849633	51	*TCRBV21-01*01*	*unresolved*	*TCRBJ01-05*01*
CASSKAGTSGRAGFYNEQFF	123	0.095849633	60	*TCRBV21-01*01*	*TCRBD02-01*02*	*TCRBJ02-01*01*
CASSLYRGGTQYF	123	0.095849633	39	*TCRBV05-06*01*	*TCRBD01-01*01*	*TCRBJ02-03*01*
CASSTYGTANYGYTF	123	0.095849633	45	*TCRBV05-01*01*	*TCRBD01-01*01*	*TCRBJ01-02*01*
CASSDFASGGGETQYF	122	0.095070368	48	*TCRBV06-01*01*	*TCRBD02-01*	*TCRBJ02-05*01*
CASSLGVGTTNEKLFF	122	0.095070368	48	*TCRBV05-01*01*	*unresolved*	*TCRBJ01-04*01*
CASSGRQGSPTDTQYF	121	0.094291102	48	*TCRBV02-01*01*	*TCRBD01-01*01*	*TCRBJ02-03*01*
CASSEAGGAHEQYF	120	0.093511837	42	*TCRBV02-01*01*	*TCRBD02-01*02*	*TCRBJ02-07*01*
CASSHLAGPYNEQFF	120	0.093511837	45	*TCRBV11-01*01*	*TCRBD02-01*01*	*TCRBJ02-01*01*
CASSIVRQAFNQPQHF	120	0.093511837	48	*TCRBV19-01*	*TCRBD01-01*01*	*TCRBJ01-05*01*
CASSLRGPGELFF	120	0.093511837	39	*TCRBV06-05*01*	*TCRBD01-01*01*	*TCRBJ02-02*01*
CASSSPNDYEQYF	120	0.093511837	39	*TCRBV07-02*01*	*unresolved*	*TCRBJ02-07*01*
CASGGRGKGNQPQHF	119	0.092732572	45	*TCRBV02-01*01*	*TCRBD02-01*02*	*TCRBJ01-05*01*
CASSLNWDRVDQSQVHEQYF	119	0.092732572	60	*TCRBV07-06*01*	*TCRBD01-01*01*	*TCRBJ02-07*01*
CATSSGQSNQPQHF	119	0.092732572	42	*TCRBV15-01*01*	*TCRBD01-01*01*	*TCRBJ01-05*01*
CASSDRVYGYTF	118	0.091953306	36	*TCRBV06-01*01*	*TCRBD01-01*01*	*TCRBJ01-02*01*
CASSLGTSVYNEQFF	118	0.091953306	45	*TCRBV06*	*TCRBD02-01*	*TCRBJ02-01*01*
CASSTGLAGGPESYEQYF	118	0.091953306	54	*TCRBV05-01*01*	*TCRBD02-01*02*	*TCRBJ02-07*01*
CATSRELGDDEQFF	118	0.091953306	42	*TCRBV15-01*01*	*unresolved*	*TCRBJ02-01*01*
CASSLAVVVYEQYF	117	0.091174041	42	*TCRBV05-05*01*	*TCRBD02-01*	*TCRBJ02-07*01*
CSATTGGNQPQHF	117	0.091174041	39	*TCRBV20*	*TCRBD01-01*01*	*TCRBJ01-05*01*
CASRGTSGSNEQFF	115	0.08961551	42	*TCRBV28-01*01*	*TCRBD02-01*02*	*TCRBJ02-01*01*
CASSLAGGKKNEQFF	115	0.08961551	45	*TCRBV05-01*01*	*TCRBD02-01*02*	*TCRBJ02-01*01*
CASSSVRGDTGELFF	115	0.08961551	45	*TCRBV02-01*01*	*unresolved*	*TCRBJ02-02*01*
CASYSGTGDQPQHF	115	0.08961551	42	*TCRBV28-01*01*	*TCRBD01-01*01*	*TCRBJ01-05*01*
CASSLRKRGGLDTQYF	114	0.088836245	48	*TCRBV28-01*01*	*TCRBD01-01*01*	*TCRBJ02-03*01*
CASSLSGSIHNEQFF	113	0.08805698	45	*TCRBV07-02*01*	*TCRBD02-01*02*	*TCRBJ02-01*01*
CASGGGTDSNQPQHF	111	0.086498449	45	*TCRBV06*	*TCRBD02-01*01*	*TCRBJ01-05*01*

CASGQDRINQPQHF	111	0.086498449	42	*TCRBV12-05*01*	*TCRBD01-01*01*	*TCRBJ01-05*01*
CASSLLGRTSTDTQYF	111	0.086498449	48	*TCRBV12*	*unresolved*	*TCRBJ02-03*01*
CASSPLTSGSSGEQYF	111	0.086498449	48	*TCRBV28-01*01*	*TCRBD02-01*02*	*TCRBJ02-07*01*
CASSYRGQSGGGTQYF	111	0.086498449	48	*TCRBV06-05*01*	*unresolved*	*TCRBJ02-03*01*
CASSKGLAGAQETQYF	109	0.084939919	48	*TCRBV06*	*TCRBD02-01*01*	*TCRBJ02-05*01*
CASSKRLTGELFF	109	0.084939919	39	*TCRBV19-01*	*TCRBD02-01*	*TCRBJ02-02*01*
CASSLGVNYEQYF	109	0.084939919	39	*TCRBV27-01*01*	*unresolved*	*TCRBJ02-07*01*
CASSQEGGGEQYF	109	0.084939919	39	*TCRBV04-02*01*	*TCRBD02-01*01*	*TCRBJ02-07*01*
CASSQGVLDQETQYF	109	0.084939919	45	*TCRBV04-01*01*	*TCRBD02-01*	*TCRBJ02-05*01*
CSARGEGGNEQFF	109	0.084939919	39	*TCRBV20*	*TCRBD02-01*02*	*TCRBJ02-01*01*
CSASQGGNQPQHF	109	0.084939919	39	*TCRBV20*	*TCRBD01-01*01*	*TCRBJ01-05*01*
CASSLVGTGNEKLFF	108	0.084160653	45	*TCRBV27-01*01*	*TCRBD01-01*01*	*TCRBJ01-04*01*
CASSSEGQYGYTF	108	0.084160653	39	*TCRBV05-01*01*	*TCRBD01-01*01*	*TCRBJ01-02*01*
CASSELQGNTGELFF	107	0.083381388	45	*TCRBV25-01*01*	*TCRBD01-01*01*	*TCRBJ02-02*01*
CASSFGQGDEQFF	107	0.083381388	39	*TCRBV07-06*01*	*TCRBD01-01*01*	*TCRBJ02-01*01*
CASSISIYTIRTEAFF	107	0.083381388	48	*TCRBV21-01*01*	*TCRBD01-01*01*	*TCRBJ01-01*01*
CASSPGLAGTKNIQYF	107	0.083381388	48	*TCRBV07-03*01*	*TCRBD02-01*01*	*TCRBJ02-04*01*
CSADPRGLAARDEQFF	107	0.083381388	48	*TCRBV20*	*TCRBD02-01*	*TCRBJ02-01*01*
CASSLSSLGRTVEKLFF	106	0.082602123	51	*TCRBV07-09*	*unresolved*	*TCRBJ01-04*01*
CASRTLAGVRTGELFF	105	0.081822857	48	*TCRBV06*	*TCRBD02-01*02*	*TCRBJ02-02*01*
CASSELASGGITDTQYF	105	0.081822857	51	*TCRBV02-01*01*	*TCRBD02-01*01*	*TCRBJ02-03*01*
CASSLFTGRPYEQYF	105	0.081822857	45	*TCRBV27-01*01*	*TCRBD01-01*01*	*TCRBJ02-07*01*
CASSSPTTNVLTF	105	0.081822857	39	*TCRBV18-01*01*	*unresolved*	*TCRBJ02-06*01*
CASSVRLAGNTGELFF	105	0.081822857	48	*TCRBV02-01*01*	*TCRBD02-01*	*TCRBJ02-02*01*
CSARGSGADTQYF	104	0.081043592	39	*TCRBV20*	*TCRBD02-01*01*	*TCRBJ02-03*01*
CASSLTGVSYNEQFF	103	0.080264327	45	*TCRBV05-01*01*	*TCRBD01-01*01*	*TCRBJ02-01*01*
CASSFGQTEAFF	102	0.079485061	36	*TCRBV07-09*	*TCRBD01-01*01*	*TCRBJ01-01*01*
CASSSTSGSWYTQYF	102	0.079485061	45	*TCRBV06-05*01*	*TCRBD02-01*02*	*TCRBJ02-03*01*
CASSVPRGLEETQYF	102	0.079485061	45	*TCRBV02-01*01*	*TCRBD01-01*01*	*TCRBJ02-05*01*
CTSSRAGGPNTDTQYF	102	0.079485061	48	*TCRBV01-01*01*	*TCRBD02-01*02*	*TCRBJ02-03*01*
CASRPEMLQQSRANVLTF	101	0.078705796	54	*TCRBV21-01*01*	*unresolved*	*TCRBJ02-06*01*
CASSARVRRQETQYF	101	0.078705796	45	*TCRBV28-01*01*	*TCRBD02-01*	*TCRBJ02-05*01*

CASSLTGMNGYTF	101	0.078705796	39	*TCRBV11-02*02*	*TCRBD01-01*01*	*TCRBJ01-02*01*
CASSEQGALYGYTF	100	0.077926531	42	*TCRBV02-01*01*	*TCRBD01-01*01*	*TCRBJ01-02*01*
CASSGLGGGTDTQYF	100	0.077926531	45	*TCRBV06-05*01*	*TCRBD02-01*01*	*TCRBJ02-03*01*
CASSQWDPFVMQDTQYF	100	0.077926531	51	*TCRBV03*	*unresolved*	*TCRBJ02-03*01*
CASSPPGDEQFF	99	0.077147266	36	*TCRBV27-01*01*	*TCRBD01-01*01*	*TCRBJ02-01*01*
CSGTSNQVTTYEQYF	99	0.077147266	45	*TCRBV20*	*TCRBD01-01*01*	*TCRBJ02-07*01*
CASSEQGPSAEAFF	98	0.076368	42	*TCRBV02-01*01*	*TCRBD01-01*01*	*TCRBJ01-01*01*
CSATAGTEINQPQHF	98	0.076368	45	*TCRBV20*	*TCRBD01-01*01*	*TCRBJ01-05*01*
CASSPRAGGETQYF	97	0.075588735	42	*TCRBV05-01*01*	*TCRBD01-01*01*	*TCRBJ02-05*01*
CASSVTESGELFF	97	0.075588735	39	*TCRBV06-01*01*	*TCRBD01-01*01*	*TCRBJ02-02*01*
CATSALGQGAPNEQFF	97	0.075588735	48	*TCRBV24*	*TCRBD01-01*01*	*TCRBJ02-01*01*
CSAVNTGELFF	97	0.075588735	33	*TCRBV20-01*01*	*TCRBD02-01*	*TCRBJ02-02*01*
CASSLIGTGPTSWQYF	96	0.07480947	48	*TCRBV05-01*01*	*unresolved*	*TCRBJ02-07*01*
CASSDAYEQYF	95	0.074030204	33	*TCRBV28-01*01*	*unresolved*	*TCRBJ02-07*01*
CASSWGEGSYNEQFF	95	0.074030204	45	*TCRBV11-03*01*	*TCRBD02-01*02*	*TCRBJ02-01*01*
CSFRQGAGNEQYF	95	0.074030204	39	*TCRBV29-01*01*	*TCRBD01-01*01*	*TCRBJ02-07*01*
CSVERGQGMNTEAFF	95	0.074030204	45	*TCRBV29-01*01*	*TCRBD01-01*01*	*TCRBJ01-01*01*
CASGPGPEADTQYF	94	0.073250939	42	*TCRBV28-01*01*	*TCRBD02-01*02*	*TCRBJ02-03*01*
CASRVDSRQPQHF	93	0.072471674	39	*TCRBV02-01*01*	*TCRBD01-01*01*	*TCRBJ01-05*01*
CASSLGAGGGTEAFF	93	0.072471674	45	*TCRBV07-08*01*	*TCRBD01-01*01*	*TCRBJ01-01*01*
CASSLGQGAEAFF	93	0.072471674	39	*TCRBV07-09*	*TCRBD01-01*01*	*TCRBJ01-01*01*
CSARGAGNTYEQYF	93	0.072471674	42	*TCRBV20*	*TCRBD01-01*01*	*TCRBJ02-07*01*
CASSEAGTGRNTIYF	92	0.071692408	45	*TCRBV02-01*01*	*TCRBD01-01*01*	*TCRBJ01-03*01*
CASSLGGARNTEAFF	92	0.071692408	45	*TCRBV27-01*01*	*TCRBD02-01*01*	*TCRBJ01-01*01*
CASSVEQLAEQFF	92	0.071692408	39	*TCRBV09-01*	*unresolved*	*TCRBJ02-01*01*
CAWSRGSSYEQYF	92	0.071692408	39	*TCRBV30-01*01*	*TCRBD02-01*01*	*TCRBJ02-07*01*
CASSGRGRGETQYF	91	0.070913143	42	*TCRBV05-01*01*	*TCRBD02-01*02*	*TCRBJ02-05*01*
CASSLNPPLNRAGQSYNEQFF	91	0.070913143	63	*TCRBV06*	*TCRBD01-01*01*	*TCRBJ02-01*01*
CASSLTSGGPGQFF	91	0.070913143	42	*TCRBV11-02*02*	*TCRBD02-01*01*	*TCRBJ02-01*01*
CASRSTGTTAGGEQFF	90	0.070133878	48	*TCRBV05-01*01*	*TCRBD02-01*01*	*TCRBJ02-01*01*
CASSLGLQGALNEQFF	90	0.070133878	48	*TCRBV05-01*01*	*TCRBD01-01*01*	*TCRBJ02-01*01*
CASSSTPGTSGSHEQFF	89	0.069354612	51	*TCRBV12*	*TCRBD02-01*02*	*TCRBJ02-01*01*

CASSHWVSRETQYF	88	0.068575347	42	*TCRBV03*	*TCRBD02-01*	*TCRBJ02-05*01*
CASSLAGAPLNEQYF	88	0.068575347	45	*TCRBV05-04*01*	*TCRBD01-01*01*	*TCRBJ02-07*01*
CASSPRGRVVLNEQFF	87	0.067796082	48	*TCRBV19-01*	*TCRBD01-01*01*	*TCRBJ02-01*01*
CSARPPGGGTTDTQYF	87	0.067796082	48	*TCRBV20*	*TCRBD02-01*01*	*TCRBJ02-03*01*
CASSERIGGSPTGDTQYF	86	0.067016817	54	*TCRBV06-01*01*	*TCRBD01-01*01*	*TCRBJ02-03*01*
CASSHHGKGGHTEAFF	86	0.067016817	48	*TCRBV07-06*01*	*unresolved*	*TCRBJ01-01*01*
CASTRTAPNYGYTF	86	0.067016817	42	*TCRBV27-01*01*	*TCRBD01-01*01*	*TCRBJ01-02*01*
CASSALISGPGTDTQYF	85	0.066237551	51	*TCRBV12*	*unresolved*	*TCRBJ02-03*01*
CASSGPGVAGGETQYF	85	0.066237551	48	*TCRBV07-09*	*TCRBD02-01*02*	*TCRBJ02-05*01*
CASSLQPGAVYTGELFF	85	0.066237551	51	*TCRBV05-05*01*	*TCRBD02-01*02*	*TCRBJ02-02*01*
CASSLREMPDTQYF	85	0.066237551	42	*TCRBV05-01*01*	*TCRBD02-01*02*	*TCRBJ02-03*01*
CASSHGQGATRNGYTF	84	0.065458286	48	*TCRBV12*	*TCRBD01-01*01*	*TCRBJ01-02*01*
CASSLDGTGNTEAFF	84	0.065458286	45	*TCRBV11-03*01*	*TCRBD01-01*01*	*TCRBJ01-01*01*
CSAQTSGRHYEQFF	84	0.065458286	42	*TCRBV20*	*TCRBD02-01*02*	*TCRBJ02-01*01*
CASSLYPPGGDSNQPQHF	83	0.064679021	54	*TCRBV27-01*01*	*TCRBD02-01*02*	*TCRBJ01-05*01*
CASSYGGEQYF	83	0.064679021	33	*TCRBV06-05*01*	*TCRBD02-01*	*TCRBJ02-07*01*
CSGTLLDTQYF	82	0.063899755	33	*TCRBV20-01*01*	*TCRBD02-01*	*TCRBJ02-03*01*
CSVSGGDYEQYF	82	0.063899755	36	*TCRBV29-01*01*	*TCRBD02-01*01*	*TCRBJ02-07*01*
CASATDPNEQFF	81	0.06312049	36	*TCRBV06-06*	*unresolved*	*TCRBJ02-01*01*
CASKGGTGIYNEQFF	81	0.06312049	45	*TCRBV02-01*01*	*TCRBD01-01*01*	*TCRBJ02-01*01*
CAWSYGGASSGANVLTF	81	0.06312049	51	*TCRBV30-01*01*	*TCRBD02-01*01*	*TCRBJ02-06*01*
CATSRDRTRRGEQYF	80	0.062341225	45	*TCRBV15-01*01*	*TCRBD02-01*01*	*TCRBJ02-07*01*
CSASPDSGRPDTQYF	79	0.061561959	45	*TCRBV20*	*TCRBD01-01*01*	*TCRBJ02-03*01*
CASGLVSSGRAVTGELFF	78	0.060782694	54	*TCRBV12-05*01*	*TCRBD02-01*02*	*TCRBJ02-02*01*
CASSSGDSADTQYF	78	0.060782694	42	*TCRBV05-06*01*	*TCRBD02-01*	*TCRBJ02-03*01*
CATTEGHSNTEAFF	78	0.060782694	42	*TCRBV10-03*01*	*TCRBD01-01*01*	*TCRBJ01-01*01*
CASNLGTANNQPQHF	77	0.060003429	45	*TCRBV02-01*01*	*TCRBD01-01*01*	*TCRBJ01-05*01*
CASSYSWQGAAEQYF	77	0.060003429	45	*TCRBV06-05*01*	*TCRBD01-01*01*	*TCRBJ02-07*01*
CASSPSSPFHSDNEQFF	76	0.059224163	51	*TCRBV03*	*TCRBD02-01*02*	*TCRBJ02-01*01*
CATRGQGGPEAFF	76	0.059224163	39	*TCRBV24*	*TCRBD01-01*01*	*TCRBJ01-01*01*
CASSAARGTNNEQFF	75	0.058444898	45	*TCRBV25-01*01*	*TCRBD02-01*	*TCRBJ02-01*01*
CASSLGFQETQYF	75	0.058444898	39	*TCRBV05-01*01*	*unresolved*	*TCRBJ02-05*01*

CSVELAGGYEQYF	75	0.058444898	39	*TCRBV29-01*01*	*TCRBD02-01*02*	*TCRBJ02-07*01*
CASLKQSRANVLTF	74	0.057665633	42	*TCRBV09-01*	*TCRBD01-01*01*	*TCRBJ02-06*01*
CASSLGSEHNEQFF	74	0.057665633	42	*TCRBV11-03*01*	*TCRBD02-01*	*TCRBJ02-01*01*
CASSPGDGGRDTQYF	74	0.057665633	45	*TCRBV28-01*01*	*TCRBD02-01*01*	*TCRBJ02-03*01*
CASSSPPGTQYF	74	0.057665633	36	*TCRBV27-01*01*	*unresolved*	*TCRBJ02-05*01*
CASSVDGSNDEQFF	74	0.057665633	42	*TCRBV09-01*	*TCRBD02-01*	*TCRBJ02-01*01*
CSAFRTGTDTQYF	74	0.057665633	39	*TCRBV20*	*TCRBD02-01*	*TCRBJ02-03*01*
CASSFPGGGETQYF	72	0.056107102	42	*TCRBV05-01*01*	*unresolved*	*TCRBJ02-05*01*
CASSLVGTGPYNEQFF	72	0.056107102	48	*TCRBV07-09*	*TCRBD01-01*01*	*TCRBJ02-01*01*
CASSLWGDNGYTF	72	0.056107102	39	*TCRBV27-01*01*	*TCRBD02-01*01*	*TCRBJ01-02*01*
CASSQSMNTEAFF	72	0.056107102	39	*TCRBV05-05*01*	*unresolved*	*TCRBJ01-01*01*
CSVVGTSGAGQKLFF	72	0.056107102	45	*TCRBV29-01*01*	*unresolved*	*TCRBJ01-04*01*
CASSLWGKSEAFF	71	0.055327837	39	*TCRBV27-01*01*	*unresolved*	*TCRBJ01-01*01*
CSARHFGTGDTDTQYF	71	0.055327837	48	*TCRBV20*	*TCRBD01-01*01*	*TCRBJ02-03*01*
CASSPGEGGRDTQYF	70	0.054548572	45	*TCRBV28-01*01*	*TCRBD02-01*	*TCRBJ02-03*01*
CASSSQGAYEQYF	70	0.054548572	39	*TCRBV06-06*	*TCRBD01-01*01*	*TCRBJ02-07*01*
CSAREGTSGGNFYEQYF	70	0.054548572	51	*TCRBV20*	*TCRBD02-01*01*	*TCRBJ02-07*01*
CASSVTGLAGVDYNEQFF	69	0.053769306	54	*TCRBV09-01*	*TCRBD02-01*02*	*TCRBJ02-01*01*
CSAERNPDTDTQYF	69	0.053769306	42	*TCRBV20*	*TCRBD02-01*02*	*TCRBJ02-03*01*
CASSIAQGATEAFF	68	0.052990041	42	*TCRBV19-01*	*TCRBD01-01*01*	*TCRBJ01-01*01*
CASSVSGSSTQYF	68	0.052990041	39	*TCRBV06-01*01*	*TCRBD02-01*02*	*TCRBJ02-03*01*
CSAPRGGLGYTF	68	0.052990041	36	*TCRBV20*	*TCRBD01-01*01*	*TCRBJ01-02*01*
CSVEAVDSSYEQYF	68	0.052990041	42	*TCRBV29-01*01*	*unresolved*	*TCRBJ02-07*01*
CAGSPNGGTNEKLFF	67	0.052210776	45	*TCRBV07-03*01*	*unresolved*	*TCRBJ01-04*01*
CASSLDGVITSNEQFF	67	0.052210776	48	*TCRBV27-01*01*	*unresolved*	*TCRBJ02-01*01*
CASSSSGGAVDTQYF	67	0.052210776	45	*TCRBV06-01*01*	*TCRBD02-01*01*	*TCRBJ02-03*01*
CASSVGSAGNNEQFF	67	0.052210776	45	*TCRBV09-01*	*TCRBD02-01*	*TCRBJ02-01*01*
CAWSLAEPRTDTQYF	67	0.052210776	45	*TCRBV30-01*01*	*TCRBD02-01*	*TCRBJ02-03*01*
CSAISGLAGAVEQFF	67	0.052210776	45	*TCRBV20*	*TCRBD02-01*02*	*TCRBJ02-01*01*
CASSLGQGPFF	66	0.05143151	33	*TCRBV11-02*02*	*TCRBD01-01*01*	*TCRBJ01-02*01*
CASSYFVPFVMQDTQYF	66	0.05143151	51	*TCRBV03*	*unresolved*	*TCRBJ02-03*01*
CSAHRGRETQYF	66	0.05143151	36	*TCRBV20*	*TCRBD01-01*01*	*TCRBJ02-05*01*

CASIVRDRENTQYF	65	0.050652245	42	*TCRBV02-01*01*	*TCRBD01-01*01*	*TCRBJ02-03*01*
CASLNLGLGETQYF	65	0.050652245	42	*TCRBV12*	*TCRBD02-01*	*TCRBJ02-05*01*
CASSLARGGNEQFF	65	0.050652245	42	*TCRBV07-02*01*	*TCRBD01-01*01*	*TCRBJ02-01*01*
CASSTRTDGHNEQFF	65	0.050652245	45	*TCRBV27-01*01*	*unresolved*	*TCRBJ02-01*01*
CSVETADYEQYF	65	0.050652245	36	*TCRBV29-01*01*	*TCRBD02-01*	*TCRBJ02-07*01*
CASLRGDVGMNIQYF	64	0.04987298	45	*TCRBV02-01*01*	*TCRBD01-01*01*	*TCRBJ02-04*01*
CASSGRGFYQPQHF	64	0.04987298	42	*TCRBV25-01*01*	*TCRBD01-01*01*	*TCRBJ01-05*01*
CASSYGVKETQYF	64	0.04987298	39	*TCRBV27-01*01*	*unresolved*	*TCRBJ02-05*01*
CASSAPDRGRTEAFF	63	0.049093714	45	*TCRBV09-01*	*TCRBD01-01*01*	*TCRBJ01-01*01*
CASSRTGHLGKTYGYTF	63	0.049093714	51	*TCRBV11-03*01*	*TCRBD01-01*01*	*TCRBJ01-02*01*
CASSSAGGGRREQFF	63	0.049093714	45	*TCRBV07-06*01*	*TCRBD02-01*01*	*TCRBJ02-01*01*
CSVEDVGDYEQYF	63	0.049093714	39	*TCRBV29-01*01*	*unresolved*	*TCRBJ02-07*01*
CASSFMNTEAFF	62	0.048314449	36	*TCRBV07-09*	*TCRBD02-01*	*TCRBJ01-01*01*
CASSFSGRPNEQFF	62	0.048314449	42	*TCRBV06*	*TCRBD02-01*02*	*TCRBJ02-01*01*
CASSLGSTDTQYF	62	0.048314449	39	*TCRBV05-01*01*	*unresolved*	*TCRBJ02-03*01*
CASSSTGGNYGYTF	62	0.048314449	42	*TCRBV05-01*01*	*TCRBD01-01*01*	*TCRBJ01-02*01*
CSVVTGAGYGYTF	62	0.048314449	39	*TCRBV29-01*01*	*unresolved*	*TCRBJ01-02*01*
CASSPTTDSYEQYF	61	0.047535184	42	*TCRBV11-02*02*	*TCRBD01-01*01*	*TCRBJ02-07*01*
CASSQTLSGELFF	61	0.047535184	39	*TCRBV03*	*TCRBD02-01*	*TCRBJ02-02*01*
CASSYRVRGTEAFF	61	0.047535184	42	*TCRBV06-05*01*	*TCRBD01-01*01*	*TCRBJ01-01*01*
CSVGSVGTEAFF	61	0.047535184	36	*TCRBV29-01*01*	*TCRBD02-01*	*TCRBJ01-01*01*
CASANGQGINTEAFF	60	0.046755919	45	*TCRBV05-01*01*	*TCRBD01-01*01*	*TCRBJ01-01*01*
CSAYGQTSKNIQYF	60	0.046755919	42	*TCRBV20*	*TCRBD01-01*01*	*TCRBJ02-04*01*
CASSPDRELDTQYF	59	0.045976653	42	*TCRBV07-08*01*	*TCRBD01-01*01*	*TCRBJ02-03*01*
CATSFRGRLSLLDTQYF	59	0.045976653	51	*TCRBV27-01*01*	*TCRBD02-01*	*TCRBJ02-03*01*
CRGAGGVDEQFF	59	0.045976653	36	*TCRBV20*	*TCRBD02-01*02*	*TCRBJ02-01*01*
CASSLDETQYF	58	0.045197388	33	*TCRBV07-02*01*	*unresolved*	*TCRBJ02-05*01*
CASSRWGLGEYNEQFF	58	0.045197388	48	*TCRBV05-04*01*	*TCRBD02-01*	*TCRBJ02-01*01*
CASSYSGSSYNEQFF	58	0.045197388	45	*TCRBV05-04*01*	*TCRBD02-01*02*	*TCRBJ02-01*01*
CAIRGGLAGKNEQFF	57	0.044418123	45	*TCRBV10-03*01*	*TCRBD02-01*02*	*TCRBJ02-01*01*
CASSLAGGQETQYF	57	0.044418123	42	*TCRBV03*	*TCRBD02-01*01*	*TCRBJ02-05*01*
CASSVLAGQKGASYEQYF	57	0.044418123	54	*TCRBV02-01*01*	*TCRBD02-01*	*TCRBJ02-07*01*

CASSLDRGSGAFF	56	0.043638857	39	*TCRBV07-03*01*	*TCRBD01-01*01*	*TCRBJ01-01*01*
CASSARTKSRKTYGYTF	55	0.042859592	51	*TCRBV07-06*01*	*TCRBD02-01*	*TCRBJ01-02*01*
CASSEASGFTDTQYF	55	0.042859592	45	*TCRBV06-04*	*TCRBD02-01*	*TCRBJ02-03*01*
CASSFSGVFGGELFF	55	0.042859592	45	*TCRBV07-09*	*TCRBD01-01*01*	*TCRBJ02-02*01*
CASSQAASYYNEQFF	55	0.042859592	45	*TCRBV03*	*unresolved*	*TCRBJ02-01*01*
CASSTRSPQSRANVLTF	55	0.042859592	51	*TCRBV07-08*01*	*TCRBD01-01*01*	*TCRBJ02-06*01*
CATSDRRPGETQYF	55	0.042859592	42	*TCRBV24*	*TCRBD02-01*01*	*TCRBJ02-05*01*
CSVEGTGQADTQYF	55	0.042859592	42	*TCRBV29-01*01*	*unresolved*	*TCRBJ02-03*01*
CASSQVPRGTDTQYF	54	0.042080327	45	*TCRBV04-01*01*	*TCRBD02-01*	*TCRBJ02-03*01*
CSARVLAEVTDTQYF	54	0.042080327	45	*TCRBV20*	*TCRBD02-01*	*TCRBJ02-03*01*
CASSEQEWGQNTQYF	53	0.041301061	45	*TCRBV02-01*01*	*TCRBD01-01*01*	*TCRBJ02-03*01*
CASSLTSGGADTQYF	53	0.041301061	45	*TCRBV07-03*01*	*TCRBD02-01*01*	*TCRBJ02-03*01*
CASSFGGVAGELFF	52	0.040521796	42	*TCRBV27-01*01*	*TCRBD02-01*01*	*TCRBJ02-02*01*
CASSFSTSTDTQYF	52	0.040521796	42	*TCRBV28-01*01*	*unresolved*	*TCRBJ02-03*01*
CASSQDGEAIGGKNIQYF	52	0.040521796	54	*TCRBV04-01*01*	*TCRBD02-01*02*	*TCRBJ02-04*01*
CASSRTANYNEQFF	51	0.039742531	42	*TCRBV19-01*	*TCRBD01-01*01*	*TCRBJ02-01*01*
CASSSGTFNNEQFF	51	0.039742531	42	*TCRBV11-03*01*	*TCRBD02-01*	*TCRBJ02-01*01*
CSASKGMETQYF	51	0.039742531	36	*TCRBV20*	*TCRBD01-01*01*	*TCRBJ02-05*01*
CARTRGTGGQSDTGELFF	50	0.038963265	54	*TCRBV06*	*TCRBD01-01*01*	*TCRBJ02-02*01*
CASNIVREYEYGYTF	50	0.038963265	45	*TCRBV05-02*01*	*unresolved*	*TCRBJ01-02*01*
CASMGRLATGRETQYF	49	0.038184	48	*TCRBV19-01*	*TCRBD01-01*01*	*TCRBJ02-05*01*
CASRTDVGGFYGYTF	49	0.038184	45	*TCRBV05-01*01*	*TCRBD02-01*02*	*TCRBJ01-02*01*
CASRVKDYGYTF	49	0.038184	36	*TCRBV28-01*01*	*unresolved*	*TCRBJ01-02*01*
CATSDPLAGVVNEQFF	49	0.038184	48	*TCRBV24*	*TCRBD02-01*02*	*TCRBJ02-01*01*
CSARSGTSGRGDTQYF	49	0.038184	48	*TCRBV20*	*TCRBD02-01*02*	*TCRBJ02-03*01*
CASSAGTGSGSEAFF	48	0.037404735	45	*TCRBV09-01*	*TCRBD01-01*01*	*TCRBJ01-01*01*
CASSQEVGRLSYNEQFF	48	0.037404735	51	*TCRBV04-01*01*	*TCRBD01-01*01*	*TCRBJ02-01*01*
CASSVPSGPYQETQYF	48	0.037404735	48	*TCRBV09-01*	*TCRBD02-01*	*TCRBJ02-05*01*
CASSISGSGNEQFF	47	0.03662547	42	*TCRBV19-01*	*TCRBD02-01*02*	*TCRBJ02-01*01*
CASSLFTGDEAFF	47	0.03662547	39	*TCRBV27-01*01*	*TCRBD01-01*01*	*TCRBJ01-01*01*
CAWSTGYGYTF	47	0.03662547	33	*TCRBV30-01*01*	*TCRBD01-01*01*	*TCRBJ01-02*01*
CASSLAVTDTQYF	46	0.035846204	39	*TCRBV05-05*01*	*TCRBD02-01*	*TCRBJ02-03*01*

CASSPRGGLNQPQHF	46	0.035846204	45	*TCRBV28-01*01*	*TCRBD02-01*02*	*TCRBJ01-05*01*
CASRVDRGQIGTGELFF	45	0.035066939	51	*TCRBV02-01*01*	*TCRBD01-01*01*	*TCRBJ02-02*01*
CSARDRGSSSGGGRNIQYF	45	0.035066939	57	*TCRBV20*	*TCRBD02-01*01*	*TCRBJ02-04*01*
CASSQGGRAEAFF	44	0.034287674	39	*TCRBV04-01*01*	*TCRBD01-01*01*	*TCRBJ01-01*01*
CSVEFSSTDTQYF	44	0.034287674	39	*TCRBV29-01*01*	*TCRBD02-01*	*TCRBJ02-03*01*
CASSLALGGNTEAFF	42	0.032729143	45	*TCRBV12*	*TCRBD02-01*01*	*TCRBJ01-01*01*
CAYRGEAGAGANVLTF	42	0.032729143	48	*TCRBV10-03*01*	*TCRBD02-01*01*	*TCRBJ02-06*01*
CASSDPHEYGYTF	41	0.031949878	39	*TCRBV25-01*01*	*unresolved*	*TCRBJ01-02*01*
CASSEGGNKNIQYF	41	0.031949878	42	*TCRBV25-01*01*	*unresolved*	*TCRBJ02-04*01*
CASSRQAKETQYF	41	0.031949878	39	*TCRBV06-04*	*TCRBD01-01*01*	*TCRBJ02-05*01*
CSVEGQGASETQYF	41	0.031949878	42	*TCRBV29-01*01*	*TCRBD01-01*01*	*TCRBJ02-05*01*
CASSAGGSYNEQFF	40	0.031170612	42	*TCRBV10-01*	*TCRBD02-01*02*	*TCRBJ02-01*01*
CASSDRIYGYTF	40	0.031170612	36	*TCRBV06-01*01*	*TCRBD01-01*01*	*TCRBJ01-02*01*
CASSLLTGTGYEKLFF	40	0.031170612	48	*TCRBV28-01*01*	*TCRBD01-01*01*	*TCRBJ01-04*01*
CASSQARQETQYF	40	0.031170612	39	*TCRBV03*	*TCRBD02-01*	*TCRBJ02-05*01*
CSGRETGNTGELFF	40	0.031170612	42	*TCRBV29-01*01*	*TCRBD01-01*01*	*TCRBJ02-02*01*
CSVERGTSLQETQYF	40	0.031170612	45	*TCRBV29-01*01*	*TCRBD02-01*	*TCRBJ02-05*01*
CAISDGRLAGGVDEQFF	39	0.030391347	51	*TCRBV10-03*01*	*TCRBD02-01*02*	*TCRBJ02-01*01*
CASSFQLAGDVYNEQFF	39	0.030391347	51	*TCRBV11-02*02*	*TCRBD02-01*02*	*TCRBJ02-01*01*
CASSLGGDSNQPQHF	39	0.030391347	45	*TCRBV05-01*01*	*TCRBD02-01*01*	*TCRBJ01-05*01*
CASSQDARRELFF	39	0.030391347	39	*TCRBV04-01*01*	*unresolved*	*TCRBJ02-02*01*
CASSLGNEQGKQFF	38	0.029612082	42	*TCRBV07-06*01*	*TCRBD01-01*01*	*TCRBJ02-01*01*
CSARDLVAGGRGTQYF	37	0.028832816	48	*TCRBV20*	*TCRBD02-01*02*	*TCRBJ02-03*01*
CAQGGLAGGTIRTDTQYF	36	0.028053551	54	*TCRBV02-01*01*	*TCRBD02-01*01*	*TCRBJ02-03*01*
CASSLEGNTEAFF	35	0.027274286	39	*TCRBV05-01*01*	*unresolved*	*TCRBJ01-01*01*
CASSVGGLAGDEQYF	35	0.027274286	45	*TCRBV09-01*	*TCRBD02-01*01*	*TCRBJ02-07*01*
CSARLLGGNEQFF	35	0.027274286	39	*TCRBV20*	*TCRBD02-01*	*TCRBJ02-01*01*
CSVEVRVDNEQFF	35	0.027274286	39	*TCRBV29-01*01*	*TCRBD01-01*01*	*TCRBJ02-01*01*
CASSFDRLLTGENEQFF	34	0.02649502	51	*TCRBV28-01*01*	*TCRBD01-01*01*	*TCRBJ02-01*01*
CASSLDQETQYF	34	0.02649502	36	*TCRBV05-01*01*	*unresolved*	*TCRBJ02-05*01*
CASSSGLAGGVSYNEQFF	34	0.02649502	54	*TCRBV05-04*01*	*TCRBD02-01*01*	*TCRBJ02-01*01*
CSVEDPGAEAFF	34	0.02649502	36	*TCRBV29-01*01*	*TCRBD01-01*01*	*TCRBJ01-01*01*

CASSGGEPLRGWNEQFF	33	0.025715755	51	*TCRBV18-01*01*	*TCRBD02-01*01*	*TCRBJ02-01*01*
CSVAWRSGTDTQYF	33	0.025715755	42	*TCRBV29-01*01*	*TCRBD02-01*	*TCRBJ02-03*01*
CASRHPTPSQGQYF	32	0.02493649	42	*TCRBV27-01*01*	*unresolved*	*TCRBJ02-03*01*
CASRQLGRSAYNEQFF	32	0.02493649	48	*TCRBV12*	*unresolved*	*TCRBJ02-01*01*
CASSLLTSGRPNEQFF	32	0.02493649	48	*TCRBV27-01*01*	*TCRBD02-01*02*	*TCRBJ02-01*01*
CSARDGRGLYEQYF	32	0.02493649	42	*TCRBV20*	*TCRBD01-01*01*	*TCRBJ02-07*01*
CAWSVLGETQYF	31	0.024157225	36	*TCRBV30-01*01*	*TCRBD02-01*	*TCRBJ02-05*01*
CSAIDFGLAGTLPVKETQYF	31	0.024157225	60	*TCRBV20*	*TCRBD02-01*02*	*TCRBJ02-05*01*
CASSFWTSGGIGNEQFF	30	0.023377959	51	*TCRBV27-01*01*	*TCRBD02-01*01*	*TCRBJ02-01*01*
CAWSSSGRAGANVLTF	30	0.023377959	48	*TCRBV30-01*01*	*TCRBD02-01*02*	*TCRBJ02-06*01*
CSIDRGAFYNEQFF	30	0.023377959	42	*TCRBV29-01*01*	*TCRBD01-01*01*	*TCRBJ02-01*01*
CSGSGPNAQYF	29	0.022598694	33	*TCRBV20-01*01*	*unresolved*	*TCRBJ02-05*01*
CASSESMGSETQYF	28	0.021819429	42	*TCRBV07-09*	*TCRBD01-01*01*	*TCRBJ02-05*01*
CASSPGFSETQYF	28	0.021819429	39	*TCRBV07-09*	*TCRBD01-01*01*	*TCRBJ02-05*01*
CASSSMTSGGETQYF	28	0.021819429	45	*TCRBV07-03*01*	*TCRBD02-01*01*	*TCRBJ02-05*01*
CSARDGGPPDTQYF	28	0.021819429	42	*TCRBV20*	*unresolved*	*TCRBJ02-03*01*
CSVGLAGSYGYNEQFF	28	0.021819429	48	*TCRBV29-01*01*	*TCRBD02-01*02*	*TCRBJ02-01*01*
CASSKGGTELSGANVLTF	27	0.021040163	54	*TCRBV12*	*unresolved*	*TCRBJ02-06*01*
CASSLRGLGTDTQYF	27	0.021040163	45	*TCRBV05-01*01*	*TCRBD02-01*01*	*TCRBJ02-03*01*
CASSLRQTAGAEAFF	27	0.021040163	45	*TCRBV27-01*01*	*unresolved*	*TCRBJ01-01*01*
CASSSTWGQEYGYTF	27	0.021040163	45	*TCRBV28-01*01*	*TCRBD01-01*01*	*TCRBJ01-02*01*
CSASNSTRVLLGEQFF	27	0.021040163	48	*TCRBV20*	*TCRBD01-01*01*	*TCRBJ02-01*01*
CSVEGQGYTDTQYF	27	0.021040163	42	*TCRBV29-01*01*	*TCRBD01-01*01*	*TCRBJ02-03*01*
CASSVGGMGYNEQFF	26	0.020260898	45	*TCRBV09-01*	*unresolved*	*TCRBJ02-01*01*
CASTSWGGHSGKETQYF	26	0.020260898	51	*TCRBV28-01*01*	*TCRBD02-01*01*	*TCRBJ02-05*01*
CSATALQRATDTQYF	26	0.020260898	45	*TCRBV20*	*unresolved*	*TCRBJ02-03*01*
CSATGQPDQPQHF	26	0.020260898	39	*TCRBV20*	*TCRBD01-01*01*	*TCRBJ01-05*01*
CSVEGIAQSDTQYF	26	0.020260898	42	*TCRBV29-01*01*	*TCRBD01-01*01*	*TCRBJ02-03*01*
CSSGGSNEQFF	25	0.019481633	33	*TCRBV29-01*01*	*TCRBD02-01*01*	*TCRBJ02-01*01*
CASSLGNRRATEAFF	24	0.018702367	45	*TCRBV05-01*01*	*TCRBD02-01*02*	*TCRBJ01-01*01*
CSVEGQGQADTQYF	24	0.018702367	42	*TCRBV29-01*01*	*TCRBD01-01*01*	*TCRBJ02-03*01*
CASSPTGGGNTDTQYF	23	0.017923102	48	*TCRBV11-02*02*	*TCRBD02-01*01*	*TCRBJ02-03*01*

CASRLRQGLTEAFF	22	0.017143837	42	*TCRBV07-02*01*	*unresolved*	*TCRBJ01-01*01*
CASSLAQGSTGELFF	22	0.017143837	45	*TCRBV13-01*01*	*TCRBD01-01*01*	*TCRBJ02-02*01*
CASGRRHLQGKTYGYTF	21	0.016364571	51	*TCRBV12-05*01*	*TCRBD01-01*01*	*TCRBJ01-02*01*
CASSPSGTPKETQYF	21	0.016364571	45	*TCRBV09-01*	*TCRBD02-01*02*	*TCRBJ02-05*01*
CASSHLGGFDTQYF	20	0.015585306	42	*TCRBV04-01*01*	*TCRBD02-01*01*	*TCRBJ02-03*01*
CASSLTISPPGEPLSYNEQFF	20	0.015585306	63	*TCRBV07-06*01*	*TCRBD01-01*01*	*TCRBJ02-01*01*
CASSYGGKDTQYF	20	0.015585306	39	*TCRBV06-04*	*TCRBD02-01*01*	*TCRBJ02-03*01*
CASSGGQHYQETQYF	19	0.014806041	45	*TCRBV25-01*01*	*TCRBD01-01*01*	*TCRBJ02-05*01*
CASSPPIARMNTEAFF	19	0.014806041	48	*TCRBV18-01*01*	*TCRBD01-01*01*	*TCRBJ01-01*01*
CASRGLALGETQYF	17	0.01324751	42	*TCRBV12*	*TCRBD02-01*	*TCRBJ02-05*01*
CATAAGGANTGELFF	17	0.01324751	45	*TCRBV28-01*01*	*TCRBD02-01*01*	*TCRBJ02-02*01*
CASSLERPTYNEQFF	16	0.012468245	45	*TCRBV05-01*01*	*TCRBD02-01*02*	*TCRBJ02-01*01*
CATSRDLGGDTQYF	16	0.012468245	42	*TCRBV15-01*01*	*unresolved*	*TCRBJ02-03*01*
CASCEMTGGRGGYTF	15	0.01168898	45	*TCRBV10-02*01*	*TCRBD02-01*01*	*TCRBJ01-02*01*
CASSHLRSGGDYEQYF	14	0.010909714	48	*TCRBV07-06*01*	*TCRBD01-01*01*	*TCRBJ02-07*01*
CASSLNLGGRNTGELFF	14	0.010909714	51	*TCRBV05-01*01*	*unresolved*	*TCRBJ02-02*01*
CASSPRLASFYPEQFF	14	0.010909714	48	*TCRBV06*	*TCRBD02-01*	*TCRBJ02-01*01*
CASSRDRVGGNSPLHF	14	0.010909714	48	*TCRBV19-01*	*TCRBD01-01*01*	*TCRBJ01-06*01*
CASSTKRALRKTYGYTF	14	0.010909714	51	*TCRBV03*	*unresolved*	*TCRBJ01-02*01*
CASSTTPGLAGGPSHEQFF	14	0.010909714	57	*TCRBV07-09*	*TCRBD02-01*02*	*TCRBJ02-01*01*
CASSVTSGIYEQYF	14	0.010909714	42	*TCRBV05-05*01*	*TCRBD02-01*02*	*TCRBJ02-07*01*
CSVEGTPGTQYF	14	0.010909714	36	*TCRBV29-01*01*	*unresolved*	*TCRBJ02-05*01*
CASSSTAGANVLTF	12	0.009351184	42	*TCRBV12*	*TCRBD01-01*01*	*TCRBJ02-06*01*
CATQPGASSGKLFF	12	0.009351184	42	*TCRBV19-01*	*unresolved*	*TCRBJ01-04*01*
CASSVEIDSDPFKNTQYF	11	0.008571918	54	*TCRBV09-01*	*TCRBD01-01*01*	*TCRBJ02-03*01*
CASSVGPLGLDTQYF	11	0.008571918	45	*TCRBV07-02*01*	*TCRBD02-01*	*TCRBJ02-03*01*
CASTLSLRNSPLHF	11	0.008571918	42	*TCRBV28-01*01*	*TCRBD02-01*02*	*TCRBJ01-06*01*
CASSAGGSCNEQFF	10	0.007792653	42	*TCRBV10-01*	*TCRBD02-01*02*	*TCRBJ02-01*01*
CAYRGAEGAGANVLTF	10	0.007792653	48	*TCRBV10-03*01*	*TCRBD02-01*01*	*TCRBJ02-06*01*
CASSEMTGGRGGYTL	9	0.007013388	45	*TCRBV10-02*01*	*TCRBD02-01*01*	*TCRBJ01-02*01*
CASSLQVGTQYF	9	0.007013388	36	*TCRBV07-06*01*	*TCRBD01-01*01*	*TCRBJ02-03*01*
CASSLRGPGANVLTF	9	0.007013388	45	*TCRBV05-06*01*	*TCRBD01-01*01*	*TCRBJ02-06*01*

CASSRGTGWNEQFF	8	0.006234122	42	*TCRBV03*	*TCRBD01-01*01*	*TCRBJ02-01*01*
CARGGRGKGNQPQHF	6	0.004675592	45	*TCRBV02-01*01*	*TCRBD02-01*01*	*TCRBJ01-05*01*
CASSEMMGGRGGYTF	6	0.004675592	45	*TCRBV10-02*01*	*TCRBD02-01*01*	*TCRBJ01-02*01*
CASSSGGSYNEQFF	6	0.004675592	42	*TCRBV11-02*02*	*TCRBD02-01*01*	*TCRBJ02-01*01*
CAYRGAAGAGANVLTF	6	0.004675592	48	*TCRBV10-03*01*	*TCRBD02-01*01*	*TCRBJ02-06*01*
CAGSAGGSCNEQFF	5	0.003896327	42	*TCRBV10-01*	*TCRBD02-01*02*	*TCRBJ02-01*01*
CASCEMKGGRGGYTF	4	0.003117061	45	*TCRBV10-02*01*	*TCRBD02-01*01*	*TCRBJ01-02*01*
CASSLCQRLAGGTYEQYF	4	0.003117061	54	*TCRBV27-01*01*	*TCRBD02-01*01*	*TCRBJ02-07*01*
CASSLGQGLHEQYF	4	0.003117061	42	*TCRBV05-04*01*	*TCRBD01-01*01*	*TCRBJ02-07*01*
CASSPGAGGRDTQYF	4	0.003117061	45	*TCRBV28-01*01*	*TCRBD02-01*01*	*TCRBJ02-03*01*
CASSRGEGGRDTQYF	4	0.003117061	45	*TCRBV28-01*01*	*TCRBD02-01*	*TCRBJ02-03*01*
CASSVLEGTDTQYF	4	0.003117061	42	*TCRBV06-05*01*	*TCRBD02-01*02*	*TCRBJ02-03*01*
CASSYSRGDRGGYERYF	4	0.003117061	51	*TCRBV18-01*01*	*TCRBD01-01*01*	*TCRBJ02-07*01*
CSARDLTSNEQHF	4	0.003117061	39	*TCRBV20*	*TCRBD02-01*	*TCRBJ02-07*01*
CASSCARD*RDYYEQYF	3	0.002337796	51	*TCRBV07-08*01*	*TCRBD02-01*02*	*TCRBJ02-07*01*
CARSLGGDSNQPQHF	2	0.001558531	45	*TCRBV05-01*01*	*TCRBD02-01*01*	*TCRBJ01-05*01*
CASRLGNSRKNYGYTF	2	0.001558531	48	*TCRBV05-07*01*	*unresolved*	*TCRBJ01-02*01*
CASSGILPISFPETQYF	2	0.001558531	51	*TCRBV03*	*unresolved*	*TCRBJ02-05*01*
CASSHLRPGGDYEQYF	2	0.001558531	48	*TCRBV07-06*01*	*TCRBD01-01*01*	*TCRBJ02-07*01*
CASSLSGTGVYEQYF	2	0.001558531	45	*TCRBV02-01*01*	*TCRBD01-01*01*	*TCRBJ02-07*01*
CASWWVPAWKTYGYTF	2	0.001558531	48	*TCRBV05-07*01*	*unresolved*	*TCRBJ01-02*01*
CSARGWAAGRGSYGQYF	2	0.001558531	51	*TCRBV20*	*TCRBD02-01*02*	*TCRBJ02-07*01*
CSASPVLQRDNEQFF	2	0.001558531	45	*TCRBV20*	*TCRBD02-01*	*TCRBJ02-01*01*
CSVEAVDSSYEQYL	2	0.001558531	42	*TCRBV29-01*01*	*TCRBD02-01*	*TCRBJ02*
CTSSVDCLCTSRANVLTF	2	0.001558531	54	*TCRBV01-01*01*	*TCRBD02-01*	*TCRBJ02-06*01*
**cDNA**						
**LESION IV**						
CSVFQDRGSSGELFF	752	3.191986078	45	*TCRBV29-01*01*	*TCRBD01-01*01*	*TCRBJ02-02*01*
CASNHAWVSNQPQHF	750	3.183496753	45	*TCRBV23-01*01*	*unresolved*	*TCRBJ01-05*01*
CASSRDDTQYF	392	1.663907636	33	*TCRBV07-09*	*TCRBD02-01*02*	*TCRBJ02-03*01*
CASSQVQAIDTQYF	382	1.621461013	42	*TCRBV04-01*01*	*TCRBD02-01*	*TCRBJ02-03*01*

CASSLSGTGVYEQYF	311	1.320089987	45	*TCRBV12*	*TCRBD01-01*01*	*TCRBJ02-07*01*
CASSVGGSYTF	286	1.213973428	33	*TCRBV05-06*01*	*TCRBD02-01*01*	*TCRBJ01-02*01*
CASSLGNGETQYF	280	1.188505454	39	*TCRBV05-06*01*	*unresolved*	*TCRBJ02-05*01*
CASSQGGRAEAFF	280	1.188505454	39	*TCRBV04-01*01*	*TCRBD01-01*01*	*TCRBJ01-01*01*
CASSGREGPGYTF	249	1.056920922	39	*TCRBV02-01*01*	*TCRBD02-01*02*	*TCRBJ01-02*01*
CASSEASPIVMQDTQYF	243	1.031452948	51	*TCRBV03*	*TCRBD02-01*	*TCRBJ02-03*01*
CASIPQAGINTEAFF	230	0.976272338	45	*TCRBV12*	*TCRBD01-01*01*	*TCRBJ01-01*01*
CASSYQGSETQYF	227	0.963538351	39	*TCRBV06-05*01*	*TCRBD01-01*01*	*TCRBJ02-05*01*
CASSASGGYNEQFF	223	0.946559701	42	*TCRBV06*	*TCRBD02-01*	*TCRBJ02-01*01*
CASSHRTSGGLVGNIQYF	220	0.933825714	54	*TCRBV03*	*TCRBD02-01*01*	*TCRBJ02-04*01*
CASSPARGTGANVLTF	218	0.925336389	48	*TCRBV28-01*01*	*TCRBD02-01*02*	*TCRBJ02-06*01*
CASSLVAGVPNEQFF	213	0.904113078	45	*TCRBV05-06*01*	*TCRBD02-01*02*	*TCRBJ02-01*01*
CASNPGTWNTEAFF	212	0.899868415	42	*TCRBV19-01*	*TCRBD01-01*01*	*TCRBJ01-01*01*
CASTDTGGRQYF	202	0.857421792	36	*TCRBV06-05*01*	*unresolved*	*TCRBJ02-07*01*
CSAGETSARGEQYF	193	0.819219831	42	*TCRBV29-01*01*	*TCRBD02-01*	*TCRBJ02-07*01*
CASIQDNVEGITGELFF	191	0.810730506	51	*TCRBV12*	*TCRBD02-01*02*	*TCRBJ02-02*01*
CSAFSTTLNEQFF	187	0.793751857	39	*TCRBV20*	*unresolved*	*TCRBJ02-01*01*
CASSIQEWSTEAFF	184	0.78101787	42	*TCRBV19-01*	*unresolved*	*TCRBJ01-01*01*
CAWSDGGREWPAIQYF	182	0.772528545	48	*TCRBV30-01*01*	*TCRBD02-01*02*	*TCRBJ02-04*01*
CASSMAGYDYEQYF	179	0.759794558	42	*TCRBV19-01*	*TCRBD02-01*01*	*TCRBJ02-07*01*
CASTRLAGEQYF	179	0.759794558	36	*TCRBV28-01*01*	*TCRBD02-01*	*TCRBJ02-07*01*
CASSLARGDTGELFF	178	0.755549896	45	*TCRBV11-02*02*	*TCRBD02-01*02*	*TCRBJ02-02*01*
CASSVVSPFVMQDTQYF	176	0.747060571	51	*TCRBV03*	*unresolved*	*TCRBJ02-03*01*
CASSLGGAGEFYEQYF	168	0.713103273	48	*TCRBV05-01*01*	*TCRBD02-01*02*	*TCRBJ02-07*01*
CASSPTGSVYEQYF	163	0.691879961	42	*TCRBV06-04*	*TCRBD01-01*01*	*TCRBJ02-07*01*
CASSSTGTNYGYTF	163	0.691879961	42	*TCRBV05-01*01*	*TCRBD01-01*01*	*TCRBJ01-02*01*
CASSPSGDGYNEQFF	161	0.683390636	45	*TCRBV07-09*	*TCRBD02-01*01*	*TCRBJ02-01*01*
CSVAPHTGSTGELFF	161	0.683390636	45	*TCRBV29-01*01*	*TCRBD01-01*01*	*TCRBJ02-02*01*
CASSSLGRVGEKLFF	159	0.674901312	45	*TCRBV07-02*01*	*TCRBD01-01*01*	*TCRBJ01-04*01*
CASSGRKTYGYTF	156	0.662167325	39	*TCRBV06-08*01*	*unresolved*	*TCRBJ01-02*01*
CSASKGMETQYF	155	0.657922662	36	*TCRBV20*	*TCRBD01-01*01*	*TCRBJ02-05*01*
CASSLAQGSTGELFF	153	0.649433338	45	*TCRBV13-01*01*	*TCRBD01-01*01*	*TCRBJ02-02*01*

CASSLLADQETQYF	151	0.640944013	42	*TCRBV05-01*01*	*TCRBD02-01*	*TCRBJ02-05*01*
CASQAGVTDTQYF	150	0.636699351	39	*TCRBV07-06*01*	*TCRBD01-01*01*	*TCRBJ02-03*01*
CASSYPRVGDSSGANVLTF	150	0.636699351	57	*TCRBV06*	*TCRBD02-01*	*TCRBJ02-06*01*
CASSFTGRAYQPQHF	149	0.632454688	45	*TCRBV28-01*01*	*unresolved*	*TCRBJ01-05*01*
CASSIGTASYNEQFF	149	0.632454688	45	*TCRBV28-01*01*	*TCRBD01-01*01*	*TCRBJ02-01*01*
CASSYSGGGYTF	146	0.619720701	36	*TCRBV06-06*	*unresolved*	*TCRBJ01-02*01*
CASSLLGRTSTDTQYF	145	0.615476039	48	*TCRBV12*	*unresolved*	*TCRBJ02-03*01*
CASSRQGQETQYF	145	0.615476039	39	*TCRBV25-01*01*	*TCRBD01-01*01*	*TCRBJ02-05*01*
CASSFAKQGGDHEQYF	143	0.606986714	48	*TCRBV07-06*01*	*TCRBD01-01*01*	*TCRBJ02-07*01*
CTSREDSLCTKRANVLTF	142	0.602742052	54	*TCRBV01-01*01*	*TCRBD02-01*02*	*TCRBJ02-06*01*
CASSPSGGQETQYF	139	0.590008065	42	*TCRBV12*	*TCRBD02-01*01*	*TCRBJ02-05*01*
CASSSQGAYEQYF	139	0.590008065	39	*TCRBV06-06*	*TCRBD01-01*01*	*TCRBJ02-07*01*
CAISDPNLDRYNEQFF	138	0.585763403	48	*TCRBV10-03*01*	*TCRBD01-01*01*	*TCRBJ02-01*01*
CASNLDRFNYGYTF	138	0.585763403	42	*TCRBV19-01*	*TCRBD01-01*01*	*TCRBJ01-02*01*
CASSQSAATGNYEQYF	138	0.585763403	48	*TCRBV07-06*01*	*TCRBD01-01*01*	*TCRBJ02-07*01*
CASRVSYEQYF	137	0.58151874	33	*TCRBV06*	*unresolved*	*TCRBJ02-07*01*
CASRTTGMGEEQYF	136	0.577274078	42	*TCRBV28-01*01*	*TCRBD01-01*01*	*TCRBJ02-07*01*
CASSQDPGSWNRGGGYTF	136	0.577274078	54	*TCRBV04-02*01*	*unresolved*	*TCRBJ01-02*01*
CASSVDGSNDEQFF	136	0.577274078	42	*TCRBV09-01*	*TCRBD02-01*	*TCRBJ02-01*01*
CSAITGTSGRRSYNEQFF	136	0.577274078	54	*TCRBV20*	*TCRBD02-01*02*	*TCRBJ02-01*01*
CASSWAGKGTDTQYF	134	0.568784753	45	*TCRBV19-01*	*TCRBD01-01*01*	*TCRBJ02-03*01*
CSVKTIYEQYF	133	0.564540091	33	*TCRBV29-01*01*	*TCRBD02-01*	*TCRBJ02-07*01*
CASSFGLLHIHHNSPLHF	132	0.560295428	54	*TCRBV21-01*01*	*unresolved*	*TCRBJ01-06*01*
CASSSTGDLYEQYF	132	0.560295428	42	*TCRBV05-04*01*	*TCRBD01-01*01*	*TCRBJ02-07*01*
CASSSSGGAVDTQYF	131	0.556050766	45	*TCRBV06-01*01*	*TCRBD02-01*01*	*TCRBJ02-03*01*
CSADPRGLAARDEQFF	131	0.556050766	48	*TCRBV20*	*TCRBD02-01*	*TCRBJ02-01*01*
CASSLALTDTQYF	130	0.551806104	39	*TCRBV07-08*01*	*unresolved*	*TCRBJ02-03*01*
CASSPISDGYYGYTF	130	0.551806104	45	*TCRBV04-01*01*	*unresolved*	*TCRBJ01-02*01*
CASSGRNKSQSRANVLTF	128	0.543316779	54	*TCRBV07-07*01*	*unresolved*	*TCRBJ02-06*01*
CASSYIGEGANVLTF	127	0.539072117	45	*TCRBV06*	*TCRBD02-01*01*	*TCRBJ02-06*01*
CATRPHDNQPQHF	127	0.539072117	39	*TCRBV24*	*unresolved*	*TCRBJ01-05*01*
CSGRADYGYTF	125	0.530582792	33	*TCRBV29-01*01*	*unresolved*	*TCRBJ01-02*01*

CASSHGQGATRNGYTF	124	0.52633813	48	*TCRBV12*	*TCRBD01-01*01*	*TCRBJ01-02*01*
CASSSGLAGDNEQFF	124	0.52633813	45	*TCRBV05-01*01*	*TCRBD02-01*02*	*TCRBJ02-01*01*
CASSIQLNTEAFF	123	0.522093467	39	*TCRBV07-09*	*TCRBD01-01*01*	*TCRBJ01-01*01*
CASSDKCRDGQSRANVLTF	122	0.517848805	57	*TCRBV07-02*01*	*TCRBD01-01*01*	*TCRBJ02-06*01*
CATSETGTRNNEQFF	122	0.517848805	45	*TCRBV24*	*TCRBD02-01*	*TCRBJ02-01*01*
CASRGDRSYEQYF	121	0.513604143	39	*TCRBV06-05*01*	*TCRBD01-01*01*	*TCRBJ02-07*01*
CASSSPTSGSPNEQFF	121	0.513604143	48	*TCRBV05-01*01*	*TCRBD02-01*02*	*TCRBJ02-01*01*
CASSPIGESWGEQYF	120	0.50935948	45	*TCRBV04-02*01*	*unresolved*	*TCRBJ02-07*01*
CASSSGPYGYTF	120	0.50935948	36	*TCRBV06-01*01*	*unresolved*	*TCRBJ01-02*01*
CAISGEEVPGAGTEAFF	119	0.505114818	51	*TCRBV10-03*01*	*unresolved*	*TCRBJ01-01*01*
CASLFNINEQYF	117	0.496625493	36	*TCRBV10-02*01*	*TCRBD02-01*	*TCRBJ02-07*01*
CASSQVEETQYF	116	0.492380831	36	*TCRBV03*	*TCRBD02-01*	*TCRBJ02-05*01*
CASRLQQNTEAFF	113	0.479646844	39	*TCRBV06-06*	*unresolved*	*TCRBJ01-01*01*
CASSTGTGSYGYTF	113	0.479646844	42	*TCRBV09-01*	*TCRBD01-01*01*	*TCRBJ01-02*01*
CASTWTGISNQPQHF	113	0.479646844	45	*TCRBV19-01*	*TCRBD01-01*01*	*TCRBJ01-05*01*
CSVDQGAYNNEQFF	113	0.479646844	42	*TCRBV29-01*01*	*TCRBD01-01*01*	*TCRBJ02-01*01*
CASRRGAVETQYF	112	0.475402182	39	*TCRBV06-05*01*	*unresolved*	*TCRBJ02-05*01*
CASSFSGNQPQHF	112	0.475402182	39	*TCRBV07-02*01*	*unresolved*	*TCRBJ01-05*01*
CASSPKVGSGYTF	111	0.471157519	39	*TCRBV19-01*	*TCRBD01-01*01*	*TCRBJ01-02*01*
CASSFLLLSDTQYF	109	0.462668195	42	*TCRBV11-02*02*	*unresolved*	*TCRBJ02-03*01*
CSARTRGLDSGNTIYF	108	0.458423532	48	*TCRBV20*	*unresolved*	*TCRBJ01-03*01*
CASSLGPAGGYEQYF	105	0.445689545	45	*TCRBV27-01*01*	*TCRBD02-01*02*	*TCRBJ02-07*01*
CASSQQRLGRGRETQYF	105	0.445689545	51	*TCRBV14-01*01*	*TCRBD02-01*01*	*TCRBJ02-05*01*
CAWSVPAGNGANVLTF	105	0.445689545	48	*TCRBV30-01*01*	*TCRBD01-01*01*	*TCRBJ02-06*01*
CASSQAFGVPGELFF	104	0.441444883	45	*TCRBV04-02*01*	*unresolved*	*TCRBJ02-02*01*
CASSFRNSPLHF	102	0.432955558	36	*TCRBV27-01*01*	*TCRBD02-01*	*TCRBJ01-06*01*
CASSPGGGRANEQFF	101	0.428710896	45	*TCRBV10-02*01*	*TCRBD02-01*02*	*TCRBJ02-01*01*
CASSTYGQGLYEQYF	100	0.424466234	45	*TCRBV05-04*01*	*TCRBD01-01*01*	*TCRBJ02-07*01*
CASTAGGTSRPQHF	100	0.424466234	42	*TCRBV02-01*01*	*TCRBD02-01*	*TCRBJ01-05*01*
CASSDAYEQYF	99	0.420221571	33	*TCRBV28-01*01*	*unresolved*	*TCRBJ02-07*01*
CATSEQGARNNEQFF	99	0.420221571	45	*TCRBV24*	*TCRBD01-01*01*	*TCRBJ02-01*01*
CSAGDLSGNTIYF	98	0.415976909	39	*TCRBV20*	*TCRBD02-01*02*	*TCRBJ01-03*01*

CSVEISKGTGNYGYTF	98	0.415976909	48	*TCRBV29-01*01*	*TCRBD01-01*01*	*TCRBJ01-02*01*
CAIRAGTGAFF	97	0.411732247	33	*TCRBV10-03*01*	*unresolved*	*TCRBJ01-01*01*
CASSQEGVRETQYF	96	0.407487584	42	*TCRBV03*	*TCRBD01-01*01*	*TCRBJ02-05*01*
CASVNGQADTEAFF	96	0.407487584	42	*TCRBV28-01*01*	*TCRBD01-01*01*	*TCRBJ01-01*01*
CASSLYRGGTQYF	91	0.386264273	39	*TCRBV05-06*01*	*TCRBD01-01*01*	*TCRBJ02-03*01*
CASSYNRGDRGGYEQYF	91	0.386264273	51	*TCRBV18-01*01*	*TCRBD01-01*01*	*TCRBJ02-07*01*
CASSLDSYEKLFF	90	0.38201961	39	*TCRBV07-02*01*	*TCRBD01-01*01*	*TCRBJ01-04*01*
CASSRTRTGAYEQYF	90	0.38201961	45	*TCRBV11-01*01*	*TCRBD02-01*01*	*TCRBJ02-07*01*
CASSMWQLDTQYF	88	0.373530286	39	*TCRBV19-01*	*TCRBD01-01*01*	*TCRBJ02-03*01*
CASSPMLVQQRKNIQYF	87	0.369285623	51	*TCRBV21-01*01*	*TCRBD01-01*01*	*TCRBJ02-04*01*
CASSRIERTSTDTQYF	87	0.369285623	48	*TCRBV12*	*TCRBD02-01*	*TCRBJ02-03*01*
CASSSVAGGPYEQYF	86	0.365040961	45	*TCRBV07-09*	*TCRBD02-01*02*	*TCRBJ02-07*01*
CASSIGGGITGELFF	85	0.360796299	45	*TCRBV19-01*	*TCRBD01-01*01*	*TCRBJ02-02*01*
CASSSTLGAYEQYF	84	0.356551636	42	*TCRBV07-09*	*TCRBD02-01*	*TCRBJ02-07*01*
CSAISGVAGPNEQFF	84	0.356551636	45	*TCRBV20*	*TCRBD02-01*	*TCRBJ02-01*01*
CASSMYQNTEAFF	83	0.352306974	39	*TCRBV19-01*	*unresolved*	*TCRBJ01-01*01*
CASSPSMNTEAFF	83	0.352306974	39	*TCRBV11-02*02*	*unresolved*	*TCRBJ01-01*01*
CASSFGQAASPLHF	82	0.348062312	42	*TCRBV12*	*unresolved*	*TCRBJ01-06*01*
CASSLAYRDESEQYF	82	0.348062312	45	*TCRBV05-06*01*	*TCRBD02-01*02*	*TCRBJ02-07*01*
CSARGPGVGETQYF	80	0.339572987	42	*TCRBV20*	*TCRBD02-01*01*	*TCRBJ02-05*01*
CASSQEVGRLSYNEQFF	79	0.335328325	51	*TCRBV04-01*01*	*TCRBD01-01*01*	*TCRBJ02-01*01*
CASSLYFSPNTDTQYF	78	0.331083662	48	*TCRBV07-03*01*	*TCRBD02-01*	*TCRBJ02-03*01*
CASSLARGGNEQFF	77	0.326839	42	*TCRBV07-02*01*	*TCRBD01-01*01*	*TCRBJ02-01*01*
CASSSERHSTYGYTF	77	0.326839	45	*TCRBV05-01*01*	*TCRBD01-01*01*	*TCRBJ01-02*01*
CASSTGTGGSDTQYF	77	0.326839	45	*TCRBV05-05*01*	*TCRBD01-01*01*	*TCRBJ02-03*01*
CASSLFTGDEAFF	75	0.318349675	39	*TCRBV27-01*01*	*TCRBD01-01*01*	*TCRBJ01-01*01*
CASSSGLAGDEQFF	74	0.314105013	42	*TCRBV07-03*01*	*TCRBD02-01*	*TCRBJ02-01*01*
CSVHTDGNQETQYF	74	0.314105013	42	*TCRBV29-01*01*	*unresolved*	*TCRBJ02-05*01*
CASNPGTAYSYEQYF	73	0.309860351	45	*TCRBV06-01*01*	*TCRBD01-01*01*	*TCRBJ02-07*01*
CASRVDRGQIGTGELFF	73	0.309860351	51	*TCRBV02-01*01*	*TCRBD01-01*01*	*TCRBJ02-02*01*
CASRRLLQGSEQFF	72	0.305615688	42	*TCRBV12*	*TCRBD01-01*01*	*TCRBJ02-01*01*
CASSHNLEAGKTYGYTF	72	0.305615688	51	*TCRBV11-01*01*	*TCRBD02-01*02*	*TCRBJ01-02*01*

CAISESSSLGTSGTDTQYF	71	0.301371026	57	*TCRBV10-03*01*	*TCRBD02-01*	*TCRBJ02-03*01*
CASRPPSDRWDYGYTF	70	0.297126364	48	*TCRBV28-01*01*	*unresolved*	*TCRBJ01-02*01*
CASSFLRGPKRYNEQFF	70	0.297126364	51	*TCRBV12*	*unresolved*	*TCRBJ02-01*01*
CASSPWYEQYF	70	0.297126364	33	*TCRBV28-01*01*	*unresolved*	*TCRBJ02-07*01*
CSFRQGAGNEQYF	70	0.297126364	39	*TCRBV29-01*01*	*TCRBD01-01*01*	*TCRBJ02-07*01*
CASKQGSGANVLTF	67	0.284392377	42	*TCRBV05-01*01*	*TCRBD01-01*01*	*TCRBJ02-06*01*
CASSSPNRVSGNTIYF	67	0.284392377	48	*TCRBV07-09*	*TCRBD01-01*01*	*TCRBJ01-03*01*
CASSWTSGGYNEQFF	67	0.284392377	45	*TCRBV19-01*	*TCRBD02-01*01*	*TCRBJ02-01*01*
CASSPPGRYGYTF	66	0.280147714	39	*TCRBV04-01*01*	*TCRBD02-01*02*	*TCRBJ01-02*01*
CASSGLEDWGNTDTQYF	65	0.275903052	51	*TCRBV05-06*01*	*unresolved*	*TCRBJ02-03*01*
CASSPEGRTPQPQHF	65	0.275903052	45	*TCRBV19-01*	*TCRBD02-01*02*	*TCRBJ01-05*01*
CATPSRGENTIYF	65	0.275903052	39	*TCRBV07-03*01*	*TCRBD01-01*01*	*TCRBJ01-03*01*
CASSSRIHGTYSTDTQYF	64	0.27165839	54	*TCRBV07-09*	*TCRBD02-01*	*TCRBJ02-03*01*
CSAREGATDTQYF	64	0.27165839	39	*TCRBV20*	*TCRBD01-01*01*	*TCRBJ02-03*01*
CASGAEEQGFSFHEQYF	62	0.263169065	51	*TCRBV12*	*TCRBD01-01*01*	*TCRBJ02-07*01*
CASSAGATMDSNSGNTIYF	62	0.263169065	57	*TCRBV02-01*01*	*TCRBD01-01*01*	*TCRBJ01-03*01*
CSVEGQGVAETQYF	62	0.263169065	42	*TCRBV29-01*01*	*TCRBD01-01*01*	*TCRBJ02-05*01*
CASTVSNSPLHF	61	0.258924403	36	*TCRBV13-01*01*	*TCRBD02-01*	*TCRBJ01-06*01*
CASSLDGTGGEQYF	60	0.25467974	42	*TCRBV11-02*02*	*TCRBD01-01*01*	*TCRBJ02-07*01*
CASSAARLASYNEQFF	59	0.250435078	48	*TCRBV05-06*01*	*TCRBD02-01*	*TCRBJ02-01*01*
CASGAGGSYNEQFF	58	0.246190416	42	*TCRBV07-06*01*	*unresolved*	*TCRBJ02-01*01*
CASSFYPRTDTQYF	57	0.241945753	42	*TCRBV27-01*01*	*TCRBD02-01*	*TCRBJ02-03*01*
CASSFYRQGWQETQYF	57	0.241945753	48	*TCRBV27-01*01*	*TCRBD01-01*01*	*TCRBJ02-05*01*
CASSLMAGDLSYEQYF	57	0.241945753	48	*TCRBV12*	*TCRBD01-01*01*	*TCRBJ02-07*01*
CASSRGTGEGQETQYF	57	0.241945753	48	*TCRBV19-01*	*unresolved*	*TCRBJ02-05*01*
CASSVGGLASDTQYF	57	0.241945753	45	*TCRBV09-01*	*TCRBD02-01*	*TCRBJ02-03*01*
CSASLISGADTQYF	57	0.241945753	42	*TCRBV20*	*TCRBD02-01*	*TCRBJ02-03*01*
CASSISTGTSATYEQYF	55	0.233456429	51	*TCRBV05-01*01*	*TCRBD02-01*	*TCRBJ02-07*01*
CASSSSAVAGRRVYEQYF	55	0.233456429	54	*TCRBV07-09*	*TCRBD02-01*01*	*TCRBJ02-07*01*
CASSLGGARNTEAFF	54	0.229211766	45	*TCRBV27-01*01*	*TCRBD02-01*01*	*TCRBJ01-01*01*
CASSQDRVTGYTF	54	0.229211766	39	*TCRBV19-01*	*TCRBD01-01*01*	*TCRBJ01-02*01*
CSALGQGNADEQFF	54	0.229211766	42	*TCRBV20*	*TCRBD01-01*01*	*TCRBJ02-01*01*

CASSMARALSHGSYNEQFF	53	0.224967104	57	*TCRBV19-01*	*TCRBD02-01*	*TCRBJ02-01*01*
CATPDEGGGRNYGYTF	53	0.224967104	48	*TCRBV28-01*01*	*TCRBD02-01*01*	*TCRBJ01-02*01*
CASSGTRDPYNEQFF	52	0.220722442	45	*TCRBV09-01*	*TCRBD02-01*	*TCRBJ02-01*01*
CASSLDVGGYTF	52	0.220722442	36	*TCRBV05-01*01*	*TCRBD02-01*	*TCRBJ01-02*01*
CASSLGTTFYNEQFF	52	0.220722442	45	*TCRBV05-01*01*	*TCRBD02-01*	*TCRBJ02-01*01*
CASSLNTGELFF	52	0.220722442	36	*TCRBV05-01*01*	*unresolved*	*TCRBJ02-02*01*
CASSAQGGIGTIYEQYF	51	0.216477779	51	*TCRBV07-08*01*	*TCRBD01-01*01*	*TCRBJ02-07*01*
CASSARRTSGSTDTQYF	51	0.216477779	51	*TCRBV02-01*01*	*TCRBD02-01*	*TCRBJ02-03*01*
CASSLFGGGAAGELFF	51	0.216477779	48	*TCRBV27-01*01*	*unresolved*	*TCRBJ02-02*01*
CASSPRDRGLGYTF	51	0.216477779	42	*TCRBV28-01*01*	*TCRBD01-01*01*	*TCRBJ01-02*01*
CASSLYGSHTEAFF	50	0.212233117	42	*TCRBV07-02*01*	*unresolved*	*TCRBJ01-01*01*
YSSAYGILGQSRANVLTF	50	0.212233117	54	*TCRBV17-01*01*	*TCRBD01-01*01*	*TCRBJ02-06*01*
CSAGTSGTETQYF	49	0.207988455	39	*TCRBV20*	*TCRBD02-01*02*	*TCRBJ02-05*01*
CASSGGHQSSYEQYF	48	0.203743792	45	*TCRBV05-01*01*	*TCRBD01-01*01*	*TCRBJ02-07*01*
CASSLISRDWLFF	48	0.203743792	39	*TCRBV11-02*02*	*TCRBD02-01*	*TCRBJ02-02*01*
CASSPQDRGLRDGYTF	48	0.203743792	48	*TCRBV27-01*01*	*TCRBD01-01*01*	*TCRBJ01-02*01*
CASSGPTTGGLSYEQYF	47	0.19949913	51	*TCRBV10-02*01*	*TCRBD01-01*01*	*TCRBJ02-07*01*
CASAGYNEQFF	46	0.195254468	33	*TCRBV12-05*01*	*TCRBD02-01*	*TCRBJ02-01*01*
CASGPEGLATYNEQFF	45	0.191009805	48	*TCRBV12-05*01*	*TCRBD02-01*	*TCRBJ02-01*01*
CASSPQGLAGLQQETQYF	45	0.191009805	54	*TCRBV28-01*01*	*TCRBD02-01*	*TCRBJ02-05*01*
CSVEGQGASETQYF	45	0.191009805	42	*TCRBV29-01*01*	*TCRBD01-01*01*	*TCRBJ02-05*01*
CASSQRPRNFEAFF	44	0.186765143	42	*TCRBV23-01*01*	*unresolved*	*TCRBJ01-01*01*
CSVKPGGIQETQYF	44	0.186765143	42	*TCRBV29-01*01*	*TCRBD01-01*01*	*TCRBJ02-05*01*
YASSFYRQGWQETQYF	43	0.18252048	48	*TCRBV27-01*01*	*TCRBD01-01*01*	*TCRBJ02-05*01*
CAISVLRSTDTQYF	42	0.178275818	42	*TCRBV10-03*01*	*TCRBD02-01*	*TCRBJ02-03*01*
CSVAGRVSNEQFF	41	0.174031156	39	*TCRBV29-01*01*	*TCRBD01-01*01*	*TCRBJ02-01*01*
CASSLSGTEGEQYF	40	0.169786493	42	*TCRBV28-01*01*	*TCRBD01-01*01*	*TCRBJ02-07*01*
CASINRSSGRFWSETQYF	39	0.165541831	54	*TCRBV28-01*01*	*TCRBD02-01*02*	*TCRBJ02-05*01*
CASSLGSFEQYF	39	0.165541831	36	*TCRBV11-02*02*	*unresolved*	*TCRBJ02-07*01*
CASSQVRLNDEQFF	39	0.165541831	42	*TCRBV04-01*01*	*TCRBD02-01*	*TCRBJ02-01*01*
CASSESRGGHTQYF	38	0.161297169	42	*TCRBV10-02*01*	*TCRBD02-01*01*	*TCRBJ02-03*01*
CASSASGGITDTQYF	36	0.152807844	45	*TCRBV05-04*01*	*TCRBD02-01*01*	*TCRBJ02-03*01*

CASSHGQSDQPQHF	35	0.148563182	42	*TCRBV07-02*01*	*TCRBD01-01*01*	*TCRBJ01-05*01*
CASSTPSSARKIYGYTF	35	0.148563182	51	*TCRBV07-04*01*	*unresolved*	*TCRBJ01-02*01*
CSASRPNTGDRSEKLFF	35	0.148563182	51	*TCRBV20*	*TCRBD01-01*01*	*TCRBJ01-04*01*
CASSCKIAKTYGYTF	34	0.144318519	45	*TCRBV10-01*	*unresolved*	*TCRBJ01-02*01*
CSASLSDPEQYF	34	0.144318519	36	*TCRBV20*	*unresolved*	*TCRBJ02-07*01*
CSAVNTGELFF	34	0.144318519	33	*TCRBV20-01*01*	*TCRBD02-01*	*TCRBJ02-02*01*
CASSRGGNSYEQYF	33	0.140073857	42	*TCRBV07-06*01*	*TCRBD02-01*02*	*TCRBJ02-07*01*
CSAFAVRGGITDTQYF	30	0.12733987	48	*TCRBV20*	*TCRBD02-01*01*	*TCRBJ02-03*01*
CASGDYEQYF	29	0.123095208	30	*TCRBV28-01*01*	*unresolved*	*TCRBJ02-07*01*
CASSFGQDAFF	29	0.123095208	33	*TCRBV05-06*01*	*unresolved*	*TCRBJ02-01*01*
CASSDLPQTSGRYGKTQYF	25	0.106116558	57	*TCRBV02-01*01*	*TCRBD02-01*02*	*TCRBJ02-05*01*
CASSLWTHITDNEQFF	23	0.097627234	48	*TCRBV28-01*01*	*unresolved*	*TCRBJ02-01*01*
CASSRQGFDLGNTEAFF	23	0.097627234	51	*TCRBV11-02*02*	*TCRBD01-01*01*	*TCRBJ01-01*01*
CASSTPSRGMNTEAFF	22	0.093382571	48	*TCRBV07-09*	*TCRBD01-01*01*	*TCRBJ01-01*01*
CSAPWGNEQFF	22	0.093382571	33	*TCRBV20-01*01*	*unresolved*	*TCRBJ02-01*01*
CAITPGPSQGGQETQYF	21	0.089137909	51	*TCRBV10-03*01*	*TCRBD01-01*01*	*TCRBJ02-05*01*
CASSLFDRGGNEKLFF	20	0.084893247	48	*TCRBV28-01*01*	*TCRBD02-01*02*	*TCRBJ01-04*01*
CASSPDSDWETQYF	20	0.084893247	42	*TCRBV07-09*	*TCRBD02-01*	*TCRBJ02-05*01*
CASSPPGTKNTGELFF	19	0.080648584	48	*TCRBV11-02*02*	*TCRBD02-01*	*TCRBJ02-02*01*
CASSPPGIFSYNEQFF	18	0.076403922	48	*TCRBV18-01*01*	*unresolved*	*TCRBJ02-01*01*
CASSGIPYEGKTYGYTF	17	0.07215926	51	*TCRBV07-01*01*	*TCRBD02-01*02*	*TCRBJ01-02*01*
CASSKLMATVMQDTQYF	15	0.063669935	51	*TCRBV21-01*01*	*TCRBD01-01*01*	*TCRBJ02-03*01*
CASKGGTGIYNEQFF	13	0.05518061	45	*TCRBV02-01*01*	*TCRBD01-01*01*	*TCRBJ02-01*01*
CASSEYGTSGYTF	12	0.050935948	39	*TCRBV25-01*01*	*TCRBD01-01*01*	*TCRBJ01-02*01*
CSARVGQGSSYNSPLHF	12	0.050935948	51	*TCRBV20*	*TCRBD01-01*01*	*TCRBJ01-06*01*
CASSPPGAGYGYTF	11	0.046691286	42	*TCRBV06-05*01*	*unresolved*	*TCRBJ01-02*01*
CATSRLTARGWGDTQYF	10	0.042446623	51	*TCRBV15-01*01*	*TCRBD02-01*01*	*TCRBJ02-03*01*
CASSLPERAETQYF	9	0.038201961	42	*TCRBV28-01*01*	*TCRBD02-01*	*TCRBJ02-05*01*
CASSGGEPLRGWNEQFF	8	0.033957299	51	*TCRBV18-01*01*	*TCRBD02-01*01*	*TCRBJ02-01*01*
CASSPQGLAGLQQGTQYF	7	0.029712636	54	*TCRBV28-01*01*	*TCRBD02-01*	*TCRBJ02-05*01*
CASSARGDHPLHNEQFF	6	0.025467974	51	*TCRBV27-01*01*	*TCRBD02-01*	*TCRBJ02-01*01*
CASSCRVSISIRTEAFF	6	0.025467974	51	*TCRBV03*	*TCRBD01-01*01*	*TCRBJ01-01*01*

CATSRLTARGWGGTQYF	6	0.025467974	51	*TCRBV15-01*01*	*TCRBD02-01*01*	*TCRBJ02-03*01*
CASSTPGRGMNTEAFF	5	0.021223312	48	*TCRBV07-09*	*TCRBD01-01*01*	*TCRBJ01-01*01*
CSARSGESGEQFF	5	0.021223312	39	*TCRBV20*	*TCRBD02-01*	*TCRBJ02-01*01*
CASSLGSFEQYF	4	0.016978649	36	*TCRBV11-02*02*	*TCRBD02-01*02*	*TCRBJ02-05*01*
CASSGASSTDTQYF	3	0.012733987	42	*TCRBV07-06*01*	*TCRBD02-01*02*	*TCRBJ02-03*01*
CASDLMHSRKTYGYTF	2	0.008489325	48	*TCRBV09-01*	*unresolved*	*TCRBJ01-02*01*
CASLYCIPGKTYGYTF	2	0.008489325	48	*TCRBV05-04*01*	*TCRBD01-01*01*	*TCRBJ01-02*01*
CASRRRPVRKTYGYTF	2	0.008489325	48	*TCRBV05-01*01*	*unresolved*	*TCRBJ01-02*01*
CASSTLSRGMNTEAFF	2	0.008489325	48	*TCRBV07-09*	*TCRBD01-01*01*	*TCRBJ01-01*01*

* CSF-TCC13.1-12 expanded in vitro from the CSF.

Table SII. Top T cell clonotypes in each sample

TOP 10 CLONOTYPES IN:		gDNA LESION I %	Rank	gDNA LESION IV %	Rank	gDNA LESION III %	Rank	gDNA CD4 %	Rank	gDNA CD8 %	Rank	cDNA LESION I %	Rank	cDNA LESION IV %	Rank	cDNA LESION III %	Rank
**LESION I gDNA**	CASNPGTAYSYEQYF*	4.01728	1	0.96239	9	9.32834	1			0.01891	550	6.10546	1	0.30986	129	0.00066	62
	CASSIQEWSTEAFF	3.03964	2	1.10578	5	0.75944	18			2.77023	3	3.65402	4	0.78102	22		
	CASSSGTNAYGYTF	2.51026	3	1.52113	4	1.15988	10			1.40326	7						
	CASSLSGTGVYEQYF*	2.26550	4	2.83730	2	3.07525	3	0.01090	2571			0.92507	37	1.32009	5	5.53934	6
	CASGDYEQYF	1.54401	5	0.16053	178					0.19173	48	0.62442	51	0.12310	205		
	CASSYNRGDRGGYEQYF	1.33482	6	3.27759	1	3.51317	2			1.24928	8			0.38626	106		
	CASNHAWVSNQPQHF	1.12919	7	2.02609	3	2.34540	4	0.01612	1174					3.18350	2		
	CSAGTIDLNTEAFF	1.04025	8			0.40043	67			0.03755	285	0.83256	39			0.04257	25
	CASSLFDRGGNEKLFF	0.87304	9	0.97096	8	0.38268	74			2.04021	4	2.10453	15	0.08489	213		
	CASSFPSTDTQYF	0.80829	10	0.66082	15	1.60174	6	0.00136	10957			2.08141	16				
	SUM FREQUENCIES	**18.56229**		**13.52259**		**22.56633**		**0.02838**		**7.71117**		**16.32747**		**6.18872**		**5.58256**	
	% CLONOTYPES AMONG 10% MOST FREQUENT**		**100**		**80**		**70**		**10**		**70**		**20**		**30**		**10**
	SUM FREQUENCIES CD4 CLONOTYPES	**4.20298**		**5.52421**		**7.02239**						**3.00648**		**4.50359**		**5.53934**	
	SUM FREQUENCIES CD8 CLONOTYPES	**14.35931**		**7.99838**		**15.54394**						**13.32100**		**1.68513**		**0.04323**	
**LESION IV gDNA**	CASSYNRGDRGGYEQYF	1.33482	6	3.27759	1	3.51317	2			1.24928	8			0.38626	106		
	CASSLSGTGVYEQYF*	2.26550	4	2.83730	2	3.07525	3	0.01090	2571			0.92507	37	1.32009	5	5.53934	6
	CASNHAWVSNQPQHF	1.12919	7	2.02609	3	2.34540	4	0.01612	1174					3.18350	2		
	CASSSGTNAYGYTF	2.51026	3	1.52113	4	1.15988	10			1.40326	7						
	CASSIQEWSTEAFF	3.03964	2	1.10578	5	0.75944	18			2.77023	3	3.65402	4	0.78102	22		
	CASSMAGYDYEQYF	0.13590	178	1.05980	6					0.01532	653			0.75979	24		
	CASSSPNRVSGNTIYF	0.03913	599	0.98265	7					0.17056	56			0.28439	139		
	CASSLFDRGGNEKLFF	0.87304	9	0.97096	8	0.38268	74			2.04021	4	2.10453	15	0.08489	213		
	CASNPGTAYSYEQYF*	4.01728	1	0.96239	9	9.32834	1			0.01891	550	6.10546	1	0.30986	129	0.00066	62
	CASNRGQVEKLFF	0.05052	495	0.85174	10			0.00795	3988								
	SUM FREQUENCIES	**15.39529**		**15.59544**		**20.56416**		**0.03497**		**7.66777**		**12.78908**		**7.10981**		**5.54000**	
	% CLONOTYPES AMONG 10% MOST FREQUENT**		**80**		**100**		**60**		**10**		**70**		**20**		**40**		**10**
	SUM FREQUENCIES CD4 CLONOTYPES	**3.44521**		**5.71513**		**5.42065**						**0.92507**		**4.50359**		**5.53934**	
	SUM FREQUENCIES CD8 CLONOTYPES	**11.95008**		**9.88030**		**15.14351**						**11.86401**		**2.60622**		**0.00066**	
**LESION III gDNA**	CASNPGTAYSYEQYF*	4.01728	1	0.96239	9	9.32834	1			0.01891	550	6.10546	1	0.30986	129	0.00066	62
	CASSYNRGDRGGYEQYF	1.33482	6	3.27759	1	3.51317	2			1.24928	8			0.38626	106		
	CASSLSGTGVYEQYF*	2.26550	4	2.83730	2	3.07525	3	0.01090	2571			0.92507	37	1.32009	5	5.53934	6
	CASNHAWVSNQPQHF	1.12919	7	2.02609	3	2.34540	4	0.01612	1174					3.18350	2		
	CASRPPSDRWDYGYTF	0.25971	56	0.59068	20	2.19548	5			0.01108	842			0.29713	134		
	CASSFPSTDTQYF	0.80829	10	0.66082	15	1.60174	6	0.00136	10957			2.08141	16				
	CASSFSGRSDEQYF	0.03913	594	0.31249	48	1.38081	7			0.00498	1778						
	CASSPDRVVGYTF			0.09975	342	1.34727	8	0.00454	6016								
	CASSFGQAASPLHF	0.36572	34	0.82680	13	1.21314	9	0.01339	1777					0.34806	118		
	CASSSGTNAYGYTF	2.51026	3	1.52113	4	1.15988	10			1.40326	7						
	SUM FREQUENCIES	**12.72991**		**13.11504**		**27.16047**		**0.04631**		**2.68751**		**9.11193**		**5.84490**		**5.54000**	
	% CLONOTYPES AMONG 10% MOST FREQUENT**		**80**		**90**		**100**		**20**		**40**		**10**		**20**		**10**
	SUM FREQUENCIES CD4 CLONOTYPES	**4.56871**		**6.45076**		**9.58280**						**3.00648**		**4.85165**		**5.53934**	
	SUM FREQUENCIES CD8 CLONOTYPES	**8.16120**		**6.66428**		**17.57767**						**6.10546**		**0.99325**		**0.00066**	
**MEM CD4 gDNA**	CASSLGQGLHEQYF			0.40911	29	0.59769	34	1.10331	1								
	CAISESDYGYTF							0.61023	2								
	CASSLRQGGSPLHF	0.06475	407					0.44745	3								
	CSATQGGNQPQHF	0.08325	331			0.24263	147	0.43588	4								
	CSARESSHSGANVLTF	0.33371	40	0.61016	18			0.30557	5			0.83256	40				
	CASSQHNQPQHF							0.29013	6								
	CSASPGGNQPQHF					0.05720	233	0.27674	7								
	CASSEGGNKNIQYF	0.07186	374	0.03195	586			0.20227	8								
	CASKLQARQETQYF							0.19910	9								
	CASSPPGRGSYEQYF	0.52226	20			0.32153	103	0.18911	10								
	SUM FREQUENCIES	**1.07583**		**1.05123**		**1.21906**		**4.05977**				**0.83256**					
**MEM CD8 gDNA**	CAWSVPAGNGANVLTF	0.59270	17	0.24781	82	0.29391	121			16.15693	1			0.44569	92		
	CASSVLEGTDTQYF	0.19354	99	0.35534	37	0.42805	62			7.08623	2						
	CASSIQEWSTEAFF	3.03964	2	1.10578	5	0.75944	18			2.77023	3	3.65402	4	0.78102	22		
	CASSLFDRGGNEKLFF	0.87304	9	0.97096	8	0.38268	74			2.04021	4	2.10453	15	0.08489	213		
	CASSLAYRDESEQYF	0.30169	45			0.55035	40			1.73059	5			0.34806	119		
	CASSSRDLRTQPQHF									1.63891	6						
	CASSSGTNAYGYTF	2.51026	3	1.52113	4	1.15988	10			1.40326	7						
	CASSYNRGDRGGYEQYF	1.33482	6	3.27759	1	3.51317	2			1.24928	8			0.38626	106		
	CASSLSRGGRNNEQFF									1.05019	9						
	CASSPQAGSPGNTIYF			0.48938	21					0.99294	10						
	SUM FREQUENCIES	**8.84569**		**7.96799**		**7.08748**				**36.11876**		**5.75856**		**2.04593**			
**LESION I cDNA**	CASNPGTAYSYEQYF*	4.01728	1	0.96239	9	9.32834	1			0.01891	550	6.10546	1	0.30986	129	0.00066	62
	CASSQGTGGIGNSPLHF	0.59341	16	0.16988	165	0.41030	66			0.20228	41	5.71230	2				
	CASSSAGYNTGELFF											3.79278	3				
	CASSIQEWSTEAFF	3.03964	2	1.10578	5	0.75944	18			2.77023	3	3.65402	4	0.78102	22		
	CAIRTGSGDTEAFF											3.49214	5				
	CAISEAGGRDTQYF	0.02063	828							0.00644	1379	3.05273	6				
	CASSRGDRGRNTEAFF	0.11242	228	0.12702	260					0.04379	256	3.00648	7				
	CASRPTRVDTGELFF							0.00159	9554			2.89084	8				
	CASSLFTGDEAFF	0.42051	27	0.03663	575					0.09759	107	2.47456	9	0.31835	126		
	CASSWTNTEAFF											2.38205	10				
	SUM FREQUENCIES	**8.20389**		**2.40170**		**10.49808**		**0.00159**		**3.13924**		**36.56337**		**1.40923**		**0.00066**	
	% CLONOTYPES AMONG 10% MOST FREQUENT**		**40**		**20**		**20**		**0**		**50**		**100**		**10**		**0**
	SUM FREQUENCIES CD4 CLONOTYPES	**0.00000**		**0.00000**		**0.00000**						**2.89084**		**0.00000**		**0.00000**	
	SUM FREQUENCIES CD8 CLONOTYPES	**8.20389**		**2.40170**		**10.49808**						**24.00555**		**1.40923**		**0.00066**	
**LESION IV cDNA**	CSVFQDRGSSGELFF*	0.51088	21	0.24391	84	0.09271	220			0.00438	2004	1.01758	34	3.19199	1	11.83341	4
	CASNHAWVSNQPQHF	1.12919	7	2.02609	3	2.34540	4	0.01612	1174					3.18350	2		
	CASSRDDTQYF	0.04838	516							0.14489	68			1.66391	3		
	CASSQVQAIDTQYF	0.41269	29	0.27352	68							1.36448	23	1.62146	4	0.00527	35
	CASSLSGTGVYEQYF*	2.26550	4	2.83730	2	3.07525	3	0.01090	2571			0.92507	37	1.32009	5	5.53934	6
	CASSVGGSYTF	0.07186	377	0.18313	145					0.00524	1693			1.21397	6		
	CASSLGNGETQYF	0.07827	355					0.00045	21608					1.18851	7		
	CASSQGGRAEAFF	0.05479	470	0.03429	581					0.71775	12			1.18851	8		
	CASSGREGPGYTF	0.11740	217	0.21586	104									1.05692	9		
	CASSEASPIVMQDTQYF													1.03145	10		
	SUM FREQUENCIES	**4.68896**		**5.81410**		**5.51336**		**0.02747**		**0.87226**		**3.30712**		**16.66030**		**17.37802**	
	% CLONOTYPES AMONG 10% MOST FREQUENT**		**40**		**30**		**20**		**10**		**20**		**0**		**100**		**20**
	SUM FREQUENCIES CD4 CLONOTYPES	**3.47296**		**4.86339**		**5.42065**						**0.92507**		**5.69209**		**5.539339**	
	SUM FREQUENCIES CD8 CLONOTYPES	**0.68591**		**0.46133**		**0.09271**						**1.01758**		**7.25837**		**11.83341**	
**LESION III cDNA**	CSASEGVYEQYF									0.00166	5617	0.23127	82			25.11251	1
	CASSSGPGEAF															20.60861	2
	CSVEISKGTGNYGYTF	0.10958	236	0.20573	114							0.37003	66	0.41598	101	20.01202	3
	CSVFQDRGSSGELFF*	0.51088	21	0.24391	84	0.09271	220			0.00438	2004	1.01758	34	3.19199	1	11.83341	4
	CSAQGLAGGHVDEQFF	0.02063	836	0.10598	318					0.57206	14	0.37003	65			7.08703	5
	CASSLSGTGVYEQYF*	2.26550	4	2.83730	2	3.07525	3	0.01090	2571			0.92507	37	1.32009	5	5.53934	6
	CASSLQGWATEAFF															2.38729	7
	CASSLGQGEQYF									0.00571	1550					1.37558	8
	CASSEGGYNSPLHF															0.97903	9
	CASSLYRGGTQYF	0.74853	12	0.09585	360			0.04790	121			2.08141	17	0.38626	105	0.93555	10
	SUM FREQUENCIES	**3.65511**		**3.48877**		**3.16797**		**0.05880**		**0.58381**		**4.99537**		**5.31432**		**95.87037**	
	% CLONOTYPES AMONG 10% MOST FREQUENT**		**30**		**10**		**10**		**10**		**10**		**0**		**20**		**100**
	SUM FREQUENCIES CD4 CLONOTYPES	**3.01402**		**2.93315**		**3.07525**						**3.00648**		**1.70635**		**6.47488**	
	SUM FREQUENCIES CD8 CLONOTYPES	**0.53151**		**0.34989**		**0.09271**						**1.61887**		**3.19199**		**45.40853**	

CD4+ clonotypes are depicted in red and CD8+ clonotypes in blue. *Clonotypes identified in the three brain lesions using both gDNA and cDNA sequencing. %, frequency of each clonotype in each sample. Rank, position of the clonotype regarding its frequency compared with all clonotypes in the sample. Filled cells, clonotypes ranking among the 10% most frequent clonotypes in each sample.

Table SIII. Presence in the peripheral blood of multiple sclerosis patients and healthy donors of the 50 T cell clonotypes shared by the three brain lesions.

	Shared CD4+ clonotypes		Shared CD8+ clonotypes	
*	#	ID Table II Frequency(%)	#	ID Table II Frequency(%)
**MS 1**	0		1	Clonotype-27 0.000303
**MS 2**	0		1	Clonotype-27 0.000424
**MS 3**	0		0	
**MS 4**	1	Clonotype-7 0.000097	0	
**MS 5**	0		2	Clonotype-27 0.000171 Clonotype-23 0.000285
**MS 6**	0		1	Clonotype-15 0.000159
**MS 7**	0		1	Clonotype-15 0.000211
**MS 8**	2	Clonotype-7 0.000270 Clonotype-37 0.000108	0	
**MS 9**	0		0	
**MS 10**	0		1	Clonotype-27 0.000351
**MS 11**	1	Clonotype-37 0.001378	0	
**MS 12**	0		0	
**MS 13**	1	Clonotype-7 0.000117	0	
**MS 14**	0		0	
**MS 15**	1	Clonotype-7 0.000225	0	
**MS 16**	0		1	Clonotype-27 0.000167
**MS 17**	1	Clonotype-7 0.000069	1	Clonotype-31 0.000519
**MS 18**	0		1	Clonotype-27 0.000793
**MS 19**	0		0	
**MS 20**	0		0	
**MS 21**	0		0	
**MS 22**	0		0	
**MS 23**	0		2	Clonotype-27 0.000442 Clonotype-40 0.001289
**MS 24**	1	Clonotype-7 0.000067	1	Clonotype-27 0.000101
**MS 25**	0		1	Clonotype-27 0.000475
**Sum %**		All 0.002331 Clonotype-7 0.000845		All 0.005690 Clonotype-27 0.003227
**Mean %**		All 0.000291 Clonotype-7 0.000140		All 0.000406 Clonotype-27 0.000358

	Shared CD4+ clonotypes				Shared CD8+ clonotypes			
	MEMORY		NAIVE		MEMORY		NAIVE	
	#	ID Table II (%)	#	ID Table II (%)	#	ID Table II (%)	#	ID Table II (%)
**HD 1**	0		0		0		0	
**HD 2**	0		0		0		0	
**HD 3**	0		0		0		1	Clonotype-27 0.002189
**HD 4**	1	Clonotype-7 0.002974	0		0		1	Clonotype-43 0.003394
**HD 5**	0		0		0		1	Clonotype-27 0.004354
**HD 6**	0		0		0		1	Clonotype-27

								0.004092
**HD 7**	0		0		0		0	
**HD 8**	0		1	Clonotype-7 0.001665	0		0	
**HD 9**	0		0		0		0	
**HD 10**	0		0		0		0	
**HD 11**	0		0		0		0	
**HD 12**	0		0		0		0	
**HD 13**	0		0		0		0	
**HD 14**	0		0		0		0	
**HD 15**	0		0		0		0	
**HD 16**	0		0		0		0	
**HD 17**	0		0		0		0	
**Sum %**		Clonotype-7 0.002974		Clonotype-7 0.001665				All 0.014029 Clonotype-27 0.010635
**Mean %**		Clonotype-7 0.002974		Clonotype-7 0.001665				All 0.003507 Clonotype-27 0.003545

*MS patients and HD Cohort from Emerson et al., 2013. %, Frequency of the clonotype in the MS or HD T cell pool.

Supplementary Figure Legends

Figure S1. (A) Coefficient of correlation (r) between the number of shared clonotypes (gDNA sequencing) in each pair of brain samples (circles), between brain and mem-CD4+ (triangles) and mem-CD8+ (squares), and the product of the intersected cloneset sizes. **(B)** Logarithmic scatter plot comparing the number of copies of each clonotype identified by gDNA sequencing in two independent DNA extractions from the same sample (brain LIII). Actual and normalized number of shared clonotypes as well as correlation coefficient (r) and coefficient of determination (r^2) are shown in all scatters. Number of clonotypes not shared are also shown in parenthesis. **(C)** Actual and normalized number of clonotypes shared by all samples analyzed in this study. AU, arbitrary units.

Figure S2. (A) Coefficient of correlation (r) between the number of shared clonotypes in each samples using gDNA and cDNA sequencing and the product of the intersected cloneset sizes. **(B)** Coefficient of correlation (r) between the number of shared clonotypes in each pair of brain samples (cDNA sequencing) and the product of the intersected cloneset sizes. **(C)** Linear regression and its equation corresponding to the correlation between the number of shared clonotypes in each pair of brain samples (gDNA sequencing) and the product of the intersected cloneset sizes.

Figure S1

A

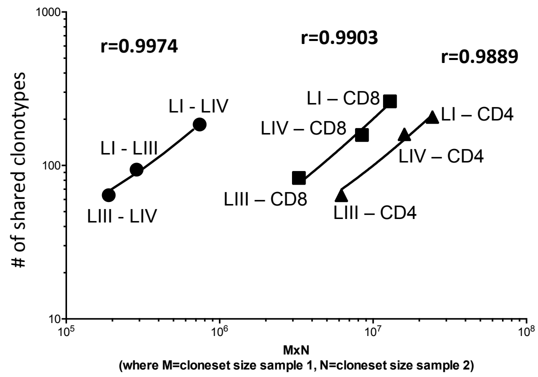

B

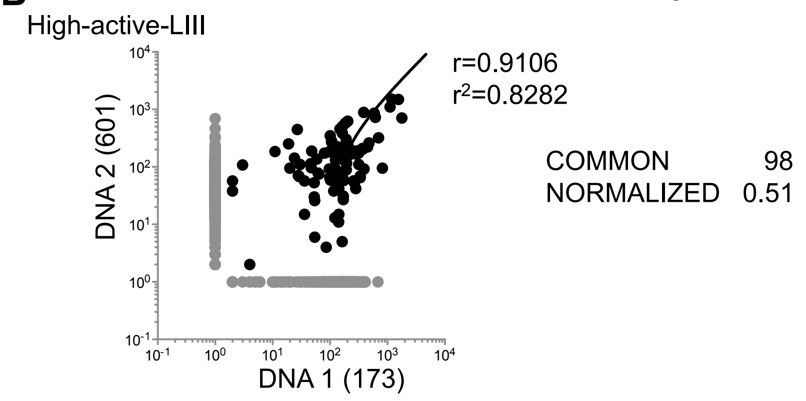

C

	# SHARED CLONOTYPES	NORMALIZED # SHARED CLONOTYPES
LESION III DNA1 - DNA2	98	0.510000
LESION I - LESION III	94	0.326613
LESION I - LESION IV	185	0.248221
LESION III - LESION IV	64	0.338341
LESION I - BIOPSY RE	0	0.000000
LESION I - BIOPSY MS 2	1	0.000458
LESION III - BIOPSY RE	0	0.000000
LESION III - BIOPSY MS 2	0	0.000000
LESION IV - BIOPSY RE	0	0.000000
LESION IV - BIOPSY MS 2	0	0.000000
BIOPSY RE - BIOPSY MS 2	1	0.000616
CD4 - LESION I	207	0.008494
CD4 - LESION III	64	0.010292
CD4 - LESION IV	160	0.009989
CD4 - BIOPSY RE	1	0.000055
CD4 - BIOPSY MS 2	7	0.000149
CD8 - LESION I	262	0.020345
CD8 - LESION III	83	0.025258
CD8 - LESION IV	158	0.018667
CD8 - BIOPSY RE	1	0.000104
CD8 - BIOPSY MS 2	2	0.000080
LESION I - LESION III - LESION IV - BIOPSY RE	0	0.000000
LESION I - LESION III - LESION IV - BIOPSY MS 2	0	0.000000

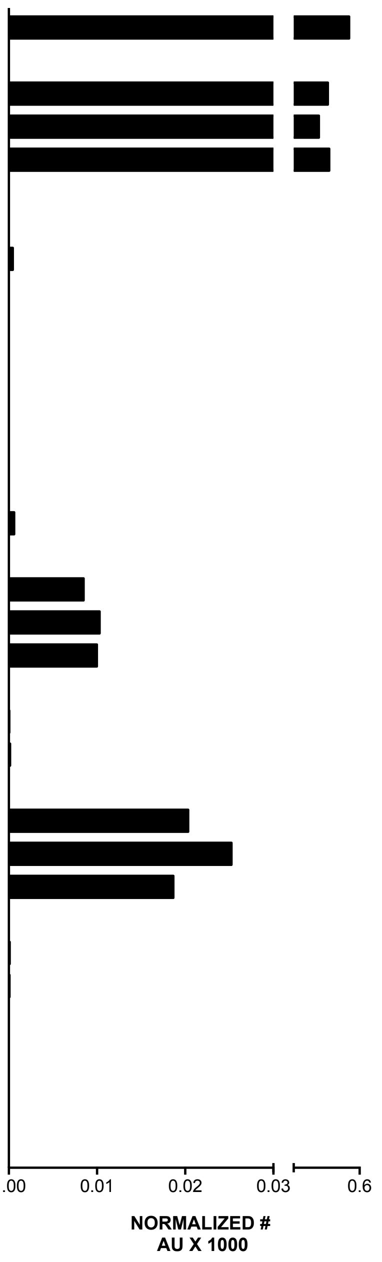

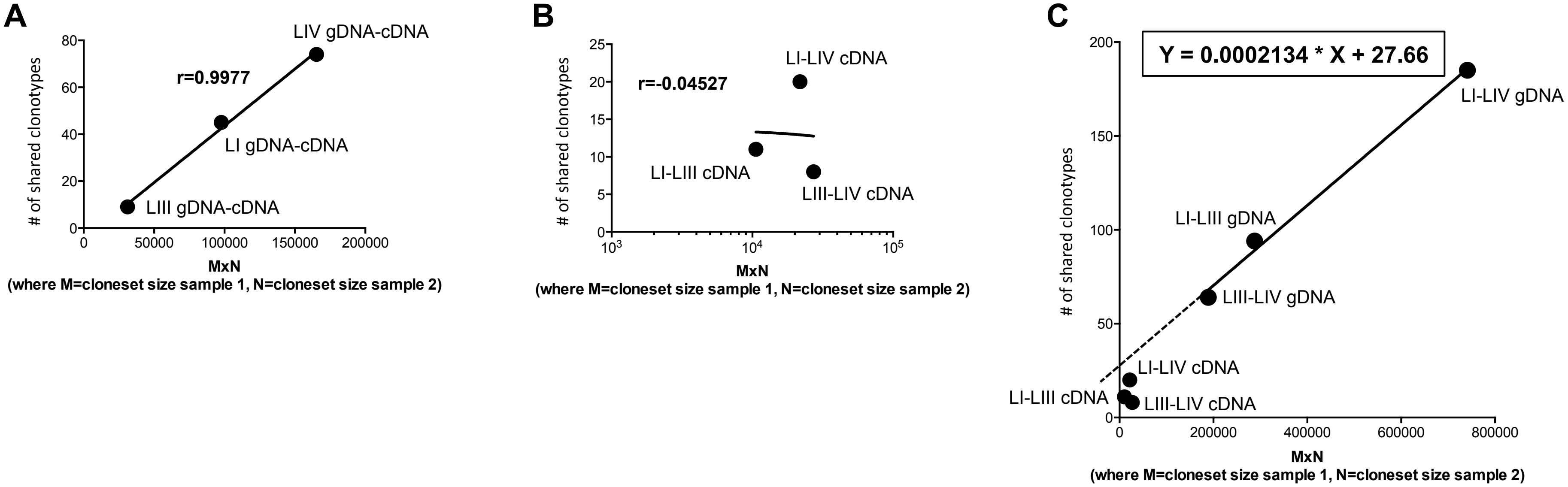
A
r=0.9977
LIV gDNA-cDNA
LI gDNA-cDNA
LIII gDNA-cDNA
# of shared clonotypes
MxN
(where M=cloneset size sample 1, N=cloneset size sample 2)
B
r=-0.04527
LI-LIV cDNA
LI-LIII cDNA
LIII-LIV cDNA
# of shared clonotypes
MxN
(where M=cloneset size sample 1, N=cloneset size sample 2)
C
Y = 0.0002134 * X + 27.66
LI-LIV gDNA
LI-LIII gDNA
LIII-LIV gDNA
LI-LIV cDNA
LI-LIII cDNA
LIII-LIV cDNA
# of shared clonotypes
MxN
(where M=cloneset size sample 1, N=cloneset size sample 2)

Figure S2
